# Lewis acid catalyzed [4+2] annulation of bicyclobutanes with dienol ethers for the synthesis of bicyclo[4.1.1]octanes†

**DOI:** 10.1039/d4sc02767a

**Published:** 2024-05-27

**Authors:** Stefano Nicolai, Jérôme Waser

**Affiliations:** a Laboratory of Catalysis and Organic Synthesis, Institute of Chemical Sciences and Engineering, Ecole Polytechnique Fédérale de Lausanne 1015 Lausanne Switzerland stefano.nicolai@epfl.ch jerome.waser@epfl.ch

## Abstract

Bicyclic carbocycles containing a high fraction of Csp^3^ have become highly attractive synthetic targets because of the multiple applications they have found in medicinal chemistry. The formal cycloaddition of bicyclobutanes (BCBs) with two- or three-atom partners has recently been extensively explored for the construction of bicyclohexanes and bicycloheptanes, but applications to the synthesis of medium-sized bridged carbocycles remained more limited. We report herein the formal [4+2] cycloaddition of BCB ketones with silyl dienol ethers. The reaction occurred in the presence of 5 mol% aluminium triflate as a Lewis acid catalyst. Upon acidic hydrolysis of the enol ether intermediates, rigid bicyclo[4.1.1]octane (BCO) diketones could be accessed in up to quantitative yields. This procedure tolerated a range of both aromatic and aliphatic substituents on both the BCB substrates and the dienes. The obtained BCO products could be functionalized through reduction and cross-coupling reactions.

## Abstract 2

Bicyclo[4.1.1]octanes (BCOs) were synthesized in up to quantitative yields through the formal [4+2] cycloaddition of aryl and alkyl bicyclobutane (BCB) ketones with dienol silyl ethers using Al(OTf)_3_ as a Lewis acid catalyst.

## Graphical abstract

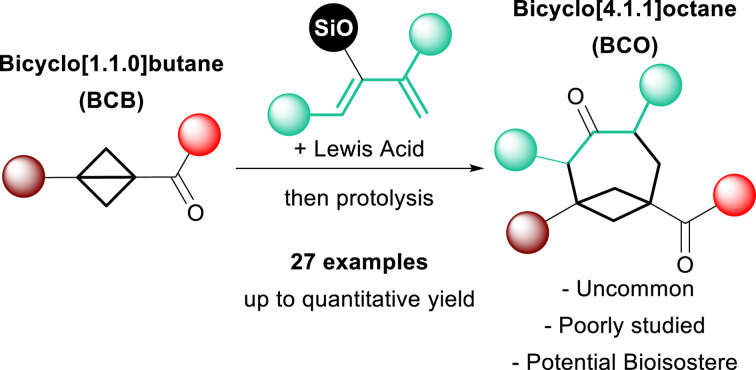

## Introduction

**Scheme 1:**
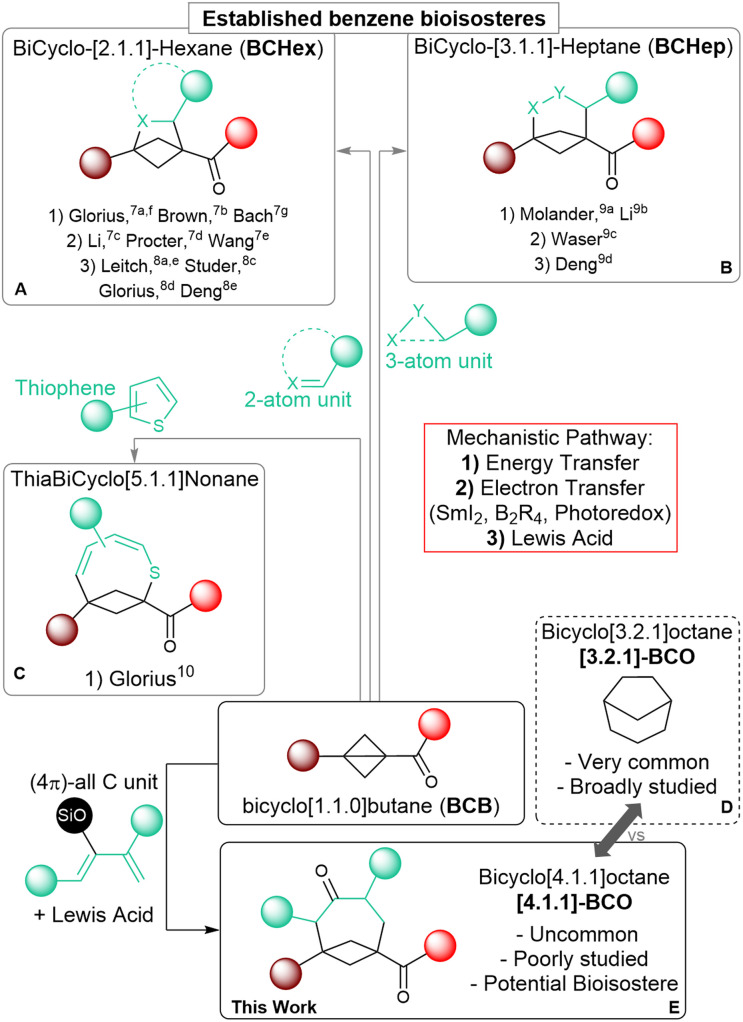
Formal cycloadditions of BCB carbonyl derivatives for: (A) the synthesis of bicyclohexanes; (B) the synthesis of bicycloheptanes; (C) the synthesis of thiabicyclononanes; (D) common bicyclo-[3.2.1]-octane scaffold; (E) this work: the synthesis of all-carbon bicyclo-[4.1.1]-octanes ([4.1.1]-BCO).

Saturated polycyclic carbocycles have gained growing attention in both medicinal and organic chemistry.^1^ Molecules incorporating these motifs exhibit enhanced pharmacokinetic and physiochemical properties compared to more common Csp^2^-rich bioactive synthetic compounds and have become privileged candidates for drug discovery.^2^ The increased conformational rigidity of these polycyclic frameworks is especially important as it can lead to improved affinity to their biological targets, as demonstrated also in many bioactive natural products.^3^ Accordingly, the efficient construction of bicycloalkanes as core elements of more complex systems has become an important goal for synthetic chemists, although it demands addressing the challenges coming from their inherent complexity.^4^ During the last two decades, strain-releasing ring-opening annulation reactions of cyclopropanes, especially donor–acceptor substituted systems (Donor–Acceptor Cyclopropanes, DACs), have been established as a reliable and powerful synthetic tool for the assemblage of larger cyclic systems.^5^ Among cyclopropanes, the even more strained bicyclo[1.1.0]butanes (BCBs) have recently attracted interest, as strongly activating substituents are less needed and more rigid bicyclic carbocycles and their heterocyclic analogs can be obtained.^6^ The synthesis of bicyclo[2.1.1]hexanes (BCHex's) through the formal [2 + 2] cycloaddition of BCBs has been extensively studied to access new bioisosteres of the benzene ring.^1*c*^ Following the seminal reports by the groups of Glorius^7*a*^ and Brown,^7*b*^ several methods have appeared that rely on radical pathways, either under light-induced energy transfer (Scheme 1, (A.1): Glorius,^7*a*,*f*^ Brown,^7*b*^ Bach^7*g*^) or electron-transfer conditions (Scheme 1, (A.2): Li,^7*c*^ Procter,^7*d*^ Wang^7*e*^). Lewis acid catalysis has also proven effective in promoting annulations following a polar mechanism (Scheme 1, (A.3): Leitch,^8*a*,*e*^ Studer,^8*b*^ Glorius,^8*c*^ Deng^8*d*^).

As a recent expansion, the annulation of BCBs with three-atom partners has been used to obtain bicyclo[3.1.1]heptanes (BCHeps) using the same three activation modes (Scheme 1B: Molander,^9*a*^ Li,^9*b*^ Waser,^9*c*^ Deng^9*d*^). Cycloadditions of BCBs affording larger saturated bicycloalkanes have however remained unexplored so far, and only one example exists, in which this kind of transformation is employed to form unsaturated thiabicyclo[5.1.1]nonanes (Scheme 1C; Glorius).^10^

Medium sized carbocycles and their bridged variants are abundant among both natural and pharmacologically relevant compounds.^11^ One example is bicyclo[3.2.1]octane ([3.2.1]-BCO), which represents a conformationally rigid analog of cycloheptane. This scaffold can be found in thousands of bioactive terpenoid derivatives, and extensive research has focused on its synthesis (Scheme 1D).^12^ In comparison, bicyclo[4.1.1]octane (BCO) is rarer in nature,^13^ it has been much less studied, and the very few preparative methods that have been established so far are limited in scope and lack convergence.^14^ In a recent study, the group of Grygorenko showcased the improved lipophilicity of this unique motif and its potential function as an isosteric replacement for both aromatic and saturated monocyclic carbocycles.^14*c*^ Further investigations on BCO ring systems would be of great benefit in the perspective of their applications in medicinal chemistry. Nonetheless, progressing in this direction is hampered by the lack of efficient synthetic methods granting expedient access to these scaffolds.

The annulation of BCBs with four-carbon partners such as dienes appears as an attractive convergent strategy to access BCOs. Such a [4+2] annulation would correspond to an unusual (formal) Diels–Alder cycloaddition, in which the π electrons of the dienophile are not provided by a C=C double bond, but by the single C–C bond of BCBs, which is known to have a significant π character.^6*a*,*d*^ However, dienes can also act as two-carbon partners, leading to the competitive formation of BCHexs. This is especially true when a radical-based mechanism is involved. In previous reports, using weakly or non-polarized dienes under photochemical conditions resulted in the formation of the [2 + 2] BCHex products.^15^ On the other side, the only reported transformation giving access to medium-sized bicyclic scaffolds relied on a photo-induced dearomative expansion of thiophenes.^10^ Therefore, we wondered if Lewis acid catalysis might constitute a more viable alternative. Herein, we describe the synthesis of BCOs through the formal [4+2] cycloaddition of BCB ketones with dienol silyl ethers under Lewis acid catalysis through the successful implementation of this strategy (Scheme 1E). To the best of our knowledge, this is the first application of BCBs to synthesize medium-sized bridged all-carbon carbocycles, and a rare example of their use as dienophiles.^16^

## Results and discussion

### Reaction design and optimization

**Scheme 2:**
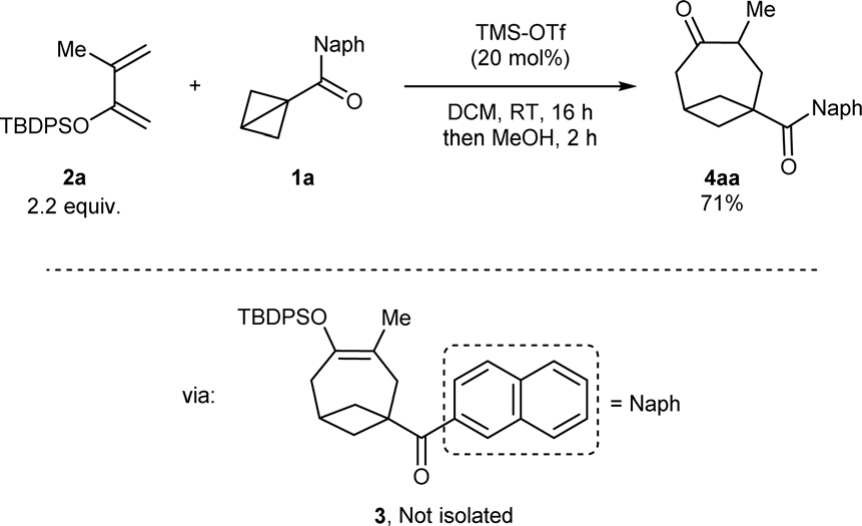
Discovery of the formal [4+2] cycloaddition of BCB ketone 1a with dienol silyl ether 2a to give BCO diketone 4aa through intermediate enol 3.

At the start of our studies, more stable naphthoyl BCB 1a was selected as our model substrate and treated with an excess (2.2 mmol) of *tert*-butyl diphenylsilyl (TBDPS) dienol ether 2a in DCM and in the presence of TMS-OTf (20 mol%, the catalyst reported by Studer for BCB activation^8*b*^) at room temperature (Scheme 2). A check of the reaction after 16 hours showed the full conversion of 1a and the formation of a less polar compound (later identified as silyl enol ether 3). After methanol was added and the resulting mixture was stirred for 2 hours, we observed that 3 had been completely transformed into BCO ketone product 4aa, which could be isolated in 71% yield.‡‡During the optimization, in some cases, isolation of 3 was possible up to *ca.* 90%. See the ESI for details.

Because the purification of the intermediate silyl enol ether was challenging, we focused directly on optimizing the formation of ketone 4aa. As the complete conversion of 3 to 4aa through the sole addition of MeOH was difficult to achieve, an excess of TMS-OTf was used. A screening of silyl protecting groups on the dienol ether using 20 mol% of TMS-OTf as catalyst showed that, compared to TBDPS (Table 1, entry 1) the smaller and less stable TBS (entry 2) and TIPS (entry 3) provided 4aa in lower yield. Ga(OTf)_3_ – the catalyst of choice in the annulation of BCB ketones with imines published by the group of Leitch^8*a*^ – led to an increased yield of over 80% (entry 4). Other Lewis acids furnished inferior results (see the ESI† for details). Reducing the catalyst loading to 10 mol% did not affect the efficiency of the reaction (entry 5). On the contrary, a smaller amount of the dienol ether (1.2 instead of 2.2 equivalents) afforded a significantly diminished yield (entry 6). Al(OTf)_3_ was next investigated as a more sustainable alternative to Ga(OTf)_3_.^17^ No diminution of yield occurred when the reaction was performed using 10 mol% Al(OTf)_3_ (entry 7). Testing other solvents confirmed the superiority of DCM to other chlorinated (entry 8) and non-chlorinated ones (entry 9).§§1,2-Dichloroethane (DCE) was however found to be as good a solvent as DCM (see the ESI). In addition, further lowering the catalyst loading of Al(OTf)_3_ to 5 mol% provided the product in even higher 90% yield (entry 10); this was not the case with Ga(OTf)_3_ (see the ESI†). Finally, the influence of the silyl-deprotecting work-up after the formal cycloaddition step was investigated. To ensure reproducibility, the scale of the process was doubled to 0.30 mmol ketone 1a. Treatment with TBAF upon solvent-switch to THF gave inferior results (entry 11) compared to the protocol involving the addition of TMS-OTf and MeOH (entry 12), which was therefore adopted as our optimal procedure. Changing TMS-OTf to methanolic HCl provided 4aa in a comparable yield (entry 13), and can be thus considered as a more cost-effective alternative.

**Table 1:** Optimization of the [4+2] annulation of BCB ketone 1a with dienol silyl ether 2a^a^

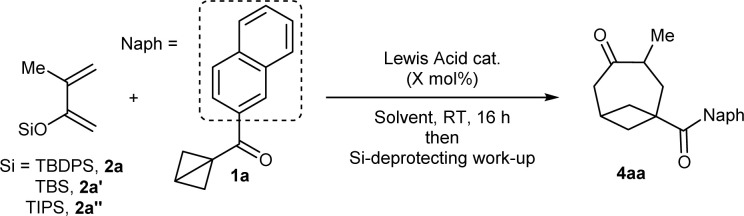				
Entry	Si group	Lewis acid (*X* mol%)	Solvent	Yield^b^
1	TBDPS	TMS-OTf (20)	DCM	70%
2	TBS	TMS-OTf (20)	DCM	33%
3	TIPS	TMS-OTf (20)	DCM	52%
4	TBDPS	Ga(OTf)_3_ (20)	DCM	83%
5	TBDPS	Ga(OTf)_3_ (10)	DCM	81%
6^c^	TBDPS	Ga(OTf)_3_ (10)	DCM	61%
7	TBDPS	Al(OTf)_3_ (10)	DCM	84%
8	TBDPS	Al(OTf)_3_ (10)	CHCl_3_	75%
9	TBDPS	Al(OTf)_3_ (10)	Et_2_O	57%
10	TBDPS	Al(OTf)_3_ (5)	DCM	90%
11^d^^,^^e^	TBDPS	Al(OTf)_3_ (5)	DCM	74%
12^d^^,^^f^	TBDPS	Al(OTf)_3_ (5)	DCM	82%
13^d^^,^^g^	TBDPS	Al(OTf)_3_ (5)	DCM	78%

a Reaction conditions: 0.15 mmol BCB ketone 1a (1.0 equiv.), 0.33 mmol silyl dienol ether 2a–a′′ (2.2 equiv.), Lewis acid (*X* mol%), in 1.5 mL solvent (0.1 M) at RT, overnight; work-up: 1.5 mL MeOH, 0.10 mL TMS-OTf (6.0 mmol, 4.0 equiv.), at RT, 4 hours.

b Isolated yield upon column chromatography.

c With 0.36 mmol 2a (1.2 equiv.).

d 0.30 mmol BCB ketone 1a (1.0 equiv.), 0.66 mmol silyl dienol ether 2a (2.2 equiv.), Lewis acid (*X* mol%), in 3.0 mL solvent (0.1 M), at RT, 2 hours.

e Upon removal of DCM: 0.75 mL TBAF (1.0 M in THF, 2.5 equiv.) in 3.0 mL THF, 0 °C to RT, 4 hours.

f Work-up: addition of 3.0 mL MeOH, 0.20 mL TMS-OTf (12 mmol, 4.0 equiv.), at RT, 4 hours.

g Work-up: addition of 2.6 mL MeOH, 0.40 mL HCl (3.0 M in MeOH, 4.0 equiv.), at RT, 2 hours.

### Applicability of the reaction

**Scheme 3:**
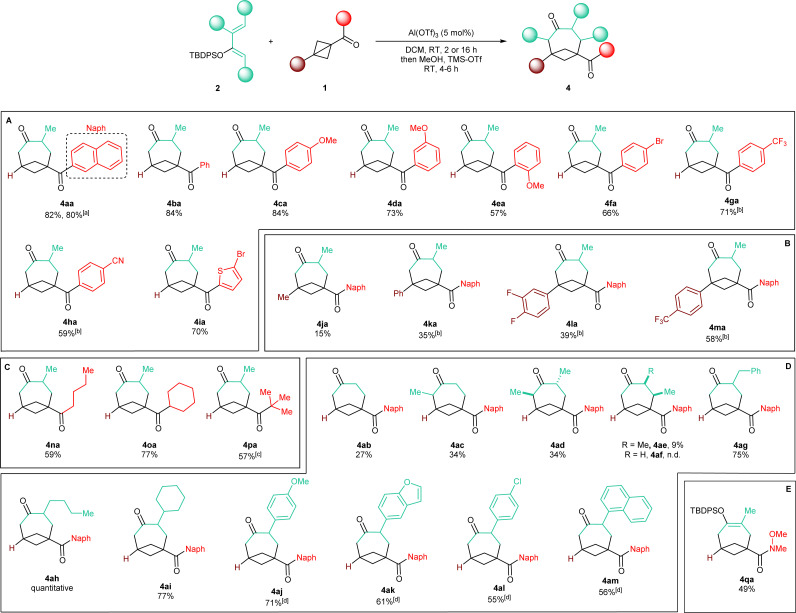
Scope of the reaction. Products obtained with: (A) diverse aryl BCB ketones 1a–1I; (B) BCB ketones bearing a substituent at the bridgehead position 1j–1m; (C) diverse alkyl BCB ketones 1n–1p; (D) diversely substituted TBDPS dienol ethers 2a–2l; (E) BCB Weinreb amide 1q. General conditions: 0.30 mmol (1.0 equiv.) BCB Ketone 1, 0.66 mmol (2.2 equiv.) TBDPS dienol ether 2, 5 mol% Al(OTf)_3_, 3.0 mL DCM (0.1 M), RT, 2 hours; then: 3.0 mL MeOH, 12 mmol TMS-OTf (4.0 equiv.), RT, 4–6 hours. ^a^Performed on a 1.5 mmol scale. ^b^The reaction was run overnight. ^c^Average yield over two reiterations. ^d^With 0.60 mmol (2.0 equiv.) TBDPS dienol ether 2, overnight; for the quench 1.2 mmol TMS-OTf (8.0 equiv.) were used.

With an optimized protocol in hand, we then assessed the generality of our method (Scheme 3). We started by considering variations of the BCB ketone substrates. Aryl-substituted BCB ketones were initially studied (Scheme 3A). A further five-fold scale-up of the reaction to 1.5 mmol of 2a produced 4aa in 80% yield, demonstrating the excellent reproducibility of the procedure. Phenyl ketone 1b afforded BCO 4ba in 84% yield. An electron-donating methoxy substituent on the aromatic ring was also tolerated in both the *para* (4ca, 84% yield) and the *meta* (4da, 73% yield) positions. With an *ortho* OMe group, BCO 4ea was isolated in 57% yield.

**Fig. 1:**
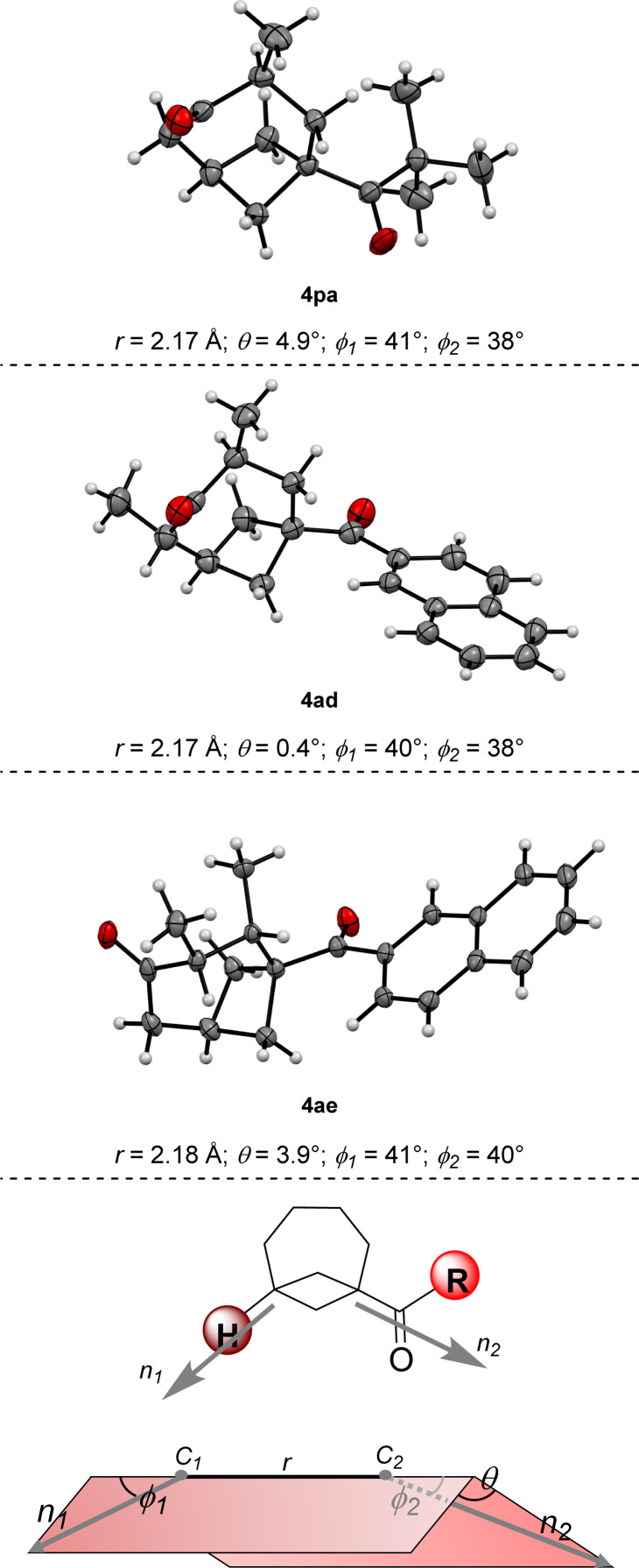
X-Ray diffraction of BCO derivatives 4pa, 4ad and 4ae and corresponding values of the geometrical parameters associated with exit vectors *n*_1_ and *n*_2_; geometrical definition of exit vectors and associated parameters.

Electron-withdrawing substituents were also compatible, albeit longer reaction times were necessary: substrates having a bromine atom, a trifluoromethyl, or a nitrile in the *para* position of the aryl group gave BCO derivatives 4g–4ia in 60–70% yield. Thiophene-containing BCB 1i gave 4ia in 70% yield. Then, BCBs with substituents on the bridgehead of the bicycle were examined (Scheme 3B). A methyl was poorly tolerated as product 4ja was obtained in only 15% yield. BCB 1k, containing a phenyl at the bridgehead carbon, was converted into 4ka in 35% yield. In the presence of more electron-poor 3,4-difluorophenyl and 4-trifluoromethyl phenyl groups, products 4la and 4am were generated in 39% and 58% yields. Finally, alkyl BCB ketones were also good starting materials (Scheme 3C): a primary ^*n*^butyl, a secondary and cyclic cyclohexyl, and a tertiary ^*t*^butyl groups on the carbonyl of the substrate were all tolerated, furnishing aliphatic BCO 4na, 4oa and 4pa in 59%, 77% and 57% yields, respectively. X-ray diffraction of 4pa gave a confirmation of the caged bicyclic framework of the synthesized BCO derivatives.¶¶Deposition number for 4oa: 2312246; contains the supplementary crystallographic data for this paper. These data are provided free of charge by the joint Cambridge Crystallographic Data Centre and Fachinformationszentrum Karlsruhe Access Structures service https://www.ccdc.cam.ac.uk/structures.Fig. 1 displays the ORTEP models of 4pa and of the other BCOs that could successfully be submitted to crystallographic analysis (*v. infra*). The values of the geometrical parameters *r*, *θ*, *φ*_1_, *φ*_2_ associated with the corresponding exit vectors are provided as well. As noted by Grygorenko and co-workers,^14*c*^ all the values fit in the *β* region of the exit vector plot and therefore can be considered as good mimics of *meta*-substituted benzenes and *cis*-1,3-disubstituted cyclohexane derivatives, assuming that substitution of the hydrogen at the ring junction would not change the bond angles dramatically.||||The calculation of these values was performed as follows: cif files were converted to xyz files with the Cell2Mol online tool https://cell2mol.materialscloud.io/, doi: https://doi.org/10.1038/s41524-022-00874-9. Angles were measured with GaussView 6.0.16. https://gaussian.com/citation/, GaussView, Version 6, Dennington, Roy; Keith, Todd A.; Millam, John M. Semichem Inc., Shawnee Mission, KS, 2016.

We then turned our interest towards varying the TBDPS dienol ether in the reaction with 1a (Scheme 3D). Unsubstituted 2b and 1-methyl substituted 2c both gave corresponding BCO derivatives, 4ab and 4ac, in modest yields (27% and 34%). Diene 2d – containing methyl groups in both C1 and C3 – also gave a moderate yield but – interestingly – product 4ad was formed as a single *trans* diastereoisomer, as determined by X-ray diffraction (Fig. 1).**Deposition number for 4ad: 2333992; contains the supplementary crystallographic data for this paper. These data are provided free of charge by the joint Cambridge Crystallographic Data Centre and Fachinformationszentrum Karlsruhe Access Structures service https://www.ccdc.cam.ac.uk/structures. Analogously, only one diastereoisomer was obtained starting from dienol silyl ether 2e, with vicinal Me in C3 and C4. Crystallographic analysis allowed us to establish that product 4ae was assembled with a *cis* relative configuration (Fig. 1).††††Deposition number for 4ae: 2356953; contains the supplementary crystallographic data for this paper. These data are provided free of charge by the joint Cambridge Crystallographic Data Centre and Fachinformationszentrum Karlsruhe Access Structures service https://www.ccdc.cam.ac.uk/structures. It should be remarked, however, that the presence of an alkyl group in C4 led to a dramatic diminution of yield, as 4ae was isolated in only 9% yield and non-cyclic addition products were instead dominant (*v. infra*). Without a substituent in C3, the desired BCO 4af was not detected, and only a mixture of non-annulated cyclobutane derivatives was observed instead.

With one substituent in the C3 position, the reaction worked consistently well with different substituents. Alkyl groups were all tolerated: benzyl-containing BCO 4ag was synthesized in high 75% yield, whereas 4ah and 4ai – with ^*n*^butyl and cyclohexyl groups – were delivered quantitatively and in 77% yield. With aryl C3-substituted dienes, readjusting the procedure was necessary: a slightly decrease in the amount of the dienol ether to 2.0 equivalents was possible, but a longer reaction time was needed, together with a larger excess of TMS-OTf during the silyl-deprotecting work-up. With diene 2j bearing an electron-rich *p*-anisyl group in C3, 4aj was formed in 71% yield. Heterocyclic diene 2k gave benzofuran-substituted BCO 4ak in 61% yield. Slightly lower yields were obtained with dienol silyl ethers bearing less electron-donating aryl substituents: 4al and 4am were accessed in 55% and 56% yields.

As a last example, Weinreb amide 1q was also examined as a non-ketone substrate (Scheme 3E). Compared to the previously studied BCB ketones, 1q reacted more slowly, requiring a reaction time of 48 hours in order to achieve full conversion. Enol silyl ether cycloadduct 4qa could be isolated in 49% yield in satisfactory purity directly after the annulation step. This is particularly convenient because the further functionalization of the amide group might be envisaged while the endocyclic ketone carbonyl remains protected in its enol form.

### Speculative mechanism

**Scheme 4:**
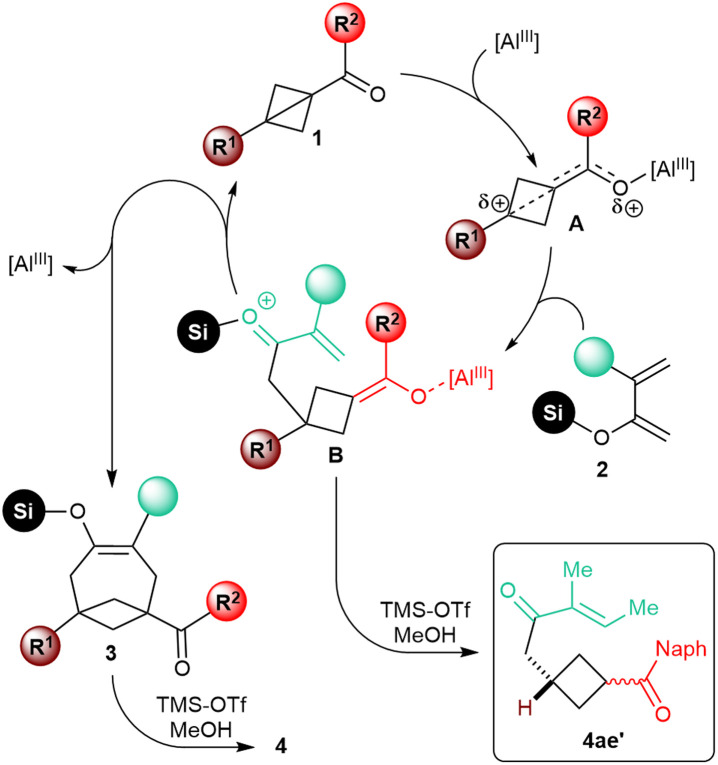
Speculative mechanism of the Lewis acid-catalyzed [4+2] annulation of carbonyl BCBs 1 with dienol silyl ethers 2.

We did not perform any in-depth mechanistic study. Based on the analogy with previously described Lewis acid-catalyzed annulative transformations of carbonyl BCB substrates,^6*f*^ it appears nonetheless plausible that, upon coordination to the catalyst, the resulting activated intermediate A would undergo nucleophilic attack of the enol moiety of the diene at the bridgehead position (Scheme 4). After this initial C–C bond-forging step, the resulting intermediate B would cyclize by conjugate intramolecular addition of the enolate onto the enone to give silyl enol ether 3. The sensitivity of the reaction to the substitution at the C4 position of the diene would be in agreement with this mechanistic speculation, as steric hindrance would slow down this second step of the annulation process. In fact, enone 4ae′, putatively generated upon protolysis of intermediate B, was isolated as the major product in the annulation of 1a with diene 4e.

### Product modifications

**Scheme 5:**
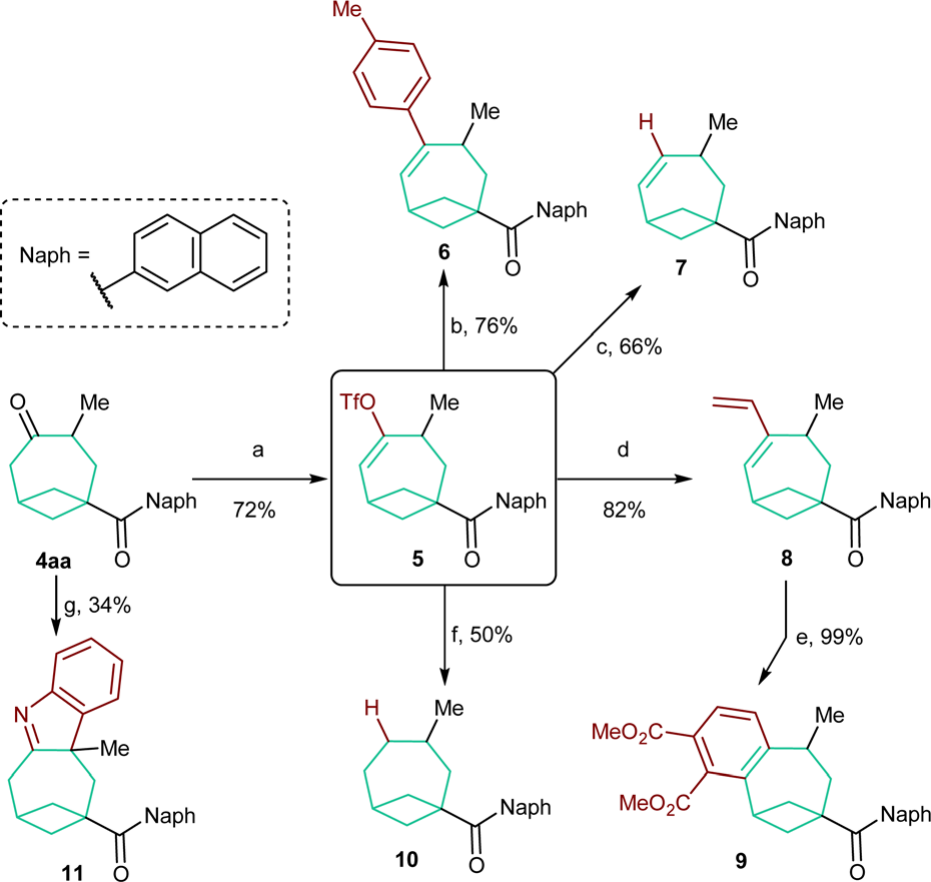
Modifications of BCO product 4aa. Reaction conditions: (a) KHMDS, PhNTf_2_, THF, −78 °C. (b) Pd(dppf)Cl_2_ (6 mol%), K_3_PO_4_, *p*-TolB(OH)_2_, THF, 65 °C. (c) Pd(PPh_3_)_4_ (2 mol%), LiCl, Bu_3_SnH, THF, RT. (d) Pd(PPh_3_)_4_ (2.5 mol%), LiCl, Bu_3_SnCH=CH_2_, THF, 65 °C. (e) DMAD, PhCH_3_ 120 °C then DDQ, 120 °C. (f) H_2_ (1 atm), Pd/C (5 mol%), Li_2_CO_3_, EtOAc, RT. (g) PhNHNH_2_, HCl, MeOH, 90 °C (MW).

In order to evaluate the synthetic versatility and utility of the obtained BCO diketones, their modifications were then investigated (Scheme 5). We focused on BCO 4aa, containing a naphthyl group on the carbonyl function. In this case, chemoselective functionalization is facilitated as only the carbonyl group on the bicyclooctane scaffold is enolizable. Accordingly, 4aa was smoothly converted into enol triflate 5 in 72% yield under kinetically controlled conditions.^18^ This compound was the starting material for a series of subsequent transformations. Styrene 6 and alkene 7 were both accessed in good yields through a Suzuki cross-coupling reaction^19^ and, respectively, a Pd^0^-catalyzed reduction with Bu_3_SnH.^20^ A Stille coupling allowed the synthesis of diene 8.^21^ Refluxing the latter with DMAD followed by oxidation with DDQ permitted benzene-fused product 9 to be forged quantitatively. The completely reduced, saturated skeleton of bicycle[4.1.1]octane could be accessed by catalytic hydrogenation of 10 in the presence of Li_2_CO_3_.^22^ Unfortunately, a yield higher than 50% could not be obtained because of its sensitivity to overreduction. Finally, an interrupted Fischer indole synthesis performed on 4aa provided tetraheterocycle 11,^23^ which represents a further example of a derivative directly obtainable from BCO cycloadducts.

## Conclusions

In summary, the first formal [4+2] cycloaddition of BCB ketones with dienol silyl ethers has been disclosed. The reaction occurred under mild conditions, using commercially available Al(OTf)_3_ as a Lewis acid catalyst, and represents a convenient modular method for the synthesis of uncommon biyclo[4.1.1]octane carbocycles. The latter could be generally obtained in good to very good yields, with a wide tolerance of substituents on both the substrate and the diene, including alkyl as well as electron-rich and -poor aryl groups. The obtained products were available for an array of further transformations, giving access to BCO derivatives with different fractions of Csp^3^. As the importance of biyclo[4.1.1]octanes has started emerging in the search for new bridged cycloalkanes with bioisosteric properties, we believe that our protocol for the expedient preparation of these intriguing frameworks will contribute to facilitating and accelerating research on them.

## Data availability

Experimental procedures, characterization data and scan of NMR spectra. Crystallographic data are available at CCDC (see notes ¶, *, and ††). Raw data for compound characterization, including NMR, IR and MS are available at https://zenodo.org/: https://doi.org/10.5281/zenodo.11264090.

## Author contributions

SN and JW conceived the project, SN run the experiments, SN and JW wrote the manuscript.

## Conflicts of interest

There are no conflicts to declare.

## Supplementary Material

SC-015-D4SC02767A-s001

SC-015-D4SC02767A-s002
