# Supplementary material for: Lewis acid catalyzed [4+2] annulation of bicyclobutanes with dienol ethers for the synthesis of bicyclo[4.1.1]octanes
Source: Chem Sci. 2024 May 27;15(28):10823–9. doi: 10.1039/d4sc02767a (PMC11253158; doi:10.1039/d4sc02767a)

Electronic Supplementary Material (ESI) for Chemical Science.
This journal is © The Royal Society of Chemistry 2024

Lewis Acid Catalyzed [4+2] Annulation of Bicyclobutanes with Dienol Ethers for the Synthesis of Bicyclo[4.1.1]octanes

Tables of Contents

**Experimental Procedures**		S-1
**1.**	**Synthesis of the Starting Materials**	S-3
**1.1**	**Synthesis of the BCB ketones**	S-3
**1.2**	**Synthesis of the dienol ethers**	S-27
**2.**	**Discovery and Optimization of the annulation reaction**	S-47
**2.1**	**Discovery of the annulation reaction**	S-47
**2.2**	**Optimization of the annulation reaction**	S-48
**2.3**	**Adjustment of the silyl-deprotecting work-up**	S-50
**2.4**	**Scale-up of the annulation reaction**	S-52
**3.**	**Scope of the annulation reaction**	S-53
**4.**	**Product modifications**	S-72
**5.**	**Refereces**	S-77
**6.**	**Crystallographic Data**	S-7832
**7.**	**NMR Spectra of new compounds**	S-106

Experimental Procedures

The NMR spectra were recorded on a Brucker DPX-400 spectrometer at 400 MHz for 1H, 101 MHz for 13C, 376 MHz for 19F. The chemical shift (δ) for ^{1}H and ^{13}C are given in ppm relative to residual signals of the solvents (chloroform-*d* - 7.26 ppm ^{1}H NMR and 77.12 ppm ^{13}C NMR; methylene chloride-d_2 5.32 ppm ^{1}H NMR and 53.8 ppm ^{13}C NMR; DMSO-d_6 2.50 ppm ^{1}H NMR and 39.52 ppm ^{13}C NMR). ^{13}C and ^{19}F spectra have been measured using broadband {1H} decoupling. Coupling constants are given in Hertz. The following abbreviations are used to indicate the multiplicity: s, singlet; d, doublet; q, quartet; m, multiplet or massive; bs, broad signal). Infrared spectra of selected compounds were recorded on a JASCO FT-IR B4100 spectrophotometer with an ATR PRO410-S and a ZnSe prisma and are reported as cm-1 (w = weak, m = medium, s = strong, br = broad). High resolution mass spectrometric measurements were performed by the mass spectrometry service of ISIC at the EPFL on a MICROMASS (ESI) Q-TOF Ultima API. The raw data obtained from the Q-TOF Waters instrument does not take into account the mass of the electron for the ion, the obtained raw data has been therefore corrected by removing the mass of the electron (5 mDa). Mass spectrometry for CEC reactions was performed on UPLC-MS system consisting of a Waters

Acquity UPLC and a Waters VION IMS QTOF. Samples were analyzed using Waters Acquity-I-UPLC Classsystem (Waters Corporation, Milford, MA, USA) coupled with a Waters Vion IMS-QTof Mass Spectrometer equipped with LockSpray. The instrument was controlled by Waters UNIFI 1.9.4 (3.1.0, Waters Corporation, Milford, MA, USA). The diffraction data for crystal structures were collected by mass spectrometry service of ISIC at the EPFL at low temperature using Cu (323) or Mo (520) Ka radiation on a Rigaku SuperNova dual system in combination with Atlas type CCD detector. The data reduction and correction were carried out by CrysAlisPro (Rigaku Oxford Diffraction, release 1.171.40.68a, 2019). The solutions and refinements were performed by SHELXT1 and SHELXL2, respectively. The crystal structures were refined using full-matrix least-squares based on F2 with all non-H atoms defined in anisotropic manner. Hydrogen atoms were placed in calculated positions by means of the “riding” model. Yields of isolated products refer to materials of >95% purity as determined by ^{1}H NMR, unless otherwise stated.

The authors are indebted to the team of the research support service of ISIC at EPFL, particularly to the NMR, X-Ray, and the High Resolution Mass Spectrometry Units.

General Procedures. All reactions were set up under a nitrogen atmosphere in oven-dried glassware using standard Schlenk techniques, unless otherwise stated. Chromatographic purification of products was accomplished using flash chromatography (FC) on SilicaFlash P60 silica gel (230 - 400 mesh). When indicated, an automatized flash chromatographer Biotage Isolera One was used to perform purifications by column chromatography. In such a case, Büchi FlashPure EcoFlex Silica cartridges of different sized (4, 12, 25, 40, 80 or 120 g) were employed as solid phases. For thin layer chromatography (TLC) analysis throughout this work, Pre-coated Suplaco silica gel 60 F254 TLC glass plates were employed, using UV light as the visualizing agent and acidic ethanolic *p*-anisaldehyde or basic aqueous potassium permanganate ($KMnO_4$) stain solutions, and heat as developing agents. Organic solutions were concentrated under reduced pressure on a Büchi rotatory evaporator.

Materials. Most of the starting materials used in this study are commercial and were purchased in the highest purity available from Sigma-Aldrich, Fluka, Alfa Aesar, Fluorochem. Synthesis grade solvents were used as purchased; anhydrous solvents (THF, Et_2O, Toluene, DCM and MeCN) were taken from a commercial SPS solvent dispenser (H_2O content < 10 ppm, Karl-Fischer titration).

1. Synthesis of the Starting Materials

1.1 Synthesis of the BCB ketones

3-(Methoxy(methyl)carbamoyl)cyclobutyl p-toluenesulfonate (**S12**)

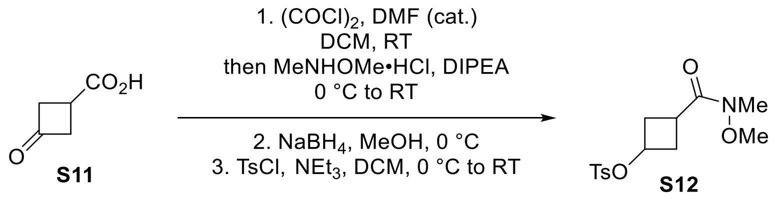

Following a reported procedure,[1] in a 500 mL, two-necked, round-bottomed flask, 3-oxocyclobutanecarboxylicacid (**S11**) (5.70 g, 50.0 mmol, 1.0 equiv.) was dissolved in DCM (200 mL). The clear, pale yellow solution was cooled to 0 °C (ice-water bath). Oxalyl chloride (4.7 mL, 56 mmol, 1.1 equiv.) was added slowly, followed by 15 drops of DMF. The clear mixture was stirred at room temperature for 8 hours. After this time, the pale yellow solution was cooled back to 0 °C, and MeNH(OMe)·HCl (5.4 g, 56 mmol, 1.1 equiv.) and DIPEA (31 mL, 177 mmol, 3.5 equiv.) were slowly added (fuming!!). The mixture turned to dark orange. It was then stirred overnight, allowing it to warm to room temperature. After 15 hours, the reaction was quenched by addition of sat. aq. $NaHCO_3$ (100 mL). Upon separation, the organic layer was washed with aq. HCl (1.0 M, 2 x 80 mL) (it turned from orange to yellow), brine, dried over Na_2SO_4, filtered, and concentrated under vacuum.

The resulting orange-brown crude oil was dissolved in MeOH (190 mL). The resulting orange solution was cooled to 0 °C (ice - water bath). $NaBH_4$ (2.11 g, 55.7 mmol, 1.1 equiv.) was then added in four portions (ca 0.530 g each) under stirring over 40 minutes, with visible release of gas. During the addition, the solution turned from orange to pale yellow. It was then concentrated under reduced pressure. The resulting pale yellow slurry was diluted with water (100 mL). The aqueous layer was then extracted with EtOAc (4 x 100 mL). The combined organic layers were washed with brine (1 x 600 mL), dried over Na_2SO_4, filtered, and concentrated under vacuum to provide a pale yellow crude oil.

Finally, the crude oil obtained from the previous step was dissolved in DCM (190 mL). The yellow solution was cooled to 0 °C (ice - water bath) and Et_3N (8.4 mL, 60 mmol, 1.2 equiv.) was added, followed by tosyl chloride (10.5 g, 55.0 mmol, 1.1 equiv.). The mixture was then stirred over the weekend, while allowing it to gradually warm to room temperature. The mixture turned to bright yellow while being stirred for 54 hours. The reaction was then quenched by addition of water (100 mL). The aqueous layer was separated and extracted with DCM (3 x 100 mL). The combined organic layers were washed with sat. aq. NH_4Cl (100 mL), brine, dried over Na_2SO_4, filtered, and concentrated under vacuum. The resulting orange-brown oil was

submitted to column chromatography (Biotage flash chromatographer; SiO_2; MeOH in DCM, 1 to 12%) to provide 3-(methoxy(methyl)carbamoyl)cyclobutyl *p*-toluenesulfonate (**S12**) (5.31 g, 16.9 mmol, 34% yield over 3 steps) as a very viscous yellow oil, which became a solid on standing at 4 °C.

^{1}H NMR (400 MHz, Chloroform-*d*) δ 7.78 (d, *J* = 8.3 Hz, 2H, Ar*H*), 7.33 (d, *J* = 8.0 Hz, 2H, Ar*H*), 4.81 (p, *J* = 7.7 Hz, 1H, OC*H*), 3.61 (s, 3H, CH_3), 3.15 (s, 3H, CH_3), 2.93 (dt, *J* = 16.3, 8.1 Hz, 1H, (CO)C*H*), 2.44 (s, 3H, ArCH_3), 2.43 – 2.39 (m, 4H, CH_2).
^{1}H-NMR signals correspond to the ones reported in the literature.[1]

N-Methoxy-N-methylbicyclo[1.1.0]butane-1-carboxamide (1q)

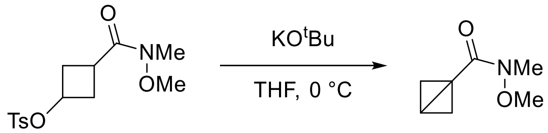

S12 **1q**

In a 100mL, round-bottomed, two-necked flask, 3-(methoxy(methyl)carbamoyl)cyclobutyl 4-methylbenzenesulfonate (**S12**) (1.51 g, 4.82 mmol, 1.0 equiv.) was dissolved in THF (dry; 32 mL). A big stirring bar was present in the flask to ensure a vigorous and constant stirring. The clear, colorless mixture was cooled to 0 °C (ice - water bath), prior to the addition of a solution of potassium tert-butoxide (0.92 g, 8.2 mmol, 1.7 equiv.) in THF (5.1 mL) in one single portion. Immediately, the mixture became milky. It was stirred at 0 °C for 20 minutes. The reaction was then quenched by addition of sat. aq. $NaHCO_3$ (25 mL). The aqueous layer was separated and extracted with Et_2O (3 x 40 mL). The combined organic layers were washed with brine, dried over Na_2SO_4, filtered, and concentrated under vacuum (heating bath temperature: < 40 °C). The resulting pale yellow, crude oil was submitted to column chromatography (Biotage flash chromatographer, SiO_2; Et_2O in pentane, 20 to 80%) to provide N-methoxy-N-methylbicyclo[1.1.0]butane-1-carboxamide (**1q**) (0.576 g, 4.08 mmol, 85% yield) as a pale yellow oil, which was immediately dissolved dissolved in THF (4.1 mL; 1.0 M solution) and stored in a freezer (-60 °C).

^{1}H NMR (400 MHz, Chloroform-*d*) δ 3.72 (s, 3H, CH_3), 3.24 (s, 3H, CH_3), 2.38 (d, *J* = 3.3 Hz, 2H, CH_2), 2.13 (p, *J* = 3.1 Hz, 1H, C*H*), 1.13 (d, *J* = 2.5 Hz, 2H, CH_2).
^{1}H-NMR signals correspond to the ones reported in the literature.[1]

Bicyclo[1.1.0]butan-1-yl(naphthalen-2-yl)methanone (1a)

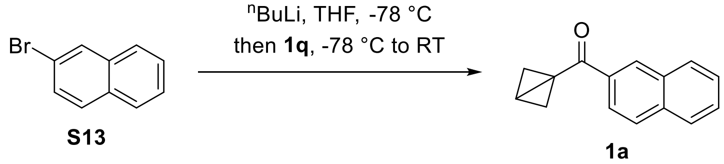

Following a reported procedure,[2] in a 50 mL, two-necked, round-bottomed flask, 2-bromonaphthalene (**S13**) (0.833 g, 4.02 mmol, 1.15 equiv.) was dissolved in THF (dry; 18.1 mL). The clear, colorless solution was chilled to -78 °C (dry ice - water bath). ^{n}BuLi (2.5 M in hexanes; 1.5 mL, 3.7 mmol, 1.05 equiv.) was then added drop-wise: the resulting mixture became yellow at first, and then turbid remaining yellow. The suspension was stirred at -78 °C for 45 minutes. A solution of N-methoxy-N-methylbicyclo[1.1.0]butane-1-carboxamide (**1q**) (3.5 mL of a 1.0 M solution in THF; 3.5 mmol, 1.0 equiv.) was added slowly at the same temperature. It was stirred at -78 °C for 30 minutes and then at room temperature for another 30 minutes. To the now clear, yellow reaction mixture was then added sat. aq. $NaHCO_3$ (15 mL). The aqueous layer was separated and extracted with Et_2O (4 x 40 mL). The combined organic layers were washed with brine, dried over Na_2SO_4, filtered, and concentrated under vacuum (heating bath at < 40 °C (!!)). The resulting pale yellow crude oil was submitted to column chromatography (Biotage flash chromatographer, SiO_2; EtOAc in pentane, 5 to 40%) to provide bicyclo[1.1.0]butan-1-yl(naphthalen-2-yl)methanone (**1a**) (0.40 g, 1.9 mmol, 54% yield) as a colorless solid, which was stored at -60 °C.

^{1}**H NMR** (400 MHz, Chloroform-*d*) δ 8.41 (s, 1H, Ar*H*), 7.97 – 7.86 (m, 4H, Ar*H*), 7.62 – 7.52 (m, 2H, Ar*H*), 2.70 (d, *J* = 3.5 Hz, 2H, C*H$_2$*), 2.25 (p, *J* = 3.3 Hz, 1H, C*H*), 1.55 (d, *J* = 3.1 Hz, 2H, C*H$_2$*).
^{1}H-NMR signals correspond to the ones reported in the literature.[2]

Bicyclo[1.1.0]butan-1-yl(phenyl)methanone (1b)

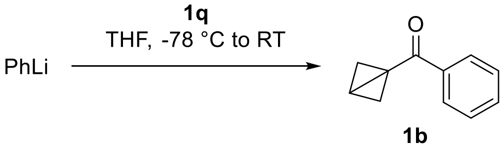

Following a slightly modified version of a reported procedure,[2] in a 50 mL, two-necked, round-bottomed flask, phenyl lithium (1.9 M in dibutyl ether; 1.6 mL, 3.0 mmol, 1.2 equiv.) was diluted with THF (dry; 11 mL). The pale brown solution was chilled to -78 °C (dry ice - water bath). A solution of N-methoxy-N-methylbicyclo[1.1.0]butane-1-carboxamide (**1q**) (0.35 g, 2.5 mmol, 1.0 equiv.) in THF (10 mL) was added slowly at the same temperature. The clear solution turned to pale yellow. TLC analysis (pentane/Et_2O 1/1) showed that the reaction was complete

with full conversion after 30 minutes. The reaction was then quenched by addition of sat. aq. $NaHCO_3$ (25 mL). The aqueous layer was separated and extracted with DCM (3 x 20 mL). The combined organic layers were washed with brine, dried over Na_2SO_4, filtered, and concentrated under vacuum (heating bath at < 40 °C (!!)). The resulting pale yellow crude oil was submitted to column chromatography (SiO_2; Pentane/Et_2O 10/1 to 9/2) to provide bicyclo[1.1.0]butan-1-yl(phenyl)methanone (**1b**) (0.30 g, 1.9 mmol, 76% yield) as a pale yellow oil, which was immediately diluted in DCM (7.5 mL) to provide a 0.25 M solution that was stored at -60 °C

^{1}H NMR (400 MHz, Chloroform-*d*) δ 7.91 – 7.80 (m, 2H, Ph*H*), 7.52 (m, 1H, Ph*H*), 7.47 – 7.39 (m, 2H, Ph*H*), 2.63 (dt, *J* = 3.6, 1.0 Hz, 2H, C*H₂*), 2.21 (p, *J* = 3.3 Hz, 1H, C*H*), 1.48 (dt, *J* = 3.2, 1.0 Hz, 2H, C*H₂*).
^{1}H-NMR signals correspond to the ones reported in the literature.[3]

Bicyclo[1.1.0]butan-1-yl(4-methoxyphenyl)methanone (1c)

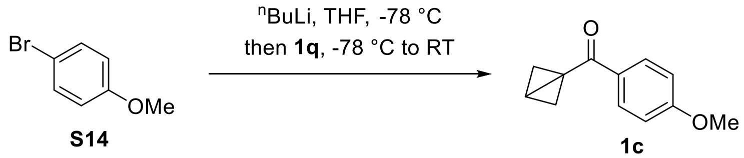

Following a reported procedure,[2] in a 25 mL, two-necked, round-bottomed flask, 4-bromoanisole (**S14**) (0.215 g, 1.15 mmol, 1.15 equiv.) was dissolved in THF (dry; 5.20 mL). The clear, colorless solution was chilled to -78 °C (dry ice - water bath). ^{n}BuLi (2.5 M in hexanes; 0.42 mL, 1.0 mmol, 1.05 equiv.) was then added drop-wise: the resulting mixture remained colorless and clear. It was stirred at -78 °C for 30 minutes. A solution of N-methoxy-N-methylbicyclo[1.1.0]butane-1-carboxamide (**1q**) (1.0 mL of a 1.0 M solution in THF; 1.0 mmol, 1.0 equiv.) was added slowly at the same temperature. The resulting clear solution was stirred at -78 °C for 30 minutes and then at room temperature for another 90 minutes. To the clear very pale yellow reaction mixture was then added sat. aq. $NaHCO_3$ (15 mL). The aqueous layer was separated and extracted with Et_2O (4 x 20 mL). The combined organic layers were washed with brine, dried over Na_2SO_4, filtered, and concentrated under vacuum (heating bath at < 40 °C (!!)). The resulting pale yellow crude oil was submitted to column chromatography (Biotage flash chromatographer, SiO_2; EtOAc in pentane, 4 to 40%) to provide bicyclo[1.1.0]butan-1-yl(4-methoxyphenyl)methanone (**1c**) (0.15 g, 0.80 mmol, 80% yield) as a pale yellow solid. 0.12 g of the latter were immediately dissolved in DCM (2.6 mL) to provide a 0.25 M solution that was stored at -60 °C.

^{1}H NMR (400 MHz, Methylene Chloride-d_2) δ 7.88 (d, J = 9.0 Hz, 2H, ArH), 6.93 (d, J = 9.0 Hz, 2H, ArH), 3.86 (s, 3H, OCH_3), 2.58 (dt, J = 3.5, 0.9 Hz, 2H, CH_2), 2.12 (p, J = 3.3 Hz, 1H, CH), 1.43 (dt, J = 3.1, 0.9 Hz, 2H, CH_2).

^{1}H-NMR signals correspond to the ones reported in the literature.[4]

Bicyclo[1.1.0]butan-1-yl(3-methoxyphenyl)methanone (1d)

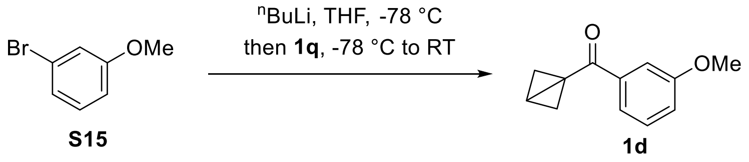

Following a reported procedure,[2] in a 25 mL, two-necked, round-bottomed flask, 3-bromo anisole (**S15**) (0.15 mL, 1.1 mmol, 1.15 equiv.) was dissolved in THF (dry; 5.20 mL). The clear, colorless solution was chilled to -78 °C (dry ice - water bath). ^{n}BuLi (2.5 M in hexanes; 0.42 mL, 1.0 mmol, 1.05 equiv.) was then added drop-wise: the resulting mixture remained colorless, but became progressively slightly turbid. This suspension was stirred at -78 °C for 45 minutes. A solution of N-methoxy-N-methylbicyclo[1.1.0]butane-1-carboxamide (**1q**) (1.0 mL of a 1.0 M solution in THF; 1.0 mmol, 1.0 equiv.) was added slowly at the same temperature. The initially turbid solution became immediately clear. It was stirred at -78 °C for 30 minutes and then at room temperature for another 30 minutes. To the clear very pale yellow reaction mixture was then added sat. aq. $NaHCO_3$ (15 mL). The aqueous layer was separated and extracted with Et_2O (4 x 20 mL). The combined organic layers were washed with brine, dried over Na_2SO_4, filtered, and concentrated under vacuum (heating bath at < 40 °C (!!)). The resulting pale yellow crude oil was submitted to column chromatography (Biotage flash chromatographer, SiO_2; Et_2O in pentane, 4 to 30%) to provide bicyclo[1.1.0]butan-1-yl(3-methoxyphenyl)methanone (**1d**) (0.097 g, 0.52 mmol, 51% yield) as a colorless oil, which was immediately diluted with DCM (2.1 mL) to provide a 0.25 M solution that was stored at -60 °C.

R_f (pentane/EtOAc 9/1) = 0.52.

^{1}H NMR (400 MHz, Methylene Chloride-d_2) δ 7.42 (dt, J = 7.7, 1.3 Hz, 1H, ArH), 7.37–7.30 (m, 2H, ArH), 7.06 (ddd, J = 8.2, 2.7, 1.0 Hz, 1H, ArH), 3.82 (s, 3H, OCH_3), 2.58 (dt, J = 3.6, 1.0 Hz, 2H, CH_2), 2.18 (p, J = 3.4 Hz, 1H, CH), 1.44 (dt, J = 3.2, 1.0 Hz, 2H, CH_2).

^{13}C NMR (101 MHz, Methylene Chloride-d_2) δ 199.6, 160.1, 139.8, 129.6, 121.9, 118.7, 113.7, 55.9, 38.2, 21.6, 17.2.

IR ($\tilde{\nu}_{max}$, cm^{-1}) 2963 (m), 1641 (s), 1581 (s), 1484 (m), 1451 (m), 1429 (m), 1381 (s), 1321 (m), 1288 (m), 1259 (s), 1201 (m), 1159 (m), 1107 (w), 1077 (w), 1046 (m), 1020 (m), 991 (m), 859 (s), 798 (m), 745 (s).

HRMS (nanochip-ESI/LTQ-Orbitrap) m/z: $[M + H]^+$ Calcd for $C_{12}H_{13}O_2^+$ 189.0910; Found 189.0908.

Bicyclo[1.1.0]butan-1-yl(2-methoxyphenyl)methanone (1e)

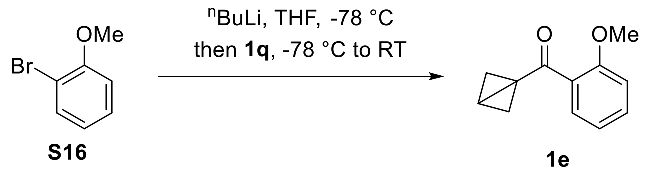

Following a reported procedure,[2] in a 25 mL, two-necked, round-bottomed flask, 2-bromoanisole (**S16**) (0.13 mL, 1.0 mmol, 1.15 equiv.) was dissolved in THF (dry; 4.7 mL). The clear, colorless solution was chilled to -78 °C (dry ice - water bath). ^{n}BuLi (2.5 M in hexanes; 0.38 mL, 0.95 mmol, 1.05 equiv.) was then added drop-wise: the resulting mixture remained colorless and clear. It was stirred at -78 °C for 30 minutes. A solution of N-methoxy-N-methylbicyclo[1.1.0]butane-1-carboxamide (**1q**) (0.90 mL of a 1.0 M solution in THF; 0.90 mmol, 1.0 equiv.) was added slowly (but not drop-wise) at the same temperature. The initially yellow solution faded to pale yellow. It was stirred at -78 °C for 30 minutes and then at room temperature for another 90 minutes, darkening to orange. To the reaction mixture was then added sat. aq. $NaHCO_3$ (15 mL). The aqueous layer was separated and extracted with Et_2O (4 x 20 mL). The combined organic layers were washed with brine, dried over Na_2SO_4, filtered, and concentrated under vacuum (heating bath at < 40 °C (!!)). The resulting pale yellow crude oil was submitted to column chromatography (Biotage flash chromatographer, SiO_2; EtOAc in pentane, 4 to 40%) to provide bicyclo[1.1.0]butan-1-yl(2-methoxyphenyl)methanone (**1e**) (90% pure; 0.129 g, 0.616 mmol, 69% yield) as a colorless oil. 0.11 g of the latter were immediately dissolved in DCM (2.45 mL) to give a 0.25 M solution that was stored at -60 °C.

R_f (pentane/EtOAc 85/15) 0.43.
^{1}H NMR (400 MHz, Methylene Chloride-d_2) δ 7.39 (ddd, *J* = 8.3, 7.5, 1.8 Hz, 1H, Ar*H*), 7.28 (m, 1H, Ar*H*), 7.97 (m, 1H, Ar*H*), 6.95 (m, 1H, Ar*H*), 3.83 (s, 3H, OCH_3), 2.36 (dt, *J* = 3.5, 1.1 Hz, 2H, CH_2), 2.28 (p, *J* = 3.2 Hz, 1H, C*H*), 1.34 (dt, *J* = 3.1, 1.1 Hz, 2H, CH_2).
^{13}C NMR (101 MHz, Methylene Chloride-d_2) δ 201.0, 156.7, 131.4, 129.0, 128.7, 120.3, 111.3, 55.5, 36.9, 22.1, 19.7.
IR ($\tilde{\nu}_{max}$, cm^{-1}) 3051 (w), 2963 (m), 2841 (w), 1598 (s), 1486 (s), 1465 (m), 1437 (m), 1389 (s), 1284 (s), 1249 (s), 1213 (m), 1166 (m), 1112 (m), 1051 (m), 1021 (s), 977 (s), 915 (w), 814 (m), 756 (s).
HRMS (ESI/QTOF) m/z: $[M + H]^+$ Calcd for $C_{12}H_{13}O_2^+$ 189.0910; Found 189.0908.

Bicyclo[1.1.0]butan-1-yl(4-bromophenyl)methanone (1f)

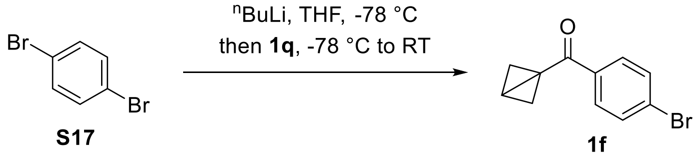

Following a reported procedure,[2] in a 25 mL, two-necked, round-bottomed flask, 1,4-dibromobenzene (**S17**) (0.271 g, 1.15 mmol, 1.15 equiv.) was dissolved in THF (dry; 5.20 mL). The clear, colorless solution was chilled to -78 °C (dry ice - water bath). ^{n}BuLi (2.5 M in hexanes; 0.42 mL, 1.0 mmol, 1.05 equiv.) was then added drop-wise: the resulting mixture remained colorless, but became progressively slightly turbid. This mixture was stirred at -78 °C for 45 minutes. A solution of N-methoxy-N-methylbicyclo[1.1.0]butane-1-carboxamide (**1q**) (1.0 mL of a 1.0 M solution in THF; 1.0 mmol, 1.0 equiv.) was added slowly at the same temperature. The initially turbid solution became immediately clear. It was stirred at -78 °C for 30 minutes and then at room temperature for another 90 minutes. To the clear very pale yellow reaction mixture was then added sat. aq. $NaHCO_3$ (15 mL). The aqueous layer was separated and extracted with Et_2O (4 x 20 mL). The combined organic layers were washed with brine, dried over Na_2SO_4, filtered, and concentrated under vacuum (heating bath at < 40 °C (!!)). The resulting pale yellow crude oil was submitted to column chromatography (Biotage flash chromatographer, SiO_2; EtOAc in pentane, 4 to 40%) to provide bicyclo[1.1.0]butan-1-yl(4-bromophenyl)methanone (**1f**) (0.17 g, 0.72 mmol, 72% yield) as a colorless oil, which was immediately diluted with DCM (2.8 mL) to provide a 0.25 M solution that was stored at -60 °C.

^{1}H NMR (400 MHz, Methylene Chloride-d_2) δ 7.81–7.72 (m, 2H, Ar*H*), 7.63 (d, *J* = 8.6 Hz, 2H, Ar*H*), 2.61 (dt, *J* = 3.6, 1.0 Hz, 2H, CH_2), 2.26 (p, *J* = 3.4 Hz, 1H, C*H*), 1.51 (dt, *J* = 3.2, 1.0 Hz, 2H, CH_2).
^{1}H-NMR signals correspond to the ones reported in the literature.[5]

Bicyclo[1.1.0]butan-1-yl(4-(trifluoromethyl)phenyl)methanone (1g)

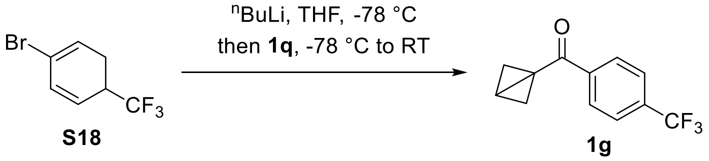

Following a reported procedure,[2] in a 50 mL, two-necked, round-bottomed flask, 1-bromo-4-(trifluoromethyl)benzene (**S18**) (0.17 mL, 1.2 mmol, 1.2 equiv.) was dissolved in THF (dry; 6.1 mL). The clear, colorless solution was chilled to -78 °C (dry ice - water bath). ^{n}BuLi (2.5 M in hexanes; 0.46 mL, 1.1 mmol, 1.15 equiv.) was then added drop-wise: the initially clear, colorless mixture became very turbid and yellow. It was stirred at -78 °C for 45 minutes. A

solution of N-methoxy-N-methylbicyclo[1.1.0]butane-1-carboxamide (**1q**) (1.0 mL of a 1.0 M solution in THF; 1.0 mmol, 1.0 equiv.) was added slowly at the same temperature, becoming clear. It was stirred at -78 °C for 45 minutes and then at room temperature for another 30 minutes. To the now clear, very pale yellow reaction mixture was then added sat. aq. $NaHCO_3$ (15 mL). The aqueous layer was separated and extracted with Et_2O (4 x 20 mL). The combined organic layers were washed with brine, dried over Na_2SO_4, filtered, and concentrated under vacuum (heating bath at < 40 °C (!!)). The resulting pale yellow crude oil was submitted to column chromatography (Biotage flash chromatographer, SiO_2; EtOAc in pentane, 4 to 30%) to provide bicyclo[1.1.0]butan-1-yl(4-(trifluoromethyl)phenyl)methanone (0.17 g, 0.76 mmol, 76% yield) (**1g**) as a colorless oil, which became a solid on standing. 0.16 g of the latter were dissolved in DCM (2.8 mL) in order to obtain a 0.25 M solution that was stored at -60 °C.

^{1}H NMR (400 MHz, Methylene Chloride-d_2) δ 7.94 (d, J = 8.0 Hz, 2H, ArH), 7.71 (d, J = 8.1 Hz, 2H, ArH), 2.58 (dt, J = 3.6, 1.0 Hz, 2H, CH_2), 2.29 (p, J = 3.4 Hz, 1H, CH), 1.51 (dt, J = 3.2, 1.0 Hz, 2H, CH_2).
^{1}H-NMR signals correspond to the ones reported in the literature.[4]

4-(Bicyclo[1.1.0]butane-1-carbonyl)benzonitrile (1h)

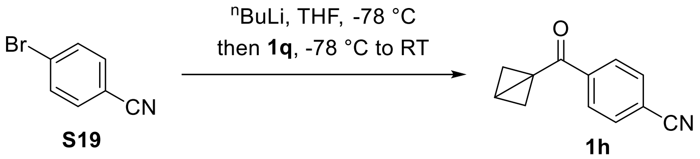

Following a reported procedure,[2] in a 25 mL, two-necked, round-bottomed flask, 4-bromobenzonitrile (**S19**) (0.209 g, 1.15 mmol, 1.15 equiv.) was dissolved in THF (dry; 5.2 mL). The clear, colorless solution was chilled to -78 °C (dry ice - water bath). ^{n}BuLi (2.5 M in hexanes; 0.42 mL, 1.0 mmol, 1.05 equiv.) was then added drop-wise: the resulting mixture remained clear but turned to yellow. This mixture was stirred at -78 °C for 45 minutes. A solution of N-methoxy-N-methylbicyclo[1.1.0]butane-1-carboxamide (**1q**) (1.0 mL of a 1.0 M solution in THF; 1.0 mmol, 1.0 equiv.) was added slowly at the same temperature. The initially yellow solution gradually darkened to orange. It was stirred at -78 °C for 30 minutes and then at room temperature for another 90 minutes. To the now clear, very pale yellow reaction mixture was then added sat. aq. $NaHCO_3$ (15 mL). The aqueous layer was separated and extracted with Et_2O (4 x 20 mL). The combined organic layers were washed with brine, dried over Na_2SO_4, filtered, and concentrated under vacuum (heating bath at < 40 °C (!!)). The resulting pale yellow crude oil was submitted to column chromatography (Biotage flash chromatographer, SiO_2; EtOAc in pentane, 4 to 40%) to provide 4-(bicyclo[1.1.0]butane-1-carbonyl)benzonitrile (**1h**) (95% pure; 0.121, 0.660 mmol, 66% yield) as pale yellow solid,

which was immediately diluted in DCM (2.6 mL) in order to obtain a 0.25 M solution that was stored at -60 °C.

R$_f$ (pentane/EtOAc 85/15) 0.45.

^{1}H NMR (400 MHz, Methylene Chloride-*d*$_2$) δ 7.93 – 7.86 (m, 2H, Ar*H*), 7.76 – 7.68 (m, 2H, Ar*H*), 2.56 (dt, *J* = 3.6, 1.1 Hz, 2H, C*H$_2$*), 2.31 (p, *J* = 3.4 Hz, 1H, C*H*), 1.51 (dt, *J* = 3.3, 1.1 Hz, 2H, C*H$_2$*).

^{13}C NMR (101 MHz, Methylene Chloride-*d*$_2$) δ 198.1, 141.2, 132.1, 129.0, 118.1, 115.3, 37.8, 22.7, 17.1.

IR ($\tilde{\nu}_{max}$, cm^{-1}) 3090 (w), 3050 (w), 2960 (w), 2231 (m), 1483 (w), 1402 (s), 1289 (w), 1219 (s), 1170 (w), 1112 (m), 1075 (w), 1015 (m), 981 (s), 928 (w), 848 (s), 816 (m), 756 (m).

HRMS (APCI/QTOF) m/z: [M + H]$^+$ Calcd for $C_{12}H_{10}NO^+$ 184.0757; Found 184.0754.

Bicyclo[1.1.0]butan-1-yl(5-bromothiophen-2-yl)methanone (1i)

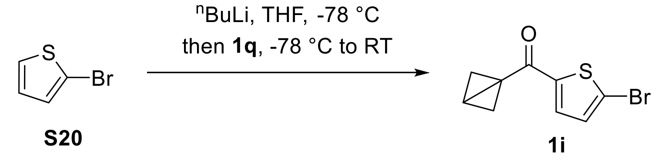

Following a reported procedure,[2] in a 50 mL, two-necked, round-bottomed flask, 2-bromothiophene (**S20**) (0.11 mL, 1.1 mmol, 1.15 equiv.) was dissolved in THF (dry; 5.2 mL). The clear, colorless solution was chilled to -78 °C (dry ice - water bath). ^{n}BuLi (2.5 M in hexanes; 0.42 mL, 1.0 mmol, 1.05 equiv.) was then added drop-wise: the resulting mixture remained clear but turned to pale yellow. This mixture was stirred at -78 °C for 45 minutes. A solution of N-methoxy-N-methylbicyclo[1.1.0]butane-1-carboxamide (**1q**) (1.0 mL of a 1.0 M solution in THF; 1.0 mmol, 1.0 equiv.) was added slowly at the same temperature. The mixture was stirred at -78 °C for 30 minutes, turning to darker yellow. The cooling bath was then removed: the solution became green and then orange-brown. It was stirred at room temperature for another 20 minutes, remaining orange-brown. To the reaction mixture was then added sat. aq. $NaHCO_3$ (15 mL). The aqueous layer was separated and extracted with Et_2O (4 x 20 mL). The combined organic layers were washed with brine, dried over Na_2SO_4, filtered, and concentrated under vacuum (heating bath at < 40 °C (!!)). The resulting orange crude oil was submitted to column chromatography (Biotage flash chromatographer, SiO_2; EtOAc in pentane, 0 to 40%) to provide bicyclo[1.1.0]butan-1-yl(5-bromothiophen-2-yl)methanone (**1i**) (0.095 g, 0.39 mmol, 39% yield) as a pale yellow oil, which was immediately diluted in DCM (dry; 2.3 mL) to give a 0.17 M solution that was stored at -60 °C.

R$_f$ (pentane/EtOAc 9/1) 0.50.

^{1}**H NMR** (400 MHz, Methylene Chloride-d_2) δ 7.48 (d, *J* = 4.0 Hz, 1H, Het*H*), 7.12 (d, *J* = 4.0 Hz, 1H, Het*H*), 2.69 (dt, *J* = 3.6, 1.0 Hz, 2H, CH_2), 2.27 (p, *J* = 3.4 Hz, 1H, C*H*), 1.43 (dt, *J* = 3.2, 1.0 Hz, 2H, CH_2).

^{13}**C NMR** (101 MHz, Methylene Chloride-d_2) δ 189.2, 145.3, 132.7, 131.1, 121.1, 37.1, 21.0, 16.0.

IR ($\tilde{\nu}_{max}$, cm^{-1}) 3100 (w), 3048 (w), 2960 (w), 2935 (w), 1614 (s), 1523 (w), 1483 (w), 1413 (s), 1388 (m), 1317 (w), 1216 (m), 1170 (w), 1108 (w), 1076 (w), 980 (m), 917 (m), 806 (m), 733 (m).

HRMS (ESI/QTOF) m/z: [M + H]$^+$ Calcd for $C_9H_8BrOS^+$ 242.9474 and 244.9452; Found 242.9470 and 244.9453.

(3-Methylbicyclo[1.1.0]butan-1-yl)(naphthalen-2-yl)methanone (1j)

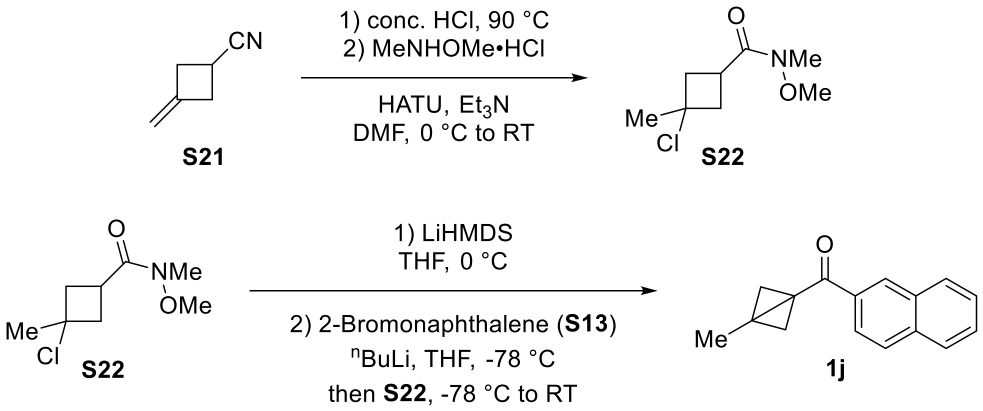

Following a reported procedure,[2] in a 100 mL glass sealable flask, 3-methylenecyclobutanecarbonitrile (**S21**) (1.6 mL, 16 mmol, 1.0 equiv.) was mixed with conc. aq. HCl (37% v/v; 13.5 mL, 160 mmol, 10 equiv.). The mixture stirred at 95-100 °C for 9 hours. The yellow solution was then allowed to cool down to room temperature, at which it became a suspension because of the precipitation of an off-white solid. It was then diluted with water (40 mL), and extracted with DCM (3 x 40 mL). The combined organic layers were then washed with brine, dried over Na_2SO_4, filtered and concentrated under reduced pressure to provide 3-chloro-3-methylcyclobutane-1-carboxylic acid (1.95 g, 2.14 mmol, 82% yield) as a viscous, colorless oil, which became an off-white solid upon standing at 4 °C (fridge) overnight.

Following a reported procedure,[6] in a 50 mL, single-necked, round-bottomed flask, part of the crude solid obtained in the previous step (3-chloro-3-methylcyclobutane-1-carboxylic acid; 0.90 g, 6.1 mmol, 1.0 equiv.) was dissolved in DMF (12 mL) together with HATU (2.53 g, 6.66 mmol, 1.1 equiv.) and N,O-dimethylhydroxylamine hydrochloride (1.18 g, 12.1 mmol, 2.0 equiv.). The resulting solution was cooled to 0 °C (ice - water bath) prior to the addition of NEt_3 (2.5 mL, 18 mmol, 3.0 equiv.), which made turn the mixture into a bright yellow suspension.

The latter was stirred at room temperature for 26 hours. The mixture was then treated with water (30 mL). The aqueous solution was extracted to EtOAc (3 x 30 mL). The combined organic layers were washed with aq. HCl (1.0 M; 30 mL), sat. aq. $NaHCO_3$ (2 x 30 mL), and brine. They were then dried over Na_2SO_4, filtered, and concentrated under reduced pressure to provide 3-chloro-N-methoxy-N,3-dimethylcyclobutane-1-carboxamide (**S22**) (1.14 g, 5.95 mmol, 98% yield) as a colorless oil, which was used directly in the next step without further purification.

Following a reported procedure,[2] in a 25 mL, round-bottomed, two-necked flask, 3-chloro-N-methoxy-N,3-dimethylcyclobutane-1-carboxamide (**S22**) (0.58 g, 3.0 mmol, 1.0 equiv.) was dissolved in THF (dry; 11.6 mL). The clear, colorless was cooled to 0 °C (ice - water bath), prior to slow the addition of LiHMDS (1.0 M in THF; 3.5 mL, 2.4 mmol, 1.15 equiv.). Immediately, the mixture became yellow. It was stirred at 0 °C for 1 hour, becoming orange. The reaction was quenched by addition of water and sat. aq. $NaHCO_3$ (20 mL). The aqueous layer was separated and extracted with Et_2O (3 x 20 mL). The combined organic layers were washed twice with brine, dried over Na_2SO_4, filtered, and concentrated under vacuum. The resulting orange, crude oil was immediately dissolved in THF (3.0 mL) to give a clear solution (solution A). Meanwhile, in a 50 mL, round-bottomed, two-necked flask, 2-bromonaphthalene (0.63 g, 3.0 mmol, 1.0 equiv.) was dissolved in THF (dry; 17.2 mL). The solution was chilled to -78 °C (dry ice - acetone bath). ^{n}BuLi (2.5 M in hexanes; 1.2 mL, 3.0 mmol, 1.0 equiv.) was added drop-wise. The resulting turbid, yellow mixture was stirred at -78 °C for 60 minutes. At this point, the solution A was added. The mixture immediately became clear, and it was stirred at the same temperature for 60 minutes. The reaction was then quenched by adding sat. aq. $NaHCO_3$ (20 mL). The aqueous layer was separated and extracted with Et_2O (3 x 20 mL). The combined organic layers were washed twice with brine, dried over Na_2SO_4, filtered, and concentrated under vacuum. The resulting orange crude oil was submitted to column chromatography (Biotage flash chromatographer, SiO_2; Et_2O in pentane, 5 to 40%) to provide (3-methylbicyclo[1.1.0]butan-1-yl)(naphthalen-2-yl)methanone (**1j**) (0.16 g, 0.70 mmol, 23% yield) as a pale yellow oil, which was immediately diluted in DCM (2.7 mL) to give a 0.25 M solution that was stored at -60 °C.

^{1}H NMR (400 MHz, Chloroform-*d*) δ 8.37 (m, 1H, Ar*H*), 7.94 (dd, *J* = 8.0, 6.9 Hz, 1H, Ar*H*), 7.92 – 7.83 (m, 3H, Ar*H*), 7.56 (dddd, *J* = 14.7, 8.3, 6.9, 1.5 Hz, 2H, Ar*H*), 2.53 (s, 2H, CH_2), 1.63 (t, *J* = 1.0 Hz, 2H, CH_2), 1.48 (s, 3H, CH_3).
^{1}H-NMR signals correspond to the ones reported in the literature.[2]

Naphthalen-2-yl(3-phenylbicyclo[1.1.0]butan-1-yl)methanone (1k)

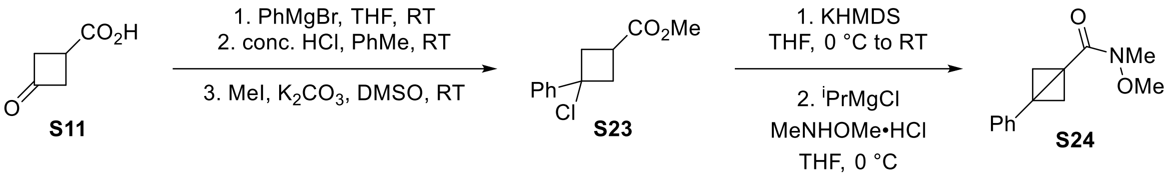

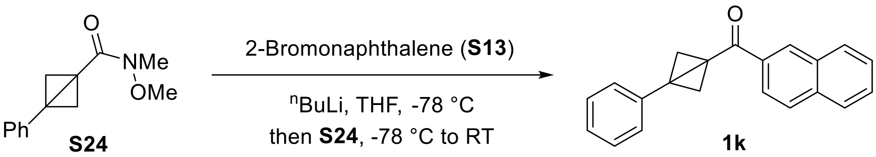

Following a reported procedure,[7] in a 100 mL two-necked, round-bottomed flask, 3-oxocyclobutanecarboxylic acid (**S11**) (0.570 g, 5.00 mmol, 1.0 equiv.) was dissolved in THF (dry; 5.4 mL). PhMgBr (1.0 M in THF; 10.2 mL, 10.2 mmol, 2.04 equiv.) was then added drop-wise at room temperature, over 2.5 hours, using a syringe pump. Once the addition was completed, the now yellow mixture was stirred for another 40 minutes at room temperature. Sat. aq. NH_4Cl (5.0 mL) and water (5.0 mL) were then added sequentially, under stirring, which made the mixture become very turbid. The latter was partially concentrated under reduced pressure, and then conc. HCl (25% v/v; 1.5 mL) and aq. $NaHSO_4$ (2.0 M; 10 mL) were added. The resulting aqueous layer was extracted with EtOAc (3 x 40 mL). The combined organic layers were washed with brine, dried over Na_2SO_4, filtered, and concentrated under vacuum to provide an off-white paste. Trituration with chloroform (4.0 mL) at 70 °C (precipitation was further promoted by addition of pentane upon allowing the mixture to cool back to room temperature) provided 3-hydroxy-3-phenylcyclobutane-1-carboxylic acid (0.62 g, 3.2 mmol, 64% yield) as an off-white solid.

In a 25 mL, single necked, round bottomed flask, the recrystallized 3-hydroxy-3-phenylcyclobutane-1-carboxylic acid (0.62 g, 3.2 mmol, 1.0 equiv.) was suspended in toluene (5.0 mL). To the resulting off-white suspension, conc. aq. HCl (37% v/v; 5.0 mL) was added drop-wise. The suspension became yellow, and it was stirred at room temperature for 24 hours. It was then diluted with water (20 mL). The organic layer was separated, and the aqueous one was extracted once with MTBE (25 mL). The combined organic layers were washed with water (20 mL), brine, dried over Na_2SO_4, filtered, and concentrated under vacuum to provide 3-chloro-3-phenylcyclobutane-1-carboxylic acid (ca. 70% pure; 0.411 g, 1.96 mmol, 61% yield; 39% overall yield) as an off-white solid.

Following a reported procedure,[2] in a 50 mL, single-necked, round-bottomed flask, 3-chloro-3-phenylcyclobutane-1-carboxylic acid (0.411 g, 1.95 mmol, 1.0 equiv.) and K_2CO_3 (0.55 g, 4.0 mmol, 2.0 equiv.) were dissolved in DMF (9.8 mL; K_2CO_3 remained partially undissolved).

Iodomethane (0.19 mL, 3.0 mmol, 1.5 equiv.) was then added. The resulting clearer mixture was stirred overnight, turning into a homogeneous off-white suspension. After 19 hours, the mixture was dissolved by addition of brine (20 mL). The aqueous layer was extracted with Et_2O (4 x 20 mL). The combined organic layers were washed with brine (2 x 25 mL), dried over Na_2SO_4, filtered, and concentrated under vacuum. The resulting pale yellow, crude oil was submitted to column chromatography (Biotage flash chromatographer, SiO_2; EtOAc in pentane, 0 to 30%) to provide methyl methyl 3-chloro-3-phenylcyclobutane-1-carboxylate (**S23**) (mixture of diastereoisomers; 0.23 g, 1.0 mmol, 52% yield) as a colorless oil.

In a 10 mL, two-necked, round-bottomed flask, methyl 3-chloro-3-(4-phenyl)cyclobutane-1-carboxylate (**S23**) (mixture of diastereoisomers; 0.23 g, 1.0 mmol, 1.0 equiv.) was dissolved in THF (dry; 5.0 mL). The solution was cooled to 0 °C (ice - water bath). KHMDS (1.0 M in THF; 1.2 mL, 1.2 mmol, 1.2 equiv.) was then added drop-wise: the mixture turned locally to yellow-orange upon the addition of each drop, and then reverted to colorless; it remained yellow after ca. 50% of the base had been added. The resulting yellow solution was stirred at 0 °C for 30 minutes, and at room temperature for 1.5 hours, becoming an orange suspension. The reaction was quenched by addition of sat. aq. $NaHCO_3$ (10 mL). The aqueous layer was separated and extracted with Et_2O (3 x 30 mL). The combined organic layers were washed with brine, dried over Na_2SO_4, filtered, and concentrated under vacuum. The resulting pale orange, crude oil was submitted to column chromatography (Biotage flash chromatographer, SiO_2; EtOAc in pentane, 0 to 40%) to provide methyl 3-phenylbicyclo[1.1.0]butane-1-carboxylate (0.17 g, 0.86 mmol, 85% yield) as an off-white solid.

^{1}H NMR (400 MHz, Chloroform-*d*) δ 7.30 (d, *J* = 4.1 Hz, 4H, Ph*H*), 7.25 (m, 1H, Ph*H*), 3.49 (s, 3H, OCH_3), 2.93 (d, *J* = 1.0 Hz, 2H, CH_2), 1.61 (s, 2H, CH_2).
^{1}H-NMR signals correspond to the ones reported in the literature.[4]

In a 25 mL, two-necked, round-bottomed flask, methyl 3-(4-phenyl)bicyclo[1.1.0]butane-1-carboxylate (0.17 g, 0.86 mmol, 1.0 equiv.) was dissolved in THF (dry; 4.3 mL). The resulting colorless solution was cooled to 0 °C (ice - water bath). Methoxy(methyl)amine hydrochloride (0.10 g, 1.0 mmol, 1.2 equiv.) was then added. The resulting mixture was stirred at 0 °C for 5 minutes. *iso*Propyl magnesium chloride (2.0 M in THF; 1.0 mL, 2.1 mmol, 2.4 equiv.) was added slowly. The pale yellow mixture was stirred at 0 °C for 1 hour and at room temperature for another 1.5 hours. It was then diluted with Et_2O, and the reaction was then quenched by addition of sat. aq. NH_4Cl (10 mL). The aqueous layer was separated and extracted with Et_2O (3 x 15 mL). The combined organic layers were washed with brine, dried over Na_2SO_4, filtered, and concentrated under vacuum. The resulting pale yellow crude oil was submitted to column

chromatography (Biotage flash chromatographer, SiO_2; EtOAc in pentane, 5 to 60%) to provide N-methoxy-N-methyl-3-phenylbicyclo[1.1.0]butane-1-carboxamide (**S24**) (0.14 g, 0.63 mmol, 73% yield) as a colorless solid.

^{1}H NMR (400 MHz, Chloroform-*d*) δ 7.39–7.26 (m, 4H, Ph*H*), 7.22 (m, 1H, Ph*H*), 3.64 (s, 3H, CH_3), 3.11 (s, 3H, CH_3), 2.99 (s, 2H, CH_2), 1.59 (s, 2H, CH_2).

In a 50 mL, two-necked, round-bottomed flask, 2-bromonaphthalene (**S13**) (0.17 g, 0.81 mmol, 1.3 equiv.) was dissolved in a THF (dry; 5.0 mL). The clear, colorless solution was chilled to -78 °C (dry ice - water bath). ^{n}BuLi (2.5 M in hexanes; 0.30 mL, 0.75 mmol, 1.15 equiv.) was then added drop-wise: the resulting mixture became yellow at first, and then very turbid remaining yellow. The suspension was stirred at -78 °C for 45 minutes. A solution of N-methoxy-N-methyl-3-phenylbicyclo[1.1.0]butane-1-carboxamide (**S24**) (0.14 g, 0.63 mmol, 1.0 equiv.) in THF (1.0 mL) was added slowly at the same temperature. The yellow, turbid solution remained as such until it was stirred at -78 °C. After 60 minutes, the cooling bath was removed, and the mixture was stirred at room temperature for another 30 minutes, becoming clear and turning to dark green. Sat. aq. $NaHCO_3$ (10 mL) was then added. The aqueous layer was separated and extracted with Et_2O (4 x 40 mL). The combined organic layers were washed with brine, dried over Na_2SO_4, filtered, and concentrated under vacuum (heating bath at < 40 °C (!!)). The resulting pale yellow crude oil was submitted to column chromatography (Biotage flash chromatographer, SiO_2; EtOAc in pentane, 0 to 40%) to provide naphthalen-2-yl(3-phenylbicyclo[1.1.0]butan-1-yl)methanone (**1k**) (0.14 g, 0.50 mmol, 80% yield) as a colorless, crystalline solid, which was stored at -60 °C.

^{1}H NMR (400 MHz, Chloroform-*d*) δ 8.14 (s, 1H, Ar*H*), 7.89 (d, *J* = 7.8 Hz, 1H, Ar*H*), 7.84 (d, *J* = 7.8 Hz, 1H, Ar*H*), 7.77 (d, *J* = 8.5 Hz, 1H, Ar*H*), 7.59 – 7.50 (m, 3H, Ar*H*), 7.21 (dd, *J* = 5.2, 1.8 Hz, 3H, Ph*H*), 7.14 (dd, *J* = 7.2, 2.5 Hz, 2H, Ph*H*), 3.24 (s, 2H, CH_2), 1.98 (s, 2H, CH_2).
^{1}H-NMR signals correspond to the ones reported in the literature.[4]

(3-(3,4-Difluorophenyl)bicyclo[1.1.0]butan-1-yl)(naphthalen-2-yl)methanone (1l)

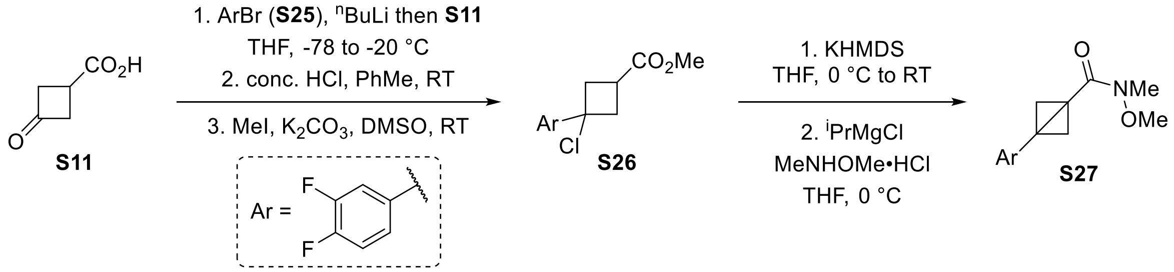

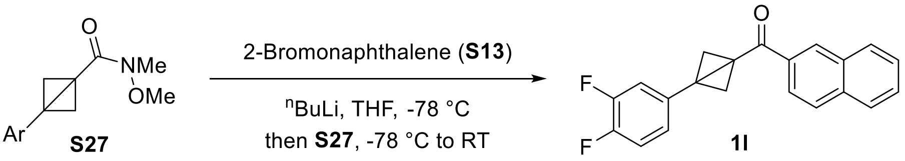

Following a reported procedure,[7] in a 100 mL two-necked, round-bottomed flask,1,2-difluoro-4-iodobenzene (**S25**) (2.1 mL, 18 mmol, 2.2 equiv.) was dissolved in THF (dry; 25 mL). The resulting colorless solution was chilled to -78 °C (dry ice - acetone bath). ^{n}BuLi (2.5 M in hexanes; 7.0 mL, 18 mmol, 2.2 equiv.) was added slowly. During the addition, the solution became very turbid and yellow. The suspension was stirred at -78 °C for 1 hour. A solution of 3-oxocyclobutane-1-carboxylic acid (**S11**) (0.91 g, 8.0 mmol, 1.0 equiv) in THF (dry; 3.1 mL) was added in one portion: the mixture immediately converted into a clear, pale yellow solution. Temperature was increased to -25 ~ -20°C (salt - ice bath), and stirring was continued for 2 hour and 30 minutes. The reaction was then quenched by addition of sat. aq. NH_4Cl (10 mL) and water (4.8 mL). To the mixture was then added pentane (ca. 40 mL), the organic layer was separated and it was extracted with water (3 x 15 mL). The combined organic layers were acidified until pH ~1 by addition of aq. $NaHSO_4$ (2.0 M). The aqueous solution was then extracted with MTBE (3 x 25 mL). The combined organic layers were washed twice with brine, dried over Na_2SO_4, filtered, and concentrated under reduced pressure to provide 3-hydroxy-3-(4-(trifluoromethyl)phenyl)cyclobutane-1-carboxylic acid (1.5 g, 6.6 mmol, 82% yield) as an off-white, pasty solid, which was not submitted to further purification.

In a 100 mL, single-necked, round-bottomed flask, part of the crude solid obtained in the previous step (1.5 g, 6.6 mmol, 1.0 equiv.) was dispersed in toluene (6.3 mL). To the resulting whitish suspension, conc. HCl (37% v/v; 6.3 mL, 76 mmol, 11 equiv.) was added dropwise under vigorous stirring at room temperature. The mixture gradually turned to pale yellow. It was stirred at room temperature for 8 hours. Water (30 mL) and EtOAc (30 mL) were then added. The layers were separated, and the aqueous one was extracted once with EtOAc (30 mL). The combined organic layers were washed with water (30 mL), twice with brine, dried over Na_2SO_4, and concentrated under vacuum to provide crude 3-chloro-3-(3,4-

difluorophenyl)cyclobutane-1-carboxylic acid (1.5 g, 6.1 mmol, 76% yield) as an off-white very viscous oil, which was as such in the following step without further purification.

Following a reported procedure,[2] in a 50 mL, single-necked, round-bottomed flask, 3-chloro-3-(3,4-difluorophenyl)cyclobutane-1-carboxylic acid (1.49 g, 6.06 mmol, 1.0 equiv.) and K_2CO_3 (1.71 g, 12.4 mmol, 2.0 equiv.) were dissolved in DMF (30 mL; K_2CO_3 remained partially undissolved). Iodomethane (0.58 mL, 9.3 mmol, 1.5 equiv.) was then added. The resulting clearer mixture was stirred overnight, turning into a homogeneous off-white suspension. After 19 hours, the mixture was dissolved by addition of brine (40 mL). The aqueous layer was extracted with Et_2O (4 x 40 mL). The combined organic layers were washed with brine (40 mL), dried over Na_2SO_4, filtered, and concentrated under vacuum. The resulting pale yellow, crude oil was submitted to column chromatography (Biotage, 40 g SiO_2; EtOAc in pentane, 0 to 30%) to provide methyl 3-chloro-3-(3,4-difluorophenyl)cyclobutane-1-carboxylate (**S26**) (mixture of diastereoisomers; 1.17 g, 4.47 mmol, 74% yield) as a colorless oil.

In a 100 mL, two-necked, round-bottomed flask, methyl 3-chloro-3-(3,4-difluorophenyl)cyclobutane-1-carboxylate (**S26**) (mixture of diastereoisomers; 1.16 g, 4.45 mmol, 1.0 equiv.) was dissolved in THF (dry; 26mL). The solution was cooled to 0 °C (ice - water bath). KHMDS (1.0 M in THF; 5.3 mL, 5.3 mmol, 1.2 equiv.) was then added drop-wise: the mixture turned locally to yellow-orange upon the addition of each drop, and then reverted to colorless; it remained yellow after ca. 50% of the base had been added. The resulting yellow solution was stirred at 0 °C for 30 minutes, and the mixture was stirred at room temperature for 1.5 hours, becoming an orange suspension. The reaction was quenched by addition of sat. aq. $NaHCO_3$ (40 mL). The aqueous layer was separated and extracted with Et_2O (3 x 50 mL). The combined organic layers were washed with brine, dried over Na_2SO_4, filtered, and concentrated under vacuum. The resulting pale orange, crude oil was submitted to column chromatography (Biotage, 25 g SiO_2; EtOAc in pentane, 0 to 30%) to provide methyl 3-(3,4-difluorophenyl)bicyclo[1.1.0]butane-1-carboxylate (0.66 g, 2.9 mmol, 66% yield) as a colorless oil, which converted into a solid upon standing at 4 °C (inside a fridge).

^{1}H NMR (400 MHz, Chloroform-*d*) δ = 7.13-7.04 (m, 2H, Ar*H*), 7.00 (m, 1H, Ar*H*), 3.53 (s, 3H, OCH_3), 2.85 (s, 2H, CH_2), 1.62 (s, 2H, CH_2).

In a 25 mL, two-necked, round-bottomed flask, methyl 3-(3,4-difluorophenyl)bicyclo[1.1.0]butane-1-carboxylate (0.66 g, 2.9 mmol, 1.0 equiv.) was dissolved in THF (dry; 14.6 mL). The resulting colorless solution was cooled to 0 °C (ice - water bath). Methoxy(methyl)amine hydrochloride (0.34 g, 3.5 mmol, 1.2 equiv.) was then added. The

resulting mixture was stirred at 0 °C for 5 minutes. ^{i}Propyl magnesium chloride (2.0 M in THF; 3.5 mL, 7.0 mmol, 2.4 equiv.) was added slowly. The pale yellow mixture was stirred at 0 °C for 1 hour and at room temperature for another hour. It was then diluted with Et_2O, and the reaction was then quenched by addition of sat. aq. NH_4Cl (40 mL). The aqueous layer was separated and extracted with Et_2O (3 x 30 mL). The combined organic layers were washed with brine, dried over Na_2SO_4, filtered, and concentrated under vacuum. The resulting pale yellow crude oil was submitted to column chromatography (Biotage, 24 g SiO_2; EtOAc in pentane, 5 to 60%) to provide 3-(3,4-difluorophenyl)-N-methoxy-N-methylbicyclo[1.1.0]butane-1-carboxamide (**S27**) (0.48 g, 1.9 mmol, 65% yield) as a colorless solid.

^{1}H NMR (400 MHz, Chloroform-*d*) δ 7.11 (m, 1H, Ar*H*), 7.11 (m, 1H, Ar*H*), 7.03 (ddd, *J* = 7.2, 3.0, 1.6 Hz, 1H, Ar*H*), 3.68 (s, 3H, C*H*$_3$), 3.12 (s, 3H, C*H*$_3$), 2.91 (s, 2H, C*H*$_2$), 1.61 (s, 2H, C*H*$_2$).

In a 50 mL, two-necked, round-bottomed flask, 2-bromonaphthalene (**S13**) (0.51 g, 2.5 mmol, 1.3 equiv.) was dissolved in a THF (dry; 15 mL). The clear, colorless solution was chilled to -78 °C (dry ice - water bath). ^{n}BuLi (2.5 M in hexanes; 0.91 mL, 2.3 mmol, 1.15 equiv.) was then added drop-wise: the resulting mixture became yellow at first, and then very turbid remaining yellow. The suspension was stirred at -78 °C for 60 minutes. A solution of 3-(3,4-difluorophenyl)-N-methoxy-N-methylbicyclo[1.1.0]butane-1-carboxamide (**S27**) (0.48 g, 1.9 mmol, 1.0 equiv.) in THF (2.0 mL) was added slowly at the same temperature. The yellow, turbid solution remained as such until it was stirred at -78 °C. After 30 minutes, the cooling bath was removed, and the mixture was stirred at room temperature for another 30 minutes, becoming clear. Sat. aq. $NaHCO_3$ (30 mL) was then added. The aqueous layer was separated and extracted with Et_2O (3 x 50 mL). The combined organic layers were washed with brine, dried over Na_2SO_4, filtered, and concentrated under vacuum (heating bath at < 40 °C (!!)). The resulting pale yellow crude oil was submitted to column chromatography (Biotage, 25 g SiO_2; EtOAc in pentane, 0 to 30%) to provide (3-(3,4-difluorophenyl)bicyclo[1.1.0]butan-1-yl)(naphthalen-2-yl)methanone (**1l**) (0.47 g, 1.4 mmol, 76% yield) as a colorless, crystallatine solid, which was stored at -60 °C.

M.P. 114.3-118.3 °C
R_f (pentane/EtOAc 9/1) 0.47
^{1}H NMR (400 MHz, Chloroform-*d*) δ 8.20 (s, 1H, Ar*H*), 7.92 (d, *J* = 7.9 Hz, 1H, Ar*H*), 7.85 (d, *J* = 7.9 Hz, 1H, Ar*H*), 7.81 (d, *J* = 8.6 Hz, 1H, Ar*H*), 7.62 (dd, *J* = 8.5, 1.7 Hz, 1H, Ar*H*), 7.61 – 7.52 (m, 2H, Ar*H*), 7.00 (dt, *J* = 10.0, 8.3 Hz, 1H, Ar*H*), 6.95 (m, 1H, Ar*H*), 6.86 (m, 1H, Ar*H*), 3.16 (t, *J* = 1.2 Hz, 2H, C*H*$_2$), 1.99 (t, *J* = 1.2 Hz, 2H, C*H*$_2$).
^{19}F NMR (376 MHz, Chloroform-*d*) δ -137.16 (d, *J* = 21.3 Hz), -139.15 (d, *J* = 21.3 Hz).

^{13}C NMR (101 MHz, Chloroform-*d*) δ 195.8, 151.3 (dd, *J* = 50.9, 13.4 Hz), 148.8 (dd, *J* = 50.9, 12.8 Hz), 135.4, 135.3, 132.3, 130.5 (dd, *J* = 6.5, 3.8 Hz), 130.2, 129.5, 128.3, 127.9, 126.9, 124.4, 122.3 (dd, *J* = 6.3, 3.5 Hz), 117.5 (d, *J* = 18.3 Hz), 115.3 (d, *J* = 18.5 Hz), 38.2, 36.9, 31.3.

IR ($\tilde{\nu}_{max}$, cm^{-1}) 3065 (w), 2957 (w), 2896 (w), 2843 (w), 1718 (s), 1602 (w), 1451 (w), 1387 (w), 1314 (m), 1228 (w), 1176 (w), 1113 (m), 1069 (w), 1027 (w), 712 (s)

HRMS (ESI/QTOF) m/z: $[M + H]^+$ Calcd for $C_{21}H_{15}F_2O^+$ 321.1085; Found 321.1084.

Naphthalen-2-yl(3-(4-(trifluoromethyl)phenyl)bicyclo[1.1.0]butan-1-yl)methanone (1m)

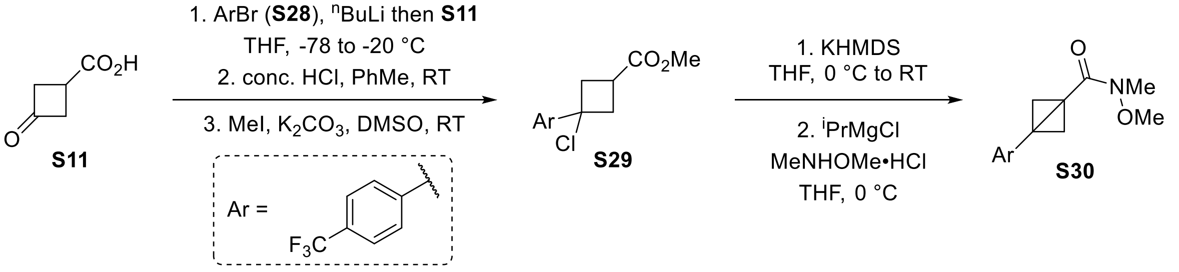

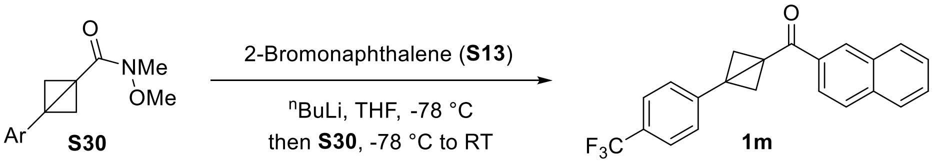

Following a reported procedure,[7] in a 100 mL two-necked, round-bottomed flask, 4-bromobenzotrifluoride (**S28**) (2.1 mL, 15 mmol, 2.2 equiv.) was dissolved in THF (dry; 22 mL). The resulting colorless solution was chilled to -78 °C (dry ice - acetone bath). ^{n}BuLi (2.5 M in hexanes; 6.1 mL, 15 mmol, 2.2 equiv.) was added slowly. During the addition, the solution became very turbid and yellow. The suspension was stirred at -78 °C for 1 hour. A solution of 3-oxocyclobutane-1-carboxylic acid (**S11**) (0.80 g, 7.0 mmol, 1.0 equiv) in THF (dry; 2.7 mL) was added in one portion: the mixture immediately converted into a clear, pale yellow solution. Temperature was increased to -25 ~ -20°C, and stirring was continued for 1 hour and 20 minutes. The reaction was then quenched by addition of sat. aq. NH_4Cl (8.8 mL) and water (5.5 mL). To the mixture was then added pentane (ca. 40 mL), the organic layer was separated and it was extracted with water (3 x 15 mL). The combined organic layers were acidified until pH ~1 by addition of aq. $NaHSO_4$ (2.0 M). The aqueous solution was then extracted with MTBE (3 x 20 mL). The combined organic layers were washed twice with brine, dried over Na_2SO_4, filtered, and concentrated under reduced pressure to provide 3-hydroxy-3-(4-(trifluoromethyl)phenyl)cyclobutane-1-carboxylic acid (1.6 g, 6.0 mmol, 85% yield) as a colorless solid, which was not submitted to further purification.

In a 100 mL, single-necked, round-bottomed flask, part of the crude solid obtained in the previous step (1.4 g, 5.3 mmol, 1.0 equiv.) was dispersed in toluene (6.0 mL). To the resulting whitish suspension, conc. HCl (37% v/v; 6.4 mL, 78 mmol, 15 equiv.) was added dropwise under vigorous stirring at room temperature. The mixture gradually turned to pale yellow. It was stirred at room temperature for 18 hours. Water (30 mL) and EtOAc (30 mL) were then added. The layers were separated, and the aqueous one was extracted once with EtOAc (30 mL). The combined organic layers were washed with water (30 mL), twice with brine, dried over Na_2SO_4, and concentrated under vacuum to provide crude 3-chloro-3-(4-(trifluoromethyl)phenyl)cyclobutane-1-carboxylic acid (1.4 g, 5.2 mmol, 98% yield) as an off-white solid, which was as such in the following step without further purification.

Following a reported procedure ,[2] in a 50 mL, single-necked, round-bottomed flask, crude 3-chloro-3-(4-(trifluoromethyl)phenyl)cyclobutane-1-carboxylic acid (0.43 g, 1.5 mmol, 1.0 equiv.) and K_2CO_3 (0.43 g, 3.1 mmol, 2.0 equiv.) were dissolved in DMF (7.7 mL; K_2CO_3 remained partially undissolved). Iodomethane (0.15 mL, 2.4 mmol, 1.6 equiv.) was then added. The resulting clearer mixture was stirred overnight, turning into a homogeneous off-white suspension. After 20 hours, the mixture was dissolved by addition of brine (20 mL). The aqueous layer was extracted with Et_2O (4 x 20 mL). The combined organic layers were washed with brine (2 x 25 mL), dried over Na_2SO_4, filtered, and concentrated under vacuum. The resulting pale yellow, crude oil was submitted to column chromatography (Biotage flash chromatographer, SiO_2; EtOAc in pentane, 0 to 30%) to provide methyl 3-chloro-3-(4-(trifluoromethyl)phenyl)cyclobutane-1-carboxylate (**S29**) (0.38 g, 1.3 mmol, 84% yield) as a colorless oil.

In a 50 mL, two-necked, round-bottomed flask, 3-chloro-3-(4-(trifluoromethyl)phenyl)cyclobutane-1-carboxylate (**S29**) (0.38 g, 1.3 mmol, 1.0 equiv.) was dissolved in THF (dry; 6.4 mL). The solution was cooled to 0 °C (ice - water bath). KHMDS (1.0 M in THF) was then added drop-wise: the mixture turned locally to yellow-orange upon the addition of each drop, and then reverted to colorless; it remained yellow after ca. 50% of the base had been added. The resulting yellow solution was stirred at 0 °C for 30 minutes, and the mixture was stirred at room temperature for 1.5 hours, becoming an orange suspension. The reaction was quenched by addition of sat. aq. $NaHCO_3$ (20 mL). The aqueous layer was separated and extracted with Et_2O (3 x 30 mL). The combined organic layers were washed with brine, dried over Na_2SO_4, filtered, and concentrated under vacuum. The resulting pale orange, crude oil was submitted to column chromatography (Biotage flash chromatographer, SiO_2; EtOAc in pentane, 0 to 45%) to provide methyl 3-(4-

(trifluoromethyl)phenyl)bicyclo[1.1.0]butane-1-carboxylate (0.26 g, 1.0 mmol, 79% yield) as a pale yellow oil.

^{1}H NMR (400 MHz, Chloroform-*d*) δ 7.55 (d, *J* = 8.2 Hz, 2H, Ar*H*), 7.38 (d, *J* = 8.2 Hz, 2H, Ar*H*), 3.50 (s, 3H, OCH_3), 2.95 (t, *J* = 1.1 Hz, 2H, CH_2), 1.67 (s, 2H, CH_2).
^{19}F NMR (376 MHz, Chloroform-*d*) δ -62.5.

In a 25 mL, two-necked, round-bottomed flask, methyl 3-[4-(trifluoromethyl)phenyl]bicyclo[1.1.0]butane-1-carboxylate (0.26 g, 1.0 mmol, 1.0 equiv.) was dissolved in THF (dry; 5.1 mL). The resulting colorless solution was cooled to 0 °C (ice - water bath). Methoxy(methyl)amine hydrochloride (0.12 g, 1.2 mmol, 1.2 equiv.) was then added. The resulting mixture was stirred at 0 °C for 5 minutes. ^{i}Propyl magnesium chloride (2.0 M in THF; 1.2 mL, 2.4 mmol, 2.4 equiv.) was added slowly. The mixture was stirred at 0 °C for 1 hour. It was then diluted with Et_2O, and the reaction was then quenched by addition of sat. aq. NH_4Cl (10 mL). The aqueous layer was separated and extracted with Et_2O (3 x 15 mL). The combined organic layers were washed with brine, dried over Na_2SO_4, filtered, and concentrated under vacuum. The resulting pale yellow crude oil was submitted to column chromatography (Biotage flash chromatographer, SiO_2; EtOAc in pentane, 5 to 50%) to provide N-methoxy-N-methyl-O-(3-(4-(trifluoromethyl)phenyl)bicyclo[1.1.0]butane-1-carbonyl)hydroxylamine (**S30**) (0.21 g, 0.73 mmol, 72% yield) as a colorless oil.

^{1}H NMR (400 MHz, Chloroform-*d*) δ 7.54 (d, *J* = 8.2 Hz, 2H, Ar*H*), 7.41 (d, *J* = 8.2 Hz, 2H, Ar*H*), 3.68 (s, 3H, CH_3), 3.11 (s, 3H, CH_3), 3.02 (s, 2H, CH_2), 1.66 (s, 2H, CH_2).
^{19}F NMR (376 MHz, Chloroform-*d*) δ -62.4.

In a 50 mL, two-necked, round-bottomed flask, 2-bromonaphthalene (**S13**) (0.18 g, 0.88 mmol, 1.2 equiv.) was dissolved in a THF (dry; 3.0 mL). The clear, colorless solution was chilled to -78 °C (dry ice - water bath). ^{n}BuLi (2.5 M in hexanes; 0.34 mL, 0.84mmol, 1.15 equiv.) was then added drop-wise: the resulting mixture became yellow at first, and then very turbid remaining yellow. The suspension was stirred at -78 °C for 45 minutes. A solution of N-methoxy-N-methyl-3-(4-(trifluoromethyl)phenyl)bicyclo[1.1.0]butane-1-carboxamide (**S30**) (0.21 g, 0.73 mmol, 1.0 equiv.) in THF (1.0 mL) was added slowly at the same temperature. The yellow, turbid solution remained as such until it was stirred at -78 °C. After 60 minutes, the cooling bath was removed, and the mixture was stirred at room temperature for another 30 minutes, becoming clear and turning to dark green. Aq. $NaHCO_3$ (10 mL) was then added. The aqueous layer was separated and extracted with Et_2O (4 x 40 mL). The combined organic layers were washed with brine, dried over Na_2SO_4, filtered, and concentrated under vacuum

(heating bath at < 40 °C (!!)). The resulting pale yellow crude oil was submitted to column chromatography (Biotage flash chromatographer, SiO_2; EtOAc in pentane, 0 to 40%) to provide naphthalen-2-yl(3-(4-(trifluoromethyl)phenyl)bicyclo[1.1.0]butan-1-yl)methanone (**1m**) (0.18 g, 0.52 mmol, 72% yield) as a colorless, crystalline solid, which was stored at -60 °C.

M.P. 127.5-130.9 °C (apparent decomposition).

R_f (pentane/EtOAc 9/1) 0.45.

^{1}H NMR (400 MHz, Chloroform-*d*) δ 8.17 (s, 1H, Ar*H*), 7.90 (d, *J* = 7.9 Hz, 1H, Ar*H*), 7.85 (d, *J* = 8.0 Hz, 1H, Ar*H*), 7.79 (d, *J* = 8.6 Hz, 1H, Ar*H*), 7.61 – 7.52 (m, 3H, Ar*H*), 7.46 (d, *J* = 8.2 Hz, 2H, Ar*H*), 7.24 (d, *J* = 8.2 Hz, 2H, Ar*H*), 3.26 (d, *J* = 1.2 Hz, 2H, C*H_2*), 2.04 (s, 2H, C*H_2*).

^{19}F NMR (376 MHz, Chloroform-*d*) δ -62.5.

^{13}C NMR (101 MHz, Chloroform-*d*) δ 195.6, 137.5 (m), 135.2 (d, *J* = 2.3 Hz), 132.2, 130.1, 129.3, 129.1 (q, *J* = 32.6 Hz), 128.2, 128.2, 127.8, 126.8, 126.3, 125.4 (q, *J* = 3.8 Hz), 125.6 (q, *J* = 3.8 Hz), 124.3, 122.8, 38.0, 36.7, 31.9.

IR ($\tilde{\nu}_{max}$, cm^{-1}) 3058 (w), 2956 (w), 1632 (m), 1620 (m), 1469 (w), 1411 (m), 1357 (m), 1324 (s), 1167 (m), 1119 (s), 1065 (m), 1015 (w), 994 (w), 943 (w), 910 (w), 867 (w), 842 (m), 778 (m), 756 (m).

HRMS (ESI/QTOF) m/z: $[M + H]^+$ Calcd for $C_{22}H_{16}F_3O^+$ 353.1148; Found 353.1146.

1-(Bicyclo[1.1.0]butan-1-yl)pentan-1-one (1m)

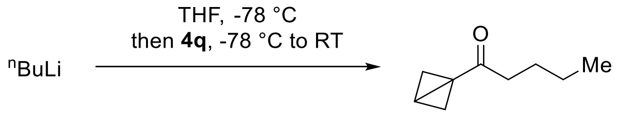

1m

Following a reported procedure,[2] in a 25 mL, two-necked, round-bottomed flask, ^{n}BuLi (2.5 M in hexanes; 0.62 mL, 1.5 mmol, 1.05 equiv.) was diluted in THF (dry; 7.3 mL). The clear, colorless solution was chilled to -78 °C (dry ice - water bath) and stirred at such a temperature for 10 minutes. A solution of N-methoxy-N-methylbicyclo[1.1.0]butane-1-carboxamide (**4q**) (1.4 mL of a 1.0 M solution in THF; 1.4 mmol, 1.0 equiv.) was added slowly at the same temperature. No change of appearance of the mixture was observed. The mixture was stirred at -78 °C for 30 minutes and then at room temperature for another 1.5 hours. To the clear and now pale yellow reaction mixture was then added sat. aq. $NaHCO_3$ (20 mL). The aqueous layer was separated and extracted with Et_2O (4 x 20 mL). The combined organic layers were washed with brine, dried over Na_2SO_4, filtered, and concentrated under vacuum (heating bath at < 40 °C (!!)). The resulting pale yellow oil was submitted to column chromatography (SiO_2; Et_2O/pentane, 1/9 to 1/3) to provide 1-(bicyclo[1.1.0]butan-1-yl)pentan-1-one (**1m**) (0.149 g,

1.08 mmol, 77% yield) as a colorless oil. The latter was dissolved in DCM (dry: 4.3 mL), and the resulting 0.25 M solution was immediately stored at -60 °C.

^{1}H NMR (400 MHz, Methylene Chloride-*d*$_2$) δ 2.43–2.36 (m, 4H, C*H*$_2$), 2.10 (ddd, *J* = 6.3, 3.4, 2.9 Hz, 1H), 1.59–1.48 (m, 2H, C*H*$_2$), 1.36–1.24 (m, 2H, C*H*$_2$), 1.17–1.13 (m, 2H, C*H*$_2$), 0.89 (t, *J* = 7.3 Hz, 3H, C*H*$_3$).

^{1}H-NMR signals correspond to the ones reported in the literature.[4]

Bicyclo[1.1.0]butan-1-yl(cyclohexyl)methanone (1n)

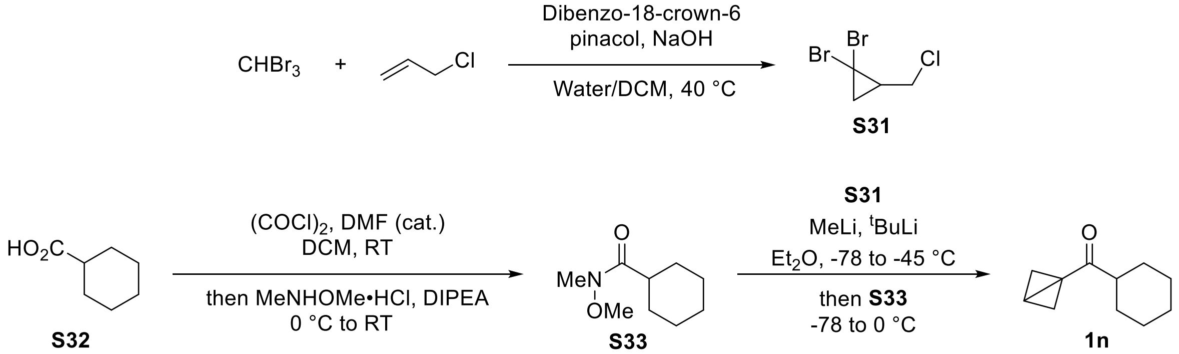

Following a reported procedure,[8] a 100 mL, two-necked, round-bottomed flask, equipped with an air condenser (Findenser©), was flushed with Ar for 5 minutes. It was then charged with dibenzo-18-crown-6 (DB18C6) (0.58 g, 1.6 mmol, 5 mol%) and pinacol (0.15 g, 1.3 mmol, 4 mol%). DCM (distilled; 12.4 mL) was added by syringe to form a clear, colorless solution. To the latter were added allyl chloride (5.2 mL, 64 mmol, 2.0 equiv.) and bromoform (2.8 mL, 32 mmol, 1.0 equiv.). Finally, a solution of NaOH (12.2 g, 304 mmol, 9.5 equiv.) in water (12.4 mL) was also added rapidly under stirring: the mixture became immediately yellow, then orange, and spontaneously warmed from 22.5 to ca. 27 °C. It was heated to 40 °C and stirred overnight, rapidly darkening further to brown-black. After 19 hours, TLC analysis (elution with pentane) showed the complete consumption of bromoform. The brown-black suspension was then allowed to cool down to room temperature and subsequently poured into a 500 mL beaker containing pentane (50 mL). The mixture was gently swirled with a glass stick and sonicated for 5 minutes. The solids and the aqueous layer were then allowed to sediment over a time of 30 minutes. The supernatant pentane layer was carefully separated and passed through a pad of celite and SiO_2. Attention was paid not to pour the black solids and the aqueous layer over the pad. A disc of filtering paper on top of the pad was used to recollect the solids poured together the organic solution. The remaining dark slurry was washed with two addition portions of pentane (70 mL each), by repeating the same procedure described above. The organic filtrate was then concentrated under reduced pressure (rotatory evaporation; 40 °C, pressure not below 500 mbar) to give a pale yellow oil. The latter was re-dissolved in pentane (ca. 40 mL) and filtered through a thin pad of SiO_2 packed over a pad of $MgSO_4$ (a disc of filtering

paper was used to separate the two pads). The double filtering pad was washed with pentane (3 x 40 mL). The clear, colorless filtrate was then concentrated under vacuum (rotatory evaporation; 40 °C, pressure not below 500 mbar) to give 1,1-dibromo-2-(chloromethyl)cyclopropane (**S31**) (3.3 g, 13 mmol, 42% yield) as a light, colorless liquid.

^{1}H NMR (400 MHz, Chloroform-*d*) δ 3.65 (d, *J* = 7.4 Hz, 2H, ClCH_2), 2.04 (dq, *J* = 10.3, 7.4 Hz, 1H, CHCH_2), 1.93 (dd, *J* = 10.3, 7.6 Hz, 1H, CHCH_2), 1.48 (t, *J* = 7.5 Hz, 1H, C*H*CH_2).
^{1}H-NMR signals correspond to the ones reported in the literature.[9]

Following a reported procedure,[10] in a 100 mL, two-necked, round-bottomed flask, cyclohexanecarboxylic acid (**S32**) (0.64 g, 5.0 mmol, 1.0 equiv.) was dissolved in DCM (dry; 16.5 mL). Oxalyl chloride (0.47 mL, 5.5 mmol, 1.1 equiv.) was added, followed by 5 drops of DMF. Immediately, gas release started, which ceased approximately 1.5 hours later. The colorless, clear solution was stirred at room temperature for 2 hours. It was then concentrated under reduced pressure to provide the acyl chloride as a yellow oil. In a second 100 mL, two-necked, round-bottomed flask, N-methoxy-N-methylamine hydrochloride (0.49 g, 5.0 mmol, 1.0 equiv.) was suspended in DCM (16.5 mL). The clear solution was cooled to 0 °C (ice-water bath), prior to the addition of triethylamine (1.4 mL, 11 mmol, 2.1 equiv.). The solution converted into a suspension, which was stirred at 0 °C for 5 minutes. Finally, the neat, crude acyl chloride was added slowly at the same temperature. The resulting bright yellow suspension was stirred at room temperature for 2 hours. The mixture was diluted with DCM (20 mL), and the reaction was then quenched by addition of aq. HCl (1.0 M; ca. 20 mL). The organic layer was separated and washed with another portion of aq. HCl (1.0 M; ca. 20 mL). The combined aqueous layers were back-extracted with DCM (2 x 20 mL). The combined organic layers were washed with sat. aq. $NaHCO_3$ (2 x 30 mL), brine, dried over Na_2SO_4, filtered, and concentrated under reduced pressure. Crude N-methoxy-N-methylcyclohexanecarboxamide (**S33**) (0.79 g, 4.6 mmol, 93%) was obtained as a pale yellow oil, which was found analytically pure and used as such in the following step.

^{1}H NMR (400 MHz, Chloroform-*d*) δ 3.69 (s, 3H, CH_3), 3.17 (s, 3H, CH_3), 2.68 (m, 1H, (CO)C*H*), 1.85–1.71 (m, 4H, Cy*H*), 1.68 (dd, *J* = 5.3, 2.9 Hz, 1H, Cy*H*), 1.56–1.40 (m, 2H, Cy*H*), 1.38–1.17 (m, 3H, Cy*H*).
^{1}H-NMR signals correspond to the ones reported in the literature.[4]

Following a reported procedure,[4] a 100 mL, two-necked, round-bottomed flask was charged with 1,1-dibromo-2-(chloromethyl)cyclopropane (**S31**) (0.36 mL, 3.0 mmol, 1.0 equiv.) and Et_2O (dry; 17.5 mL). The resulting colorless solution was chilled to -78 °C (dry ice - acetone

bath). MeLi (1.6 M in Et_2O; 1.9 mL, 3.0 mmol, 1.0 equiv.) was added slowly. The initially clear mixture became slightly turbid. It was stirred at -78 °C for 30 minutes, and then at -50 ~-40 °C (liquid nitrogen in a 50 : 50 mixture of water and MeOH) for 60 minutes. The turbid mixture was then chilled back to -78 °C, prior to the drop-wise addition of ^{t}BuLi (1.7 M in pentane; 1.8 mL, 3.0 mmol, 1.0 equiv.). After stirring at this temperature for another 30 minutes, a solution of N-methoxy-N-methylcyclohexanecarboxamide (**S33**) (0.77 g, 4.5 mmol, 1.5 equiv.) in Et_2O (dry; 0.75 mL) was added slowly. Stirring was continued at -78 °C for 30 minutes and then at 0 °C (water - ice bath) for 1 hour. The reaction was then quenched by addition of sat. aq. NH_4Cl (25 mL). The aqueous layer was separated and extracted with Et_2O (3 x 30 mL). The combined organic layers were washed with brine, dried over Na_2SO_4, filtered, and concentrated under vacuum. The resulting pale yellow, crude oil was submitted to column chromatography (Biotage flash chromatographer, SiO_2; Et_2O in pentane, 0 to 25%) to afford bicyclo[1.1.0]butan-1-yl(cyclohexyl)methanone (**1n**) (0.15 g, 0.93 mmol, 31% yield) as a colorless oil, which was immediately dissolved in DCM (dry; 3.7 mL) in order to obtain a 2.5 M solution. The latter was stored at -60 °C.

^{1}H NMR (400 MHz, Chloroform-*d*) δ 2.47 (tt, *J* = 11.6, 3.2 Hz, 1H, (CO)C*H*), 2.38 (dt, *J* = 3.4, 1.0 Hz, 2H, C*H_2*), 2.15 (p, *J* = 3.1 Hz, 1H, C*H*), 1.83–1.72 (m, 4H, Cy*H*), 1.71–1.60 (m, 1H, Cy*H*)), 1.50–1.36 (m, 2H, Cy*H*)), 1.33–1.20 (m, 3H, Cy*H*)), 1.14 (dt, *J* = 2.6, 1.0 Hz, 2H, C*H_2*). ^{1}H-NMR signals correspond to the ones reported in the literature.[4]

1-(Bicyclo[1.1.0]butan-1-yl)-2,2-dimethylpropan-1-one (1o)

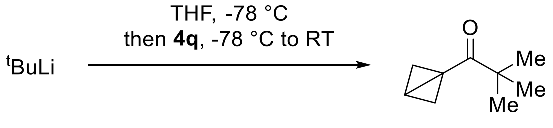

1o

Following a reported procedure,[2] in a 25 mL, two-necked, round-bottomed flask, ^{t}BuLi (1.7 M in pentane; 0.90 mL, 1.5 mmol, 1.5 equiv.) was diluted in THF (dry; 7.3 mL). The clear, colorless solution was chilled to -78 °C (dry ice - water bath) and stirred at such a temperature for 10 minutes. A solution of N-methoxy-N-methylbicyclo[1.1.0]butane-1-carboxamide (**4q**) (1.0 mL of a 1.0 M solution in THF; 1.0 mmol, 1.0 equiv.) was added slowly at the same temperature. No change of appearance of the mixture was observed. The was stirred at -78 °C for 30 minutes and then at room temperature for another hour. To the clear and now pale yellow reaction mixture was then added sat. aq. $NaHCO_3$ (20 mL). The aqueous layer was separated and extracted with Et_2O (4 x 20 mL). The combined organic layers were washed with brine, dried over Na_2SO_4, filtered, and concentrated under vacuum (heating bath at < 40 °C (!!)). The resulting pale yellow oil was submitted to column chromatography (SiO_2;

Et_2O/pentane, 1/9 to 1/7) to provide 1-(bicyclo[1.1.0]butan-1-yl)-2,2-dimethylpropan-1-one (**1o**) (0.106 g, 0.767 mmol, 77% yield) as a colorless oil. The latter was dissolved in DCM (dry: 3.0 mL), and the resulting 0.25 M solution was immediately stored at -60 °C.

^{1}H NMR (400 MHz, Methylene Chloride-*d_2*) δ 2.46 (d, *J* = 3.3 Hz, 2H, C*H_2*), 2.30 (m, 1H, *CH)*, 1.17 (s, 9H, C(C*H_3*)$_3$), 1.11–1.07 (m, 2H, C*H_2*).
^{1}H-NMR signals correspond to the ones reported in the literature.[4]

1.2 Synthesis of the dienol ethers

tert-Butyl((3-methylbuta-1,3-dien-2-yl)oxy)diphenylsilane (2a)

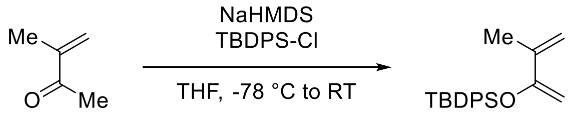

S34 **2a**

Following a reported procedure,[11] In a 100 mL, two-necked, round-bottomed flask NaHMDS (1.0 M in THF; 16 mL, 16 mmol, 1.3 equiv.) was diluted with THF (dry; 32 mL). The resulting yellow solution was chilled to -78 °C (dry ice - acetone bath). 3-Methylbut-3-en-2-one (**S34**) (1.2 mL, 12 mmol, 1.0 equiv.) was then added drop-wise at the same temperature. The mixture, still looking clear, turned to yellow-green. It was stirred at -78 °C for 30 minutes prior to the addition of *tert*-butyldiphenylchlorosilane (3.6 mL, 14 mmol, 1.1 equiv.), drop-wise by syringe. The now yellow mixture was then stirred for 40 hours. After this time, the reaction was quenched by addition of sat. aq. $NaHCO_3$ (20 mL). The aqueous layer was then separated and extracted with ether (3 x 30 mL). The combined organic layers were washed with brine, dried over Na_2SO_4, filtered, and concentrated under vacuum. The resulting crude oil was submitted to column chromatography (SiO_2; packing with pentane/Et_3N 250/1; elution with pentane/EtOAc/Et_3N 200/1/1) to afford *tert*-butyl((3-methylbuta-1,3-dien-2-yl)oxy)diphenylsilane (**2a**) (2.78 g, 8.62 mmol, 70% yield) as a viscous, colorless oil that became an amorphous solid on standing at room temperature.

^{1}H NMR (400 MHz, Chloroform-*d*) δ 7.79–7.69 (m, 4H, Ph*H* in TBDPS), 7.48–7.31 (m, 6H, Ph*H* in TBDPS), 5.75 (d, *J* = 2.5 Hz, 1H, C=C*H_2*), 5.07 (m, 1H, C=C*H_2*), 4.31 (q, *J* = 1.1 Hz, 1H, C=C*H_2*), 3.94 (dt, *J* = 2.6, 1.3 Hz, 1H, C=C*H_2*), 1.89 (m, 3H, CH_3), 1.05 (m, 9H, C(C*H_3*)$_3$ in TBDPS).
^{1}H-NMR signals correspond to the ones reported in the literature.[11]

tert-Butyldimethyl((3-methylbuta-1,3-dien-2-yl)oxy)silane (2a')

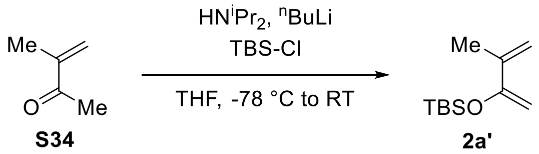

Following a reported procedure,[12] in a 50 mL, two-necked, round-bottomed flask, di*iso*propyl amine (1.3 mL, 9.3 mmol, 1.16 equiv.) was dissolved in THF (dry; 20 mL). The colorless solution was chilled to -78 °C (dry ice - acetone bath), and then ^{n}BuLi (2.5 M in hexanes; 3.7 mL, 9.3 mmol, 1.16 equiv.) was added slowly at the same temperature. Once the addition was completed, the mixture was warmed to 0 °C (ice - water bath) and stirred at this temperature for 30 minutes. The still colorless LDA solution was chilled back to -78 °C, and a solution of 3-methylbut-3-ene-2-one (**S34**) (0.78 mL, 8.0 mmol, 1.0 equiv.) in THF (2.5 mL) was added dropwise. The mixture was stirred at -78 °C for 30 minutes, prior to the slow addition of TBS-OTf (2.1 mL, 9.3 mmol, 1.16 equiv.). The colorless mixture was then stirred overnight, while allowing it to warm to room temperature. After 17 hours, the mixture was diluted with pentane (20 mL) and ether (20 mL) and the reaction was quenched by addition of sat. aq. $NaHCO_3$ (20 mL) and water (10 mL). The aqueous layer was separated, and the organic layer was washed with sat. aq. $NaHCO_3$ (20 mL), and brine, dried over Na_2SO_4, filtered, and concentrated under vacuum. The resulting pale yellow crude oil was submitted to column chromatography (SiO_2; Et_3N in pentane, 1/250) to provide a colorless oil. The latter was submitted to kugelrohr distillation (15 mbar, 90 °C) to provide *tert*-butyldimethyl((3-methylbuta-1,3-dien-2-yl)oxy)silane (**2a'**) (0.96 g, 4.8 mmol, 60% yield) as a colorless oil.

^{1}H NMR (400 MHz, Chloroform-*d*) δ 5.44 (d, *J* = 1.9 Hz, 1H, C=CH_2), 4.96 (s, 1H, C=CH_2), 4.48 (s, 1H, C=CH_2), 4.32 (s, 1H, C=CH_2), 1.88 (s, 3H, CH_3), 0.97 (s, 9H, C(CH_3)$_3$ in TBS), 0.17 (s, 6H, CH_3 in TBS).
^{1}H-NMR signals correspond to the ones reported in the literature.[12]

Triisopropyl((3-methylbuta-1,3-dien-2-yl)oxy)silane (2a'')

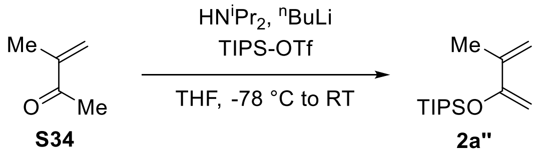

Following a modified version of a reported procedure,[12] in a 50 mL, two-necked, round-bottomed flask, di*iso*propyl amine (1.0 mL, 7.0 mmol, 1.16 equiv.) was dissolved in THF (dry; 15 mL). The colorless solution was chilled to -78 °C (dry ice - acetone bath), and then ^{n}BuLi (2.5 M in hexanes; 2.8 mL, 7.0 mmol, 1.16 equiv.) was added slowly at the same temperature. Once the addition was completed, the mixture was warmed to 0 °C (ice - water bath) and stirred at this temperature for 30 minutes. The still colorless LDA solution was chilled back to -78 °C, and a solution of 3-methylbut-3-ene-2-one (**S34**) (0.60 mL, 6.0 mmol, 1.0 equiv.) in THF (2.0 mL) was added

dropwise. The mixture was stirred at -78 °C for 30 minutes, prior to the slow addition of TIPS-OTf (1.9 mL, 7.0 mmol, 1.16 equiv.). The colorless mixture was then stirred overnight, while allowing it to warm to room temperature. The mixture was then diluted with pentane (30 mL) and the reaction was quenched by addition of sat. aq. $NaHCO_3$ (20 mL) and water (10 mL). The aqueous layer was separated and extracted with pentane (2 x 30 mL). The organic layers were washed with sat. aq. $NaHCO_3$ (2 x 30 mL), and brine, dried over Na_2SO_4, filtered, and concentrated under vacuum. The resulting pale yellow crude oil was submitted to column chromatography (SiO_2; Et_3N in pentane, 1/250) to provide tri*iso*propyl((3-methylbuta-1,3-dien-2-yl)oxy)silane (**2a''**) (1.1 g, 4.7 mmol, 78% yield) as a colorless oil.

^{1}H NMR (400 MHz, Chloroform-*d*) δ 5.51 (m, 1H, C=C*H₂*), 4.97 (m, 1H, C=C*H₂*), 4.44 (s, 1H, C=C*H₂*), 4.32 (s, 1H, C=C*H₂*), 1.89 (m, 3H, C*H₃*), 1.25 (m, 3H, C*H*(CH_3)$_2$ in TIPS), 1.11 (d, *J* = 7.1 Hz, 18H, CH(C*H₃*)$_2$ in TIPS).

^{1}H-NMR signals correspond to the ones reported in the literature.[13]

(Buta-1,3-dien-2-yloxy)(tert-butyl)diphenylsilane (2b)

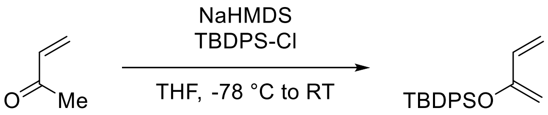

S35 **2b**

Following a reported procedure,[11] in a 50 mL, two-necked, round-bottomed flask, NaHMDS (1.0 M in THF; 6.5 mL, 6.5 mmol, 1.3 equiv.) was diluted with THF (dry; 13 mL). The yellow solution was cooled to -78 °C (dry ice - acetone bath). Methyl vinyl ketone (**S35**) (0.42 mL, 5.0 mmol, 1.0 equiv.) was added drop-wise. The resulting yellow solution was stirred at -78 °C for 40 minutes. TBDPS-Cl (1.5 mL, 5.6 mmol, 1.1 equiv.) was then added drop-wise at the same temperature. The mixture was then stirred over the weekend, while allowing it to warm to room temperature and becoming very turbid and orange-brown. After 3 days, the reaction was quenched by addition of sat. aq. $NaHCO_3$ (30 mL). The aqueous layer was extracted with Et_2O (3 x 35 mL). The combined organic layers were washed with brine, dried over Na_2SO_4, filtered, and concentrated under vacuum. The resulting orange crude oil was submitted to column chromatography (SiO_2; Pentane/Et_3N 249/1). (Buta-1,3-dien-2-yloxy)(*tert*-butyl)diphenylsilane (**2b**) (0.27 g, 0.86 mmol, 17% yield) was obtained as a colorless, viscous oil.

^{1}H NMR (400 MHz, Chloroform-*d*) δ 7.78–7.67 (m, 4H, Ph*H* in TBDPS), 7.47–7.34 (m, 6H, Ph*H* in TBDPS), 6.21 (dd, *J* = 16.9, 10.5 Hz, 1H, CH=CH_2), 5.80 (dd, *J* = 16.9, 1.9 Hz, 1H, CH=CH_2), 5.19 (dt, *J* = 10.5, 1.3 Hz, 1H, CH=CH_2), 4.16 (s, 1H, C=C*H₂*), 3.94 (t, *J* = 1.2 Hz, 1H, C=C*H₂*), 1.07 (s, 9H, C(C*H₃*)$_3$ in TBDPS).

^{1}H-NMR signals correspond to the ones reported in the literature.[11]

(Z)-tert-Butyl(penta-1,3-dien-3-yloxy)diphenylsilane (2c)

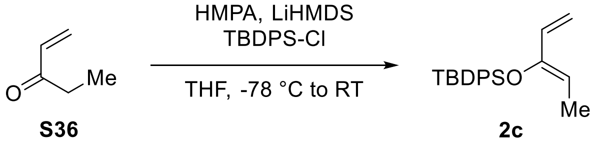

Following a slightly modified version of a reported procedure,[14] in a 100 mL, two-necked, round-bottomed flask, a mixture of THF (dry; 22.5 mL) and HMPA (dry; 22.5 mL) was chilled to -78 °C (dry ice - acetone bath) under stirring (the mixture completely froze into a solid within 5 minutes at this temperature). LiHMDS (1.0 M in THF; 9.0 mL, 9.0 mmol, 1.2 equiv.) was then added, which resulted in the solvent mixture to revert into a liquid, pale yellow solution. The latter was stirred at -78 °C for 5 minutes. A solution of ethyl vinyl ketone (**S36**) (0.74 mL, 7.5 mmol, 1.0 equiv.) in THF (dry; 8.0 mL), slowly. The yellow mixture was stirred at -78 °C for 60 minutes. A solution of TBDPS-Cl (2.1 mL, 8.2 mmol, 1.1 equiv.) in THF (2.2 mL) was then added drop-wise, still at -78 °C. The resulting clear, yellow solution was stirred for 3 hours, while allowing it to warm to room temperature. The reaction was then quenched by addition of water (50 mL). The aqueous layer was extracted with pentane (3 x 40 mL). The combined organic layers were washed with brine, dried over Na_2SO_4, filtered, and concentrated under vacuum. The resulting yellow-orange crude oil was submitted to column chromatography (SiO_2; Pentane/Et_3N 250/1) to provide (Z)-*tert*-Butyl(penta-1,3-dien-3-yloxy)diphenylsilane (**YYb**) (single geometrical isomer – Z-geometry assigned by on analogy with **2c**, *v. infra*; 1.43 g, 4.43 mmol, 59% yield) as a colorless solid.

M.P. 86.0-89.4 °C.
R_f (pentane) 0.50.
^{1}H NMR (400 MHz, Methylene Chloride-d_2) δ 7.82 – 7.75 (m, 4H, Ph*H* in TBDPS), 7.50 – 7.33 (m, 6H, Ph*H* in TBDPS), 6.10 (dd, *J* = 17.1, 10.8 Hz, 1H, C*H*=CH$_2$), 5.36 (d, *J* = 17.1 Hz, 1H, CH=C*H$_2$*), 4.86 (d, *J* = 10.9 Hz, 1H, CH=C*H$_2$*), 4.79 (q, *J* = 7.2 Hz, 1H, C=C*H*CH$_3$), 1.31 (d, *J* = 7.2 Hz, 3H, C=CHC*H$_3$*), 1.08 (s, 9H, C(C*H$_3$*)$_3$ in TBDPS).
^{13}C NMR (101 MHz, Methylene Chloride-d_2) δ 150.1, 136.1, 136.0, 134.6, 130.2, 128.1, 112.5, 108.7, 27.1, 20.4, 12.5.
IR ($\tilde{\nu}_{max}$, cm^{-1}) 3071 (w), 3047 (w), 3023 (w), 2964 (m), 2935 (m), 2892 (w), 2858 (m), 1652 (w), 1607 (w), 1591 (w), 1472 (w), 1428 (m), 1409 (w), 1388 (w), 1346 (m), 1317 (w), 1291 (w), 1206 (m), 1112 (m), 1084 (m), 1061 (m), 1014 (w), 985 (w), 935 (w), 899 (m), 823 (m), 795 (w), 741 (m), 702 (s).
HRMS (ESI/QTOF) m/z: [M + H]$^+$ Calcd for $C_{21}H_{27}OSi^+$ 323.1826; Found 323.1832.

(Z)-tert-Butyl((2-methylpenta-1,3-dien-3-yl)oxy)diphenylsilane (2d)

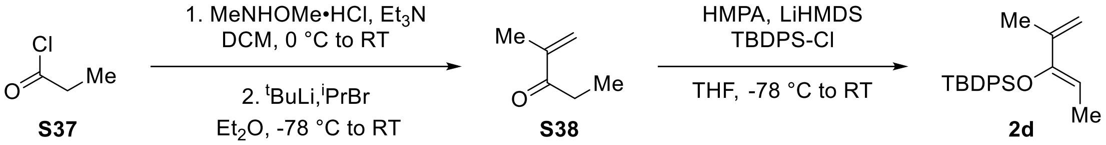

Following a modified version of a reported procedure,[15] in a 100 mL, two-necked, round-bottomed flask, N,O-dimethylhydroxylamine hydrochloride (0.78 g, 8.0 mmol, 1.0 equiv.) was suspended in DCM (dry; 20 mL). After the suspension was cooled to 0 °C (ice - water bath), triethylamine (2.2 mL, 16 mmol, 2.0 equiv.) was added drop-wise over 5 minutes. The mixture became a thicker, homogeneous white suspension, which was stirred at 0 °C for 15 minutes. Propionyl chloride (**S37**) (0.70 mL, 8.0 mmol, 1.0 equiv.) was then added drop-wise. The cooling bath was removed, and the resulting suspension was stirred at room temperature for 2 hours. Upon dilution with DCM (20 mL), the reaction was then quenched by addition of sat. aq. $NaHCO_3$ (40 mL). The aqueous layer was separated, and the organic one was washed with aq. HCl (1.0 M; 30 mL), brine, dried over Na_2SO_4, filtered, and concentrated under vacuum to provide the desired Weinreb amide as a colorless oil. Meanwhile, in a second 100 mL, two-necked, round-bottomed flask, 2-bromopropene (0.81 mL, 9.2 mmol, 1.15 equiv.) was dissolved in Et_2O (dry; 32 mL). The clear solution was chilled to -78 °C (dry ice - acetone bath) prior to the drop-wise addition of ^{t}BuLi (1.7 M in pentanes; 5.0 mL, 8.4 mmol, 1.05 equiv.). The resulting clear mixture was stirred at -78 °C for 50 minutes. A solution of the previously prepared Weinreb amide in Et_2O (2 mL) was then added slowly. The solution became turbid. It was stirred at -78 °C for 30 minutes, and then warmed up to room temperature. This resulted in the solution becoming a milky suspension. The latter was stirred at room temperature for another 30 minutes, after which time TLC analysis (pentane/Et_2O 95/5) showed the complete conversion of the starting material. The reaction was therefore quenched by addition of sat. aq. $NaHCO_3$ (35 mL) under vigorous stirring, which led to the dissolution of the solids. The aqueous layer was separated and extracted with Et_2O (3 x 30 mL). The combined organic layers were washed with brine, dried over Na_2SO_4, filtered, and concentrated in vacuum. The resulting colorless crude oil was submitted to column chromatography (SiO_2; pentane/Et_2O 245/5 to 235/15) to provide 2-methylpent-1-en-3-one (**S38**) (70% in pentane; 0.25 g, 1.8 mmol, 23% yield – low yield due to the partial loss of this volatile compound during rotatory evaporation) as a colorless oil.

Following a slightly modified version of a reported procedure,[14] in a 50 mL, two-necked, round-bottomed flask, a mixture of THF (dry; 12.2 mL) and HMPA (dry; 12.2 mL) was chilled to -78 °C (dry ice - acetone bath) under stirring (the mixture complety froze into a solid within 5 minutes at this temperature). LiHMDS (1.0 M in THF; 4.9 mL, 4.9 mmol, 1.2 equiv.) was then added, which resulted in partial reversion of the solid mixture into a liquid solution (yellow-orange). The latter was stirred at -78 °C for 5 minutes. A solution of 2-methylpent-1-en-3-one (**S38**) (0.40 g, 4.1 mmol, 1.0 equiv.) in THF (dry; 8.0 mL), slowly. The orange mixture was stirred at -78 °C for 60 minutes. A solution of TBDPS-Cl (1.2 mL, 4.5 mmol, 1.2 equiv.) in THF (1.0 mL) was then added drop-wise, still at -78 °C. The resulting clear, yellow solution was stirred for 3 hours, while allowing it to warm to room temperature. The reaction was then quenched by addition of water (50 mL). The aqueous layer was extracted with Et_2O (3 x 40 mL). The combined organic layers were washed with brine, dried over Na_2SO_4, filtered, and concentrated under vacuum. The resulting yellow-

orange crude oil was submitted to column chromatography (SiO_2; Pentane/Et_3N 250/1) to provide *tert*-butyl((2-methylpenta-1,3-dien-3-yl)oxy)diphenylsilane (**2d**) (single geometrical isomer – Z-geometry assigned based on NOESY analysis; 0.50 g, 1.5 mmol, 36% yield) as a very viscous, colorless oil.

R_f (pentane) 0.45.
^{1}H NMR (400 MHz, Methylene Chloride-d_2) δ 7.76 (dd, *J* = 8.0, 1.5 Hz, 4H, Ph*H* in TBDPS), 7.46 – 7.32 (m, 6H, Ph*H* in TBDPS), 5.31 (m, 1H, C=CH_2; partial overlap with the peak of methylene chloride-d_2), 4.87 (m, 1H, C=C*H*CH_3), 4.83 (s, 1H, C=CH_2), 1.80 (s, 3H, (CH_3)C=C), 1.23 (d, *J* = 7.1 Hz, 3H, C=CHCH_3), 1.05 (s, 9H, C(CH_3)$_3$ in TBDPS).
^{13}C NMR (101 MHz, Methylene Chloride-d_2) δ 151.4, 141.4, 136.0, 134.7, 130.1, 128.0, 112.6, 105.4, 27.2, 20.9, 20.5, 12.8.
IR ($\tilde{\nu}_{max}$, cm^{-1}) 3072 (w), 3051 (w), 2961 (m), 2932 (m), 2893 (w), 2859 (m), 1648 (w), 1613 (w), 1472 (w), 1447 (w), 1429 (m), 1388 (w), 1360 (w), 1339 (m), 1309 (w), 1186 (w), 1152 (m), 1110 (m), 1076 (m), 1005 (w), 940 (w), 894 (w), 862 (w), 822 (w), 798 (w), 741 (m)
HRMS (ESI/QTOF) m/z: $[M + H]^+$ Calcd for $C_{22}H_{29}OSi^+$ 337.1982; Found 337.1987.

(E)-tert-Butyl((3-methylpenta-1,3-dien-2-yl)oxy)diphenylsilane (2e)

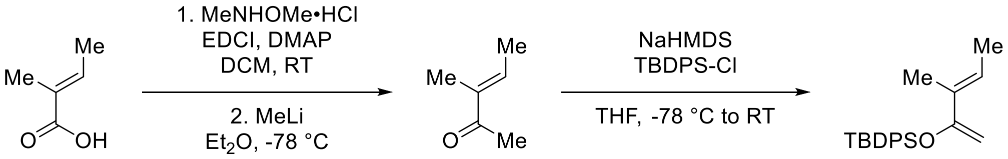

S39 **S40** **2e**

Following a reported procedure,[16] in a 100 mL, single-necked, round-bottomed flask, *trans*-2-methyl-2-butenoic acid (**S39**) (1.00 g, 10.0 mmol, 1.0 equiv.) was dissolved in DCM (60 mL). To the clear, colorless solution was added N,O-dimethylhydroxylamine hydrochloride (1.46 g, 15.0 mmol, 1.5 equiv.). A suspension was formed, to which EDC hydrochloride (2.88 g, 15.0 mmol, 1.5 equiv.) and DMAP (1.83 g, 15.0 mmol, 1.5 equiv.) were subsequently added. The resulting clear, pale yellow solution was stirred at room temperature overnight. After 17 hours, it was diluted with DCM (40 mL), and treated with sat. aq. $NaHCO_3$ (50 mL). The organic layer was then washed with aq. HCl (1.0 M; 2 x 40 mL), and brine, dried over Na_2SO_4, filtered and concentrated under vacuum. Crude (E)-N-methoxy-N,2-dimethylbut-2-enamide (1.27 g, 8.87 mmol, 89% yield) was obtained as a pale yellow oil, which was found pure enough to be used directly in the following step without further purification.

In a 100 mL, two-necked, round-bottomed flask, crude (E)-N-methoxy-N,2-dimethylbut-2-enamide (1.27 g, 8.87 mmol, 1.0 equiv.) was dissolved in Et_2O (dry; 46 mL). The colorless solution was chilled to -78 °C (dry ice - acetone bath). Methyl lithium (1.6 M solution in Et_2O; 3.8 mL, 6.1 mmol, 1.1 equiv.) was added drop-wise under stirring. The resulting pale yellow solution was stirred at -78 °C for 2 hours. The reaction was quenched by addition of sat. aq. $NaHCO_3$ (30 mL).

The aqueous layer was separated and extracted with Et_2O (3 x 30 mL). The combined organic layers were washed with brine, dried over Na_2SO_4, filtered, and concentrated under vacuum. Crude (E)-3-methylpent-3-en-2-one (**S40**) (0.70 g, 7.1 mmol, 80% yield) was obtained as a pale yellow oil, which was used directly in the following step, without further purification.

^{1}H NMR (400 MHz, Chloroform-*d*) δ 6.74 (qd, *J* = 6.9, 1.3 Hz, 1H, C=C*H*CH_3), 2.29 (s, 3H, C*H*$_3$), 1.86 (dd, *J* = 6.9, 1.0 Hz, 3H, C=CHC*H*$_3$), 1.80–1.72 (m, 3H, HC=CC*H*$_3$).
^{1}H-NMR signals correspond to the ones reported in the literature.[17]

Following a reported procedure,[11] in a 50 mL, two-necked, round-bottomed flask NaHMDS (1.0 M in THF; 4.4 mL, 4.4 mmol, 1.1 equiv.) was diluted with THF (dry; 10.4 mL). The resulting yellow solution was chilled to -78 °C (dry ice - acetone bath). (E)-3-Methylpent-3-en-2-one (**S40**) (0.40 mL, 4.0 mmol, 1.0 equiv.) was then added drop-wise at the same temperature. The mixture, still looking clear, turned to yellow. It was stirred at -78 °C for 60 minutes prior to the addition of *tert*-butyldiphenylchlorosilane (1.0 mL, 4.0 mmol, 1.0 equiv.), drop-wise by syringe. The yellow mixture was then stirred for 16 hours, while allowing it to warm to room temperature. After this time, the mixture looked like a turbid, orange suspension. The reaction was quenched by addition of sat. aq. $NaHCO_3$ (40 mL). The aqueous layer was then separated and extracted with ether (3 x 50 mL). The combined organic layers were washed with brine, dried over Na_2SO_4, filtered, and concentrated under vacuum. The resulting crude oil was submitted to column chromatography (SiO_2; equilibration and elution with pentane/Et_3N 250/1) to afford (E)-*tert*-butyl((3-methylpenta-1,3-dien-2-yl)oxy)diphenylsilane (**2e**) (0.51, 1.5 mmol, 38% yield) as a viscous, colorless oil.

R_f (pentane) 0.46.
^{1}H NMR (400 MHz, Methylene Chloride-*d*$_2$) δ 7.73 (dd, *J* = 7.8, 1.5 Hz, 4H, Ph*H* in TBDPS), 7.47 – 7.35 (m, 6H, Ph*H* in TBDPS), 6.41 (q, *J* = 6.9 Hz, 1H, C=C*H*CH_3), 4.22 (s, 1H, C=C*H*$_2$), 3.81 (s, 1H, C=C*H*$_2$), 1.79 (d, *J* = 7.1 Hz, 3H, C=CHC*H*$_3$), 1.76 (s, 3H, C*H*$_3$), 1.04 (s, 9H, C(C*H*$_3$)$_3$ in TBDPS).
^{13}C NMR (101 MHz, Methylene Chloride-*d*$_2$) δ 157.5, 136.0, 133.5, 132.2, 130.3, 128.2, 123.1, 91.8, 26.9, 19.9, 14.2, 13.2.
IR ($\tilde{\nu}_{max}$, cm^{-1}) 3072 (w), 3051 (w), 2956 (m), 2932 (m), 2893 (w), 2856 (m), 1642 (w), 1594 (m), 1472 (w), 1429 (m), 1388 (w), 1343 (w), 1291 (s), 1152 (m), 1112 (m), 1028 (m), 1000 (m), 943 (w), 822 (m), 791 (m), 737 (m), 702 (s).
HRMS (ESI/QTOF) m/z: $[M + H]^+$ Calcd for $C_{22}H_{29}OSi^+$ 337.1982; Found 337.1979.

(E)-tert-Butyl(penta-1,3-dien-2-yloxy)diphenylsilane (2f)

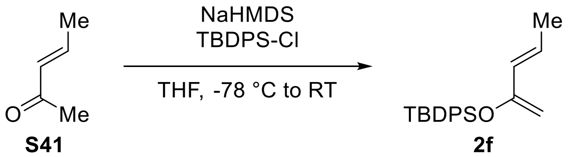

Following a reported procedure,[11] in a 25 mL, two-necked, round-bottomed flask, NaHMDS (1.0 M in THF; 5.3 mL, 5.3 mmol, 1.3 equiv.) was diluted with THF (dry; 8.2 mL). The yellow solution was cooled to -78 °C (dry ice - acetone bath). 3-Penten-2-one (**S41**) (mostly E; 0.38 mL, 4.1 mmol, 1.0 equiv.) was added as a solution in THF (2.0 mL), drop-wise. The resulting orange solution was stirred at -78 °C for 40 minutes. TBDPS-Cl (1.2 mL, 4.5 mmol, 1.1 equiv.) was then added drop-wise at the same temperature. The mixture was then stirred overnight, while allowing it to warm to room temperature and becoming orange-brown. After 40 hours, the reaction was quenched by addition of sat. aq. $NaHCO_3$ (15 mL). The aqueous layer was extracted with Et_2O (3 x 25 mL). The combined organic layers were washed with brine, dried over Na_2SO_4, filtered, and concentrated under vacuum. The resulting orange crude oil was submitted to column chromatography Biotage flash chromatographer, SiO_2; preliminarily deactivated with Et_3N in pentane, 2% v/v; elution with pure pentane). (E)-*tert*-Butyl(penta-1,3-dien-2-yloxy)diphenylsilane (**2f**) (0.49 g, 1.5 mmol, 37% yield) was obtained as a pale yellow, viscous oil.

R_f (pentane) 0.64.

^{1}H NMR (400 MHz, Chloroform-*d*) δ 7.74 (dt, *J* = 7.8, 2.8 Hz, 4H, Ph*H* in TBDPS), 7.48–7.33 (m, 6H, Ph*H* in TBDPS), 6.31 (m, 1H, CH=C*H*CH_3), 5.93 (dq, *J* = 15.2, 1.6 Hz, 1H), 4.02 (s, 1H, C=CH_2), 3.79 (s, 1H, C=CH_2), 1.84 (dd, *J* = 6.8, 1.5 Hz, 3H, CH=CHCH_3), 1.06 (s, 9H, C(CH_3)$_3$ in TBDPS).

^{13}C NMR (101 MHz, Methylene Chloride-d_2) δ 154.7, 135.4, 132.8, 129.8, 128.9, 127.7, 126.5, 94.2, 26.3, 19.3, 17.5.

IR ($\tilde{\nu}_{max}$, cm^{-1}) 3072 (w), 3050 (w), 2960 (w), 2932 (w), 2858 (w), 1653 (w), 1591 (m), 1472 (w), 1429 (w), 1364 (w), 1314 (m), 1187 (w), 1112 (m), 1022 (m), 960 (m), 823 (m), 737 (m), 702 (s).

HRMS (APCI/QTOF) m/z: $[M + H]^+$ Calcd for $C_{21}H_{27}OSi^+$ 323.1826; Found 323.1815

((3-Benzylbuta-1,3-dien-2-yl)oxy)(tert-butyl)diphenylsilane (2g)

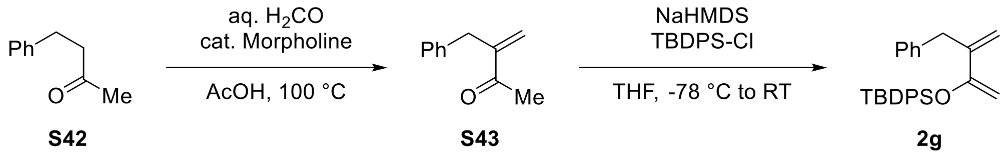

Following a reported procedure,[18] a 25 mL, round-bottomed vial was charged with 4-phenyl-2-butanone (**S42**) (2.4 mL, 16 mmol, 1.0 equiv) and acetic acid (14.4 mL). Aq. formaldehyde (37% v/v; 3.8 mL, 52 mmol, 3.2 equiv.) was then added, followed by a catalytic amount of morpholine (5 drops). The vial was sealed with a PTFE septum, and the colorless mixture was heated to 100 °C. It was then stirred at this temperature for 20 hours, becoming yellow. After this time, it was

allowed to cool down to room temperature, and subsequently neutralized by careful addition of aq. NaOH (0.1 M). The aqueous layer was extracted with EtOAc (4 x 40 mL). The combined organic layers were washed with sat. aq. $NaHCO_3$ (30 mL), and brine, dried over Na_2SO_4, filtered, and concentrated under vacuum. The resulting orange crude oil was submitted to column chromatography (Biotage flash chromatographer, SiO_2; EtOAc in pentane, 0 to 30%) to obtain 3-benzylbut-3-en-2-one (**S42**) (0.46 g, 2.8 mmol, 18% yield) as a pale yellow oil.

^{1}H NMR (400 MHz, Chloroform-*d*) δ 7.29 (t, *J* = 7.3 Hz, 2H, Ph*H*), 7.21 (dd, *J* = 7.7, 1.8 Hz, 1H, Ph*H*), 7.19–7.14 (m, 2H, Ph*H*), 6.09 (s, 1H, C=CH_2), 5.64 (s, 1H, C=CH_2), 3.59 (s, 2H, CH_2Ph), 2.34 (s, 3H, CH_3).
^{1}H-NMR signals correspond to the ones reported in the literature.[18]

Following a reported procedure,[11] in a 25 mL, two-necked, round-bottomed flask, NaHMDS (1.0 M in THF; 3.6 mL, 3.6 mmol, 1.3 equiv.) was diluted with THF (dry; 5.6 mL). The yellow solution was cooled to -78 °C (dry ice - acetone bath). 3-Benzylbut-3-en-2-one (**S43**) (0.45 g, 2.8 mmol, 1.0 equiv.) was added as a solution in THF (1.5 mL), drop-wise. The resulting orange solution was stirred at -78 °C for 40 minutes. TBDPS-Cl (0.85 mL, 3.1 mmol, 1.1 equiv.) was then added drop-wise at the same temperature. The mixture was then stirred overnight, while allowing it to warm to room temperature and becoming orange-brown. After 44 hours, the reaction was quenched by addition of sat. aq. $NaHCO_3$ (15 mL). The aqueous layer was extracted with Et_2O (3 x 25 mL). The combined organic layers were washed with brine, dried over Na_2SO_4, filtered, and concentrated under vacuum. The resulting orange crude oil was submitted to column chromatography (SiO_2; Pentane/Et_3N/Et_2O 249/1/0 to 240/1/9). ((3-Benzylbuta-1,3-dien-2-yl)oxy)(tert-butyl)diphenylsilane (**2g**) (0.622 g, 1.56 mmol, 56% yield) was obtained as a colorless, viscous oil.

^{1}H NMR (400 MHz, Chloroform-*d*) δ 7.71 (dt, *J* = 8.0, 1.2 Hz, 6H, Ph*H*), 7.45 – 7.39 (m, 2H, Ph*H*), 7.39 – 7.33 (m, 4H, Ph*H*), 7.29 (d, *J* = 8.4 Hz, 1H, Ph*H*), 7.21 (d, *J* = 6.5 Hz, 2H, Ph*H*), 5.92 (s, 1H, C=CH_2), 5.01 (s, 1H, C=CH_2), 4.33 (s, 1H, C=CH_2), 3.95 (s, 1H, C=CH_2), 3.58 (s, 2H, CH_2Ph), 1.05 (d, *J* = 0.9 Hz, 9H, C(CH_3)$_3$ in TBDPS).
^{1}H-NMR signals correspond to the ones reported in the literature.[19]

***tert*-Butyl((3-methylenehept-1-en-2-yl)oxy)diphenylsilane (2h)**

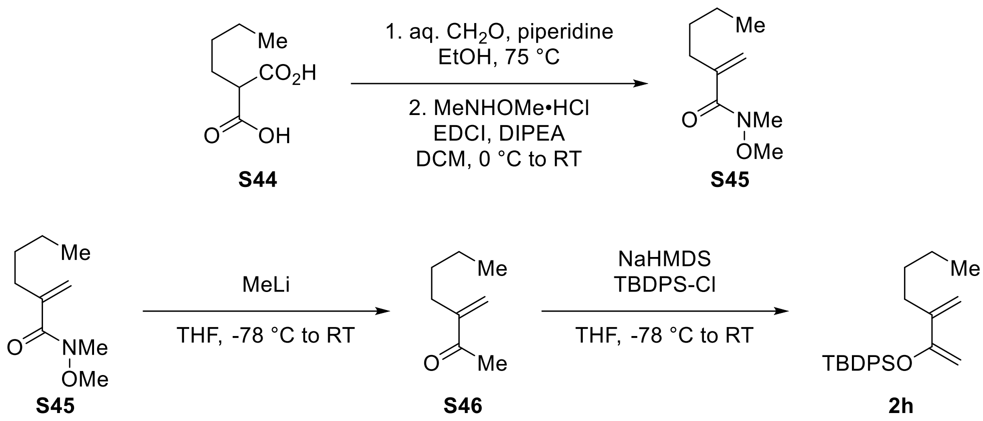

Following a modified version of a reported procedure,[20] a 100 mL, round-bottomed flask, equipped with an air condenser (Findenser©), butylmalonic acid (**S44**) (2.4 g, 15 mmol, 1.0 equiv.) was dissolved in ethanol (28 mL). Piperidine (1.8 mL, 18 mmol, 1.2 equiv.) and aq. formaldehyde (37% w/w; 5.6 mL, 75 mmol, 5 equiv.) were added. The resulting turbid mixture was then heated to 75 °C under stirring. It became a white suspension, that gradually turned back to clear by heating. After stirring at 75 °C for 16 hours, it looked like a clear, pale yellow solution. It was then allowed to cool down to room temperature, and concentrated under reduced pressure. The resulting pale yellow, oily residue was dissolved in Et_2O (150 mL). The organic solution was washed with aq. HCl (1.0 M; 2 x 75 mL) and brine. It was then dried over Na_2SO_4, filtered, and concentrated under vacuum to give a crude, pale yellow liquid that was directly used in the following step, without further purification.

Following a modified version of a reported procedure,[21] in a 250 mL, round-bottomed, two-necked flask, the crude liquid prepared in the previous step was dissolved in DCM (dry; 75 mL), and DIPEA (7.8 mL, 45 mmol, 3.0 equiv.) was added. The solution was cooled to 0 °C (ice - water bath) prior to the addition of EDCI (3.2 g, 16 mmol, 1.1 equiv.). The resulting solution was stirred for 10 minutes. Finally, N,O-dimethylhydroxylamine hydrochloride (1.6 g, 16 mmol, 1.1 equiv.) was also added in one portion. The mixture was stirred overnight, while allowing it to warm to room temperature. The organic solution was diluted with additional DCM (75 mL) and then washed with sat. aq. $NaHCO_3$ (70 mL). The aqueous layer was back extracted with DCM (2 x 70 mL). The combined organic layers were washed with brine, dried over Na_2SO_4, filtered and concentrated under vacuum. The resulting pale yellow oil was submitted to column chromatography (SiO_2; pentane/Et_2O 4/1 to 3/2). The collected fractions were washed with aq. HCl (1.0 M; 2 x 50 mL), brine, dried over Na_2SO_4, filtered and concentrated to provide N-methoxy-N-methyl-2-methylenehexanamide (**S45**) (0.95 g, 5.5 mmol, 37% yield) as a colorless liquid.

^{1}H NMR (400 MHz, Chloroform-*d*) δ 5.26 (d, *J* = 1.0 Hz, 1H, C=C*H*$_2$), 5.20 (d, *J* = 1.2 Hz, 1H, C=C*H*$_2$), 3.65 (s, 3H, C*H*$_3$), 3.24 (s, 3H, C*H*$_3$), 2.36–2.27 (m, 2H, C*H*$_2$), 1.44 (dddd, *J* = 12.1, 8.2, 5.2, 1.1 Hz, 2H, C*H*$_2$), 1.40–1.30 (m, 2H, C*H*$_2$), 0.91 (t, *J* = 7.2 Hz, 3H, C*H*$_3$).

In a 100 mL, two-necked, round-bottomed flask, methyl lithium (1.6 M solution in Et_2O; 3.8 mL, 6.1 mmol, 1.1 equiv.) was diluted in Et_2O (dry; 29 mL). The solution was chilled to -78 °C (dry ice - acetone bath). N-methoxy-N-methyl-2-methylenehexanamide (**S45**) (0.95 g, 5.5 mmol, 1.0 equiv.) was added drop-wise under stirring. The resulting pale yellow solution was stirred at -78 °C for 30 minutes, and then warmed to room temperature. After 1 hour at this temperature, TLC analysis (pentane/EtOAc 96/4) showed the complete conversion of the Weinreb amide. The reaction was quenched by addition of sat. aq. $NaHCO_3$ (30 mL). The aqueous layer was separated and extracted with Et_2O (3 x 30 mL). The combined organic layers were washed with brine, dried over Na_2SO_4, filtered, and concentrated under vacuum. Crude 3-methyleneheptan-2-one (**S46**) (0.674 g, 5.34 mmol, 96% yield) was obtained as a colorless oil, which was used directly in the following step, without further purification.

Following a reported procedure,[11] in a 25 mL, two-necked, round-bottomed flask, NaHMDS (1.0 M in THF; 6.4 mL, 6.4 mmol, 1.2 equiv.) was diluted with THF (dry; 10.6 mL). The yellow solution was cooled to -78 °C (dry ice - acetone bath). Crude 3-methyleneheptan-2-one (**S46**) from the previous step (0.67 g, 5.3 mmol, 1.0 equiv.) was added as a solution in THF (1.0 mL), drop-wise. The resulting orange solution was stirred at -78 °C for 40 minutes. TBDPS-Cl (1.4 mL, 5.3 mmol, 1.1 equiv.) was then added drop-wise at the same temperature. The mixture was then stirred overnight, while allowing it to warm to room temperature and becoming orange-brown. After 17 hours, the reaction was quenched by addition of sat. aq. $NaHCO_3$ (15 mL). The aqueous layer was extracted with Et_2O (3 x 25 mL). The combined organic layers were washed with brine, dried over Na_2SO_4, filtered, and concentrated under vacuum. The resulting orange crude oil was submitted to column chromatography (SiO_2; Pentane/Et_3N 249/1). *tert*-Butyl((3-methylenehept-1-en-2-yl)oxy)diphenylsilane (**2h**) (0.66 g, 1.8 mmol, 34% yield) was obtained as a colorless, viscous oil, which became a colorless solid on standing.

R_f (pentane) 0.70.
^{1}H NMR (400 MHz, Chloroform-*d*) δ 7.74 (dd, *J* = 7.9, 1.6 Hz, 4H, Ph*H* in TBDPS), 7.48–7.32 (m, 6H, Ph*H* in TBDPS), 5.75 (d, *J* = 2.2 Hz, 1H, C=C*H₂*), 5.05 (s, 1H, C=C*H₂*), 4.34 (d, *J* = 1.9 Hz, 1H, C=C*H₂*), 3.94 (s, 1H, C=C*H₂*), 2.26–2.13 (m, 2H, C*H₂*), 1.53–1.40 (m, 2H, C*H₂*), 1.34 (dq, *J* = 14.3, 7.2 Hz, 2H, C*H₂*), 1.05 (s, 9H, C(C*H₃*)₃ in TBDPS), 0.91 (t, *J* = 7.3 Hz, 3H, C*H₃*).
^{13}C NMR (101 MHz, Chloroform-*d*) δ 155.4, 144.5, 135.5, 132.8, 129.7, 127.6, 112.4, 93.4, 32.6, 31.0, 26.6, 22.7, 19.5, 14.0.
IR ($\tilde{\nu}_{max}$, cm^{-1}) 3662 (w), 3072 (w), 3054 (w), 2959 (m), 2932 (m), 2896 (m), 2863 (m), 1627 (w), 1588 (m), 1469 (m), 1428 (m), 1389 (m), 1339 (w), 1310 (w), 1256 (w), 1216 (m), 1173 (m), 1110 (m), 1079 (m), 1053 (m), 1017 (m), 938 (w), 903 (m), 823 (m), 793 (w), 742 (m), 704 (s)
HRMS (APCI/QTOF) m/z: $[M + H]^+$ Calcd for $C_{24}H_{33}OSi^+$ 365.2295; Found 365.2295.

***tert*-Butyl((3-cyclohexylbuta-1,3-dien-2-yl)oxy)diphenylsilane (2i)**

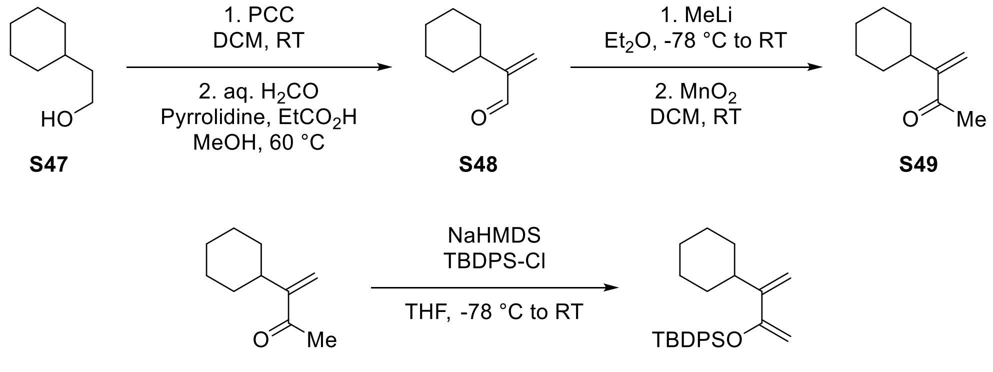

A 1 L round-bottomed flask was charged with 2-cycloheylethanol (**S47**) (2.1 mL, 15 mmol, 1.0 equiv.) and DCM (390 mL). PCC (4.9 g, 23 mmol, 1.5 equiv.) was added in one portion to the colorless solution, which immediately turned to orange and then converted into a brown suspension. The latter was stirred at room temperature for 2.5 hours. After this time, TLC analysis (pentane/EtOAc 8/2) showed the complete conversion of the starting alcohol. Et_2O (320 mL) was added, and the mixture was stirred for another hour. SiO_2 was also added, the suspension was stirred for 10 minutes, and then it was filtered through a pad of celite, which was afterwards washed with Et_2O (100 mL). The brown filtrate was concentrated under reduced pressure. The resulting brown-black oil was eluted through a short column (SiO_2) using Et_2O. Upon removal of the solvent by rotatory evaporation, the intermediate aldehyde was obtained as a pale yellow oil and directly used in the following step without further purification.

Following a reported procedure,[22] in a 25 mL, round bottomed vial, the crude aldehyde obtained in the previous step was diluted in MeOH (11 mL), and aq. formaldehyde (37% w/w; 1.4 mL, 18 mmol, 1.2 equiv.) was added. Upon the addition of pyrrolidine (0.25 mL, 3.0 mmol, 0.20 equiv.) and of propionic acid (0.22 mL, 3.0 mmol, 0.2 equiv.), the vial was sealed with a PTFE septum, and the mixture was stirred at 60 °C for 17 hours. The resulting yellow solution was allowed to cool down to room temperature and diluted with water (100 mL). The aqueous layer was extracted with Et_2O (5 x 50 mL). The combined organic layers were washed with brine, dried over Na_2SO_4, filtered, and concentrated under vacuum. The resulting pale yellow, crude oil was submitted to column chromatography (SiO_2; pentane/Et_2O 24/1 to 8/1) to provide 2-cyclohexylprop-2-enal (**S48**) (0.77 g, 5.6 mmol, 37% yield over two steps) as a colorless liquid.

In a 50 mL, two-necked, round-bottomed flask, 2-cyclohexylprop-2-enal (**S48**) (0.77 g, 5.60 mmol, 1.0 equiv.) was dissolved in Et_2O (8.0 mL). The clear colorless solution was chilled to -78 °C (dry ice - acetone bath). MeLi (1.6 M in Et_2O; 3.7 mL, 5.9 mmol, 1.05 equiv.) was then added drop-wise. The resulting mixture was stirred at -78 °C and then at room-temperature for another 2 hours. At this point, TLC analysis (pentane/EtOAc 9/1) showed complete conversion and the clean formation of a new, more polar product. The reaction was then quenched by cautions addition of $NaHCO_3$ (8.0 mL; release of gas!). The aqueous layer was separated and extracted

with Et_2O (4 x 10 mL). The combined organic layers were washed with brine, dried over Na_2SO_4, filtered, and concentrated under vacuum to provide a pale yellow crude oil, which was used directly in the following step without further purification.

Inside a 100 mL, single-necked, round bottomed flask, the crude oil obtained in the previous step was dissolved in DCM (8.0 mL). Manganese oxide (4.9 g, 56 mmol, 10 equiv.) was added, and the resulted black suspension was vigorously stirred at room temperature for 10 hours. Two further portions of DCM (8.0 mL each) and MnO_2 (4.9 g, 56 mmol, 10 equiv. each) were afterwards added after 24 and 36 hours since the beginning of the reaction. After 48 hours, TLC analysis (pentane/EtOAc 4/1) showed the clean complete conversion of the intermediate alcohol into the desired methyl ketone. The solid were filtered off through a pad of celite, which was then washed with several portions of DCM (ca. 100 mL overall). The resulting filtrate was concentrated under reduced pressure to provide 3-cyclohexylbut-3-en-2-one (**S48**) (0.51 g, 3.4 mmol, 60% yield over 2 steps) as a pale yellow oil, which was found pure enough not to proceed to any further purification.

^{1}H NMR (400 MHz, Chloroform-*d*) δ 5.98 (s, 1H, C=C*H₂*), 5.69 (d, *J* = 0.8 Hz, 1H, C=C*H₂*), 2.56 (ddd, *J* = 14.4, 10.6, 2.6 Hz, 1H, C*H*OH), 1.81–1.64 (m, 6H, Cy*H* and O*H*), 1.37 (ddd, *J* = 15.8, 9.5, 3.2 Hz, 2H, Cy*H*), 1.29 (m, 1H, Cy*H*), 1.26–1.13 (m, 2H, Cy*H*), 1.06 (qd, *J* = 12.4, 2.7 Hz, 2H, Cy*H*).

Following a reported procedure,[11] in a 25 mL, two-necked, round-bottomed flask, NaHMDS (1.0 M in THF; 5.4 mL, 5.4 mmol, 1.3 equiv.) was diluted with THF (dry; 8.3 mL). The yellow solution was cooled to -78 °C (dry ice - acetone bath). 3-Cyclohexylbut-3-en-2-one (**S48**) (0.51 g, 3.4 mmol, 1.0 equiv.) was added as a solution in THF (2.0 mL), drop-wise. The resulting orange solution was stirred at -78 °C for 40 minutes. TBDPS-Cl (0.87 mL, 3.4 mmol, 1.0 equiv.) was then added drop-wise at the same temperature. The mixture was then stirred overnight, while allowing it to warm to room temperature. After 19 hours, the reaction was quenched by addition of sat. aq. $NaHCO_3$ (15 mL). The aqueous layer was extracted with Et_2O (3 x 25 mL). The combined organic layers were washed with brine, dried over Na_2SO_4, filtered, and concentrated under vacuum. The resulting orange crude oil was submitted to column chromatography (SiO_2; pentane/Et_3N 245/1) to provide *tert*-butyl((3-cyclohexylbuta-1,3-dien-2-yl)oxy)diphenylsilane (**2i**) (0.57 g, 1.4 mmol, 43% yield) as a colorless, viscous oil.

R_f (pentane) 0.67.
^{1}H NMR (400 MHz, Chloroform-*d*) δ 7.80–7.68 (m, 4H, Ph*H* in TBDPS), 7.46–7.34 (m, 6H, Ph*H* in TBDPS), 5.72 (d, *J* = 1.7 Hz, 1H, C=C*H₂*), 5.02 (s, 1H, C=C*H₂*), 4.37 (d, *J* = 1.9 Hz, 1H, C=C*H₂*), 3.94 (m, 1H, C=C*H₂*), 2.19 (t, *J* = 11.1 Hz, 1H, C*H*OSi), 1.78 (td, *J* = 31.7, 30.2, 12.0 Hz, 5H, Cy*H*), 1.35–1.14 (m, 5H, Cy*H*), 1.05 (s, 9H, C(C*H₃*)₃ in TBDPS).

^{13}C NMR (101 MHz, Chloroform-*d*) δ 155.7, 150.4, 135.5, 132.9, 129.7, 127.6, 110.1, 92.8, 39.6, 33.5, 27.1, 26.6, 26.5, 19.5.

IR ($\tilde{\nu}_{max}$, cm^{-1}) 3072 (w), 3047 (w), 3000 (w), 2931 (m), 2855 (m), 1623 (w), 1587 (m), 1469 (w), 1436 (w), 1429 (m), 1389 (w), 1322 (w), 1284 (m), 1234 (w), 1189 (m), 1160 (m), 1112 (m), 1051 (w), 1013 (m), 939 (w), 905 (w), 891 (w), 855 (w), 824 (m), 740 (m), 702 (s)

HRMS (ESI/QTOF) m/z: $[M + Ag]^+$ Calcd for $C_{26}H_{34}AgOSi^+$ 497.1424; Found 497.1411.

***tert*-Butyl((3-(4-methoxyphenyl)buta-1,3-dien-2-yl)oxy)diphenylsilane (2j)**

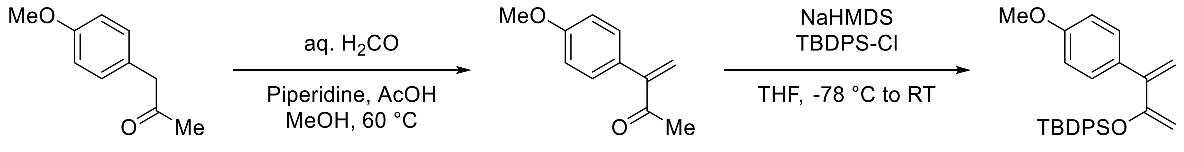

S50 **S51** **2j**

Following a reported procedure,[22] a 25 mL, round-bottomed vial was charged with 4-methoxyphenylacetone (**S50**) (1.2 mL, 8.0 mmol, 1.0 equiv.), aq. formaldehyde (37% w/w; 0.89 mL, 12 mmol, 1.5 equiv.) and methanol (5.6 mL). Piperidine (0.12 mL, 1.2 mmol 0.15 equiv.) and acetic acid (0.68 mL, 1.2 mmol, 0.1 equiv.) were added. The vial was sealed with a PTFE septum, and the yellow solution was stirred at 60 °C overnight. After 17 hours, the yellow-orange solution was allowed to cool down to room temperature, and it was diluted with water (60 mL). Brine was added in order to prevent the formation of an emulsion, which occurred upon initial treatment with Et_2O. The aqueous layer was extracted with Et_2O (5 x 40 mL). The combined organic layers were wahsed with brine, dried over Na_2SO_4, filtered, and concentrated under vacuum. The resulting orange, crude oil was submitted to column chromatography (Biotage flash chromatographer, SiO_2; EtOAc in pentane, 0 to 30%) to provide 3-(4-methoxyphenyl)but-3-en-2-one (**S51**) (0.49 g, 2.8 mmol, 35% yield) as a a pale yellow oil.

^{1}H NMR (400 MHz, Chloroform-*d*) δ 7.29 – 7.24 (m, 2H, Ar*H)*, 6.93 – 6.88 (m, 2H, Ar*H*), 6.10 (s, 1H, C=CH_2), 5.93 (s, 1H, C=CH_2), 3.83 (s, 3H, ArOCH_3), 2.45 (s, 3HCH_3).

^{1}H-NMR signals correspond to the ones reported in the literature.[23]

Following a reported procedure,[11] in a 25 mL, two-necked, round-bottomed flask, NaHMDS (1.0 M in THF; 3.9 mL, 3.9 mmol, 1.3 equiv.) was diluted with THF (dry; 6.9 mL). The yellow solution was cooled to -78 °C (dry ice - acetone bath). 3-(4-Methoxyphenyl)but-3-en-2-one (**S51**) (0.49 g, 2.8 mmol, 1.0 equiv.) was added as a solution in THF (1.5 mL), drop-wise. The resulting golden yellow solution was stirred at -78 °C for 60 minutes. TBDPS-Cl (0.76 mL, 2.9 mmol, 1.05 equiv.) was then added drop-wise at the same temperature. The mixture was then stirred overnight, while allowing it to warm to room temperature. It became orange-brown during this time. After 18 hours, the reaction was quenched by addition of sat. aq. $NaHCO_3$ (20 mL). The aqueous layer was extracted with Et_2O (3 x 25 mL). The combined organic layers were washed with brine, dried over Na_2SO_4, filtered, and concentrated under vacuum. The resulting orange crude oil was submitted

to column chromatography (SiO_2; Et_2O/pentane 5/245 to 20/230, with 0.5% Et_3N) to afford *tert*-butyl((3-(4-methoxyphenyl)buta-1,3-dien-2-yl)oxy)diphenylsilane (**2j**) (0.47 g, 3.9 mmol, 40% yield) as a pale yellow, viscous oil.

^{1}H NMR (400 MHz, Methylene Chloride-*d_2*) δ 7.78 (dd, *J* = 7.9, 1.6 Hz, 4H, Ph*H* in TBDPS), 7.51–7.36 (m, 6H, Ph*H* in TBDPS), 7.28–7.18 (m, 2H, Ar*H*), 6.88–6.80 (m, 2H, Ar*H*), 5.87 (d, *J* = 2.0 Hz, 1H, C=C*H_2*), 5.20 (s, 1H, C=C*H_2*), 4.13 (s, 1H, C=C*H_2*), 4.09 (s, 1H, C=C*H_2*), 3.79 (s, 3H, ArOC*H_3*), 1.06 (s, 9H, C(C*H_3*)$_3$ in TBDPS).
^{1}H-NMR signals correspond to the ones reported in the literature.[19]

((3-(Benzofuran-5-yl)buta-1,3-dien-2-yl)oxy)(tert-butyl)diphenylsilane (2k)

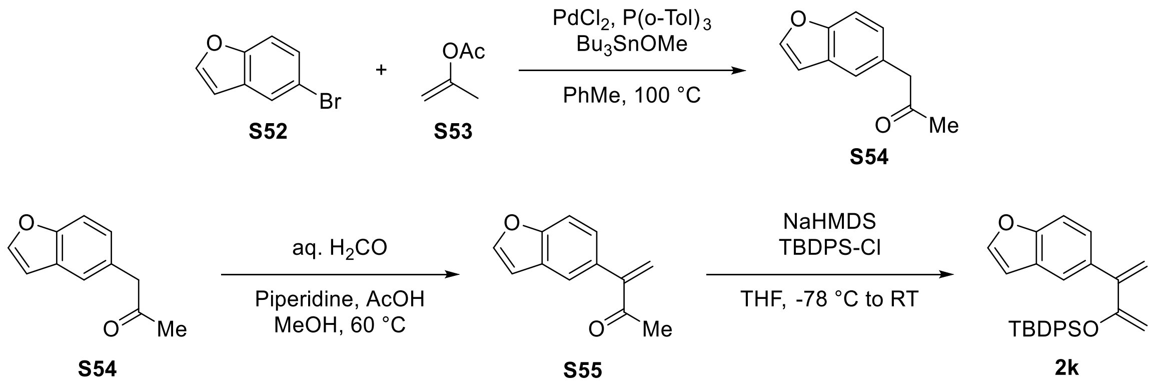

Following a reported procedure,[24] in a 100 mL, two-necked, round-bottomed flask equipped with an air condenser (Findenser©), 5-bromobenzofuran (**S52**) (1.25 mL, 10.0 mmol, 1.0 equiv.) was dissolved in toluene (dry; 40 mL). Tri(*o*-tolyl)phosphine (0.18 g, 0.60 mmol, 6 mol%), tributyltin methoxide (4.2 mL, 15 mmol, 1.5 equiv.) and isopropenyl acetate (**S53**) (1.7 mL, 15 mmol, 1.5 equiv.) were then also added, after which the mixture was sparged with nitrogen for 20 minutes. Palladium dichloride (0.12 g, 0.70 mmol, 7 mol%) was then added rapidly. The resulting dark brown mixture was subsequently stirred at 100 °C for 18 hours. The mixture was then allowed to cool down to room temperature, and it was concentrated under reduced pressure. The resulting black residue was diluted with EtOAc (up to a volume of 70 mL) and most of the solids were removed by filtration through a pad of celite (then washed with EtOAc). The organic solution was washed with water, then with a sat. aq. solution of KF (70 mL; precipitation occured), and brine. It was then treated with Na_2SO_4, and filtered through a pad of celite to give a yellow filtrate. The latter was concentrated under vacuum to provide a yellow crude oil, which was submitted to column chromatography (Biotage, 40 g SiO_2; EtOAc in pentane, 0 to 40%) to furnish 1-(benzofuran-5-yl)propan-2-one (**S54**) (1.48 g, 8.51 mmol, 85% yield) as a yellow oil.

^{1}H NMR (400 MHz, Chloroform-d) δ 7.62 (d, *J* = 2.1 Hz, 1H, Ar*H*), 7.47 (d, *J* = 8.4 Hz, 1H, Ar*H*), 7.44 (s, 1H, Ar*H*), 7.12 (dd, *J* = 8.4, 1.5 Hz, 1H, Ar*H*), 6.74 (m, 1H, Ar*H*), 3.78 (s, 2H, COC*H_2*), 2.16 (s, 3H, C*H_3*).

Following a reported procedure,[25] In a 25 mL, round-bottomed vial, 1-(benzofuran-5-yl)propan-2-one (**S54**) (0.80 g, 4.6 mmol, 1.0 equiv.) was dissolved in DMF (dry; 9.1 mL). Paraformaldehyde (0.83 g, 28 mmol, 6.0 equiv.) was added. Finally, piperidine (0.59 mL, 0.59 mmol, 13 mol%) and acetic acid (0.58 mL, 1.0 mmol, 22 mol%) were also added in this order. The vial was sealed with a PTFE septum and the mixture was stirred at 90-95 °C for 1.5 hours, darkening to orange-brown. It was then allowed to cool down to room temperature and diluted with brine (50 mL). The aqueous layer was extracted with EtOAc (4 x 40 mL). The combined organic layers were washed with brine (twice), dried over Na_2SO_4, filtered, and concentrated under vacuum. The resulting orange/brown oily residue was submitted to column chromatography (Biotage, 25 g SiO_2; EtOAc in pentane, 5 to 40%) to provide 3-(benzofuran-5-yl)but-3-en-2-one (**S55**) (0.56 g, 3.0 mmol, 65% yield) as a colorless oil.

^{1}H NMR (400 MHz, Chloroform-*d*) δ 7.63 (d, *J* = 2.2 Hz, 1H, Ar*H*), 7.55 (d, *J* = 1.7 Hz, 1H, Ar*H*), 7.48 (d, *J* = 8.5 Hz, 1H, Ar*H*), 7.23 (dd, *J* = 8.5, 1.8 Hz, 1H, Ar*H*), 6.77 (dd, *J* = 2.2, 0.8 Hz, 1H, Ar*H*), 6.19 (s, 1H, C=CH_2), 5.98 (s, 1H, C=CH_2), 2.46 (s, 3H, CH_3).

Following a reported procedure,[11] in a 25 mL, two-necked, round-bottomed flask, NaHMDS (1.0 M in THF; 3.9 mL, 3.9 mmol, 1.3 equiv.) was diluted with THF (dry; 7.4 mL). The yellow solution was cooled to -78 °C (dry ice - acetone bath). 3-(benzofuran-5-yl)but-3-en-2-one (**S55**) (0.56 g, 3.0 mmol, 1.0 equiv.) was added as a solution in THF (1.0 mL), drop-wise. The resulting golden yellow solution was stirred at -78 °C for 60 minutes. TBDPS-Cl (0.78 mL, 23 mmol, 1.0 equiv.) was then added drop-wise at the same temperature. The mixture was then stirred overnight, while allowing it to warm to room temperature, turning into an orange-brown suspension. After 18 hours, the reaction was quenched by addition of sat. aq. $NaHCO_3$ (30 mL). The aqueous layer was extracted with Et_2O (3 x 30 mL). The combined organic layers were washed with brine, dried over Na_2SO_4, filtered, and concentrated under vacuum. The resulting yellow-orange crude oil was submitted to column chromatography (SiO_2; pentane/Et_2O/NEt_3 250/0/1 to 245/5/1) to provide ((3-(benzofuran-5-yl)buta-1,3-dien-2-yl)oxy)(tert-butyl)diphenylsilane (**2k**) (0.85 g, 2.0 mmol, 67% yield) as a very viscous, colorless oil.

R_f (pentane/EtOAc 9/1) 0.95

^{1}H NMR (400 MHz, Methylene Chloride-*d*$_2$) δ = 7.84 – 7.76 (m, 4H, Ph*H* in TBDPS), 7.64 (d, *J*=2.2, 1H, Ar*H*), 7.53 (d, *J*=1.7, 1H, Ar*H*), 7.49 – 7.39 (m, 7H, Ph*H* in TBDPS and Ar*H*), 7.23 (dd, *J*=8.5, 1.8, 1H, Ar*H*), 6.77 (dd, *J*=2.2, 0.8, 1H, Ar*H*), 5.97 (d, *J*=2.0, 1H, C=CH_2), 5.26 (s, 1H, C=CH_2), 4.12 (s, 2H, C=CH_2), 1.08 (s, 9H C(CH_3)$_3$ in TBDPS).

^{13}C NMR (101 MHz, Methylene Chloride-*d*$_2$) δ 156.9, 155.0, 147.7, 146.0, 136.1, 136.0, 133.2, 130.4, 128.3, 127.7, 126.2, 122.1, 115.6, 111.0, 107.1, 98.4, 26.9, 19.9.

IR ($\tilde{\nu}_{max}$, cm^{-1}) 3071 (w), 3047 (w), 2960 (w), 2933 (w), 2895 (w), 2859 (w), 1618 (w), 1582 (m), 1537 (w), 1468 (m), 1426 (w), 1368 (w), 1346 (w), 1254 (m), 1206 (m), 1158 (m), 1132 (m), 1110 (s), 1072 (w), 1011 (m), 906 (w), 888 (w), 860 (w), 839 (w), 820 (m), 773 (m), 738 (s), 702 (s)
HRMS (ESI/QTOF) m/z: $[M + H]^+$ Calcd for $C_{28}H_{29}O_2Si^+$ 425.1931; Found 425.1927.

***tert*-Butyl((3-(4-chlorophenyl)buta-1,3-dien-2-yl)oxy)diphenylsilane (2l)**

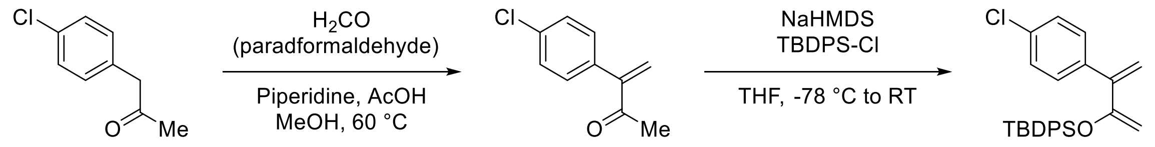

S56 **S57** **2l**

Following a reported procedure,[25] in a 25 mL, round-bottomed vial, 1-(4-chlorophenyl)propan-2-one (**S56**) (1.2 mL, 8.0 mmol, 1.0 equiv.) was dissolved in DMF (dry; 16 mL). Paraformaldehyde (1.20 g, 40.0 mmol, 5.0 equiv.) was added. Finally, piperidine (0.10 mL, 1.0 mmol, 13 mol%) and acetic acid (0.10 mL, 1.8 mmol, 22 mol%) were also added in this order. The vial was sealed with a PTFE septum and the mixture was stirred at 90-95 °C for 2 hours, darkening from pale yellow to orange. It was then allowed to cool down to room temperature and diluted with water (60 mL). The aqueous layer was extracted with EtOAc (4 x 40 mL). The combined organic layers were washed with brine, dried over Na_2SO_4, filtered, and concentrated under vacuum. The resulting dark orange/brown oily residue was submitted to column chromatography (Biotage flash chromatographer, SiO_2; EtOAc in pentane, 0 to 45%) to provide 3-(4-chlorophenyl)but-3-en-2-one (**S57**) (0.54 g, 3.0 mmol, 41% yield) as a pale yellow oil.

^{1}H NMR (400 MHz, Chloroform-*d*) δ 7.35 – 7.31 (m, 2H, Ar*H*), 7.26 – 7.23 (m, 2H, Ar*H*), 6.21 (s, 1H, C=C*H₂*), 6.01 (s, 1H, C=C*H₂*), 2.46 (s, 3H, C*H₃*).

Following a reported procedure,[11] in a 25 mL, two-necked, round-bottomed flask, NaHMDS (1.0 M in THF; 3.9 mL, 3.9 mmol, 1.3 equiv.) was diluted with THF (dry; 7.5 mL). The yellow solution was cooled to -78 °C (dry ice - acetone bath). 3-(4-Chlorophenyl)but-3-en-2-one (**S57**) (0.54 g, 3.0 mmol, 1.0 equiv.) was added as a solution in THF (1.0 mL), drop-wise. The resulting golden yellow solution was stirred at -78 °C for 60 minutes. TBDPS-Cl (0.82 mL, 3.2 mmol, 1.05 equiv.) was then added drop-wise at the same temperature. The mixture was then stirred overnight, while allowing it to warm to room temperature, turning into an orange-brown suspension. After 16 hours, the reaction was quenched by addition of sat. aq. $NaHCO_3$ (30 mL). The aqueous layer was extracted with Et_2O (3 x 25 mL). The combined organic layers were washed with brine, dried over Na_2SO_4, filtered, and concentrated under vacuum. The resulting orange crude oil was submitted to column chromatography (SiO_2; Pentane/Et_2O 24/1 to 21/3 containing Et_3N 0.5%) to provide *tert*-butyl((3-(4-chlorophenyl)buta-1,3-dien-2-yl)oxy)diphenylsilane (**2l**) (0.51 g, 1.8 mmol, 36% yield) as a viscous, colorless oil.

R_f (pentane) 0.51.

^{1}H NMR (400 MHz, Methylene Chloride-d_2) δ 7.80 – 7.74 (m, 4H, Ph*H* in TBDPS), 7.49 – 7.39 (m, 6H, Ph*H* in TBDPS), 7.30 – 7.26 (m, 2H, Ar*H*), 7.26 – 7.20 (m, 2H, Ar*H,* C=C*H$_2$*), 5.93 (d, *J* = 1.7 Hz, 1H, C=C*H$_2$*), 5.24 (s, 1H, C=C*H$_2$*), 4.11 (s, 1H, C=C*H$_2$*), 4.09 (s, 1H, C=C*H$_2$*), 1.06 (s, 9H, C(C*H$_3$*)$_3$ in TBDPS).

^{13}C NMR (101 MHz, Methylene Chloride-d_2) δ 155.6, 146.0, 139.0, 135.5, 133.1, 132.5, 130.3, 129.9, 128.0, 127.7, 115.5, 97.7, 26.3, 19.3.

IR ($\tilde{\nu}_{max}$, cm^{-1}) 3658 (w), 3069 (w), 2962 (m), 2935 (m), 2898 (m), 2859 (m), 1623 (w), 1584 (w), 1487 (w), 1472 (w), 1429 (m), 1390 (m), 1345 (w), 1258 (w), 1218 (m), 1180 (w), 1111 (m), 1093 (m), 1061 (m), 1017 (s), 910 (w), 885 (w), 836 (m), 777 (w), 742 (m), 702 (s)

HRMS (ESI/QTOF) m/z: $[M + Ag]^+$ Calcd for $C_{26}H_{27}AgClOSi^+$ 525.0565 and 527.0557; Found 525.0573 and 527.0572.

tert-Butyl((3-(naphthalen-1-yl)buta-1,3-dien-2-yl)oxy)diphenylsilane (2m)

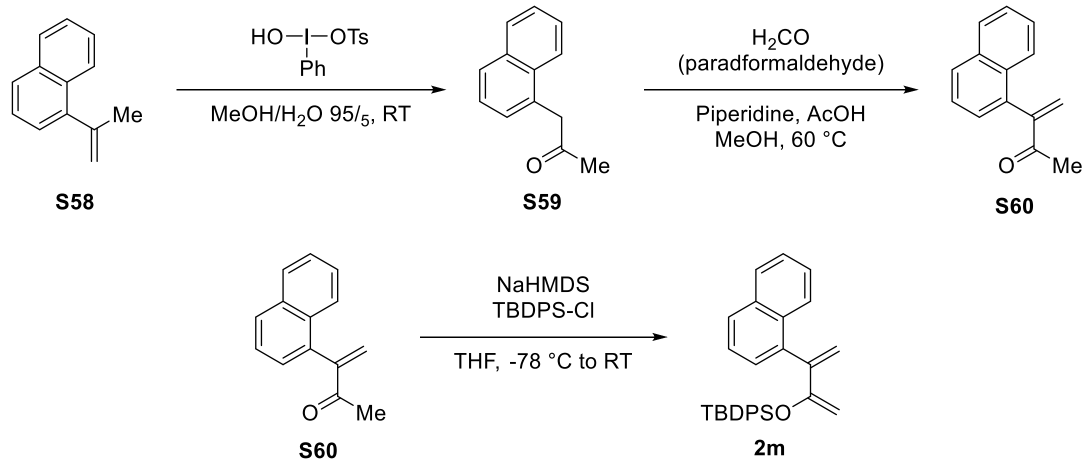

Following a reported procedure,[26] in a 100 mL, single-necked flask, 1-(prop-1-en-2-yl)naphthalene (**S58**) (1.4 g, 8.5 mmol, 1.1 equiv.) was dissolved in a 95/5 mixture of methanol (33 mL) and water (1.5 mL). Hydroxy(phenyl)-λ_3-iodaneyl 4-methylbenzenesulfonate (Koser's reagent; 3.1 g, 7. 8 mmol, 1.0 equiv.) was added, getting rapidly dissolved as the solution turned from colorless to pale yellow. Mild heat release was observed (condensation on the walls of the flask). The solution was stirred at room temperature for 20 minutes, during which time the solution turned back to colorless. It was then concentrated under reduced pressure. The resulting pale yellow pasty residue was partitioned between water (40 mL) and DCM (40 mL). The aqueous layer was separated and extracted with DCM (3 x 30 mL). The combined organic layers were washed with brine, dried over Na_2SO_4, filtered, and concentrated under vacuum. The resulting yellow crude oil was submitted to column chromatography (Biotage flash chromatographer, SiO_2; EtOAc in pentane, 2 to 35%) to provide 1-(naphthalen-1-yl)propan-2-one (**S59**) (1.17 g, 6.37 mmol, 82% yield) as a pale yellow oil.

^{1}H NMR (400 MHz, Chloroform-*d*) δ 7.91 (ddd, *J* = 6.5, 4.3, 1.9 Hz, 2H, Ar*H*), 7.84 (d, *J* = 8.2 Hz, 1H, Ar*H*), 7.58 – 7.50 (m, 2H, Ar*H*), 7.48 (m, 1H, Ar*H*), 7.42 (d, *J* = 6.5 Hz, 1H, Ar*H*), 4.15 (s, 2H, ArCH_2), 2.14 (s, 3H, CH_3).
^{1}H-NMR signals correspond to the ones reported in the literature.[27]

Following a reported procedure,[25] in a 25 mL, round-bottomed vial, 1-(naphthalen-1-yl)propan-2-one (**S59**) (1.17 g, 6.35 mmol, 1.0 equiv.) was dissolved in DMF (dry; 12.7 mL). Paraformaldehyde (0.953 g, 31.7 mmol, 5.0 equiv.) was added. Finally, piperidine (0.80 mL, 0.82 mmol, 13 mol%) and acetic acid (0.80 mL, 1.4 mmol, 22 mol%) were also added in this order. The vial was sealed with a PTFE septum and the mixture was stirred at 90-95 °C for 2 hours, darkening to orange-yellow. It was then allowed to cool down to room temperature and diluted with water (60 mL). The aqueous layer was extracted with Et_2O (4 x 40 mL). The combined organic layers were washed with brine (twice), dried over Na_2SO_4, filtered, and concentrated under vacuum. The resulting dark orange/brown oily residue was submitted to column chromatography (Biotage flash chromatographer, SiO_2; EtOAc in pentane, 0 to 45%) to provide 3-(naphthalen-1-yl)but-3-en-2-one (**S60**) (0.77 g, 3.9 mmol, 61% yield) as a colorless oil.

^{1}H NMR (400 MHz, Chloroform-*d*) δ 7.92 – 7.83 (m, 2H, Ar*H*), 7.65 (m, 1H, Ar*H*), 7.54 – 7.42 (m, 2H, Ar*H*), 7.33 (dd, *J* = 7.0, 1.1 Hz, 1H, Ar*H*), 6.52 (d, *J* = 1.3 Hz, 1H, C=CH_2), 5.86 (d, *J* = 1.3 Hz, 1H, C=CH_2), 2.29 (s, 3H, CH_3).

Following a reported procedure,[11] in a 100 mL, two-necked, round-bottomed flask, NaHMDS (1.0 M in THF; 5.1 mL, 5.1 mmol, 1.3 equiv.) was diluted with THF (dry; 9.6 mL). The yellow solution was cooled to -78 °C (dry ice - acetone bath). 3-(Naphthalen-1-yl)but-3-en-2-one (**S60**) (0.765 g, 3.90 mmol, 1.0 equiv.) was added as a solution in THF (1.0 mL), drop-wise. The resulting golden yellow solution was stirred at -78 °C for 60 minutes. TBDPS-Cl (1.1 mL, 4.1 mmol, 1.05 equiv.) was then added drop-wise at the same temperature. The mixture was then stirred overnight, while allowing it to warm to room temperature, turning into an orange-brown suspension. After 16 hours, the reaction was quenched by addition of sat. aq. $NaHCO_3$ (30 mL). The aqueous layer was extracted with Et_2O (3 x 35 mL). The combined organic layers were washed with brine, dried over Na_2SO_4, filtered, and concentrated under vacuum. The resulting orange crude oil was submitted to column chromatography (SiO_2; Pentane/Et_2O/Et_3N 249/0/1 to 245/4/1) to provide *tert*-butyl((3-(naphthalen-1-yl)buta-1,3-dien-2-yl)oxy)diphenylsilane (**2m**) (0.98 g, 2.3 mmol, 58% yield) as a colorless solid.

M.P. 68.0-70.7 °C
R_f (pentane) 0.41.
^{1}H NMR (400 MHz, Methylene Chloride-d_2) δ 7.87–7.81 (m, 6H, Ar*H* and Ph*H* in TBDPS), 7.79 (d, *J* = 8.3 Hz, 1H, Ar*H*), 7.50–7.38 (m, 9H, Ar*H* and Ph*H* in TBDPS), 7.34 (dd, *J* = 7.0, 1.2 Hz,

1H, Ar*H*), 6.32 (d, *J* = 2.2 Hz, 1H, C=C*H*2), 5.30 (t, *J* = 1.5 Hz, 1H, C=C*H*2), 4.01 (s, 1H, C=C*H*2), 3.73 (m, 1H, C=C*H*2), 1.14 (s, 9H, C(C*H*3)3 in TBDPS).

^{13}C NMR (101 MHz, Methylene Chloride-*d*2) δ 156.4, 145.7, 138.9, 136.2, 134.0, 133.1, 132.7, 130.5, 128.5, 128.3, 128.2, 127.5, 126.7, 126.3, 126.2, 125.7, 117.2, 98.7, 27.0, 19.9.

IR ($\tilde{\nu}_{max}$, cm^{-1}) 3070 (w), 3048 (w), 2958 (m), 2932 (m), 2891 (m), 2858 (m), 1818 (w), 1617 (w), 1584 (m), 1505 (w), 1469 (w), 1428 (w), 1389 (w), 1371 (w), 1335 (w), 1260 (w), 1230 (m), 1177 (m), 1119 (m), 1111 (m), 1062 (w), 1016 (m), 937 (w), 914 (w), 892 (w), 827 (m), 802 (m), 780 (m), 739 (m), 702 (s)

HRMS (ESI/QTOF) m/z: $[M + H]^+$ Calcd for $C_{30}H_{31}OSi^+$ 435.2139; Found 435.2145.

2. Discovery and Optimization of the annulation reaction

2.1 Discovery of the annulation reaction

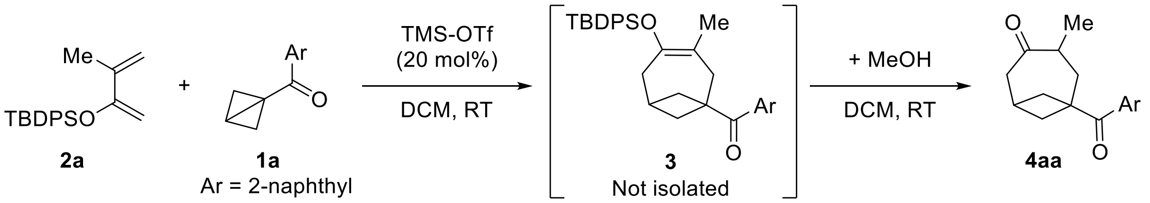

A 10 mL, round-bottomed vial was charged with bicyclo[1.1.0]butan-1-yl(naphthalen-2-yl)methanone (**1a**) (0.031 g, 0.15 mmol, 1.0 equiv.) and *tert*-butyl((3-methylbuta-1,3-dien-2-yl)oxy)diphenylsilane (**2a**) (0.106 g, 0.330 mmol, 2.2 equiv.). The vial was sealed with a PTFE septum, evacuated and back-filled with nitrogen (3 times). DCM (dry; 1.0 mL) was added by syringe, giving a clear, colorless solution. A solution of TMSOTf in DCM (0.5 mL of a solution prepared by dissolving 0.044 mL TMSOTf in 4.0 mL DCM, corresponding to 0.0054 mL TMSOTf, 0.030 mmol, 20 mol%) was added at room temperature: the reaction mixture darkened from colorless to yellow during the addition, and then reverted to colorless. The mixture was stirred at room temperature for 18 hours. After this time, TLC analysis of the mixture showed the complete consumption of starting material **2a** and the formation of a new, less polar product (**3**, R_f (pentane/EtOAc 9/1) 0.90). The reaction was quenched by addition of MeOH (0.5 mL), which resulted in the colorless mixture to become immediately orange. The mixture was left to stand for 2 hours, during which time it turned back to pale yellow. At this point, TLC analysis showed the complete consumption of **3** and its conversion into more polar compound **4aa** (R_f (pentane/EtOAc 9/1) 0.30). The mixture was then concentrated under reduced pressured. The resulting wet, brown crude oil was submitted to column chromatography (Biotage flash chromatographer, SiO_2; EtOAc in pentane, 0 to 50% - the elution of the product occurred when EtOAc was ca 20%) to afford 6-(2-naphthoyl)-4-methylbicyclo[4.1.1]octan-3-one (**4aa**) (0.031 g, 0.11 mmol, 71% yield) as an off-white solid.

Characterization of 6-(2-naphthoyl)-4-methylbicyclo[4.1.1]octan-3-one (4aa)

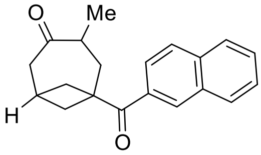

4aa

M.P. 136.2-139.6 °C.

R_f (pentane/EtOAc 9/1) 0.30.

^{1}H NMR (400 MHz, Chloroform-*d*) δ 8.27 (s, 1H, Ar*H*), 7.94 (d, *J* = 7.8 Hz, 1H, Ar*H*), 7.91 – 7.80 (m, 3H, Ar*H*), 7.65 – 7.50 (m, 2H, Ar*H*), 3.30 (dp, *J* = 12.4, 6.2 Hz, 1H, C*H*CH_3), 2.97 (dd, *J* = 12.3, 8.9 Hz, 1H, C*H_2*), 2.90 (dd, *J* = 12.1, 8.3 Hz, 1H, C*H_2*), 2.73 (d, *J* = 5.1 Hz, 2H, C*H_2*), 2.61-2.49 (m, 2H, C*H* and C*H_2*), 2.17 (m, 1H, C*H_2*), 1.84 (m, 1H, C*H_2*), 1.79 (dd, *J* = 13.7, 11.9 Hz, 1H, C*H_2*), 1.14 (d, *J* = 6.4 Hz, 3H, C*H_3*).

^{13}C NMR (101 MHz, Chloroform-*d*) δ 213.4, 203.6, 135.4, 132.4, 131.4, 130.6, 129.6, 128.6, 128.5, 127.8, 126.8, 124.8, 52.1, 48.7, 43.2, 41.5, 36.4, 33.5, 25.1, 15.5.

IR ($\tilde{v}_{max}$, cm^{-1}) 3061 (w), 2963 (m), 2936 (m), 2868 (m), 1704 (s), 1668 (s), 1626 (m), 1594 (w), 1462 (m), 1443 (m), 1374 (m), 1353 (m), 1278 (s), 1234 (m), 1216 (m), 1195 (m), 1153 (m), 1122 (m), 1051 (w), 1019 (w), 981 (w), 885 (w), 867 (m), 826 (m), 803 (m), 777 (s), 762 (s), 734 (m).

HRMS (APCI/QTOF) m/z: $[M + H]^+$ Calcd for $C_{20}H_{21}O_2^+$ 293.1536; Found 293.1530.

2.2 Optimization of the annulation reaction

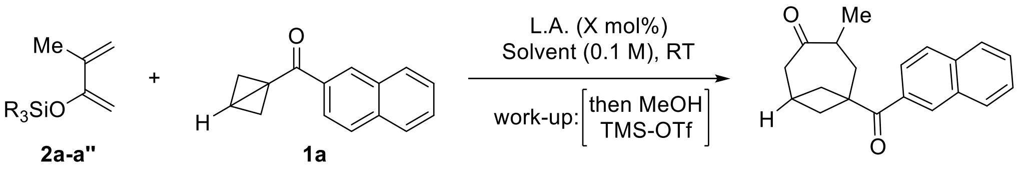

General Procedure (GP1, Table 1): Inside a glove box, a 10 mL, round-bottomed vial was charged with the catalyst, the dienol sylyl ether **2a-a''**, and bicyclo[1.1.0]butan-1-yl(naphthalen-2-yl)methanone (**1a**) (0.031 g, 0.15 mmol, 1.0 equiv). The vial was sealed with a PTFE septum and taken out of the glove-box. DCM (dry; 1.5 mL) was added by syringe, giving a clear, colorless to pale yellow solution. The mixture was stirred at room temperature overnight. After 16-20 hours, TLC analysis (pentane/EtOAc 9/1) of the mixture was used to verify that the full conversion of the starting material had been achieved, with formation of intermediate silyl enol ether **3**. At this point, the vial was uncapped, and MeOH (ca. 1.5 mL) was added followed by TMS-OTf (0.10 mL, 0.60 mmol, 4.0 equiv.). The resulting turbid mixture was stirred at room temperature for 4 hours to ensure the complete hydrolysis of **3** into **1aa**. The mixture was then concentrated under reduced pressured. The resulting wet, brown crude oil was submitted to column chromatography (Biotage flash chromatographer, SiO_2; EtOAc in pentane, 0 to 50%) to afford 6-(2-naphthoyl)-4-methylbicyclo[4.1.1]octan-3-one (**4aa**) as an off-white solid.

Exceptions to the GP1:

- TMS-OTf and BF_3●OEt_2 were added by syringe as solutions in DCM to mixtures of the aforementioned amounts of **1a** and **2a** in DCM (1.0 mL). Respectively:
  - TMS-OTf: 0.5 mL of a solution prepared by dissolving 0.044 mL TMSOTf in 4.0 mL DCM, corresponding to 0.0054 mL TMSOTf, 0.030 mmol, 20 mol%

- BF_3●OEt_2: 0.5 mL of a solution prepared by dissolving 0.031 mL BF_3●OEt_2 in 4.0 mL DCM, corresponding to 0.0038 mL TMSOTf, 0.030 mmol, 20 mol%).

- **2a’** and **2a’’** being oils, the corresponding required amounts were added as solutions in DCM (1.0 mL).
- In all cases, variable amounts of a non-identified impurity resulting from the used dienol silyl ether were formed. This impurity was systematically co-eluted with enol silyl ether intermediate **3** making all attempts to isolate the latter in higher purity than 90% unsuccessful.

  N.B.: For few entries of Table 1, yields refer to **3**. In the corresponding experiments, according to TLC analysis, (deceivingly) lower amounts of the aforementioned impurity were generated. In such cases, once the annulative step was found complete, the reaction mixture was concentrated under reduced pressure, without proceeding to the hydrolytic quench with TMS-OTf/MeOH. The resulting crude material was then submitted to column chromatography (Biotage flash chromatographer, SiO_2; Et_2O in pentane, slow gradient, 0 to 25%) and yield was determined based on the isolation of **3**. For none of these entries, purity of **3** exceeded the range of 90-95%.

Table 1: Optimization of the annulation reaction between BCB ketone **1a** and dienol silyl ether **2a** (**2a'**, **2a''**)

Entry	R_3Si	n equiv. YY	L.A. (x mol%)	Solvent	MeOH/TMS-OTf?	Isolated yield[a]
1	TBDPS (**2a**)	2.2	TMS-OTf (20)	DCM	Yes	70%
2	TBS (**2a'**)	2.2	TMS-OTf (20)	DCM	Yes	33%
3	TIPS (**2a''**)	2.2	TMS-OTf (20)	DCM	Yes	52%
4	TBDPS (**2a**)	2.2	BF_3●OEt_2 (20)	DCM	No	**3**,< 63%
5	TBDPS (**2a**)	2.2	$Cu(ClO_4)_2$ (20)	DCM	Yes	78%
6	TBDPS (**2a**)	2.2	$Cu(ClO_4)_2$ (5)	DCM	Yes	77%
7	TBDPS (**2a**)	2.2	$Cu(BF_4)_2$ (20)	DCM	Yes	64%
8	TBDPS (**2a**)	2.2	$Cu(OTf)_2$ (20)	DCM	Yes	55%
9	TBDPS (**2a**)	2.2	$In(OTf)_3$ (20)	DCM	Yes	73%
10	TBDPS (**2a**)	2.2	$Bi(OTf)_3$ (20)	DCM	Yes	70%
11	TBDPS (**2a**)	2.2	$Yb(OTf)_3$ (10)	DCM	No	**3**,< 64%[b]
12	TBDPS (**2a**)	2.2	$Dy(OTf)_3$ (20)	DCM	No	**3**,< 58%[b]
13	TBDPS (**2a**)	2.2	$Ga(OTf)_3$ (20)	DCM	Yes	83%
14	TIPS (**2a''**)	2.2	$Ga(OTf)_3$ (20)	DCM	Yes	58%
15	TBDPS (**2a**)	2.2	$Ga(OTf)_3$ (10)	DCM	Yes	81%
16	TBDPS (**2a**)	1.2	$Ga(OTf)_3$ (10)	DCM	Yes	61%
17	TBDPS (**2a**)	2.2	$Ga(OTf)_3$ (5)	DCM	Yes	62%
18	TBDPS (**2a**)	2.2	$Al(OTf)_3$ (20)	DCM	Yes	80%
19	TBDPS (**2a**)	2.2	$Al(OTf)_3$ (10)	DCM	Yes	84%
20	TBDPS (**2a**)	2.2	$Al(OTf)_3$ (5)	DCM	Yes	90%
22	TBDPS (**2a**)	2.2	$Al(OTf)_3$ (10)	Et_2O	Yes	57%
23	TBDPS (**2a**)	2.2	$Al(OTf)_3$ (10)	$CHCl_3$	Yes	75%
24	TBDPS (**2a**)	2.2	$Al(OTf)_3$ (5)	DCE	Yes	89%

General conditions: 1a (0.15 mmol,1.0 equiv.), **2a-a''** (0.18 to 0.33 mmol, 1.2 to 2.2 equiv.), Lewis acid catalyst (0.0075 to 0.030 mmol, 5 to 20 mol%), solvent (1.5 mL), room temperature, 16-18 hours; then MeOH (1.5 mL) and TMS-OTf (0.10 mL, 6.0 mmol, 4.0 equiv.), room temperature, 4 hours. a) Referred to ketone product **4aa**, unless otherwise indicated. b) <90% purity.

Characterization of (4-((tert-butyldiphenylsilyl)oxy)-3-methylbicyclo[4.1.1]oct-3-en-1-yl)(naphthalen-2-yl)methanoneenol (enol silyl ether intermediate 3)

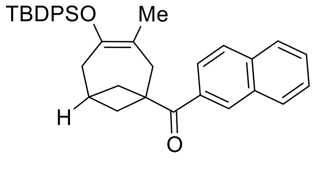

3

Ca. 90% pure. Viscous, pale yellow oil.

R_f (pentane/EtOAc 9/1) 0.90.

^{1}H NMR (400 MHz, DMSO-d_6; the signal corresponding to two aliphatic H is mostly overlapped with the solvent peak) δ 8.38 (s, 1H, Ar*H*), 8.14 (d, *J* = 7.9 Hz, 1H, Ar*H*), 7.99 (t, *J* = 9.0 Hz, 2H, Ar*H*), 7.84 (d, *J* = 8.5 Hz, 1H, Ar*H*), 7.76 – 7.71 (m, 4H, Ph*H* in TBDPS), 7.64 (dt, *J* = 18.5, 6.8 Hz, 2H, Ar*H*), 8.39 – 8.34 (m, 6H, Ph*H* in TBDPS), 2.44 (d, *J* = 8.8 Hz, 2H, C*H₂*), 2.19 (br s, 2H, C*H₂*), 2.08 (s, 1H, C*H₂*), 1.84 (d, *J* = 10.4 Hz, 2H, C*H₂*), 1.75 (s, 3H, C*H₃*), 1.05 (s, 8H, C(C*H₃*)₃ in TBDPS).

^{13}C NMR (101 MHz, DMSO-d_6; the signal corresponding to one aliphatic carbon was not resolved) δ 203.9, 142.8, 135.4, 135.3, 134.2, 132.5, 131.5, 130.9, 130.4, 130.2, 129.1, 128.8, 128.3, 128.0, 127.4, 125.0, 111.1, 50.6, 42.4, 34.0, 27.2, 25.4, 19.5, 19.3.

HRMS (ESI/QTOF) m/z: [M + H]$^+$ Calcd for $C_{36}H_{39}O_2Si^+$ 531.2714; Found 531.2718.

2.3 Adjustment of the silyl-deprotecting work-up.

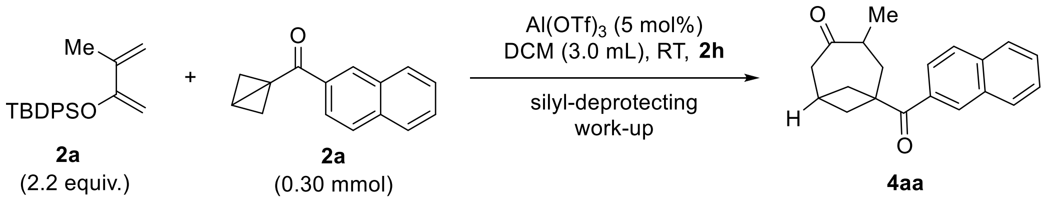

Inside a glove-box, a 20 mL, round-bottomed vial was charged with $Al(OTf)_3$ (7.1 mg, 0.015 mmol, 5 mol%), bicyclo[1.1.0]butan-1-yl(naphthalen-2-yl)methanone (**1a**) (0.062 g, 0.30 mmol) and *tert*-butyl((3-methylbuta-1,3-dien-2-yl)oxy)diphenylsilane (**2a**) (0.21 g, 0.66 mmol). The vial was sealed with a PTFE septum and taken out of the glove-box. DCM (dry; 3.0 mL) was added by syringe. The resulting colorless solution was stirred at room temperature for 2 hours, after which TLC analysis showed the complete conversion of **1a** into **3**.

Work-up 1: The vial was then uncapped, and MeOH (3.0 mL) was added, followed by TMS-OTf (0.20 mL, 1.2 mmol, 4.0 equiv.). The resulting mixture was then stirred at room temperature for 2 hours. It was then concentrated under reduced pressure to provide a wet orange-brown crude oil. The latter was submitted to column chromatography (Biotage flash chromatographer, 25 g SiO_2; EtOAc in pentane, 0 to 50%) to provide 6-(2-naphthoyl)-4-methylbicyclo[4.1.1]octan-3-one (**4aa**) (0.072 g, 0.25 mmol, 82% yield) as a colorless solid.

Work-up 2: The vial was then uncapped and the mixture was then concentrated under reduced pressure. The resulting whitish residue was dissolved in THF (dry; 1.2 mL). The colorless solution was cooled to 0 °C (ice - water bath) prior to the slow addition of TBAF (1.0 M in THF; 0.75 mL, 0.75 mmol, 2.5 equiv.). The solution turned to orange and then to yellow. It was stirred for 4 hours, while allowing it to warm to room temperature. It was then diluted with Et_2O (10 mL) and washed with sat. aq. NH_4Cl (10 mL). The aqueous layer was extracted with EtOAc (4 x 10 mL). The combined organic layers were washed with brine, dried over Na_2SO_4, filtered, and concentrated under vacuum. The resulting pale yellow, crude oil was submitted to column chromatography (Biotage flash chromatographer, 25 g SiO_2; EtOAc in pentane, 0 to 50%) to provide 6-(2-naphthoyl)-4-methylbicyclo[4.1.1]octan-3-one (**4aa**) (0.065 g, 0.22 mmol, 74% yield) as a colorless solid.

Work-up 3: MeOH (dry; 2.6 mL) was added by syringe, followed by HCl (3.0 M in MeOH; 0.40 mL, 1.2 mmol, 4.0 equiv.). The initially slightly turbid solution was stirred at room temperature for 2 hours, becoming clear during this time. The vial was then uncapped, and the mixture was concentrated under vacuum to provide a clear, pale yellow crude oil. The latter was submitted to column chromatography (Biotage flash chromatographer, 25 g SiO_2; EtOAc in pentane, 0 to 50%) to provide 6-(2-naphthoyl)-4-methylbicyclo[4.1.1]octan-3-one (**4aa**) (0.068 g, 0.23 mmol, 78% yield) as colorless solid.

Table 2: Silyl-deprotecting work-up - Summary

	Conditions	Yield
**Work-up 1**	MeOH, TMS-OTf (4.0 equiv.), 2 h, RT	82%
**Work-up 2**	Solvent switch to THF, TBAF (2.5 equiv.), 4 h, 0 °C to RT	74%
**Work-up 3**	MeOH, HCl 3.0 M (4.0 equiv.), 2 h, RT	78%

Practical remark on Work-up 1: Upon concentration, the crude product usually appeared as an oil containing a small amount of dispersed water (see Figure 1). It could be directly loaded on packed SiO_2 for column chromatography by dispersing it in a minimal amount of DCM. The presence of residual water did not compromise the quality of separation.

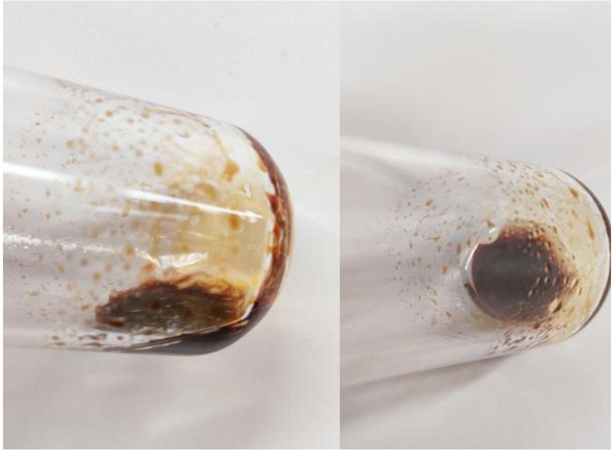

Figure 1: Typical appearance of the crude oil

2.4 Scale-up of the annulation reaction

Inside a glove-box, a 50 mL, two-necked, round-bottomed flask was charged with $Al(OTf)_3$ (0.036 g, 0.075 mmol, 5 mol%), naphthalen-2-yl(3-(4-(trifluoromethyl)phenyl)bicyclo[1.1.0]butan-1-yl)methanone (**1a**) (0.31 g, 1.5 mmol, 1.0 equiv.) and *tert*-butyl((3-methylbuta-1,3-dien-2-yl)oxy)diphenylsilane (**2a**) (1.06 g, 3.30 mmol, 2.2 equiv.). The flask was sealed with septums, taken out of the glove box, and rapidly connected to a nitrogen line. DCM (dry; 15 mL) was added by syringe, and the resulting colorless solution was stirred at room temperature for 2 hours. After this time, MeOH (15 mL) and TMS-OTf (1.0 mL, 6.0 mmol, 4.0 equiv.) were then added into the open flask. The resulting turbid mixture was stirred at room temperature for 4 hours. It was then concentrated under reduced pressure. The so-obtained wet, orange crude oil was submitted to column chromatography (Biotage flash chromatographer, 40 g SiO_2; EtOAc in pentane, 0 to 50%) to provide 6-(2-naphthoyl)-4-methylbicyclo[4.1.1]octan-3-one (**4aa**) (0.249 g, 1.19 mmol, 80% yield) as a colorless solid.

3. Scope of the annulation reaction

General procedures (GPs) for the annulation reaction between BCB ketones 2 and dienol silyl ethers (DSE) 2.

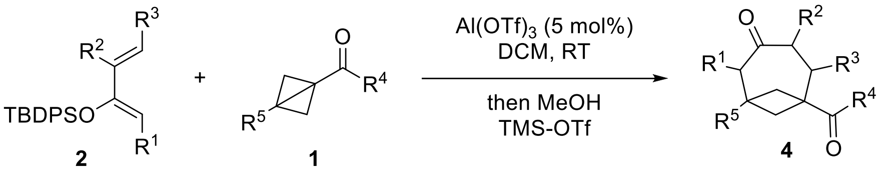

GP2 – with solid DSEs and solid BCB ketones used neat

Inside a glove-box, a 20 mL, round-bottomed vial was charged with $Al(OTf)_3$ (7.1 mg, 0.015 mmol, 5 mol%), the DSE **2** (0.66 mmol, 2.2 equiv.), and the BCB ketone **1** (0.30 mmol, 1.0 equiv.). The vial was sealed with a PTFE septum and taken out of the glove-box. DCM (dry; 3.0 mL) was added by syringe. The resulting (usually colorless and clear) solution was stirred at room temperature for the indicated time.[1] The vial was then uncapped, and MeOH (3.0 mL) was added, followed by TMS-OTf (0.20 mL, 1.2 mmol, 4.0 equiv.). The resulting mixture was then stirred at room temperature for 2 to 6 hours. It was then concentrated under reduced pressure to provide a wet crude oil (usual colors: orange or red). The BCO ketone product **4** was isolated upon column chromatography on SiO_2.

GP3 – with solid DSEs and BCB ketones used as 0.25 M solutions in DCM

Inside a glove-box, a 20 mL, round-bottomed vial was charged with $Al(OTf)_3$ (7.1 mg, 0.015 mmol, 5 mol%) and the DSE **2** (0.66 mmol, 2.2 equiv.). The vial was sealed with a PTFE septum and taken out of the glove-box. DCM (dry; 1.8 mL) was added by syringe. A solution of the BCB ketone **1** in DCM (0.25 M; 1.2 mL, 0.30 mmol, 1.0 equiv.) was then also added by syringe. The resulting (usually colorless and clear) solution was stirred at room temperature for the indicated time.[1] The vial was then uncapped, and MeOH (3.0 mL) was added, followed by TMS-OTf (0.20 mL, 1.2 mmol, 4.0 equiv.). The resulting mixture was then stirred at room temperature for 2 to 6 hours. It was then concentrated under reduced pressure to provide a wet crude oil (usual colors: orange or red). The BCO ketone product **4** was isolated upon column chromatography on SiO_2.

GP4 – with liquid DSEs and solid BCB ketones used neat

Inside a glove-box, a 20 mL, round-bottomed vial was charged with $Al(OTf)_3$ (7.1 mg, 0.015 mmol, 5 mol%) and the BCB ketone **1** (0.30 mmol, 1.0 equiv.). The vial was sealed with a PTFE septum and taken out of the glove-box. A solution of the DSE **2** (0.66 mmol, 2.2 equiv.) in DCM (dry; 3.0 mL) was then added by syringe. The resulting (usually colorless and clear) solution was stirred at room temperature for the indicated time.[1] The vial was then uncapped, and MeOH (3.0 mL) was added, followed by TMS-OTf (0.20 mL, 1.2 mmol, 4.0 equiv.). The resulting mixture was then

[1] Once the reaction was finished according to TLC analysis, the mixture could be stored in a freezer prior to being treated with MeOH/TMS-OTf.

stirred at room temperature for 2 to 6 hours. It was then concentrated under reduced pressure to provide a wet crude oil (usual colors: orange or red). The BCO ketone product **4** was isolated upon column chromatography on SiO_2.

6-Benzoyl-4-methylbicyclo[4.1.1]octan-3-one (4ba)

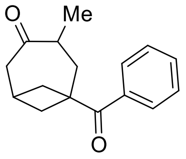

4ba

It was prepared following the **GP3** and using bicyclo[1.1.0]butan-1-yl(phenyl)methanone **1b** (0.25 M solution in DCM; 1.2 mL, 0.30 mmol, 1.0 equiv.) and *tert*-butyl((3-methylbuta-1,3-dien-2-yl)oxy)diphenylsilane (**2a**) (0.21 g, 0.66 mmol, 2.2 equiv.). Upon column chromatography (Biotage flash chromatographer, 12 g SiO_2; EtOAc in pentane, 0 to 40%), the title compound **4ba** (0.061 g, 0.25 mmol, 84% yield) was obtained as a colorless solid.

M.P. 85.1-88.5 °C.

R_f (pentane/EtOAc 9/1) 0.25.

^{1}H NMR (400 MHz, Chloroform-*d*) δ 7.83 – 7.74 (m, 2H, Ph*H*), 7.54 (m, 1H, Ph*H*), 7.44 (t, *J* = 7.6 Hz, 2H, Ph*H*), 3.25 (dp, *J* = 12.8, 6.4 Hz, 1H, C*H*CH_3), 2.90 (dd, *J* = 12.1, 8.8 Hz, 1H, C*H_2*), 2.82 (dd, *J* = 12.0, 8.4 Hz, 1H, C*H_2*), 2.74 – 2.64 (m, 2H, C*H_2*), 2.56 – 2.45 (m, 2H, C*H* and C*H_2*), 2.11 (ddd, *J* = 13.7, 6.2, 1.4 Hz, 1H, C*H_2*), 1.74 (m, 1H, C*H_2*), 1.69 (dd, *J* = 13.7, 11.9 Hz, 1H, C*H_2*), 1.13 (d, *J* = 6.4 Hz, 2H, C*H_3*).

^{13}C NMR (101 MHz, Chloroform-*d*) δ 213.3, 203.3, 134.0, 133.0, 129.1, 128.6, 51.9, 48.6, 43.1, 41.3, 36.2, 33.4, 25.0, 15.5.

IR ($\tilde{\nu}_{max}$, cm^{-1}) 2971 (m), 2935 (m), 1706 (s), 1673 (s), 1597 (m), 1451 (m), 1375 (w), 1317 (m), 1270 (s), 1216 (w), 1187 (w), 1158 (w), 1056 (m), 968 (m), 910 (s), 858 (m), 820 (m), 730 (s).

HRMS (APCI/QTOF) m/z: $[M + H]^+$ Calcd for $C_{16}H_{19}O_2^+$ 243.1380; Found 243.1376.

6-(4-methoxybenzoyl)-4-methylbicyclo[4.1.1]octan-3-one (4ca)

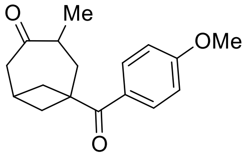

4ca

It was prepared following the **GP3** and using bicyclo[1.1.0]butan-1-yl(4-methoxyphenyl)methanone **1c** (0.25 M solution in DCM; 1.2 mL, 0.30 mmol, 1.0 equiv.) and *tert*-butyl((3-methylbuta-1,3-dien-2-yl)oxy)diphenylsilane (**2a**) (0.21 g, 0.66 mmol, 2.2 equiv.). Upon column chromatography (Biotage flash chromatographer, 25 g SiO_2; EtOAc in pentane, 0 to 40%), the title compound **4ca** (0.069 g, 0.25 mmol, 84% yield) was obtained as a viscous, colorless oil, which converted into an amorphous, pasty, colorless solid upon standing at 4 °C.

R_f (pentane/EtOAc 85/15) 0.33.

^{1}H NMR (400 MHz, Chloroform-*d*) δ 7.78 (d, *J* = 9.0 Hz, 2H, Ar*H*), 6.90 (d, *J* = 8.9 Hz, 2H, Ar*H*), 3.85 (s, 3H, OC*H_3*), 3.23 (dp, *J* = 12.6, 6.3 Hz, 1H, C*H*CH_3), 2.86 (dd, *J* = 12.1, 8.7 Hz, 1H, C*H_2*), 2.79 (dd, *J* = 11.8, 8.2 Hz, 1H, C*H_2*), 2.72–2.62 (m, 2H, C*H_2*), 2.53–2.42 (m, 2H, C*H* and C*H_2*),

2.09 (ddd, *J* = 13.6, 6.2, 1.2 Hz, 1H, C*H₂*), 1.70 (q, *J* = 6.3 Hz, 1H, C*H₂*), 1.66 (m, 1H, C*H₂*), 1.11 (d, *J* = 6.4 Hz, 3H, C*H₃*).

^{13}C NMR (101 MHz, Chloroform-*d*) δ 213.4, 202.2, 163.3, 131.4, 126.8, 113.8, 55.5, 51.8, 48.7, 43.1, 41.5, 36.3, 33.5, 24.9, 15.4.

IR ($\tilde{\nu}_{max}$, cm^{-1}) 2967 (m), 2936 (m), 2844 (w), 1704 (m), 1663 (m), 1598 (s), 1510 (m), 1454 (m), 1420 (w), 1375 (w), 1313 (m), 1253 (s), 1213 (w), 1175 (m), 1153 (m), 1113 (w), 1029 (m), 965 (w), 847 (m), 766 (w).

HRMS (nanochip-ESI/LTQ-Orbitrap) m/z: [M + H]$^+$ Calcd for $C_{17}H_{21}O_3^+$ 273.1485; Found 273.1483.

6-(3-Methoxybenzoyl)-4-methylbicyclo[4.1.1]octan-3-one (4da)

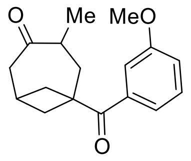

4da

It was prepared following the **GP3** and using bicyclo[1.1.0]butan-1-yl(4-methoxyphenyl)methanone **1d** (0.25 M solution in DCM; 1.2 mL, 0.30 mmol, 1.0 equiv.) and *tert*-butyl((3-methylbuta-1,3-dien-2-yl)oxy)diphenylsilane (**2a**) (0.21 g, 0.66 mmol, 2.2 equiv.). Upon column chromatography (Biotage flash chromatographer, 12 g SiO_2; EtOAc in pentane, 0 to 40%), the title compound **4da** (0.060 g, 0.22 mmol, 73% yield) was obtained as a viscous, colorless oil.

R_f (pentane/EtOAc 85/15) 0.36.

^{1}H NMR (400 MHz, Chloroform-*d*) δ 7.37 (dd, *J* = 2.9, 1.1 Hz, 1H, Ar*H*), 7.35 – 7.31 (m, 2H, Ar*H*), 7.08 (m, 1H, Ar*H*), 3.84 (s, 3H, OC*H₃*), 3.23 (m, 1H, C*H*CH₃), 2.88 (dd, *J* = 12.2, 8.8 Hz, 1H), 2.82 (dd, *J* = 11.9, 8.4 Hz, 1H, C*H₂*), 2.70 – 2.67 (m, 2H, C*H₂*), 2.55 – 2.44 (m, 2H, C*H* and C*H₂*), 2.11 (ddd, *J* = 13.6, 6.2, 1.4 Hz, 1H, C*H₂*), 1.72 (m, 1H, C*H₂*), 1.68 (dd, *J* = 13.5, 11.7 Hz, 1H, C*H₂*), 1.12 (d, *J* = 6.4 Hz, 3H, C*H₃*).

^{13}C NMR (101 MHz, Chloroform-*d*) δ 213.3, 203.4, 159.8, 135.4, 129.5, 121.5, 119.2, 113.7, 55.5, 52.0, 48.6, 43.1, 41.3, 36.2, 33.6, 25.0, 15.5.

IR ($\tilde{\nu}_{max}$, cm^{-1}) 3072 (w), 2964 (m), 2935 (m), 2867 (m), 2131 (w), 1706 (s), 1673 (s), 1593 (m), 1584 (m), 1509 (w), 1486 (m), 1458 (m), 1429 (m), 1377 (w), 1343 (w), 1321 (m), 1272 (s), 1182 (w), 1145 (w), 1111 (w), 1094 (w), 1044 (m), 1018 (w), 982 (w), 906 (w), 885 (w), 845 (w), 823 (w), 800 (m), 781 (m), 752 (s), 704 (m).

HRMS (ESI/QTOF) m/z: [M + Na]$^+$ Calcd for $C_{17}H_{20}NaO_3^+$ 295.1305; Found 295.1315.

6-(2-Methoxybenzoyl)-4-methylbicyclo[4.1.1]octan-3-one (4ea)

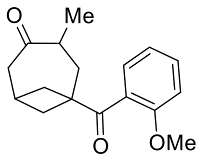

4ea

It was prepared following the **GP3** and using bicyclo[1.1.0]butan-1-yl(2-methoxyphenyl)methanone (**1e**) (0.25 M solution in DCM; 1.2 mL, 0.30 mmol, 1.0 equiv.) and *tert*-butyl((3-methylbuta-1,3-dien-2-yl)oxy)diphenylsilane (**2a**) (0.21 g, 0.66 mmol, 2.2 equiv.). Upon column chromatography (Biotage flash chromatographer, 25 g SiO_2; EtOAc in pentane, 0 to 40%, slow gradient with a plateau of steady

polarity at 20% EtOAc), the title compound **4ea** (0.047 g, 0.17 mmol, 57% yield) was obtained as a crystalline, colorless solid.

M.P. 102.5-104.0 °C.

R$_f$ (pentane/EtOAc 85/15) 0.33.

^{1}H NMR (400 MHz, Chloroform-*d*) δ 7.43 (m, 1H, Ar*H*), 7.39 (dd, *J* = 7.6, 1.7 Hz, 1H, Ar*H*), 7.00 (td, *J* = 7.5, 0.9 Hz, 1H, Ar*H*), 6.91 (d, *J* = 8.3 Hz, 1H, Ar*H*), 3.82 (s, 3H, OC*H$_3$*), 3.13 (dp, *J* = 12.7, 6.3 Hz, 1H, C*H*CH$_3$), 2.70 (dd, *J* = 12.1, 8.7 Hz, 1H, C*H$_2$*), 2.64 – 2.59 (m, 3H, C*H$_2$*), 2.39 (m, 1H, C*H*), 2.33 (dd, *J* = 12.7, 5.7 Hz, 1H, C*H$_2$*), 2.19 (ddd, *J* = 13.4, 6.2, 1.4 Hz, 1H, C*H$_2$*), 1.61 (dd, *J* = 13.3, 11.9 Hz, 1H, C*H$_2$*), 1.54 (m, 1H, C*H$_2$*), 1.12 (d, *J* = 6.4 Hz, 3H, C*H$_3$*).

^{13}C NMR (101 MHz, Chloroform-*d*) δ 213.8, 208.3, 156.9, 132.5, 129.7, 128.0, 120.8, 111.0, 55.3, 52.4, 48.6, 43.1, 39.7, 35.6, 32.6, 24.4, 15.6.

IR ($\tilde{\nu}_{max}$, cm^{-1}) 2969 (m), 2938 (m), 2871 (w), 1703 (s), 1667 (m), 1597 (m), 1486 (m), 1461 (m), 1438 (m), 1371 (w), 1292 (s), 1252 (s), 1215 (w), 1184 (w), 1162 (w), 1116 (m), 1047 (w), 1023 (m), 968 (m), 856 (w), 823 (w), 758 (s).

HRMS (ESI/QTOF) m/z: [M + Na]$^+$ Calcd for $C_{17}H_{20}NaO_3^+$ 295.1305; Found 295.1307.

6-(4-bromobenzoyl)-4-methylbicyclo[4.1.1]octan-3-one (4fa)

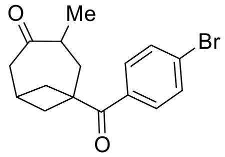

4fa

It was prepared following the **GP3** and using bicyclo[1.1.0]butan-1-yl(4-bromophenyl)methanone **1f** (0.25 M solution in DCM; 1.2 mL, 0.30 mmol, 1.0 equiv.) and *tert*-butyl((3-methylbuta-1,3-dien-2-yl)oxy)diphenylsilane (**2a**) (0.21 g, 0.66 mmol, 2.2 equiv.). Upon column chromatography (Biotage flash chromatographer, 12 g SiO$_2$; EtOAc in pentane, 0 to 40%), the title compound **4fa** (0.064 g, 0.20 mmol, 66% yield) was obtained as a colorless solid.

M.P. 85.0-89.0 °C.

R$_f$ (pentane/EtOAc 85/15) 0.46.

^{1}H NMR (400 MHz, Chloroform-*d*) δ 7.66 (m, 2H, Ar*H*), 7.58 (m, 2H, Ar*H*), 3.22 (m, 1H, C*H*CH$_3$), 2.87 (dd, *J* = 12.0, 8.7 Hz, 1H, C*H$_2$*), 2.79 (dd, *J* = 11.8, 8.4 Hz, 1H, C*H$_2$*), 2.74 – 2.63 (m, 2H, C*H$_2$*), 2.52 (dt, *J* = 8.8, 4.3 Hz, 1H, C*H*), 2.46 (dd, *J* = 12.2, 5.7 Hz, 1H, C*H$_2$*), 2.06 (dd, *J* = 13.6, 6.2 Hz, 1H, C*H$_2$*), 1.71 (dd, *J* = 12.1, 5.7 Hz, 1H, C*H$_2$*), 1.65 (dd, *J* = 13.4, 11.9 Hz, 1H, C*H$_2$*), 1.12 (d, *J* = 6.4 Hz, 3H, C*H$_3$*).

^{13}C NMR (101 MHz, Chloroform-*d*) δ 213.1, 202.5, 132.7, 132.0, 130.6, 128.1, 51.8, 48.6, 43.1, 41.1, 36.1, 33.4, 25.0, 15.4.

IR ($\tilde{\nu}_{max}$, cm^{-1}) 2975 (m), 2935 (m), 2874 (w), 1705 (s), 1677 (s), 1584 (s), 1573 (m), 1483 (m), 1454 (m), 1396 (m), 1375 (m), 1310 (w), 1267 (s), 1220 (m), 1182 (w), 1152 (w), 1108 (w), 1071 (m), 1011 (m), 970 (m), 932 (w), 856 (m), 820 (m), 753 (m).

HRMS (ESI/QTOF) m/z: [M + Na]$^+$ Calcd for $C_{16}H_{17}BrNaO_2^+$ 343.0304 and 345.0293; Found 343.0306 and 345.0285.

4-Methyl-6-(4-(trifluoromethyl)benzoyl)bicyclo[4.1.1]octan-3-one (4ga)

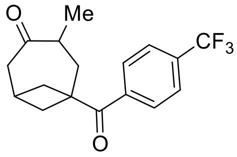

4ga

It was prepared following the **GP3** and using bicyclo[1.1.0]butan-1-yl(4-(trifluoromethyl)phenyl)methanone **1g** (0.25 M solution in DCM; 1.2 mL, 0.30 mmol, 1.0 equiv.) and *tert*-butyl((3-methylbuta-1,3-dien-2-yl)oxy)diphenylsilane (**2a**) (0.21 g, 0.66 mmol, 2.2 equiv.). Full conversion of the starting material **1g** was achieved after stirring the reaction mixture overnight (16 hours).Upon column chromatography (Biotage flash chromatographer, 25 g SiO_2; EtOAc in pentane, 0 to 40%), the title compound **4ga** (ca. 95% pure; 0.066 g, 0.21 mmol, 71% yield) was obtained as a colorless solid.

M.P. 64.7-69.8 °C.

R_f (pentane/EtOAc 9/1) 0.37.

^{1}H NMR (400 MHz, Chloroform-*d*) δ 7.90 (d, *J* = 8.1 Hz, 2H, Ar*H*), 7.71 (d, *J* = 8.3 Hz, 2H, Ar*H*), 3.24 (m, 1H, C*H*CH_3), 2.90 (dd, *J* = 12.1, 8.7 Hz, 1H, C*H_2*), 2.82 (dd, *J* = 11.8, 8.3 Hz, 1H), 2.73 – 2.67 (m, 2H, C*H_2*), 2.55 (m, 1H, C*H*), 2.49 (dd, *J* = 12.5, 5.8 Hz, 1H, C*H_2*), 2.07 (m, 1H, C*H_2*), 1.74 (dd, *J* = 11.9, 5.3 Hz, 1H, C*H_2*), 1.66 (dd, *J* = 13.6, 11.7 Hz, 1H, C*H_2*), 1.13 (d, *J* = 6.4 Hz, 3H, C*H_3*).

^{19}F NMR (376 MHz, Chloroform-*d*) δ -63.2.

^{13}C NMR (101 MHz, Chloroform-*d*) δ 212.9, 202.4, 136.9, 134.3 (q, *J* = 32.8 Hz), 129.3, 125.7 (q, *J* = 3.7 Hz), 123.5 (q, *J* = 272.7 Hz), 51.9, 48.5, 43.1, 40.9, 36.0, 33.3, 25.0, 15.4.

IR ($\tilde{\nu}_{max}$, cm^{-1}) 2938 (w), 2877 (w), 1708 (s), 1678 (m), 1457 (w), 1410 (w), 1326 (s), 1267 (m), 1216 (w), 1170 (s), 1131 (s), 1068 (s), 1018 (w), 968 (w), 860 (m), 825 (w), 771 (w).

HRMS (ESI/QTOF) m/z: $[M + Na]^+$ Calcd for $C_{17}H_{17}F_3NaO_2^+$ 333.1073; Found 333.1057.

4-(3-Methyl-4-oxobicyclo[4.1.1]octane-1-carbonyl)benzonitrile (4ha)

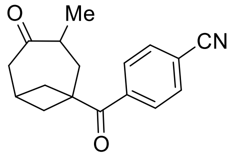

4ha

It was prepared following the **GP3** and using 4-(bicyclo[1.1.0]butane-1-carbonyl)benzonitrile **1g** (0.25 M solution in DCM; 1.2 mL, 0.30 mmol, 1.0 equiv.) and *tert*-butyl((3-methylbuta-1,3-dien-2-yl)oxy)diphenylsilane (**2a**) (0.21 g, 0.66 mmol, 2.2 equiv.). Full conversion of the starting material **1h** was achieved after stirring the reaction mixture overnight (16 hours). Upon column chromatography (Biotage flash chromatographer, 12 g SiO_2; EtOAc in pentane, 0 to 40%), the title compound **4ha** (0.047 g, 0.18 mmol, 59% yield) was obtained as a pale yellow solid.

M.P. 94.4-97.2 °C.

R_f (pentane/EtOAc 9/1) 0.10.

^{1}H NMR (400 MHz, Chloroform-*d*) δ 7.91 – 7.82 (m, 2H, Ar*H*), 7.79 – 7.65 (m, 2H, Ar*H*), 3.23 (m, 1H, C*H*CH_3), 2.89 (dd, *J* = 12.1, 8.7 Hz, 1H, C*H_2*), 2.80 (dd, *J* = 11.9, 8.3 Hz, 1H, C*H_2*), 2.69 (d, *J* = 4.3 Hz, 2H, C*H_2*), 2.55 (dddq, *J* = 8.2, 4.8, 3.2, 1.6 Hz, 1H, C*H_2*), 2.47 (m, 1H, C*H*), 2.05 (ddd,

J = 13.6, 6.2, 1.4 Hz, 1H, C*H₂*), 1.72 (dd, *J* = 12.0, 5.3 Hz, 1H, C*H₂*), 1.63 (dd, *J* = 13.5, 11.8 Hz, 1H, C*H₂*), 1.12 (d, *J* = 6.4 Hz, 3H, C*H₃*).

^{13}C NMR (101 MHz, Chloroform-*d*) δ 212.7, 202.0, 137.3, 132.5, 129.4, 117.9, 116.2, 51.8, 48.4, 43.0, 40.8, 35.9, 33.2, 25.0, 15.4.

IR ($\tilde{v}_{max}$, cm^{-1}) 2971 (m), 2935 (m), 2871 (w), 2231 (w), 1703 (s), 1678 (s), 1606 (w), 1563 (w), 1454 (w), 1404 (w), 1375 (w), 1346 (w), 1317 (w), 1267 (s), 1216 (m), 1187 (w), 1152 (w), 1054 (w), 1014 (w), 967 (m), 915 (w), 860 (m), 822 (w), 764 (m), 730 (m).

HRMS (ESI/QTOF) m/z: $[M + Na]^+$ Calcd for $C_{17}H_{17}NNaO_2^+$ 290.1151; Found 290.1154.

6-(5-Bromofuran-2-carbonyl)-4-methylbicyclo[4.1.1]octan-3-one (4ia)

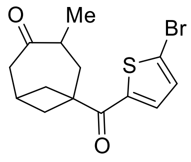

4ia

It was prepared following the **GP3** and using bicyclo[1.1.0]butan-1-yl(4-(trifluoromethyl)phenyl)methanone **1g** (0.25 M solution in DCM; 1.2 mL, 0.30 mmol, 1.0 equiv.) and *tert*-butyl((3-methylbuta-1,3-dien-2-yl)oxy)diphenylsilane (**2a**) (0.21 g, 0.66 mmol, 2.2 equiv.). Upon column chromatography (Biotage flash chromatographer, 25 g SiO_2; EtOAc in pentane, 0 to 40%), the title compound **4ia** (0.069 g, 0.21 mmol, 70% yield) was obtained as a pale yellow solid.

M.P. 98.0-104.0 °C.

R_f (Pentane/EtOAc 85/15) 0.44.

^{1}H NMR (400 MHz, Chloroform-*d*) δ 7.25 (d, *J* = 4.1 Hz, 1H, Ar*H*), 7.08 (d, *J* = 4.0 Hz, 1H, Ar*H*), 3.19 (dq, *J* = 12.8, 6.4 Hz, 1H, C*H*CH₃), 2.84 (dd, *J* = 12.1, 8.8 Hz, 1H, C*H₂*), 2.77 (dd, *J* = 11.8, 8.4 Hz, 1H, C*H₂*), 2.70 – 2.64 (m, 2H, C*H₂*), 2.53 (dt, *J* = 8.2, 3.7 Hz, 1H, C*H*), 2.42 (dd, *J* = 11.8, 5.9 Hz, 1H, C*H₂*), 2.11 (dd, *J* = 12.9, 5.8 Hz, 1H, C*H₂*), 1.71 – 1.62 (m, 2H, C*H₂*), 1.13 (d, *J* = 6.4 Hz, 3H, C*H₃*).

^{13}C NMR (101 MHz, Chloroform-*d*) δ 212.9, 195.8, 142.3, 132.6, 131.2, 122.6, 51.5, 48.5, 43.0, 41.6, 35.8, 33.0, 25.0, 15.4.

IR ($\tilde{v}_{max}$, cm^{-1}) 3097 (w), 2969 (m), 2935 (m), 1705 (s), 1650 (s), 1522 (w), 1451 (m), 1410 (s), 1375 (w), 1323 (m), 1273 (m), 1215 (m), 1057 (w), 980 (m), 954 (w), 848 (w), 817 (m), 787 (m), 748 (w).

HRMS (Sicrit plasma/LTQ-Orbitrap) m/z: $[M + H]^+$ Calcd for $C_{14}H_{16}BrO_2S^+$ 327.0049 and 329.0027; Found 327.0050 and 329.0029.

6-(2-Naphthoyl)-1,4-dimethylbicyclo[4.1.1]octan-3-one (4ja)

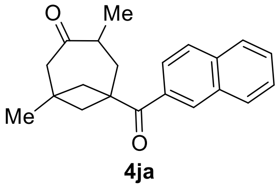

4ja

It was prepared following the **GP3** and using bicyclo[1.1.0]butan-1-yl(4-(trifluoromethyl)phenyl)methanone **1g** (0.25 M solution in DCM; 1.2 mL, 0.30 mmol, 1.0 equiv.) and *tert*-butyl((3-methylbuta-1,3-dien-2-yl)oxy)diphenylsilane (**2a**) (0.21 g, 0.66 mmol, 2.2 equiv.). Upon column chromatography (Biotage flash chromatographer, 25 g SiO_2; EtOAc in pentane, 0 to 40%), the

title compound **4ja** (ca. 95%; 0.014 g, 0.044 mmol, 15% yield) was obtained as a pasty, colorless solid.

R$_f$ (pentane/EtOAc 9/1) 0.38

^{1}H NMR (400 MHz, Chloroform-*d*) δ 8.27 (s, 1H, Ar*H*), 7.93 (d, *J* = 8.0 Hz, 1H, Ar*H*), 7.87 (d, *J* = 9.9 Hz, 3H, Ar*H*), 7.64–7.52 (m, 2H, Ar*H*), 3.29 (m, 1H, C*H*CH$_3$), 2.78 (dd, *J* = 12.0, 5.4 Hz, 1H, C*H$_2$*), 2.69 (m, 1H, C*H$_2$*), 2.62 (d, *J* = 12.1 Hz, 1H, C*H$_2$*), 2.52 (m, 1H, C*H$_2$*), 2.49 (d, *J* = 8.5 Hz, 1H, C*H$_2$*), 2.19 (m, 1H, C*H$_2$*), 2.06 (m, 1H), C*H$_2$*, 1.78 (m, 1H, C*H$_2$*), 1.18 (s, 3H, CC*H$_3$*), 1.12 (d, *J* = 6.4 Hz, 3H, CHC*H$_3$*).

^{13}C NMR (101 MHz, Chloroform-*d*) δ 213.1, 203.6, 135.4, 132.4, 131.3, 130.7, 129.6, 128.6, 128.5, 127.8, 126.8, 124.8, 55.9, 47.6, 43.0, 42.4, 42.0, 40.3, 31.8, 29.9, 15.4.

IR ($\tilde{\nu}_{max}$, cm^{-1}) 2957 (s), 2929 (s), 2865 (m), 1706 (s), 1669 (s), 1627 (m), 1462 (m), 1375 (w), 1357 (w), 1321 (w), 1279 (m), 1181 (w), 1155 (w), 1116 (w), 1005 (w), 912 (w), 867 (w), 831 (w), 784 (w), 740 (m)

HRMS (APCI/QTOF) m/z: [M + Na]$^+$ Calcd for $C_{21}H_{22}NaO_2^+$ 329.1512; Found 329.1499.

Naphthalen-2-yl(3-phenylbicyclo[1.1.0]butan-1-yl)methanone (4ka)

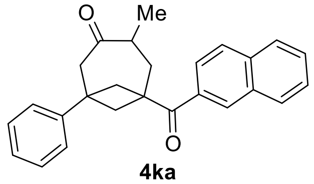

4ka

It was prepared following the **GP2** and using bicyclo[1.1.0]butan-1-yl(4-(trifluoromethyl)phenyl)methanone **1k** (0.085 g, 0.30 mmol, 1.0 equiv.) and *tert*-butyl((3-methylbuta-1,3-dien-2-yl)oxy)diphenylsilane (**2a**) (0.21 g, 0.66 mmol, 2.2 equiv.). Full conversion of the starting material **1k** was achieved after stirring the reaction mixture overnight (18 hours). Upon column chromatography (twice; Biotage flash chromatographer, 25 g SiO_2; EtOAc in pentane, 0 to 50%), the title compound **4ka** (ca. 90% pure; 0.0430 g, 0.105 mmol, 35% yield) was obtained as a pale yellow foam.

R$_f$ (pentane/EtOAc 85/15) 0.46.

^{1}H NMR (400 MHz, Chloroform-*d*) δ 8.29 (s, 1H, Ar*H*), 7.95 (d, *J* = 7.8 Hz, 1H, Ar*H*), 7.91 – 7.83 (m, 3H, Ar*H*), 7.65–7.51 (m, 2H, Ar*H*), 7.32 – 7.27 (m, 2H, Ph*H*), 7.20 – 7.10 (m, 3H, Ph*H*), 3.44 (dt, *J* = 12.8, 6.6 Hz, 1H, C*H*CH$_3$), 3.20 (d, *J* = 4.1 Hz, 1H, C*H$_2$*), 3.14 (d, *J* = 12.2 Hz, 1H, C*H$_2$*), 3.03 (d, *J* = 11.7 Hz, 1H, C*H$_2$*), 2.94 (d, *J* = 14.0 Hz, 1H, C*H$_2$*), 2.73 (d, *J* = 14.0 Hz, 1H, C*H$_2$*), 2.51 (dd, *J* = 11.8, 5.0 Hz, 1H, C*H$_2$*), 2.31 (dd, *J* = 13.8, 5.8 Hz, 1H C*H$_2$*), 1.93 (dd, *J* = 13.6, 11.7 Hz, 1H, C*H$_2$*), 1.18 (d, *J* = 6.4 Hz, 3H, CHC*H$_3$*).

^{13}C NMR (101 MHz, Chloroform-*d*; the signal corresponding to one aliphatic carbon was not resolved) δ 212.3, 203.1, 149.5, 135.4, 132.4, 131.2, 130.7, 129.6, 128.7, 128.6, 128.5, 127.8, 126.9, 126.2, 124.8, 124.6, 58.1, 48.1, 42.6, 42.2, 39.1, 38.5, 15.4.

IR ($\tilde{\nu}_{max}$, cm^{-1}) 3057 (m), 3025 (m), 2968 (m), 2935 (m), 1706 (s), 1671 (s), 1627 (m), 1599 (m), 1550 (m), 1496 (m), 1465 (m), 1353 (m), 1321 (m), 1279 (m), 1191 (m), 1120 (m), 911 (s), 777 (m), 732 (m).

HRMS (ESI/QTOF) m/z: [M + H]$^+$ Calcd for $C_{26}H_{25}O_2^+$ 369.1849; Found 369.1850.

6-(2-Naphthoyl)-1-(3,4-difluorophenyl)-4-methylbicyclo[4.1.1]octan-3-one (4la)

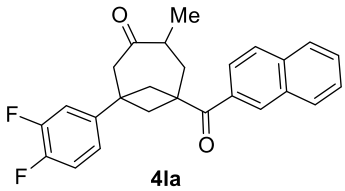

4la

It was prepared following the **GP2** and using (3-(3,4-difluorophenyl)bicyclo[1.1.0]butan-1-yl)(naphthalen-2-yl)methanone **1l** (0.096 g, 0.30 mmol, 1.0 equiv.) and *tert*-butyl((3-methylbuta-1,3-dien-2-yl)oxy)diphenylsilane (**2a**) (0.21 g, 0.66 mmol, 2.2 equiv.). Full conversion of the starting material **1l** was achieved after stirring the reaction mixture overnight (18 hours). Upon column chromatography (twice; Biotage flash chromatographer, 25 g SiO_2; EtOAc in pentane, 0 to 50%), the title compound **4la** (0.048 g, 0.12 mmol, 39% yield) was obtained as a pale yellow foam.

$\mathbf{R_f}$ (pentane/EtOAc 90/10) 0.26

^{1}H NMR (400 MHz, Chloroform-*d*) δ 8.28 (s, 1H, Ar*H*), 7.95 (d, *J* = 7.8 Hz, 1H, Ar*H*), 7.92–7.85 (m, 3H, Ar*H*), 7.64–7.60 (m, 1H), 7.58 (m, 1H, Ar*H*), 7.08 (dt, *J* = 10.2, 8.3 Hz, 1H, Ar*H*), 6.94 (ddd, *J* = 11.2, 7.4, 2.3 Hz, 1H, Ar*H*), 6.85 (d, *J* = 8.6 Hz, 1H, Ar*H*), 3.41 (m, 1H, C*H*CH_3), 3.18 (dd, *J* = 11.9, 5.4 Hz, 1H, CH_2), 3.08 (d, *J* = 12.2 Hz, 1H, CH_2), 2.98 (d, *J* = 11.8 Hz, 1H, CH_2), 2.91 (d, *J* = 13.9 Hz, 1H, CH_2), 2.68 (d, *J* = 14.0 Hz, 1H, CH_2), 2.48 (dd, *J* = 11.7, 5.2 Hz, 1H, CH_2), 2.31 (dd, *J* = 14.4, 5.9 Hz, 1H, CH_2), 1.92 (m, 1H, CH_2), 1.18 (d, *J* = 6.4 Hz, 3H, CH_3).

^{19}F NMR (376 MHz, $CDCl_3$) δ -137.05, -141.06 (d, *J* = 21.2 Hz).

^{13}C NMR (101 MHz, Chloroform-*d*) δ 211.7, 202.8, 150.8 (dd, *J* = 152.8, 12.7 Hz), 148.3 (dd, *J* = 150.8, 12.7 Hz), 146.5 (m), 135.6, 132.5, 131.0, 130.8, 129.7, 128.9, 128.7, 127.9, 127.1, 124.8, 120.7 (dd, *J* = 6.2, 3.6 Hz), 117.5 (d, *J* = 17.2 Hz), 114.0 (d, *J* = 17.3 Hz), 57.9, 47.9, 42.7, 42.1, 41.9, 39.2, 38.2, 15.4.

IR ($\tilde{\nu}_{max}$, cm^{-1}) 3060 (w), 2975 (m), 2939 (m), 2874 (w), 1704 (s), 1670 (s), 1625 (m), 1606 (m), 1519 (s), 1461 (m), 1423 (m), 1376 (w), 1353 (m), 1328 (m), 1281 (s), 1216 (m), 1195 (m), 1173 (m), 1119 (m), 910 (m), 822 (s), 773 (s), 741 (s)

HRMS (ESI/QTOF) m/z: $[M + H]^+$ Calcd for $C_{26}H_{23}F_2O_2^+$ 405.1661; Found 405.1653.

6-(2-Naphthoyl)-4-methyl-1-(4-(trifluoromethyl)phenyl)bicyclo[4.1.1]octan-3-one (4ma)

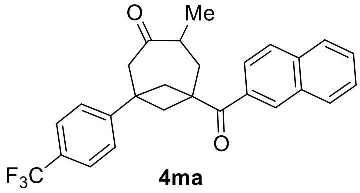

4ma

It was prepared following the **GP2** and using naphthalen-2-yl(3-(4-(trifluoromethyl)phenyl)bicyclo[1.1.0]butan-1-yl)methanone **1m** (0.105 g, 0.30 mmol, 1.0 equiv.) and *tert*-butyl((3-methylbuta-1,3-dien-2-yl)oxy)diphenylsilane (**2a**) (0.21 g, 0.66 mmol, 2.2 equiv.). Full conversion of the starting material **1m** was achieved after stirring the reaction mixture overnight (18 hours). Upon column chromatography (twice; Biotage flash chromatographer, 25 g SiO_2; EtOAc in pentane, 0 to 50%), the title compound **4ma** (0.075 g, 0.17 mmol, 58% yield) was obtained as a colorless foam.

$\mathbf{R_f}$ (pentane/EtOAc 85/15) 0.34.

^{1}H NMR (400 MHz, Chloroform-*d*) δ 8.29 (s, 1H, Ar*H*), 7.96 (d, *J* = 7.7 Hz, 1H, Ar*H*), 7.89 – 7.85 (m, 3H, Ar*H*), 7.64–7.58 (m, 2H, Ar*H*), 7.56 (d, *J* = 8.8 Hz, 2H, Ar*H*), 7.29–7.23 (m, 2H, Ar*H*), 3.44 (dp, *J* = 12.7, 6.4 Hz, 1H, C*H*CH_3), 3.24 (dd, *J* = 12.0, 5.2 Hz, 1H, CH_2), 3.13 (dd, *J* = 12.0, 1.3 Hz, 1H, CH_2), 3.03 (d, *J* = 11.9 Hz, 1H, CH_2), 2.94 (dd, *J* = 14.0, 1.5 Hz, 1H, CH_2), 2.70 (dd, *J* = 14.0, 1.4 Hz, 1H, CH_2), 2.54 (dd, *J* = 11.8, 5.2 Hz, 1H, CH_2), 2.33 (m, 1H, CH_2), 1.94 (dd, *J* = 13.7, 11.8 Hz, 1H), 1.19 (d, *J* = 6.4 Hz, 3H, CH_3).

^{13}C NMR (101 MHz, Chloroform-*d*) δ 211.5, 202.7, 153.3, 135.5, 132.4, 131.0, 130.7, 129.6, 128.8, 128.6, 128.6 (d, *J* = 32.7 Hz), 127.8, 127.0, 125.6 (q, *J* = 3.7 Hz), 125.1, 124.7, 124.6 (q, *J* = 270.1 Hz), 57.6, 48.1, 42.7, 42.0, 41.8, 38.9, 38.6, 15.3.

^{19}F NMR (376 MHz, Chloroform-*d*) δ -62.4.

IR ($\tilde{\nu}_{max}$, cm^{-1}) 3058 (w), 2971 (w), 2937 (w), 1705 (m), 1669 (m), 1622 (m), 1461 (w), 1411 (w), 1324 (s), 1277 (m), 1191 (m), 1166 (s), 1119 (s), 1070 (m), 1018 (m), 974 (w), 935 (w), 840 (m), 778 (w), 765 (w), 737 (m).

HRMS (ESI/QTOF) m/z: $[M + H]^+$ Calcd for $C_{27}H_{24}F_3O_2^+$ 437.1723; Found 437.1718.

4-Methyl-6-pentanoylbicyclo[4.1.1]octan-3-one (4ma)

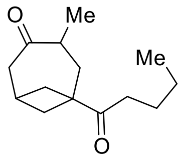

4ma

It was prepared following the **GP3** and using 1-(bicyclo[1.1.0]butan-1-yl)pentan-1-one **1m** (0.25 M solution in DCM; 1.2 mL, 0.30 mmol, 1.0 equiv.) and *tert*-butyl((3-methylbuta-1,3-dien-2-yl)oxy)diphenylsilane (**2a**) (0.21 g, 0.66 mmol, 2.2 equiv.). Upon column chromatography (SiO_2; pentane/Et_2O 15/2 to 5/1), the title compound **4ma** (0.040 g, 0.18 mmol, 59% yield) was obtained as a colorless oil.

R_f (Pentane/Et_2O 85/15) 0.29.

^{1}H NMR (400 MHz, Chloroform-*d*) δ 3.07 (dp, *J* = 12.7, 6.4 Hz, 1H, C*H*CH_3), 2.65 (dd, *J* = 12.0, 8.4 Hz, 1H, CH_2), 2.59 (d, *J* = 4.6 Hz, 2H, CH_2), 2.45 (m, 1H, C*H* or CH_2 in BCO), 2.39 (m, 1H, C*H* or CH_2 in BCO), 2.33 (td, *J* = 7.3, 5.6 Hz, 2H, CH_2 in ^{n}Bu), 2.12 (dd, *J* = 12.1, 5.4 Hz, 1H, CH_2), 1.90 (m, 1H, CH_2), 1.56–1.45 (m, 3H, CH_2 in ^{n}Bu and CH_2 in BCO), 1.40 (dd, *J* = 11.4, 6.5 Hz, 1H, CH_2), 1.27 (dq, *J* = 14.7, 7.4 Hz, 2H, CH_2 in ^{n}Bu), 1.11 (d, *J* = 6.4 Hz, 3H, CHCH_3), 0.88 (t, *J* = 7.3 Hz, 3H, CH_3 in ^{n}Bu).

^{13}C NMR (101 MHz, Chloroform-*d*) δ 213.2, 213.1, 52.3, 48.5, 42.8, 39.5, 35.9, 34.4, 30.7, 25.7, 23.9, 22.4, 15.5, 13.9.

IR ($\tilde{\nu}_{max}$, cm^{-1}) 2958 (m), 2936 (m), 2872 (w), 1704 (s), 1452 (w), 1414 (w), 1375 (w), 1261 (w), 1220 (w), 1186 (w), 1122 (w), 1029 (w), 982 (w), 815 (w), 759 (w).

HRMS (APCI/QTOF) m/z: $[M + H]^+$ Calcd for $C_{14}H_{23}O_2^+$ 223.1693; Found 223.1689.

6-(Cyclohexanecarbonyl)-4-methylbicyclo[4.1.1]octan-3-one (4na)

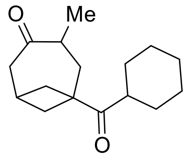

4na

It was prepared following the **GP3** and using bicyclo[1.1.0]butan-1-yl(cyclohexyl)methanone **1n** (0.25 M solution in DCM; 1.2 mL, 0.30 mmol, 1.0 equiv.) and *tert*-butyl((3-methylbuta-1,3-dien-2-yl)oxy)diphenylsilane (**2a**) (0.21 g, 0.66 mmol, 2.2 equiv.). Upon column chromatography (SiO_2; pentane/Et_2O 19/1 to 10/4), the title compound **4na** (0.057 g, 0.23 mmol, 77% yield) was obtained as a colorless oil.

R$_f$ (pentane/Et_2O 9/1) 0.26.

^{1}H NMR (400 MHz, Chloroform-*d*) δ 3.09 (dp, *J* = 12.7, 6.4 Hz, 1H, C*H*CH_3), 2.68 (dd, *J* = 12.1, 8.7 Hz, 1H, CH_2 in BCO), 2.65–2.58 (m, 2H, CH_2 in BCO), 2.56 (t, *J* = 3.3 Hz, 1H, C*H* in Cy), 2.52 (m, 1H, CH or CH_2), 2.42 (ddq, *J* = 11.8, 8.5, 4.7, 4.0 Hz, 1H, C*H* in BCO), 2.15 (dd, *J* = 12.1, 5.5 Hz, 1H, CH_2 in BCO), 1.94 (ddd, *J* = 13.2, 6.2, 1.2 Hz, 1H, CH_2 in BCO), 1.82–1.72 (m, 2H), 1.66 (d, *J* = 12.9 Hz, 2H, CH_2 in Cy), 1.51 (d, *J* = 11.9 Hz, 1H, CH_2 in BCO), 1.47–1.32 (m, 4H, CH_2 in BCO and Cy), 1.32–1.16 (m, 3H, Cy*H*), 1.13 (d, *J* = 6.4 Hz, 3H, CHCH_3).

^{13}C NMR (101 MHz, Chloroform-*d*) δ 215.9, 213.3, 52.5, 48.5, 45.6, 42.9, 39.3, 34.2, 30.9, 29.8, 29.6, 25.7, 23.9, 15.6.

IR ($\tilde{\nu}_{max}$, cm^{-1}) 2971 (m), 2931 (s), 2856 (m), 1703 (s), 1644 (w), 1451 (m), 1372 (w), 1250 (w), 1216 (w), 1139 (w), 1097 (w), 1047 (w), 983 (w), 899 (w), 817 (w), 766 (w), 744 (w).

HRMS (ESI/QTOF) m/z: $[M + Na]^+$ Calcd for $C_{16}H_{24}NaO_2^+$ 271.1669; Found 271.1672.

4-Methyl-6-pivaloylbicyclo[4.1.1]octan-3-one (4oa)

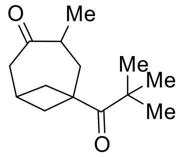

4oa

It was prepared following the **GP3** and using 1-(bicyclo[1.1.0]butan-1-yl)-2,2-dimethylpropan-1-one **1o** (0.25 M solution in DCM; 1.2 mL, 0.30 mmol, 1.0 equiv.) and *tert*-butyl((3-methylbuta-1,3-dien-2-yl)oxy)diphenylsilane (**2a**) (0.21 g, 0.66 mmol, 2.2 equiv.). Upon column chromatography (SiO_2; pentane/Et_2O 15/2 to 5/1), the title compound **4oa** (0.038 g, 0.17 mmol, 54% yield; average with a second reiteration of the experiment: 57% yield) was obtained as a colorless oil.

M.P. 59.6-63.3 °C.

R$_f$ (Pentane/Et_2O 85/15) 0.29.

^{1}H NMR (400 MHz, Chloroform-*d*) δ 3.12 (dp, *J* = 12.5, 6.4 Hz, 1H, C*H*CH_3), 2.72 (dd, *J* = 12.3, 9.0 Hz, 1H, CH_2), 2.65 (m, 1H, CH_2), 2.59 (d, *J* = 4.1 Hz, 2H, CH_2), 2.40 (m, 1H, C*H*), 2.32 (dd, *J* = 12.4, 5.6 Hz, 1H, CH_2), 1.94 (ddd, *J* = 13.4, 6.1, 1.5 Hz, 1H, CH_2), 1.60 – 1.49 (m, 2H, CH_2), 1.18 (s, 9H, C(CH_3)$_3$), 1.12 (d, *J* = 6.4 Hz, 3H, CHCH_3).

^{13}C NMR (101 MHz, Methylene Chloride-*d*$_2$; the signal of an aliphatic carbon was not resolved) δ 218.2, 212.7, 48.2, 43.9, 42.7, 40.2, 35.9, 33.3, 28.0, 24.3, 15.3.

IR ($\tilde{\nu}_{max}$, cm^{-1}) 2969 (m), 2935 (m), 2875 (w), 1685 (s), 1479 (m), 1459 (m), 1364 (w), 1321 (w), 1260 (w), 1220 (w), 1187 (w), 1133 (w), 1115 (w), 1100 (w), 1014 (w), 981 (m), 863 (w), 820 (w), 791 (w), 733 (w).

HRMS (APCI/QTOF) m/z: $[M + H]^+$ Calcd for $C_{14}H_{23}O_2^+$ 223.1693; Found 223.1682.

X-Ray diffraction: *v. infra*.

6-(2-Naphthoyl)bicyclo[4.1.1]octan-3-one (4ab)

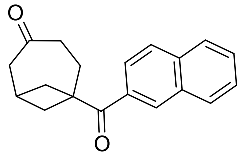

4ba

It was prepared following the **GP4** and using bicyclo[1.1.0]butan-1-yl(naphthalen-2-yl)methanone **1a** (0.063 g, 0.30 mmol, 1.0 equiv.) and (buta-1,3-dien-2-yloxy)(*tert*-butyl)diphenylsilane (**2b**) (0.203 g, 0.660 mmol, 2.2 equiv.). Upon column chromatography (Biotage flash chromatographer, 12 g SiO_2; EtOAc in pentane, 0 to 40%), the title compound **4ab** (0.023 g, 0.082 mmol, 27% yield) was obtained as a colorless oil.

R_f (pentane/EtOAc 85/15) 0.33.

^{1}H NMR (400 MHz, Chloroform-*d*) δ 8.27 (s, 1H, ArH), 7.96–7.84 (m, 4H, ArH), 7.58 (dt, *J* = 20.6, 6.9 Hz, 2H, ArH), 2.98 (td, *J* = 9.4, 2.7 Hz, 2H, C*H₂*), 2.90 (t, *J* = 6.8 Hz, 2H, C*H₂*), 2.70 (d, *J* = 3.9 Hz, 2H, C*H₂*), 2.57 (m, 1H, C*H*), 2.23–2.10 (m, 4H, C*H₂*).

^{13}C NMR (101 MHz, Chloroform-*d*; the signal corresponding to one aliphatic C was not resolved) δ 212.2, 203.5, 135.4, 132.4, 131.3, 130.6, 129.6, 128.6, 128.5, 127.8, 126.9, 124.7, 52.1, 48.3, 40.7, 34.7, 32.3, 25.0.

IR ($\tilde{\nu}_{max}$, cm^{-1}) 3059 (w), 2939 (m), 2867 (w), 1701 (s), 1665 (s), 1626 (m), 1596 (w), 1466 (m), 1447 (m), 1353 (m), 1283 (m), 1232 (m), 1191 (m), 1119 (m), 996 (w), 968 (w), 938 (w), 913 (m), 866 (m), 823 (m), 761 (s), 730 (s).

HRMS (nanochip-ESI/LTQ-Orbitrap) m/z: $[M + H]^+$ Calcd for $C_{19}H_{19}O_2^+$ 279.1380; Found 279.1378.

6-(2-Naphthoyl)-2-methylbicyclo[4.1.1]octan-3-one (4ac)

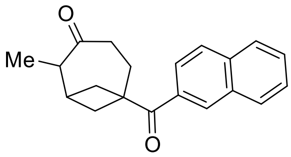

4ac

It was prepared following the **GP2** and using bicyclo[1.1.0]butan-1-yl(naphthalen-2-yl)methanone (**1a**) (0.062 g, 0.30 mmol, 1.0 equiv.) and *(*Z)-tert-Butyl(penta-1,3-dien-3-yloxy)diphenylsilane (**2c**) (0.213 g, 0.660 mmol, 2.2 equiv.)*.* Upon column chromatography (Biotage flash chromatographer, 25 g SiO_2; EtOAc in pentane, 0 to 40%), the title compound **4ac** (0.030 g, 0.10 mmol, 34% yield) was obtained as a colorless solid.

M.P. 95.0-99.7 °C.

R_f (pentane/EtOAc 9/1) 0.25.

^{1}H NMR (400 MHz, Chloroform-*d*) δ 8.27 (s, 1H, Ar*H*), 7.93 (d, *J* = 7.9 Hz, 1H, Ar*H*), 7.91 – 7.84 (m, 3H, Ar*H*), 7.64 – 7.51 (m, 2H, Ar*H*), 3.02 – 2.90 (m, 2H, C*H₂*), 2.87 – 2.75 (m, 3H, C*H₂* and

C*H*CH_3), 2.39 – 2.27 (m, 2H, C*H2* and C*H*), 2.13 (dd, *J* = 8.0, 5.7 Hz, 2H, C*H2*), 1.97 (dd, *J* = 12.5, 5.2 Hz, 1H, C*H2*), 1.12 (d, *J* = 6.8 Hz, 3H, CHC*H3*).

^{13}C NMR (101 MHz, Chloroform-*d*) δ 213.7, 203.7, 135.4, 132.4, 131.4, 130.6, 129.6, 128.6, 128.5, 127.8, 126.8, 124.8, 52.4, 50.0, 39.1, 34.2, 33.0, 32.4, 32.0, 14.8.

IR ($\tilde{\nu}_{max}$, cm^{-1}) 3060 (w), 2966 (m), 2931 (m), 2867 (w), 1704 (s), 1669 (s), 1627 (m), 1596 (w), 1452 (m), 1373 (w), 1355 (w), 1292 (m), 1281 (m), 1231 (w), 1191 (w), 1125 (w), 1101 (w), 1054 (w), 997 (w), 927 (w), 867 (w), 827 (m), 761 (s).

HRMS (ESI/QTOF) m/z: $[M + Na]^+$ Calcd for $C_{20}H_{20}NaO_2^+$ 315.1356; Found 315.1353.

6-(2-Naphthoyl)-2,4-trans-dimethylbicyclo[4.1.1]octan-3-one (4ad)

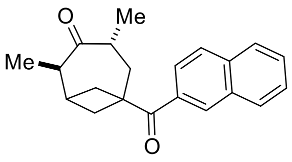

4ad

It was prepared following the **GP2** and using bicyclo[1.1.0]butan-1-yl(naphthalen-2-yl)methanone (**1a**) (0.062 g, 0.30 mmol, 1.0 equiv.) and (Z)-*tert*-butyl((2-methylpenta-1,3-dien-3-yl)oxy)diphenylsilane (**2d**) (0.222 g, 0.660 mmol, 2.2 equiv.). Upon column chromatography (Biotage flash chromatographer, 25 g SiO_2; EtOAc in pentane, 0 to 40%), the title compound **4ad** (0.031 g, 0.10 mmol, 34% yield) was obtained as a crystalline, colorless solid.

M.P. 133.7-138.5 °C.

R_f (pentane/EtOAc 85/15) 0.65.

^{1}H NMR (400 MHz, Chloroform-*d*) δ 8.27 (s, 1H, Ar*H*), 7.94 (d, *J* = 7.9 Hz, 1H, Ar*H*), 7.87 (d, *J* = 9.5 Hz, 3H, Ar*H*), 7.65–7.49 (m, 2H, Ar*H*), 3.34 (tt, *J* = 12.7, 6.3 Hz, 1H, CH_2C*H*CH_3), 2.92 (dd, *J* = 11.4, 9.0 Hz, 1H, C*H2*), 2.84 (m, 1H, CHC*H*CH_3), 2.76 (dd, *J* = 12.5, 8.8 Hz, 1H, C*H2*), 2.59 (dd, *J* = 12.2, 5.6 Hz, 1H, C*H2*), 2.31 (m, 1H, (CO)CHC*H*), 2.11 (ddd, *J* = 13.6, 6.1, 1.4 Hz, 1H, C*H2*), 1.83 (dd, *J* = 12.0, 4.7 Hz, 1H, C*H2*), 1.75 (dd, *J* = 13.5, 11.8 Hz, 1H, C*H2*), 1.25 (d, *J* = 7.2 Hz, 3H, CHCHC*H3*), 1.12 (d, *J* = 6.4 Hz, 3H, CH_2CHC*H3*).

^{13}C NMR (101 MHz, Chloroform-*d*) δ 216.8, 203.7, 135.4, 132.4, 131.5, 130.6, 129.6, 128.6, 128.5, 127.8, 126.8, 124.8, 52.4, 50.6, 41.6, 39.1, 37.0, 31.2, 28.7, 15.5, 15.1.

IR ($\tilde{\nu}_{max}$, cm^{-1}) 3061 (w), 2972 (m), 2935 (m), 2871 (w), 1702 (s), 1669 (s), 1627 (m), 1595 (w), 1461 (m), 1378 (m), 1358 (w), 1278 (m), 1195 (w), 1155 (w), 1126 (w), 1097 (w), 1038 (m), 1007 (m), 951 (m), 915 (s), 867 (w), 827 (w), 807 (w), 763 (m), 734 (s).

HRMS (ESI/QTOF) m/z: $[M + Na]^+$ Calcd for $C_{21}H_{22}NaO_2^+$ 329.1512; Found 329.1521.

X-Ray diffraction: *v. infra*.

6-(2-Naphthoyl)-4,5-cis-dimethylbicyclo[4.1.1]octan-3-one (4ae)

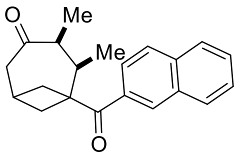

4ae

It was prepared following the **GP4** and using bicyclo[1.1.0]butan-1-yl(naphthalen-2-yl)methanone (**1a**) (0.062 g, 0.30 mmol, 1.0 equiv.) and (*E*)-*tert*-butyl((3-methylpenta-1,3-dien-2-yl)oxy)diphenylsilane (**2e**) (0.247 g, 0.660 mmol, 2.2 equiv.). Upon column chromatography (Biotage flash chromatographer, 25 g SiO_2; Et_2O in pentane, 0 to 50%), the title compound **4ae** (0.008 g, 0.03

mmol, 9% yield) was obtained as a crystalline, colorless solid. 1-(3-(2-Naphthoyl)cyclobutyl)-3-methylpent-3-en-2-one (**4ae'**) (ca 80%; mixture of diastereoisomers, ca. 6 : 4 d.r.; 0.045 g, 0.12 mmol, 39% yield) was the major product, and was isolated as a colorless oil.

R$_f$ (pentane/EtOAc 9/1) 0.45.
^{1}H NMR (400 MHz, Chloroform-*d*) δ 8.34 (s, 1H, Ar*H*), 7.95 (d, *J* = 7.9 Hz, 1H, Ar*H*), 7.88 (d, *J* = 9.9 Hz, 3H, Ar*H*), 7.65–7.52 (m, 2H, Ar*H*), 3.69 (p, *J* = 6.6 Hz, 1H, C*H*), 2.99–2.90 (m, 2H, CH_2), 2.81 (dd, *J* = 12.7, 8.4 Hz, 1H, CH_2), 2.72–2.64 (m, 2H, CH_2), 2.59 (dd, *J* = 12.6, 6.2 Hz, 1H, C*H*), 2.53 (ddd, *J* = 8.4, 5.1, 3.1 Hz, 1H, C*H*), 1.66 (dd, *J* = 12.7, 3.1 Hz, 1H, CH_2), 1.11 (d, *J* = 6.7 Hz, 3H, CHCH_3), 0.70 (d, *J* = 6.8 Hz, 3H, CHCH_3).
^{13}C NMR (101 MHz, Chloroform-*d*) δ 212.5, 203.5, 135.3, 132.5, 131.6, 130.6, 129.7, 128.6, 128.5, 127.9, 127.0, 125.0, 57.5, 48.9, 45.7, 38.7, 36.3, 29.3, 26.2, 13.5, 9.0.
IR ($\tilde{\nu}_{max}$, cm^{-1}) 3059 (w), 2975 (m), 2941 (m), 2880 (w), 1702 (s), 1667 (s), 1627 (m), 1595 (w), 1461 (m), 1443 (w), 1386 (m), 1351 (m), 1321 (w), 1278 (m), 1227 (m), 1199 (m), 1155 (w), 1123 (m), 1083 (w), 1052 (w), 1022 (w), 975 (w), 934 (w), 914 (m), 863 (w), 827 (m), 809 (m), 766 (m), 734 (s)
HRMS (ESI/QTOF) m/z: [M + Na]$^+$ Calcd for $C_{21}H_{22}NaO_2^+$ 329.1512; Found 329.1503.
X-Ray diffraction: *v. infra*.

1-(3-(2-Naphthoyl)cyclobutyl)-3-methylpent-3-en-2-one (4ae')

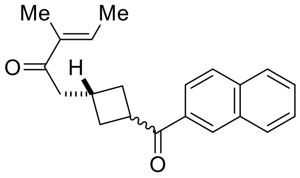

4ae'

R$_f$ (pentane/Et_2OAc 70/30) 0.44.
^{1}H NMR (400 MHz, Chloroform-*d*; the signals assigned to the minor isomer are underlined) δ 8.38 (s, 1H, Ar*H*), 8.34 (s, 1H, Ar*H*), 7.98 (ddd, *J* = 8.6, 4.2, 1.7 Hz, 2.4H, Ar*H*), 7.94 (d, *J* = 8.0 Hz, 2.8H, Ar*H*), 7.90 – 7.84 (m, 5.7H, Ar*H*), 7.62 – 7.49 (m, 5.5H, Ar*H*), 6.78 (m, 1H, C=C*H*CH_3), 6.73 (m, 1H, C=C*H*CH_3), 4.13 (ddd, *J* = 15.1, 9.5, 5.9 Hz, 1H, C*H*), 4.04 (p, *J* = 8.9 Hz, 1H, C*H*), 2.85 (m, 1.4H, CH_2), 2.79 – 2.64 (m, 4.2H, CH_2), 2.58 (qd, *J* = 8.9, 2.4 Hz, 3.4H, CH_2), 2.22 – 2.03 (m, 6.0H, CH_2), 1.88 (d, *J* = 6.9 Hz, 3H, CH_3), 1.84 (d, *J* = 6.9 Hz, 3H, CH_3), 1.76 (d, *J* = 7.6 Hz, 5H, CH_3).
^{13}C NMR (101 MHz, Chloroform-*d*; only clearly resolved signals are reported; the signals corresponding to the two isomers are partially resolved) δ 200.9, 200.6, 138.5, 138.4, 137.5, 137.5, 135.6, 135.5, 133.0, 132.9, 132.6, 130.0, 129.9, 129.5, 129.5, 128.5, 128.4, 128.4, 128.4, 128.3, 127.8, 126.7, 126.7, 43.9, 43.8, 39.2, 38.9, 31.8, 30.3, 28.6, 27.8, 14.8, 14.8, 10.9, 10.9.
HRMS (ESI/QTOF) m/z: [M + Na]$^+$ Calcd for $C_{21}H_{22}NaO_2^+$ 329.1512; Found 329.1502.

***Unsuccessful*: 6-(2-naphthoyl)-5-methylbicyclo[4.1.1]octan-3-one (4af)**

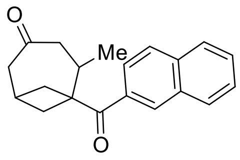

4af
Not formed

An attempt to prepare this compound was made following the **GP4** and using bicyclo[1.1.0]butan-1-yl(naphthalen-2-yl)methanone (**1a**) (0.062 g, 0.30 mmol, 1.0 equiv.) and (E)-*tert*-Butyl(penta-1,3-dien-2-yloxy)diphenylsilane (**2f**) (0.212 g, 0.660 mmol, 2.2 equiv.). Upon stirring the reaction mixture for 2 hours, TLC analysis (pentane/EtOAc 9/1) showed that the starting material **1a** had been completely consumed with the concomitant formation of a mixture of compounds. After the usual hydrolytic quench with MeOH and TMS-OTf, followed by column chromatography (Biotage flash chromatographer, 25 g SiO_2; EtOAc in pentane, 0 to 50%) a mixture of non-annulated cyclobutane derivatives were obtained.

6-(2-Naphthoyl)-4-benzylbicyclo[4.1.1]octan-3-one (4ag)

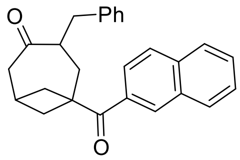

4ag

It was prepared following the **GP4** and using bicyclo[1.1.0]butan-1-yl(naphthalen-2-yl)methanone (**1a**) (0.062 g, 0.30 mmol, 1.0 equiv.) and ((3-benzylbuta-1,3-dien-2-yl)oxy)(*tert*-butyl)diphenylsilane (**2g**) (0.263 g, 0.660 mmol, 2.2 equiv.). Upon column chromatography (Biotage flash chromatographer, 25 g SiO_2; Et_2O in pentane, 0 to 50%), the title compound **4ag** (ca. 95% pure; 0.082 g, 0.23 mmol, 75% yield) was obtained as a colorless solid.

M.P. 142.5-149.5 °C.

$\mathbf{R_f}$ (pentane/Et_2O 80/20) 0.31.

^{1}H NMR (400 MHz, Chloroform-*d*) δ 8.21 (s, 1H, Ar*H*), 7.90 (d, *J* = 7.9 Hz, 1H, Ar*H*), 7.88 – 7.81 (m, 3H, Ar*H*), 7.58 (dddd, *J* = 20.0, 8.1, 6.9, 1.3 Hz, 2H, Ar*H*), 7.30–7.20 (m, 3H, Ph*H*), 7.15 (dd, *J* = 8.6, 7.4 Hz, 2H, Ph*H*), 3.48 (td, *J* = 12.9, 6.9 Hz, 1H, C*H*CH_2Ph), 3.27 (m, 1H, C*H_2*), 2.92 (m, 1H, C*H_2*), 2.87 (m, 1H, C*H_2*), 2.71 (d, *J* = 4.0 Hz, 2H, C*H_2*), 2.60 (dd, *J* = 14.0, 6.5 Hz, 1H, CHC*H_2*Ph), 2.55 (m, 1H, C*H*), 2.50 (m, 1H, C*H_2*), 2.18 (ddd, *J* = 13.6, 5.9, 1.1 Hz, 1H, CHC*H_2*Ph), 1.93–1.85 (m, 2H, C*H_2*).

^{13}C NMR (101 MHz, Chloroform-*d*) δ 212.3, 203.5, 139.9, 135.4, 132.4, 131.2, 130.6, 129.6, 129.0, 128.6, 128.5, 128.4, 127.7, 126.8, 126.2, 124.7, 51.9, 50.9, 49.0, 39.4, 36.4, 36.2, 33.4, 25.1.

IR ($\tilde{\nu}_{max}$, cm^{-1}) 3060 (w), 3028 (w), 2943 (m), 1704 (s), 1627 (m), 1599 (w), 1496 (w), 1453 (m), 1442 (m), 1352 (m), 1280 (m), 1234 (m), 1191 (m), 1120 (m), 978 (w), 939 (w), 913 (m), 867 (w), 827 (m), 777 (m), 760 (s), 732 (s).

HRMS (ESI/QTOF) m/z: $[M + Na]^+$ Calcd for $C_{26}H_{24}NaO_2^+$ 391.1669; Found 391.1669.

6-(2-Naphthoyl)-4-butylbicyclo[4.1.1]octan-3-one (4ah)

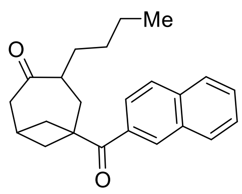

4ah

It was prepared following the **GP2** and using bicyclo[1.1.0]butan-1-yl(naphthalen-2-yl)methanone (**1a**) (0.062 g, 0.30 mmol, 1.0 equiv.) and *tert*-Butyl((3-methylenehept-1-en-2-yl)oxy)diphenylsilane (**2h**) (0.241 g, 0.660 mmol, 2.2 equiv.). Upon column chromatography (Biotage flash chromatographer, 25 g SiO_2; EtOAc in pentane, 0 to 40%), the title compound **4ah** (0.100 g, 0.300 mmol, quantitative yield) was obtained as a crystalline, colorless solid.

M.P. 66.6-68.4 °C.

R_f (pentane/EtOAc 9/1) 0.40.

^{1}H NMR (400 MHz, Chloroform-*d*) δ 8.26 (s, 1H, Ar*H*), 7.93 (d, *J* = 7.8 Hz, 1H, Ar*H*), 7.87 (d, *J* = 9.0 Hz, 3H, Ar*H*), 7.64–7.52 (m, 2H, Ar*H*), 3.13 (dq, *J* = 11.3, 6.3, 5.5 Hz, 1H, C*H*^{n}Bu), 2.95 (dd, *J* = 12.2, 8.8 Hz, 1H, C*H$_2$*), 2.87 (dd, *J* = 11.8, 8.2 Hz, 1H, C*H$_2$*), 2.77–2.65 (m, 2H, C*H$_2$*), 2.59 – 2.48 (m, 2H, C*H* and C*H$_2$*), 2.16 (dd, *J* = 13.8, 4.9 Hz, 1H, C*H$_2$*), 1.95–1.82 (m, 2H, C*H$_2$*), 1.82–1.71 (m, 1H, C*H$_2$*), 1.36–1.15 (m, 5H, C*H$_2$*), 0.86 (t, *J* = 6.9 Hz, 3H, C*H$_3$* in ^{n}Bu).

^{13}C NMR (101 MHz, Chloroform-*d*) δ 213.2, 203.7, 135.4, 132.4, 131.4, 130.7, 129.6, 128.6, 128.5, 127.8, 126.8, 124.8, 52.1, 49.2, 49.0, 39.8, 36.3, 33.5, 30.0, 29.8, 25.1, 22.9, 14.0.

IR ($\tilde{\nu}_{max}$, cm^{-1}) 3059 (w), 2952 (s), 2934 (s), 2864 (m), 1705 (s), 1669 (s), 1627 (m), 1596 (w), 1464 (m), 1441 (m), 1382 (w), 1353 (w), 1279 (s), 1234 (m), 1220 (w), 1218 (w), 1191 (m), 1151 (w), 1122 (m), 1062 (w), 1018 (w), 975 (w), 943 (w), 914 (w), 866 (w), 826 (m), 820 (m), 778 (m), 764 (m), 735 (m).

HRMS (ESI/QTOF) m/z: $[M + Na]^+$ Calcd for $C_{23}H_{26}NaO_2^+$ 357.1825; Found 357.1813.

6-(2-Naphthoyl)-4-cyclohexylbicyclo[4.1.1]octan-3-one (4ai)

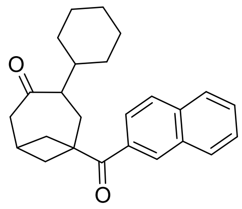

4ai

It was prepared following the **GP4** and using bicyclo[1.1.0]butan-1-yl(naphthalen-2-yl)methanone (**1a**) (0.062 g, 0.30 mmol, 1.0 equiv.) and *tert*-butyl((3-cyclohexylbuta-1,3-dien-2-yl)oxy)diphenylsilane (**2i**) (0.241 g, 0.660 mmol, 2.2 equiv.). Upon column chromatography (Biotage flash chromatographer, 25 g SiO_2; EtOAc in pentane, 0 to 40%), the title compound **4ai** (ca. 95% pure; 0.083 g, 0.23 mmol, 77% yield) was obtained as a colorless solid.

M.P. 144.0-146.5 °C.

R_f (pentane/EtOAc 9/1) 0.50.

^{1}H NMR (400 MHz, Chloroform-*d*) δ 8.25 (s, 1H, Ar*H*), 7.92 (d, *J* = 7.9 Hz, 1H, Ar*H*), 7.86 (d, *J* = 9.0 Hz, 3H, Ar*H*), 7.63–7.51 (m, 2H, Ar*H*), 2.97–2.86 (m, 2H, C*H*Cy and C*H$_2$*), 2.81 (dd, *J* = 11.7, 8.1 Hz, 1H, C*H$_2$*), 2.72 (d, *J* = 12.1 Hz, 1H, C*H$_2$*), 2.65 (dd, *J* = 12.7, 5.8 Hz, 1H, C*H$_2$*), 2.56 – 2.44 (m, 2H, C*H* and C*H$_2$*), 2.20 (m, 1H, C*H$_2$*), 1.88 (dd, *J* = 11.8, 6.1 Hz, 2H, C*H$_2$*), 1.78–1.57 (dt, *J* = 21.9, 12.1 Hz, 6H, Cy*H*), 1.36–1.03 (ddq, *J* = 55.9, 22.6, 11.3, 10.2 Hz, 3H, Cy*H*), 0.95–0.78 (m, 2H, Cy*H*).

^{13}**C NMR** (101 MHz, Chloroform-*d*) δ 213.6, 203.9, 135.4, 132.4, 131.3, 130.7, 129.6, 128.6, 128.5, 127.7, 126.8, 124.8, 54.7, 51.9, 49.8, 37.3, 36.0, 35.8, 33.3, 32.2, 29.8, 26.5, 26.2, 26.2, 25.1.

IR ($\tilde{\nu}_{max}$, cm^{-1}) 3058 (w), 2921 (s), 2856 (m), 1702 (s), 1668 (s), 1624 (m), 1595 (w), 1574 (w), 1508 (w), 1464 (m), 1445 (m), 1382 (w), 1353 (m), 1275 (m), 1234 (w), 1186 (m), 1150 (w), 1119 (m), 1101 (w), 1062 (w), 985 (w), 968 (w), 941 (w), 897 (w), 866 (m), 823 (m), 778 (m), 762 (m), 735 (s), 702 (m).

HRMS (ESI/QTOF) m/z: $[M + H]^+$ Calcd for $C_{25}H_{29}O_2^+$ 361.2162; Found 361.2150.

6-(2-Naphthoyl)-4-(4-methoxyphenyl)bicyclo[4.1.1]octan-3-one (4aj)

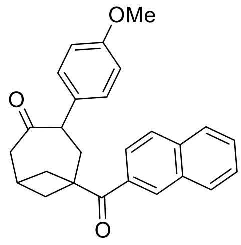

4aj

It was prepared following the **GP4** and using bicyclo[1.1.0]butan-1-yl(naphthalen-2-yl)methanone (**1a**) (0.062 g, 0.30 mmol, 1.0 equiv.) and *tert*-butyl((3-(4-methoxyphenyl)buta-1,3-dien-2-yl)oxy)diphenylsilane (**2j**) (0.249 g, 0.600 mmol, 2.0 equiv.). Full conversion of the starting material **1a** was achieved after stirring the reaction mixture overnight (16 hours); after this time, MeOH (3.0 mL) and TMS-OTf (0.40 mL, 24 mmol, 8.0 equiv.) were added, and the mixture was stirred for 6 hours. Upon column chromatography (Biotage flash chromatographer, 25 g SiO_2; EtOAc in pentane, 0 to 40%), the title compound **4aj** (0.082 g, 0.21 mmol, 71% yield) was obtained as an off-white foam.

$\mathbf{R_f}$ (pentane/EtOAc 85/15) 0.43.

^{1}**H NMR** (400 MHz, Chloroform-*d*) δ 8.29 (s, 1H, Ar*H*), 7.93 (d, *J* = 7.8 Hz, 1H, Ar*H*), 7.86 (d, *J* = 11.4 Hz, 3H, Ar*H*), 7.64 – 7.52 (m, 2H, Ar*H*), 7.27 – 7.20 (m, 2H, Ar*H*), 6.88 – 6.80 (m, 2H, Ar*H*), 4.45 (dd, *J* = 11.0, 7.1 Hz, 1H, C*H*Ar), 3.76 (s, 3H, OCH_3), 3.05 (dd, *J* = 12.3, 8.8 Hz, 1H, CH_2), 2.98 (m, 1H, CH_2), 2.91 (d, *J* = 13.4 Hz, 1H, CH_2), 2.80 (dd, *J* = 13.7, 5.9 Hz, 1H, CH_2), 2.72 (dd, *J* = 12.4, 5.6 Hz, 1H, CH_2), 2.65 (m, 1H, C*H*), 2.34 (m, 1H, CH_2), 2.32 (d, *J* = 5.9 Hz, 1H, CH_2), 2.00 (dd, *J* = 12.3, 5.4 Hz, 1H, CH_2).

^{13}**C NMR** (101 MHz, Chloroform-*d*) δ 210.4, 203.4, 158.8, 135.4, 132.4, 131.4, 130.6, 130.0, 129.9, 129.6, 128.6, 128.5, 127.8, 126.9, 124.7, 113.8, 55.2, 54.1, 52.2, 49.0, 40.9, 36.3, 33.6, 25.1.

IR ($\tilde{\nu}_{max}$, cm^{-1}) 3061 (w), 2939 (m), 2834 (w), 1708 (s), 1668 (s), 1620 (m), 1612 (m), 1513 (s), 1465 (m), 1440 (w), 1353 (w), 1292 (m), 1250 (s), 1184 (m), 1119 (m), 1068 (w), 1033 (m), 975 (w), 867 (w), 828 (s), 781 (m), 736 (m).

HRMS (ESI/QTOF) m/z: $[M + Na]^+$ Calcd for $C_{26}H_{24}NaO_3^+$ 407.1618; Found 407.1618.

6-(2-Naphthoyl)-4-(benzofuran-5-yl)bicyclo[4.1.1]octan-3-one (4ak)

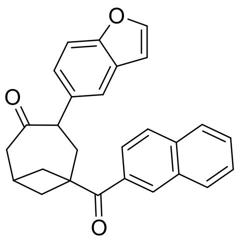

4ak

It was prepared following the **GP4** and using bicyclo[1.1.0]butan-1-yl(naphthalen-2-yl)methanone (**1a**) (0.062 g, 0.30 mmol, 1.0 equiv.) and ((3-(benzofuran-5-yl)buta-1,3-dien-2-yl)oxy)(*tert*-butyl)diphenylsilane (**2k**) (0.255 g, 0.600 mmol, 2.0 equiv.). Full conversion of the starting material **1a** was achieved after stirring the reaction mixture overnight (16 hours); after this time, MeOH (3.0 mL) and TMS-OTf (0.40 mL, 24 mmol, 8.0 equiv.) were added, and the mixture was stirred for 6 hours. Upon column chromatography (Biotage flash chromatographer, 25 g SiO_2; EtOAc in pentane, 0 to 40%), the title compound **4ak** (0.082 g, 0.18 mmol, 61% yield) was obtained as an off-white foam.

R_f (pentane/EtOAc 80/20) 0.47

^{1}H NMR (400 MHz, Chloroform-*d*) δ 8.31 (s, 1H, Ar*H*), 7.94 (d, *J* = 7.8 Hz, 1H, Ar*H*), 7.92–7.84 (m, 2H, Ar*H*), 7.63–7.53 (m, 4H, ArH and OC*H*=CH), 7.44 (d, *J* = 8.5 Hz, 1H, Ar*H*), 7.23 (m, 1H, Ar*H*), 6.71 (dd, *J* = 2.2, 0.9 Hz, 1H, OCH=C*H*), 4.61 (t, *J* = 9.0 Hz, 1H, ArC*H*), 3.05 (ddd, *J* = 21.6, 12.2, 8.6 Hz, 2H, CH_2), 2.96 (d, *J* = 13.3 Hz, 1H, CH_2), 2.85 (d, *J* = 5.9 Hz, 1H, CH_2), 2.83–2.73 (m, 2H, CH_2), 2.68 (m, 1H, C*H*), 2.42 (d, *J* = 9.5 Hz, 2H, CH_2), 2.04 (dd, *J* = 12.1, 5.4 Hz, 1H, CH_2).

^{13}C NMR (101 MHz, Chloroform-*d*) δ 210.4, 203.4, 154.2, 145.3, 135.4, 132.4, 132.3, 131.4, 130.6, 129.6, 128.6, 128.6, 127.8, 127.5, 126.9, 125.5, 124.7, 121.5, 111.1, 106.6, 54.8, 52.3, 49.1, 41.1, 36.4, 33.6, 25.2.

IR ($\tilde{\nu}_{max}$, cm^{-1}) 3058 (w), 2935 (w), 2876 (w), 1709 (s), 1667 (s), 1627 (w), 1595 (w), 1468 (m), 1440 (w), 1353 (w), 1273 (m), 1194 (m), 1116 (m), 1070 (w), 1031 (w), 941 (w), 910 (m), 867 (w), 819 (m), 775 (m), 734 (s)

HRMS (ESI/QTOF) m/z: $[M + Na]^+$ Calcd for $C_{27}H_{22}NaO_3^+$ 417.1461; Found 417.1456.

6-(2-Naphthoyl)-4-(4-chlorophenyl)bicyclo[4.1.1]octan-3-one (4al)

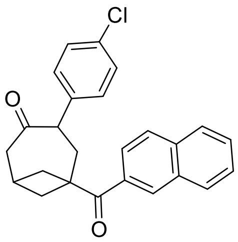

4al

It was prepared following the **GP4** and using bicyclo[1.1.0]butan-1-yl(naphthalen-2-yl)methanone (**1a**) (0.062 g, 0.30 mmol, 1.0 equiv.) and *tert*-butyl((3-(4-chlorophenyl)buta-1,3-dien-2-yl)oxy)diphenylsilane (**2l**) (0.254 g, 0.600 mmol, 2.0 equiv.). Full conversion of the starting material **1a** was achieved after stirring the reaction mixture overnight (16 hours); after this time, MeOH (3.0 mL) and TMS-OTf (0.40 mL, 24 mmol, 8.0 equiv.) were added, and the mixture was stirred for 6 hours. Upon column chromatography (Biotage flash chromatographer, 25 g SiO_2; EtOAc in pentane, 0 to 40%), the title compound **4al** (0.064 g, 0.16 mmol, 55% yield) was obtained as an off-white foam.

R_f (pentane/EtOAc 80/20) 0.50.

^{1}**H NMR** (400 MHz, Chloroform-*d*) δ 8.29 (s, 1H, Ar*H*), 7.93 (d, *J* = 7.7 Hz, 1H, Ar*H*), 7.88 – 7.83 (m, 3H, Ar*H*), 7.58 (dddd, *J* = 18.9, 8.1, 6.9, 1.3 Hz, 2H, Ar*H*), 7.30 – 7.27 (m, 2H, Ar*H*), 7.27 – 7.23 (m, 2H, Ar*H*), 4.46 (dd, *J* = 11.2, 6.9 Hz, 1H, C*H*Ar), 3.07 (m, 1H, C*H*$_2$), 3.00 (m, 1H, C*H*$_2$), 2.92 (d, *J* = 13.2 Hz, 1H, C*H*$_2$), 2.81 (dd, *J* = 13.8, 5.8 Hz, 1H, C*H*$_2$), 2.71 (dd, *J* = 12.9, 6.0 Hz, 1H, C*H*$_2$), 2.64 (m, 1H, C*H*), 2.37 – 2.25 (m, 2H, C*H*$_2$), 2.00 (dd, *J* = 12.1, 5.4 Hz, 1H, C*H*$_2$).

^{13}**C NMR** (101 MHz, Chloroform-*d*) δ 209.6, 203.2, 136.2, 135.4, 133.2, 132.4, 131.3, 130.6, 130.4, 129.6, 128.7, 128.6, 128.5, 127.8, 126.9, 124.6, 54.4, 52.2, 49.0, 40.7, 36.4, 33.5, 25.1.

IR ($\tilde{\nu}_{max}$, cm^{-1}) 3059 (w), 2939 (m), 2867 (w), 1710 (s), 1668 (s), 1626 (m), 1595 (w), 1492 (m), 1466 (w), 1440 (w), 1416 (w), 1354 (m), 1281 (m), 1263 (m), 1231 (w), 1193 (m), 1116 (m), 1093 (m), 1014 (m), 942 (w), 866 (w), 826 (s), 779 (m), 737 (s).

HRMS (ESI/QTOF) m/z: [M + Na]$^+$ Calcd for $C_{25}H_{21}ClNaO_2^+$ 411.1122 and 413.1033; Found 411.1110 and 413.1095.

6-(2-Naphthoyl)-4-(naphthalen-1-yl)bicyclo[4.1.1]octan-3-one (4am)

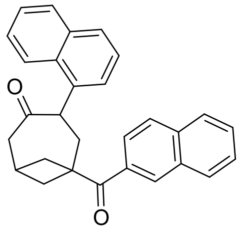

4am

It was prepared following the **GP4** and using bicyclo[1.1.0]butan-1-yl(naphthalen-2-yl)methanone (**1a**) (0.062 g, 0.30 mmol, 1.0 equiv.) and *tert*-butyl((3-(naphthalen-1-yl)buta-1,3-dien-2-yl)oxy)diphenylsilane (**2m**) (0.261 g, 0.600 mmol, 2.0 equiv.). Full conversion of the starting material **1a** was achieved after stirring the reaction mixture overnight (16 hours); after this time, MeOH (3.0 mL) and TMS-OTf (0.40 mL, 24 mmol, 8.0 equiv.) were added, and the mixture was stirred for 6 hours. Upon column chromatography (Biotage flash chromatographer, 25 g SiO_2; EtOAc in pentane, 0 to 40%), the title compound **4an** (0.068 g, 0.17 mmol, 56% yield) was obtained as an off-white foam.

$\mathbf{R_f}$ (pentane/EtOAc 80/20) 0.50.

^{1}**H NMR** (400 MHz, Chloroform-*d*) δ 8.35 (s, 1H, Ar*H*), 7.93 (dd, *J* = 8.6, 1.5 Hz, 2H, Ar*H*), 7.90–7.84 (m, 4H, Ar*H*), 7.77 (d, *J* = 8.2 Hz, 1H, Ar*H*), 7.61 (dd, *J* = 6.9, 1.3 Hz, 1H, Ar*H*), 7.56 (dddd, *J* = 9.6, 8.5, 3.6, 2.2 Hz, 2H, Ar*H*), 7.49 (m, 1H, Ar*H*), 7.46 (d, *J* = 9.3 Hz, 1H, Ar*H*), 7.42 (d, *J* = 7.5 Hz, 1H, Ar*H*), 5.36 (dd, *J* = 12.2, 5.4 Hz, 1H, C*H*Ar), 3.23 (d, *J* = 13.6 Hz, 1H, C*H*$_2$), 3.15 (dd, *J* = 12.4, 8.7 Hz, 1H, C*H*$_2$), 3.07 (dd, *J* = 12.0, 8.3 Hz, 1H, C*H*$_2$), 2.92 (dt, *J* = 11.1, 5.4 Hz, 2H, C*H*$_2$), 2.75 (q, *J* = 8.5, 7.8 Hz, 1H, C*H*$_2$), 2.68 (t, *J* = 12.8 Hz, 1H, C*H*$_2$), 2.54 (m, 1H, C*H*), 2.06 (dd, *J* = 12.4, 5.3 Hz, 1H, C*H*$_2$).

^{13}**C NMR** (101 MHz, Chloroform-*d*) δ 210.4, 203.4, 135.5, 134.1, 133.9, 132.4, 131.6, 131.2, 130.7, 129.7, 129.4, 128.7, 128.6, 128.0, 127.8, 126.9, 126.4, 125.8, 125.5, 125.4, 124.8, 122.4, 52.2, 49.8, 49.2, 38.0, 36.5, 33.8, 25.4.

IR ($\tilde{\nu}_{max}$, cm^{-1}) 3079 (w), 3050 (w), 3024 (w), 2960 (m), 2933 (m), 2892 (w), 2859 (m), 1649 (w), 1607 (w), 1471 (w), 1428 (m), 1409 (w), 1388 (w), 1346 (m), 1317 (w), 1292 (w), 1206 (m), 1110 (m), 1084 (m), 1061 (m), 1019 (w), 985 (w), 939 (w), 900 (m), 822 (m), 799 (w), 741 (m), 704 (s).

HRMS (ESI/QTOF) m/z: [M + Na]$^+$ Calcd for $C_{29}H_{24}NaO_2^+$ 427.1669; Found 427.1672.

4-((tert-Butyldiphenylsilyl)oxy)-N-methoxy-N,3-dimethylbicyclo[4.1.1]oct-3-ene-1-carboxamide (4qa)

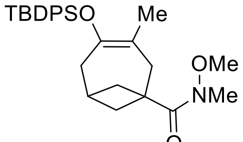

4qa

It was prepared following a modified version of **GP3** and using N-methoxy-N-methylbicyclo[1.1.0]butane-1-carboxamide (**1q**) (1.0 M in DCM; 0.30 mL, 0.30 mmol, 1.0 equiv.) and *tert*-butyl((3-methylbuta-1,3-dien-2-yl)oxy)diphenylsilane (**2a**) (0.212 g, 0.660 mmol, 2.0 equiv.). Full conversion of the starting material **1q** was achieved after stirring the reaction mixture for 48 hours. After this time, the resulting clear, colorless solution was directly concentrated under reduced pressure to provide a turbid, off-white, crude oil. The latter was submitted to column chromatography (Biotage, 25 g SiO_2; Et_2O in pentane (containing 1% v/v Et_3N), 20 to 60%, slow gradient) to provide the title compound **4qa** (0.067 g, 0.15 mmol, 49% yield) as a viscous, yellow oil.

R_f (pentane/Et_2O 3/7) 0.54.

^{1}H NMR (400 MHz, Methylene Chloride-d_2) δ 7.74 (dd, *J* = 7.9, 1.5 Hz, 4H, Ph*H* in TBDPS), 7.45–7.32 (m, 6H, Ph*H* in TBDPS), 3.60 (s, 3H, OCH_3), 3.09 (s, 3H, NCH_3), 2.34 (br s, 2H, CH_2), 2.28 (td, *J* = 8.9, 2.8 Hz, 2H, CH_2), 2.15 (d, *J* = 1.8 Hz, 2H, CH_2), 2.02 (d, *J* = 8.8 Hz, 1H, C*H*), 1.78 (br s, 2H, CH_2), 1.60 (m, 3H, CH_3), 1.07 (s, 9H, C(CH_3)$_3$ in TBDPS).

^{13}C NMR (101 MHz, Methylene Chloride-d_2; the signals corresponding to one sp^2 and one sp^3 carbons were not resolved) δ 143.0, 136.0, 135.2, 130.1, 128.1, 111.2, 61.1, 46.4, 41.0, 40.7, 33.9, 27.2, 25.7, 19.9, 19.3.

IR ($\tilde{\nu}_{max}$, cm^{-1}) 3072 (w), 2960 (m), 2935 (m), 2888 (m), 2860 (m), 2817 (w), 1654 (m), 1469 (w), 1432 (m), 1375 (m), 1154 (s), 1110 (s), 1003 (w), 892 (m), 821 (m), 744 (m), 704 (s).

HRMS (ESI/QTOF) m/z: [M + Na]$^+$ Calcd for $C_{28}H_{37}NNaO_3Si^+$ 486.2435; Found 486.2436.

4. Product modifications

6-(2-Naphthoyl)-4-methylbicyclo[4.1.1]oct-2-en-3-yl trifluoromethanesulfonate (5)

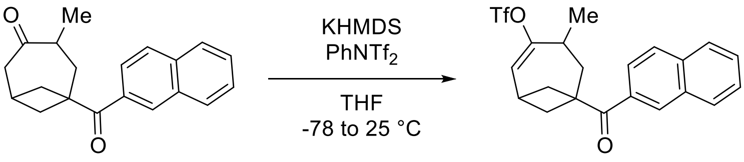

4aa **5**

Following a reported procedure,[28] in a 10 mL, two-necked, round-bottomed flask, 6-(2-naphthoyl)-4-methylbicyclo[4.1.1]octan-3-one (**4aa**) (0.300 g, 1.03 mmol, 1.0 equiv.) and N-phenyl bistriflimide (0.367 g, 1.03 mmol, 1.0 equiv.) were dissolved in THF (dry; 5.1 mL). The clear, pale yellow solution was chilled to -78 °C (dry ice - acetone bath). KHMDS (1.0 M in THF; 1.1 mL, 1.1 mmol, 1.1 equiv.) was then added drop-wise at the same temperature. During the addition, the solution initially became yellow, then bright red and, finally, brown-orange. The mixture was stirred at -78 °C for 30 minutes. The reaction was then quenched by addition of sat.

aq. NH_4Cl (5.0 mL); the mixture was allowed to warm up to room temperature, and the aqueous layer was extracted with EtOAc (3 x 10 mL). The combined organic layers were washed with brine, dried over $MgSO_4$, filtered, and concentrated under vacuum. The resulting pale yellow crude oil was submitted to column chromatography (Biotage, 40 g SiO_2; Et_2O in pentane, 2 to 40%) to provide 6-(2-naphthoyl)-4-methylbicyclo[4.1.1]oct-2-en-3-yl trifluoromethanesulfonate (**5**) (0.312 g, 0.735 mmol, 72% yield) as a pale yellow oil, which became a solid upon standing at 4 °C.

M.P. 86.0-88.0 °C.

R_f (pentane/EtOAc 95/5) 0.42.

^{1}H NMR (400 MHz, Chloroform-*d*) δ 8.30 (s, 1H, Ar*H*), 7.96 (d, *J* = 7.8 Hz, 1H, Ar*H*), 7.91–7.86 (m, 3H, Ar*H*), 7.64–7.54 (m, 2H, Ar*H*), 6.34 (dd, *J* = 9.0, 2.2 Hz, 1H, CHC*H*), 3.45 (m, 1H, C*H*CH_3), 2.98 (dd, *J* = 10.6, 7.9 Hz, 1H, C*H_2*), 2.84–2.71 (m, 2H, C*H* and C*H_2*), 2.55 (dd, *J* = 10.7, 6.2 Hz, 1H, C*H_2*), 2.47 (ddd, *J* = 14.5, 6.2, 1.1 Hz, 1H, C*H_2*), 2.15–2.06 (m, 2H, C*H_2*), 1.34 (d, *J* = 7.1 Hz, 3H, CHC*H_3*).

^{19}F NMR (376 MHz, Chloroform-*d*) δ -73.8.

^{13}C NMR (101 MHz, Chloroform-*d*) δ 202.8, 152.5, 135.5, 132.5, 131.0, 130.6, 129.6, 128.7, 128.6, 127.8, 127.5, 126.9, 124.6, 118.6 (q, *J* = 320.1 Hz), 52.0, 42.7, 41.3, 35.4, 34.8, 27.0, 19.5.

IR ($ν_{max}$, cm^{-1}) 3061 (w), 2985 (w), 2942 (w), 1670 (m), 1627 (w), 1595 (w), 1465 (w), 1413 (s), 1357 (w), 1281 (m), 1245 (m), 1141 (s), 1054 (w), 1014 (w), 971 (m), 934 (w), 885 (m), 863 (m), 831 (m), 764 (m), 737 (w)

HRMS (APCI/QTOF) m/z: $[M + H]^+$ Calcd for $C_{21}H_{20}F_3O_4S^+$ 425.1029; Found 425.1043.

(3-Methyl-4-(p-tolyl)bicyclo[4.1.1]oct-4-en-1-yl)(naphthalen-2-yl)methanone (6)

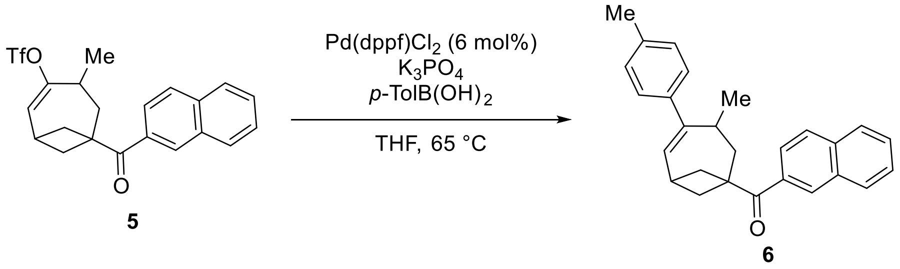

Following a modified version of a reported procedure,[29] inside a glove box, a 25 mL, round-bottomed vial was charged with $Pd(dppf)Cl_2$ (7.8 mg, 0.011 mmol, 5 mol%), K_3PO_4 (0.092 g, 0.43 mmol, 2.0 equiv.), (4-methylphenyl)boronic acid (0.040 g, 0.30 mmol, 1.4 equiv.), and 6-(2-naphthoyl)-4-methylbicyclo[4.1.1]oct-2-en-3-yl trifluoromethanesulfonate (**5**) (0.090 g, 0.21 mmol, 1.0 equiv.). The vial was sealed with a PTFE septum, and it was taken out of the glove-box. THF (dry, degassed by freeze-pump-thaw technique; 1.6 mL) was added by syringe. The resulting orange-brown suspension was stirred at 65 °C for 2.5 hours. After this time, full conversion of the starting material was observed based on TLC analysis (pentane/EtOAc 94/6). The mixture was allowed to cool down to room temperature. It was then filtered through a pad of SiO_2, which was

washed with several portions of DCM. The resulting orange filtrate was concentrated under reduced pressure. The so-obtained brown crude paste was submitted to column chromatography (Biotage, 12 SiO_2; EtOAc in pentane, 2 to 30%) to provide (3-methyl-4-(p-tolyl)bicyclo[4.1.1]oct-4-en-1-yl)(naphthalen-2-yl)methanone (**6**) (0.059 g, 0.16 mmol, 76% yield) as a colorless, viscous oil, which slowly became a solid upon standing at 4 °C (inside a fridge).

M.P. 107.7-109.2 °C.

R_f (pentane/EtOAc 94/6) 0.63.

^{1}H NMR (400 MHz, Chloroform-*d*) δ 8.41 (s, 1H, Ar*H*), 8.03–7.94 (m, 2H, Ar*H*), 7.94–7.84 (m, 2H, Ar*H*), 7.63–7.49 (m, 2H, Ar*H*), 7.18 (d, *J* = 8.2 Hz, 2H, Ar*H*), 7.13 (d, *J* = 7.9 Hz, 2H, Ar*H*), 6.13 (d, *J* = 7.2 Hz, 1H, C=C*H*), 3.47 (qd, *J* = 6.5, 1.6 Hz, 1H, C*H*CH_3), 2.89 – 2.77 (m, 3H, C*H* and/or CH_2), 2.52 (dd, *J* = 14.5, 6.5 Hz, 1H, C*H* or CH_2), 2.43–2.36 (m, 2H, C*H* and/or CH_2), 2.36 (s, 3H, PhCH_3), 2.20 (dd, *J* = 14.7, 6.2 Hz, 1H, C*H* or CH_2), 1.17 (d, *J* = 7.3 Hz, 3H, CHCH_3).

^{13}C NMR (101 MHz, Chloroform-*d*) δ 204.3, 144.9, 142.3, 135.9, 135.3, 133.8, 132.5, 131.7, 130.7, 129.6, 128.6, 128.4, 128.3, 127.8, 127.5, 126.7, 125.0, 52.8, 43.6, 39.9, 38.3, 35.6, 30.8, 23.9, 21.1.

IR (ν_{max}, cm^{-1}) 3017 (m), 2983 (m), 2925 (m), 1670 (s), 1627 (m), 1510 (m), 1459 (w), 1407 (m), 1356 (m), 1277 (m), 1227 (m), 1197 (m), 1152 (m), 1123 (m), 1068 (m), 990 (m), 912 (s), 812 (s), 777 (s), 737 (s).

HRMS (ESI/QTOF) m/z: $[M + Na]^+$ Calcd for $C_{27}H_{26}NaO^+$ 389.1876; Found 389.1870.

(3-Methylbicyclo[4.1.1]oct-4-en-1-yl)(naphthalen-2-yl)methanone (7)

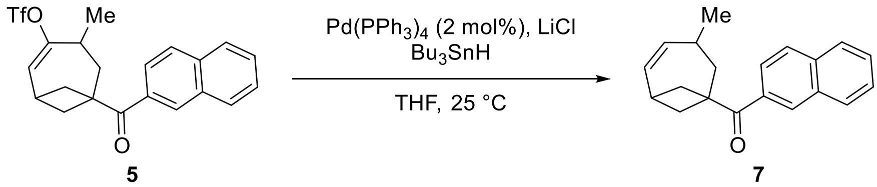

Following a reported procedure,[30] inside a glove-box, a 25 mL, two-necked, round-bottomed flask was charged with $Pd(PPh_3)_4$ (5.8 mg, 0.0050 mmol, 2 mol%), lithium chloride (85 mg, 2.0 mmol, 8.0 equiv.) and 6-(2-naphthoyl)-4-methylbicyclo[4.1.1]oct-2-en-3-yl trifluoromethanesulfonate (**5**) (0.106 g, 0.250 mmol, 1.0 equiv.). The flask was capped with septa, and taken out of the glove-box. THF (dry and degassed by freeze-pump-thaw technique; 2.0 mL) was added, followed by tributylstannane (0.10 mL, 0.37 mmol, 1.5 equiv.). The resulting yellow solution was stirred at room temperature for 3 hours. It was then diluted with EtOAc (6.0 mL) and treated with aq. KF (1.0 M; 0.055 mL). Upon stirring vigorously for 30 minutes, the mixture was filtered through a pad of celite, which was then washed with several portions of EtOAc. The filtrate was concentrated under vacuum. The residue was redissolved in Et_2O (3.0 mL) and stirred again with aq. KF (1.0 M; 0.055 mL) for 40 minutes. To the resulting suspension was added Na_2SO_4. The suspension was then filtered through a pad of celite, which was then washed with additional ether. The filtrate was concentrated to give a pale yellow, oily residue, which was then submitted to column

chromatography (Biotage, 12 g SiO_2; EtOAc in pentane, 1 to 25%) to provide (3-Methylbicyclo[4.1.1]oct-4-en-1-yl)(naphthalen-2-yl)methanone (**7**) (0.046 g, 0.16 mmol, 66% yield) as a colorless oil, which became a solid on standing.

M.P. 91.1-93.3 °C.
R$_f$ (pentane/EtOAc 96/4) 0.62.
^{1}H NMR (400 MHz, Chloroform-*d*) δ 8.33 (s, 1H, Ar*H*), 7.96 – 7.91 (m, 2H, Ar*H*), 7.90 – 7.84 (m, 2H, Ar*H*), 7.57 (dddd, *J* = 20.0, 8.1, 6.9, 1.3 Hz, 2H), 6.07 (ddd, *J* = 10.9, 7.6, 3.1 Hz, 1H, C=C*H*), 5.55 (dd, *J* = 11.0, 1.9 Hz, 1H, C=C*H*), 3.18 (dtq, *J* = 10.8, 5.5, 3.0 Hz, 1H, CHCH_3), 2.96 (m, 1H, CH_2), 2.76 – 2.59 (m, 3H, C*H* and CH_2), 2.29 (dd, *J* = 14.1, 5.7 Hz, 1H, CH_2), 1.90 (dd, *J* = 10.2, 6.4 Hz, 1H, CH_2), 1.83 (dd, *J* = 14.1, 11.0 Hz, 1H, CH_2), 1.14 (d, *J* = 7.1 Hz, 3H, CHCH_3).
^{13}C NMR (101 MHz, Chloroform-*d*) δ 204.3, 135.3, 134.8, 134.7, 132.5, 131.6, 130.5, 129.6, 128.4, 128.3, 127.7, 126.7, 124.9, 52.4, 44.1, 43.8, 34.0, 31.8, 31.2, 23.1.
IR (ν_{max}, cm^{-1}) 3058 (w), 3014 (m), 2957 (m), 2933 (m), 2870 (m), 1670 (s), 1627 (m), 1460 (w), 1353 (m), 1278 (m), 1193 (m), 1153 (m), 1119 (m), 989 (w), 934 (w), 910 (m), 867 (m), 823 (m), 789 (m), 761 (s), 722 (m).
HRMS (ESI/QTOF) m/z: [M + H]$^+$ Calcd for $C_{20}H_{21}O^+$ 277.1587; Found 277.1580.

(3-Methyl-4-vinylbicyclo[4.1.1]oct-4-en-1-yl)(naphthalen-2-yl)methanone (8)

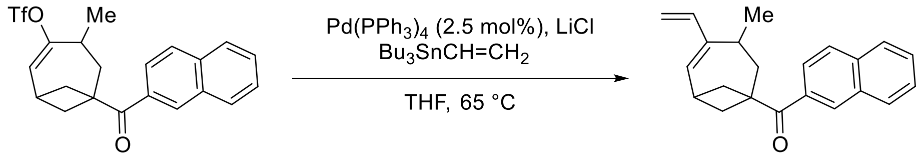

5 **8**

Following a reported procedure,[31] inside a glove-box, a 5 mL round-bottomed vial was charged with $Pd(PPh_3)_4$ (5.8 mg, 0.0050 mmol, 2.5 mol%), anhydrous LiCl (30 mg, 0.7 mmol, 3.5 equiv.) and 6-(2-naphthoyl)-4-methylbicyclo[4.1.1]oct-2-en-3-yl trifluoromethanesulfonate (**5**) (0.085 g, 0.20 mmol, 1.0 equiv.). The vial was sealed with a PTFE septum and taken out of the glove-box. THF (dry and degassed according to the freeze-pump-thaw procedure; 1.5 m) was added by syringe, followed by tributyl vinyltin (0.058 mL, 0.20 mmol, 1.0 equiv.). The pale yellow suspension was then refluxed for 19 hours. It was then allowed to cool down to room temperature and diluted with Et_2O (5 mL). The resulting organic mixture was washed with aq. KF (1.0 M; 3 x 10 mL). The combined aqueous extracts were back-extracted with Et_2O (2 x 10 mL). The combined organic layers were washed with brine, dried over Na_2SO_4, filtered and concentrated under vacuum. The resulting yellow crude oil was submitted to column chromatography (Biotage, 12 g SiO_2; Et_2O in pentane, 0 to 20%) to provide (3-methyl-4-vinylbicyclo[4.1.1]oct-4-en-1-yl)(naphthalen-2-yl)methanone (**8**) (0.0497 g, 0.164 mmol, 82% yield) as a pale yellow, viscous oil.

R$_f$ (Pentane/Et_2O 9/1) 0.67.

^{1}**H NMR** (400 MHz, Chloroform-*d*) δ 8.42 (s, 1H, Ar*H*), 8.00 (dd, *J* = 8.6, 1.7 Hz, 1H, Ar*H*), 7.92 (m, 3H, Ar*H*), 7.59 (dddd, *J* = 21.5, 8.0, 6.9, 1.3 Hz, 2H, Ar*H*), 6.40 (dd, *J* = 17.4, 11.0 Hz, 1H, C*H*=CH_2), 6.27 (d, *J* = 8.1 Hz, 1H, C=C*H*), 5.18 (d, *J* = 17.5 Hz, 1H, CH=CH_2), 5.01 (d, *J* = 10.9 Hz, 1H, CH=CH_2), 3.26 (ddt, *J* = 12.2, 7.6, 3.8 Hz, 1H, C*H*CH_3), 3.95 (m, 1H, C*H* or CH_2), 2.81 (q, *J* = 7.7 Hz, 1H, C*H* or CH_2), 2.72 (m, 2H, C*H* and/or CH_2), 2.45 (dd, *J* = 14.8, 6.9 Hz, 1H, C*H* or CH_2), 2.26 (dt, *J* = 14.7, 2.0 Hz, 1H, C*H* or CH_2), 2.02 (dd, *J* = 10.2, 5.4 Hz, 1H, C*H* or CH_2), 1.54 (d, *J* = 7.5 Hz, 3H, CHCH_3).

^{13}**C NMR** (101 MHz, Chloroform-*d*) δ 204.2, 142.0, 141.1, 137.1, 135.3, 132.5, 131.6, 130.7, 129.5, 128.4, 128.3, 127.8, 126.7, 125.0, 110.5, 53.1, 41.7, 41.6, 36.7, 32.1, 30.6, 25.0.

IR (ν_{max}, cm^{-1}) 2969 (m), 2933 (m), 1670 (s), 1627 (m), 1599 (w), 1461 (w), 1364 (w), 1278 (m), 1198 (w), 1151 (w), 1043 (m), 993 (m), 898 (m), 860 (m), 816 (m), 777 (m), 741 (m).

HRMS (APCI/QTOF) m/z: [M + Na]$^+$ Calcd for $C_{22}H_{22}NaO^+$ 325.1563; Found 325.1561.

Dimethyl 7-(2-naphthoyl)-9-methyl-6,7,8,9-tetrahydro-5H-5,7-methanobenzo[7]annulene-2,3-dicarboxylate (9)

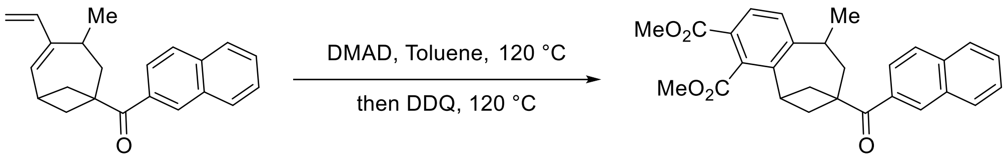

8 **9**

In a 5 mL, round-bottomed vial, sealed with a PTFE septum, (3-methyl-4-vinylbicyclo[4.1.1]oct-4-en-1-yl)(naphthalen-2-yl)methanone (**8**) (0.042 g, 0.14 mmol, 1.0 equiv.) was dissolved in toluene (dry; 2.4 mL). Dimethyl acetylene dicarboxylate (0.026 mL, 0.21 mmol, 1.5 equiv.) was added by syringe. The resulting pale yellow solution was stirred at 120 °C for 6 hours. TLC analysis (pentane/Et_2O 9/1) showed that, after this time, the starting diene had been completely consumed. The solution was allowed to cool down to room temperature. The septum was removed, and DDQ (0.047 g, 0.21 mmol, 1.5 equiv.) was added in a single portion. Immediately, the solution turned into a dark red suspension. The vial was sealed again, and the mixture was stirred at 110 °C for 4 hours. Complete conversion of the previously obtained Diels-Alder cycloadduct was observed based on TLC analysis. The mixture was then allowed to cool down to room temperature. It was then partitioned between EtOAc (10 mL) and water (10 mL). Upon separation, the aqueos layer was extracted with EtOAc (3 x 10 mL). The combined organic layers were washed with brine, dried over Na_2SO_4, filtered, and concentrated under vacuum. The resulting dark brown crude solid was submitted to column chromatography (Biotage, 12 g SiO_2; a few drops of acetone were needed in order to dissolved the solid and load it on the cartridge; EtOAc in pentane, 10 to 60%) to provide dimethyl 7-(2-naphthoyl)-9-methyl-6,7,8,9-tetrahydro-5H-5,7-methanobenzo[7]annulene-2,3-dicarboxylate (**9**) (0.061 g, 0.137 mmol, 99% yield) as a pale yellow, viscous oil.

$\mathbf{R_f}$ (pentane/EtOAc 7/3) 0.58.

^{1}**H NMR** (400 MHz, Chloroform-*d*) δ 8.31 (s, 1H, Ar*H*), 7.95–7.85 (m, 5H, Ar*H*), 7.60 (m, 1H, Ar*H*), 7.55–7.49 (m, 2H, Ar*H*), 3.93 (s, 3H, OC*H₃*), 3.90 (s, 3H, OC*H₃*), 3.86 (m, 1H, C*H*CH₃), 3.46 (t, *J* = 8.3 Hz, 1H, C*H* or C*H₂*), 3.15 (t, *J* = 9.7 Hz, 1H, C*H* or C*H₂*), 2.98 (dd, *J* = 12.0, 5.9 Hz, 1H, C*H* or C*H₂*), 2.88 (dd, *J* = 11.9, 8.3 Hz, 1H, C*H* or C*H₂*), 2.42 (dd, *J* = 14.3, 3.7 Hz, 1H, C*H* or C*H₂*), 2.21 (dd, *J* = 14.2, 10.9 Hz, 1H, C*H* or C*H₂*), 2.07 (dd, *J* = 11.6, 6.7 Hz, 1H), 1.54 (d, *J* = 6.7 Hz, 3H, CHC*H₃*).

^{13}**C NMR** (101 MHz, Chloroform-*d*) δ 203.4, 170.5, 166.0, 148.2, 141.3, 135.4, 135.2, 132.4, 131.5, 130.7, 129.6, 128.7, 128.6, 128.5, 127.9, 127.8, 126.8, 125.4, 124.8, 52.6, 52.4, 52.0, 44.3, 42.6, 35.9, 35.0, 32.2, 21.7.

IR (ν_{max}, cm^{-1}) 2984 (m), 2950 (m), 2879 (w), 1727 (s), 1668 (m), 1591 (m), 1468 (m), 1439 (m), 1353 (m), 1279 (s), 1194 (m), 1149 (m), 1113 (m), 1025 (m), 913 (m), 788 (m), 735 (s)

HRMS (APCI/QTOF) m/z: [M + Na]$^+$ Calcd for $C_{28}H_{26}NaO_5^+$ 465.1672; Found 465.1662.

(3-Methylbicyclo[4.1.1]octan-1-yl)(naphthalen-2-yl)methanone (10)

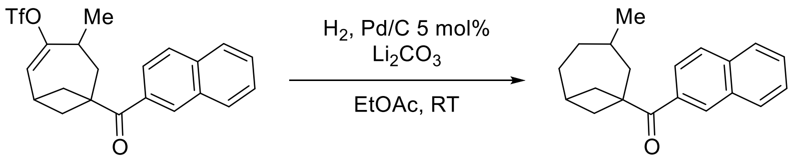

5 **10**

Following a reported procedure,[32] a 25 mL, round-bottomed vial was charged with 6-(2-naphthoyl)-4-methylbicyclo[4.1.1]oct-2-en-3-yl trifluoromethanesulfonate (**5**) (0.059 g, 0.14 mmol, 1.0 equiv.), Pd (5% on charcoal; 0.015 g, 0.0070 mmol, 5 mol%) and Li_2CO_3 (0.021 g, 0.28 mmol, 2.0 equiv.). The vials was sealed with a PTFE septum, and EtOAc (dry; 2.8 mL) was added by syringe. The headspace over the resulting black suspension was evacuated (until incipient bubbling of the mixture) and backfilled with hydrogen (balloon) for 3 times. The mixture was then sparged with hydrogen for 5 minutes and then stirred under an H_2 atmosphere at room temperature. The progress of the reaction were monitored by TLC analysis (pentane/Et_2O 9/1). After 48 hours, full conversion was observed, as well as the formation of both a less polar product and a more polar one compared to the starting material. The mixture was flushed with nitrogen for 5 minutes and then the solids were removed by filtration (micropore syringe filter; washed with several portions of DCM and MeOH). The filtrate was concentrated under reduced pressure. The resulting pale yellow, crude oil was submitted to column chromatography (Biotage, 12 g SiO_2; Et_2O in pentane, 0 to 15%) to provide (3-methylbicyclo[4.1.1]octan-1-yl)(naphthalen-2-yl)methanone (**10**) (19.6 mg, 0.0704 mmol, 50% yield) as a colorless oil. A mixture of different, inseparable over-reduced products was also collected.

R$_f$ (pentane/Et_2O 9/1) 0.68.

^{1}**H NMR** (400 MHz, Chloroform-*d*) δ 8.33 (s, 1H, Ar*H*), 7.97–7.89 (m, 2H, Ar*H*), 7.86 (d, *J* = 8.7 Hz, 2H, Ar*H*), 7.58 (ddd, *J* = 8.2, 6.9, 1.5 Hz, 1H, Ar*H*), 7.54 (ddd, *J* = 8.1, 6.9, 1.4 Hz, 1H, Ar*H*), 2.70 (q, *J* = 9.3, 8.8 Hz, 2H, C*H₂*), 2.44 (q, *J* = 7.4 Hz, 1H, C*H₂*), 2.31–2.11 (m, 3H, C*H₂* and

CHCH3), 2.07 (m, 1H, CH2), 1.85 (m, 1H, CH2), 1.82–1.57 (m, 3H, CH2), 1.46 (dd, J = 13.7, 11.3 Hz, 1H, CH2), 1.01 (d, J = 6.6 Hz, 3H, CHCH3).

^{13}C NMR (101 MHz, Chloroform-*d*) δ 205.5, 135.2, 132.5, 131.9, 130.6, 129.6, 128.3, 128.1, 127.7, 126.6, 125.1, 52.6, 44.1, 35.5, 33.7, 33.6, 32.0, 30.7, 29.8, 23.9.

IR ($ν_{max}$, cm^{-1}) 3059 (w), 2951 (s), 2917 (s), 2848 (m), 1800 (w), 1731 (w), 1667 (s), 1627 (m), 1595 (w), 1457 (m), 1353 (w), 1279 (s), 1233 (m), 1194 (m), 1152 (m), 1122 (m), 985 (m), 934 (w), 910 (m), 865 (m), 821 (m), 763 (m).

HRMS (ESI/QTOF) m/z: $[M + H]^+$ Calcd for $C_{20}H_{23}O^+$ 279.1743; Found 279.1745.

(10a-Methyl-7,8,10,10a-tetrahydro-7,9-methanocyclohepta[b]indol-9(6H)-yl)(naphthalen-2-yl)methanone (11)

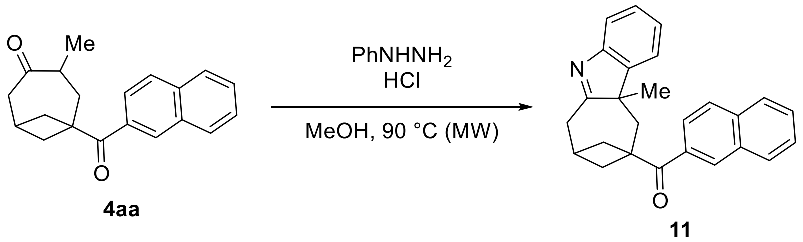

In a sealed 10 mL, round-bottomed vial, 6-(2-naphthoyl)-4-methylbicyclo[4.1.1]octan-3-one (**4aa**) (0.065 g, 0.22 mmol, 1.0 equiv.) was suspended in MeOH (dry; 2.2 mL). Phenyl hydrazine (0.044 mL, 0.45 mmol, 2.0 equiv.) was added by syringe, followed by methanolic HCl (3.0 M; 0.22 mL, 0.66 mmol, 3.0 equiv.). The resulting off-white suspension was stirred in a microwave reactor at 90 °C for 100 minutes, turning into an orange-brown, clear solution. The latter was partitioned between aq. NaOH (1.0 M; 20 mL) and Et_2O (30 mL). Upon separation, the aqueous layer was extracted with Et_2O (3 x 30 mL). The combined organic layers were washed with brine, dried over Na_2SO_4, filtered, and concentrated under vacuum. The resulting yellow-orange crude oil was submitted to column chromatography (Biotage, 12 g SiO_2; EtOAc in pentane, 0 to 80%) to provide (10a-methyl-7,8,10,10a-tetrahydro-7,9-methanocyclohepta[b]indol-9(6H)-yl)(naphthalen-2-yl)methanone (**11**) (0.028 g, 0.077 mmol, 34% yield) as a yellow foam.

R_f (pentane/EtOAc 50/50) 0.59.

^{1}H NMR (400 MHz, Chloroform-*d*) δ 8.27 (s, 1H, ArH), 7.91-7.83 (m, 4H, ArH), 7.63 (m, 2H, ArH), 7.55 (m, 1H, ArH), 7.33 (m, 1H, ArH), 7.18 (s, 1H, ArH), 7.18 (s, 1H, ArH), 3.35 (dd, J = 15.3, 5.9 Hz, 1H, CH), 3.03 (dd, J = 18.2, 9.5 Hz, 2H, CH2), 2.90 (dd, J = 12.0, 8.3 Hz, 1H, CH2), 2.81 (dd, J = 12.5, 5.5 Hz, 1H, CH2), 2.67 (m, 1H, CH2), 2.57 (d, J = 14.3 Hz, 1H, CH2), 1.95 (d, J = 14.3 Hz, 1H, CH2), 1.78 (dd, J = 13.1, 6.2 Hz, 1H, CH2), 1.75 (s, 3H, CH3).

^{13}C NMR (101 MHz, Chloroform-*d*) δ 205.1, 189.9, 154.6, 151.0, 137.2, 134.3, 133.3, 132.5, 131.5, 130.4, 130.3, 129.6, 129.3, 128.7, 127.5, 126.7, 122.9, 121.9, 58.5, 44.5, 38.3, 37.1, 36.9, 31.9, 28.3, 27.6.

IR (ν_{max}, cm^{-1}) 3059 (w), 2967 (m), 2939 (m), 2861 (w), 1670 (s), 1627 (w), 1598 (w), 1567 (m), 1508 (w), 1465 (m), 1436 (w), 1378 (w), 1354 (w), 1299 (m), 1278 (m), 1235 (m), 1195 (w), 1159 (w), 1119 (w), 936 (w), 910 (m), 866 (w), 827 (w), 778 (m), 752 (s), 735 (s)
HRMS (ESI/QTOF) m/z: $[M + H]^+$ Calcd for $C_{26}H_{24}NO^+$ 366.1852; Found 366.1866.

5. Refereces

[1] D. Ni, S. Hu, X. Tan, Y. Yu, Z. Li, L. Deng, *Angew. Chem. Int. Ed.* **2023**, *62*, e202308606.

[2] R. Guo, Y.-C. Chang, L. Herter, C. Salome, S. E. Braley, T. C. Fessard, M. K. Brown, *J. Am. Chem. Soc.* **2022**, *144*, 7988–7994.

[3] M. de Robichon, T. Kratz, F. Beyer, J. Zuber, C. Merten, T. Bach, *J. Am. Chem. Soc.* **2023**, *145*, 24466–24470.

[4] N. Radhoff, C. G. Daniliuc, A. Studer, *Angew. Chem. Int. Ed.* **2023**, *62*, e202304771.

[5] B. D. Schwartz, M. Y. Zhang, R. H. Attard, M. G. Gardiner, L. R. Malins, *Chem. Eur. J.* **2020**, *26*, 2808–2812.

[6] J. McCartney, A. R. Abela, S. Abraham, C. D. Anderson, V. Arumugan, J. Chau, J. Clemens, T. Cleveland, T. R. Coon, T. A. Dwight, et al., **2022**, WO2022/76622 A2.

[7] R. Bychek, P. K. Mykhailiuk, *Angew. Chem. Int. Ed.* **2022**, *61*, e202205103.

[8] L. Guo, A. Noble, V. K. Aggarwal, *Angew. Chem. Int. Ed.* **2021**, *60*, 212–216.

[9] S. H. Bennett, A. Fawcett, E. H. Denton, T. Biberger, V. Fasano, N. Winter, V. K. Aggarwal, *J. Am. Chem. Soc.* **2020**, *142*, 16766–16775.

[10] A. Bauer, G. Di Mauro, J. Li, N. Maulide, *Angew. Chem. Int. Ed.* **2020**, *59*, 18208–18212.

[11] H. Xu, J.-L. Hu, L. Wang, S. Liao, Y. Tang, *J. Am. Chem. Soc.* **2015**, *137*, 8006–8009.

[12] S. D. Mendoza, M. Rombola, Y. Tao, S. J. Zuend, R. Götz, M. J. McLaughlin, S. E. Reisman, *Org. Lett.* **2022**, *24*, 3802–3806.

[13] H.-J. Liu, D.-X. Wang, J. B. Kim, E. N. C. Browne, Y. Wang, *Can. J. Chem.* **1997**, *75*, 899–912.

[14] M. S. . K. Baranov Zinaida M.; Gritcenko, Roman; Postikova, Svetlana G.; Ivashkin, Pavel E.; Kislukhin, Alexander A.; Moskvin, Dmitrii I.; Mineev, Konstantin S.; Arseniev, Alexander S.; Labas, Yulii A.; Yampolsky, Ilia V., *Synlett* **2017**, *28*, 583–588.

[15] G. Schwertz, M. C. Witschel, M. Rottmann, R. Bonnert, U. Leartsakulpanich, P. Chitnumsub, A. Jaruwat, W. Ittarat, A. Schäfer, R. A. Aponte, et al., *J. Med. Chem.* **2017**, *60*, 4840–4860.

[16] Y. Tan, W. Yuan, L. Gong, E. Meggers, *Angew. Chem. Int. Ed.* **2015**, *54*, 13045–13048.

[17] B. B. C. Peters, J. Jongcharoenkamol, S. Krajangsri, P. G. Andersson, *Org. Lett.* **2021**, *23*, 242–246.

[18] E. P. Siqueira Filho, J. A. R. Rodrigues, P. J. S. Moran, *Tetrahedron: Asymmetry* **2001**, *12*, 847–852.

[19] C.-Z. Zhu, J.-J. Feng, J. Zhang, *Angew. Chem. Int. Ed.* **2017**, *56*, 1351–1355.

[20] M. G. Hunter, R. P. Beckett, J. M. Clements, W. Mark, S. J. Davies, L. M. Pratt, Z. M. Spavold, S. Launchbury, *EP1210330 B1*, **2005**.

[21] L. Herter, I. Koutsopetras, L. Turelli, T. Fessard, C. Salomé, *Org. Biomol. Chem.* **2022**, *20*, 9108–9111.

[22] F. Romanov-Michailidis, K. F. Sedillo, J. M. Neely, T. Rovis, *J. Am. Chem. Soc.* **2015**, *137*, 8892–8895.

[23] W. J. Kerr, A. J. Morrison, M. Pazicky, T. Weber, *Org. Lett.* **2012**, *14*, 2250–2253.

[24] M. Baggot, *WO2021/252538 A2*, **2021**.

[25] S.-S. Jiang, B.-Q. Gu, M.-Y. Zhu, X. Yu, W.-P. Deng, *Tetrahedron* **2015**, *71*, 1187–1191.

[26] M. W. Justik, G. F. Koser, *Tetrahedron Lett.* **2004**, *45*, 6159–6163.

[27] L. K. Johnson, G. L. Barnes, S. A. Fernandez, C. D. Vanderwal, *Angew. Chem. Int. Ed.* **2023**, *62*, e202303228.

[28] X. Yang, D. Wu, Z. Lu, H. Sun, A. Li, *Org. Biomol. Chem.* **2016**, *14*, 5591–5594.

[29] Y. Liu, S. Battaglioli, L. Lombardi, A. Menichetti, G. Valenti, M. Montalti, M. Bandini, *Org. Lett.* **2021**, *23*, 4441–4446.

[30] P. A. Wender, M. K. Hilinski, N. Soldermann, S. L. Mooberry, *Org. Lett.* **2006**, *8*, 1507–1510.

[31] W. J. Scott, J. K. Stille, *J. Am. Chem. Soc.* **1986**, *108*, 3033–3040.

[32] D. L. Comins, R. S. Al-awar, *J. Org. Chem.* **1995**, *60*, 711–716.

6. Crystallographic Data

4-Methyl-6-pivaloylbicyclo[4.1.1]octan-3-one (4pa)

CCDC deposition Number 2312246

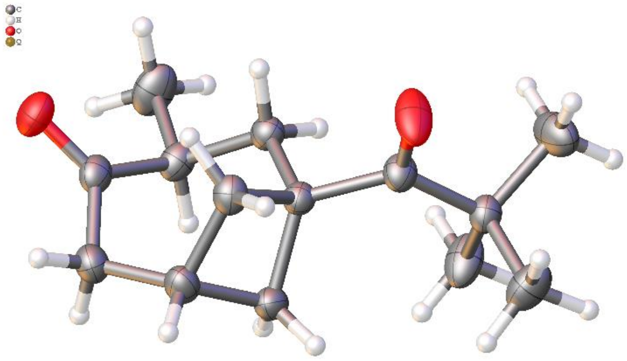

Crystals of compound **5pa** were collected upon spontaneous crystallization out of deuterated chloroform at room temperature.

Experimental. Single clear pale colourless prism-shaped crystals of **4pa** were used as supplied. A suitable crystal with dimensions 0.62 × 0.09 × 0.06 mm^3 was selected and mounted on a SuperNova, Dual, Cu at home/near, AtlasS2 diffractometer. The crystal was kept at a steady T = 140.00(10) K during data collection. The structure was solved with the ShelXT (Sheldrick, 2015)

solution program using dual methods and by using Olex2 1.5 (Dolomanov et al., 2009) as the graphical interface. The model was refined with ShelXL 2019/3 (Sheldrick, 2015) using full matrix least squares minimisation on $\boldsymbol{F^2}$.

Crystal Data. $C_{14}H_{22}O_2$, M_r = 222.31, monoclinic, $P2_1/c$ (No. 14), a = 6.17394(11) Å, b = 8.78352(19) Å, c = 24.0129(5) Å, β = 91.6839(18)°, α = γ = 90°, V = 1301.63(4) Å^3, T = 140.00(10) K, Z = 4, Z' = 1, μ(Cu K_α) = 0.578, 6239 reflections measured, 2676 unique (R_{int} = 0.0262) which were used in all calculations. The final wR_2 was 0.1110 (all data) and R_1 was 0.0405 (I≥2 σ(I)).

Compound 4oa

Formula	$C_{14}H_{22}O_2$
$D_{calc.}$/ g cm^{-3}	1.134
μ/mm^{-1}	0.578
Formula Weight	222.31
Colour	clear pale colourless
Shape	prism
Size/mm^3	0.62×0.09×0.06
T/K	140.00(10)
Crystal System	monoclinic
Space Group	$P2_1/c$
a/Å	6.17394(11)
b/Å	8.78352(19)
c/Å	24.0129(5)
α/°	90
β/°	91.6839(18)
γ/°	90
V/Å^3	1301.63(4)
Z	4
Z'	1
Wavelength/Å	1.54184
Radiation type	Cu K□
Θ_{min}/°	3.683
Θ_{max}/°	75.945
Measured Refl's.	6239
Indep't Refl's	2676
Refl's I≥2 σ(I)	2387
R_{int}	0.0262
Parameters	234
Restraints	0
Largest Peak	0.266
Deepest Hole	-0.220
GooF	1.070
wR_2 (all data)	0.1110
wR_2	0.1068
R_1 (all data)	0.0448
R_1	0.0405

Structure Quality Indicators

**Reflections:**	d min (CuKα) 2Θ=151.9° 0.79	I/σ(I) 31.8	Rint m=2.45 2.62%	Full 135.4° 99% to 151.9° 100
**Refinement:**	Shift -0.001	Max Peak 0.3	Min Peak -0.2	GooF 1.070

A clear pale colourless prism-shaped crystal with dimensions 0.62 × 0.09 × 0.06 mm^3 was mounted. Data were collected using a SuperNova, Dual, Cu at home/near, AtlasS2 diffractometer operating at T = 140.00(10) K.

Data were measured using ω scans with Cu K_{α} radiation. The diffraction pattern was indexed and the total number of runs and images was based on the strategy calculation from the program CrysAlisPro system (CCD 43.98a 64-bit (release 24-11-2023)). The maximum resolution that was achieved was Θ = 75.945° (0.79 Å).

The unit cell was refined using CrysAlisPro 1.171.43.98a (Rigaku OD, 2023) on 3470 reflections, 56% of the observed reflections.

Data reduction, scaling and absorption corrections were performed using CrysAlisPro 1.171.43.98a (Rigaku OD, 2023). The final completeness is 100.00 % out to 75.945° in Θ. A gaussian absorption correction was performed using CrysAlisPro 1.171.43.98a (Rigaku Oxford Diffraction, 2023). The numerical absorption correction was based on gaussian integration over a multifaceted crystal model. The empirical absorption correction was done using spherical harmonics, implemented in SCALE3 ABSPACK scaling algorithm. The absorption coefficient μ of this crystal is 0.578 mm^{-1} at this wavelength (λ = 1.54184Å) and the minimum and maximum transmissions are 0.660 and 1.000.

The structure was solved and the space group $P2_1/c$ (# 14) determined by the ShelXT (Sheldrick, 2015) structure solution program using dual methods and refined by full matrix least squares minimisation on $\boldsymbol{F}^2$ using version 2019/3 of ShelXL (Sheldrick, 2015). All non-hydrogen atoms were refined anisotropically. Hydrogen atom positions were calculated geometrically and refined freely.

There is a single formula unit in the asymmetric unit, which is represented by the reported sum formula. In other words: Z is 4 and Z' is 1. The moiety formula is C14 H22 O2.

Data Plots: Diffraction Data

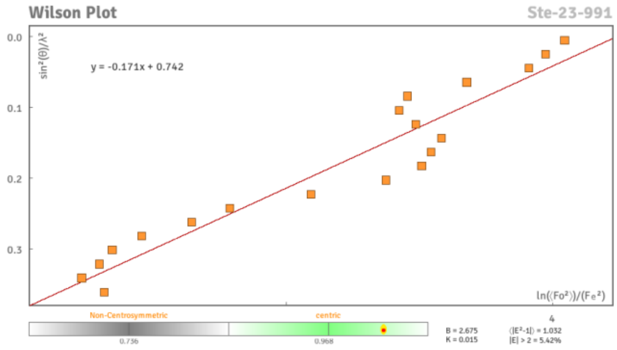

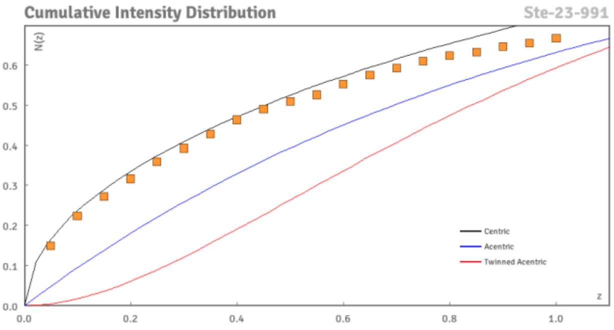

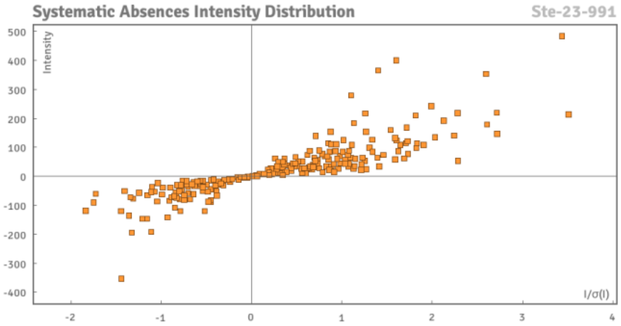

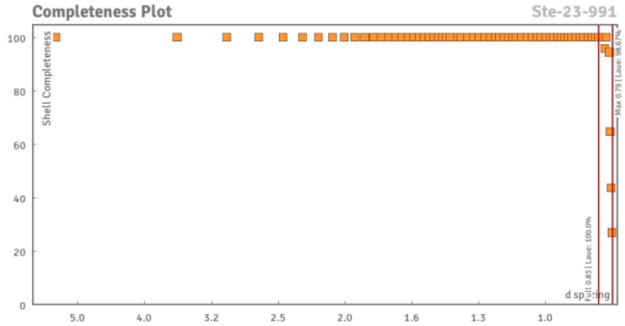

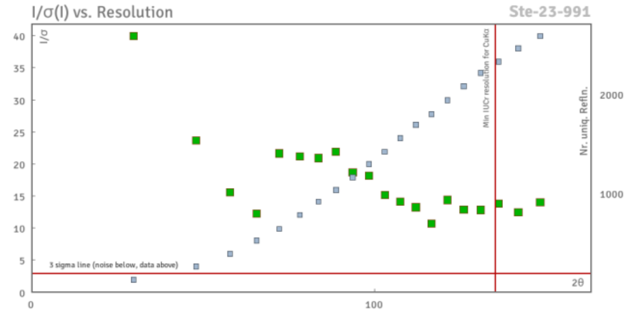

Data Plots: Refinement and Data

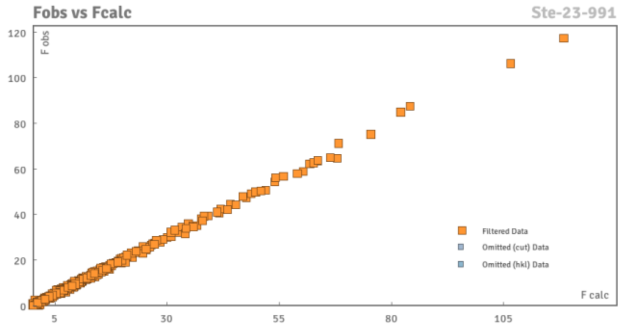

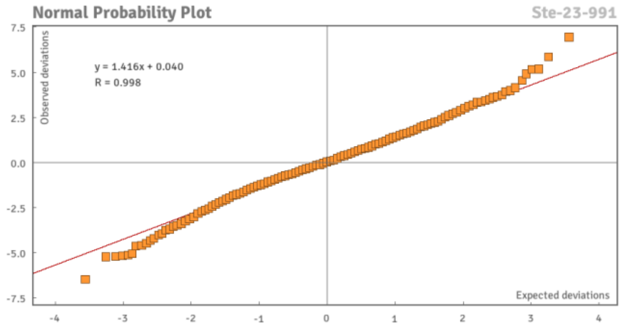

Reflection Statistics

Total reflections (after filtering)	6547	Unique reflections	2676
Completeness	0.987	Mean I/σ	17.39
hkl$_{max}$ collected	(5, 10, 30)	hkl$_{min}$ collected	(-7, -10, -29)
hkl$_{max}$ used	(7, 10, 30)	hkl$_{min}$ used	(-7, 0, 0)
Lim d$_{max}$ collected	100.0	Lim d$_{min}$ collected	0.77
d$_{max}$ used	12.0	d$_{min}$ used	0.79
Friedel pairs	405	Friedel pairs merged	1
Inconsistent equivalents	1	R$_{int}$	0.0262
R$_{sigma}$	0.0314	Intensity transformed	0
Omitted reflections	0	Omitted by user (OMIT hkl)	0
Multiplicity	(3101, 1244, 264, 39, 2)	Maximum multiplicity	8
Removed systematic absences	308	Filtered off (Shel/OMIT)	0

Table 3: Fractional Atomic Coordinates (×10^4) and Equivalent Isotropic Displacement Parameters (Å^2×10^3) for **4pa**. U_{eq} is defined as 1/3 of the trace of the orthogonalised U_{ij}.

Atom	x	y	z	U_{eq}
O1	1694.9(16)	8933.7(11)	7024.4(4)	40.5(2)
O2	6943.8(15)	4316.4(14)	6020.4(5)	52.3(3)
C1	597.2(18)	6858.8(13)	6438.0(5)	29.4(3)
C2	1366.0(18)	7572.0(13)	6984.6(5)	29.3(3)
C3	1716(2)	6530.2(15)	7480.5(5)	33.7(3)
C4	3208(2)	5177.9(14)	7371.5(5)	32.0(3)
C5	2369.8(19)	4016.0(13)	6930.0(5)	29.4(3)
C6	3742.3(16)	4852.9(12)	6489.5(4)	24.7(2)
C7	5082.7(18)	5572.9(14)	6980.8(5)	29.5(3)
C8	2478.0(18)	6059.3(13)	6146.0(5)	27.0(3)
C9	-456(3)	8034.7(17)	6050.3(7)	45.9(4)
C10	5082.0(18)	3936.9(14)	6086.4(5)	29.4(3)
C11	4177.8(18)	2555.7(13)	5759.7(5)	28.5(3)
C12	1714(2)	2406.2(18)	5735.0(8)	46.1(4)
C13	5153(3)	1144.9(16)	6044.8(7)	46.1(4)
C14	5013(3)	2652(2)	5165.6(6)	50.4(4)

Table 4: Anisotropic Displacement Parameters (×10[4]) for **4pa**. The anisotropic displacement factor exponent takes the form: $-2\pi^2[h^2a^{*2} \times U_{11} + ... +2hka^* \times b^* \times U_{12}]$

Atom	U_{11}	U_{22}	U_{33}	U_{23}	U_{13}	U_{12}
O1	48.0(5)	27.4(5)	46.1(5)	-7.1(4)	-0.5(4)	-1.3(4)
O2	27.8(5)	61.0(7)	68.7(7)	-32.2(6)	15.1(4)	-10.8(4)
C1	25.5(5)	26.0(6)	36.5(6)	-1.9(5)	-3.1(4)	1.5(4)
C2	24.2(5)	27.9(6)	36.1(6)	-4.7(4)	4.2(4)	2.2(4)
C3	36.7(6)	33.5(6)	31.1(6)	-4.3(5)	6.9(5)	1.0(5)
C4	38.0(6)	31.8(6)	26.3(5)	0.8(4)	2.3(4)	4.1(5)
C5	32.3(6)	25.2(6)	31.0(6)	1.4(4)	6.9(4)	0.7(4)
C6	22.7(5)	23.9(5)	27.4(5)	-1.5(4)	1.8(4)	-0.7(4)
C7	26.4(5)	31.1(6)	30.8(6)	-3.7(5)	-2.4(4)	2.4(4)
C8	28.7(5)	25.6(5)	26.6(5)	-0.3(4)	0.3(4)	-0.5(4)
C9	48.4(8)	35.9(7)	52.4(8)	-0.7(6)	-16.0(6)	11.2(6)
C10	24.4(5)	29.7(6)	34.3(6)	-4.8(5)	3.2(4)	0.0(4)
C11	27.5(5)	26.8(6)	31.1(6)	-4.9(4)	0.8(4)	1.2(4)
C12	30.2(6)	40.7(8)	67.2(10)	-20.9(7)	-2.7(6)	-3.2(5)
C13	51.7(8)	30.5(7)	55.2(9)	0.1(6)	-12.1(7)	3.5(6)
C14	61.4(10)	57.8(10)	32.1(7)	-8.2(6)	5.0(6)	-16.6(8)

Table 5: Bond Lengths in Å for **4pa**.

Atom	Atom	Length/Å
O1	C2	1.2164(15)
O2	C10	1.2117(15)

Atom	Atom	Length/Å
C1	C2	1.5177(16)
C1	C8	1.5431(16)
C1	C9	1.5234(17)
C2	C3	1.5122(17)
C3	C4	1.5303(17)
C4	C5	1.5495(16)
C4	C7	1.5505(17)
C5	C6	1.5588(15)
C6	C7	1.5554(14)
C6	C8	1.5410(15)
C6	C10	1.5217(15)
C10	C11	1.5404(15)
C11	C12	1.5265(17)
C11	C13	1.5305(18)
C11	C14	1.5335(19)

Table 6: Bond Angles in ° for **4pa**.

Atom	Atom	Atom	Angle/°
C2	C1	C8	111.12(9)
C2	C1	C9	111.45(10)
C9	C1	C8	110.13(11)
O1	C2	C1	121.40(11)
O1	C2	C3	120.89(11)
C3	C2	C1	117.71(10)
C2	C3	C4	114.07(10)
C3	C4	C5	115.96(10)
C3	C4	C7	113.17(10)
C5	C4	C7	88.49(9)
C4	C5	C6	88.65(9)
C7	C6	C5	87.98(8)
C8	C6	C5	114.24(9)
C8	C6	C7	112.10(9)
C10	C6	C5	119.92(10)
C10	C6	C7	114.23(9)
C10	C6	C8	107.42(9)
C4	C7	C6	88.74(8)
C6	C8	C1	116.48(9)
O2	C10	C6	118.25(11)
O2	C10	C11	118.75(10)
C6	C10	C11	123.00(9)
C12	C11	C10	115.68(10)
C12	C11	C13	109.04(12)

Atom	Atom	Atom	Angle/°
C12	C11	C14	109.30(12)
C13	C11	C10	106.14(10)
C13	C11	C14	108.80(12)
C14	C11	C10	107.67(10)

Table 7: Torsion Angles in ° for **4pa**.

Atom	Atom	Atom	Atom	Angle/°
O1	C2	C3	C4	-126.61(13)
O2	C10	C11	C12	-163.70(14)
O2	C10	C11	C13	75.23(16)
O2	C10	C11	C14	-41.15(17)
C1	C2	C3	C4	53.28(15)
C2	C1	C8	C6	54.88(13)
C2	C3	C4	C5	-64.31(14)
C2	C3	C4	C7	35.85(14)
C3	C4	C5	C6	96.53(11)
C3	C4	C7	C6	-99.05(10)
C4	C5	C6	C7	18.56(8)
C4	C5	C6	C8	-94.82(10)
C4	C5	C6	C10	135.54(10)
C5	C4	C7	C6	18.66(8)
C5	C6	C7	C4	-18.55(8)
C5	C6	C8	C1	29.82(13)
C5	C6	C10	O2	-132.84(13)
C5	C6	C10	C11	47.22(15)
C6	C10	C11	C12	16.24(17)
C6	C10	C11	C13	-104.83(13)
C6	C10	C11	C14	138.79(13)
C7	C4	C5	C6	-18.62(8)
C7	C6	C8	C1	-68.29(12)
C7	C6	C10	O2	-30.45(17)
C7	C6	C10	C11	149.61(11)
C8	C1	C2	O1	102.28(13)
C8	C1	C2	C3	-77.61(13)
C8	C6	C7	C4	96.86(10)
C8	C6	C10	O2	94.55(14)
C8	C6	C10	C11	-85.39(13)
C9	C1	C2	O1	-20.96(17)
C9	C1	C2	C3	159.15(12)
C9	C1	C8	C6	178.88(10)
C10	C6	C7	C4	-140.66(10)
C10	C6	C8	C1	165.43(9)

Table 8: Hydrogen Fractional Atomic Coordinates (×10^4) and Equivalent Isotropic Displacement Parameters (Å^2×10^3) for **4pa**. U_{eq} is defined as 1/3 of the trace of the orthogonalised U_{ij}.

Atom	x	y	z	U_{eq}
H1	-490(20)	6082(18)	6527(6)	32(4)
H3A	2320(30)	7155(19)	7788(7)	36(4)
H3B	300(30)	6160(20)	7584(7)	49(5)
H4	3690(30)	4730(20)	7735(7)	42(4)
H5A	2950(30)	2990(20)	7009(7)	37(4)
H5B	800(20)	3932(17)	6860(6)	29(3)
H7A	5460(30)	6679(19)	6949(7)	38(4)
H7B	6400(30)	4962(18)	7065(6)	35(4)
H8A	1870(20)	5596(17)	5802(6)	27(3)
H8B	3560(30)	6844(19)	6030(7)	38(4)
H9A	-1700(40)	8590(30)	6235(9)	67(6)
H9B	620(30)	8840(30)	5957(9)	63(6)
H9C	-1000(40)	7550(20)	5712(9)	65(6)
H12A	1300(30)	1500(20)	5519(7)	41(4)
H12B	1120(40)	2310(20)	6095(9)	64(6)
H12C	1020(40)	3310(30)	5555(10)	82(7)
H13A	4740(30)	230(20)	5819(9)	61(5)
H13B	6760(40)	1230(30)	6091(9)	69(6)
H13C	4620(40)	990(30)	6432(11)	81(7)
H14A	4530(30)	1760(20)	4956(9)	61(6)
H14B	4370(40)	3590(30)	4988(10)	74(7)
H14C	6640(40)	2740(30)	5172(9)	74(7)

6-(2-Naphthoyl)-2,4-trans-dimethylbicyclo[4.1.1]octan-3-one (4ad)

CCDC deposition Number 2333992

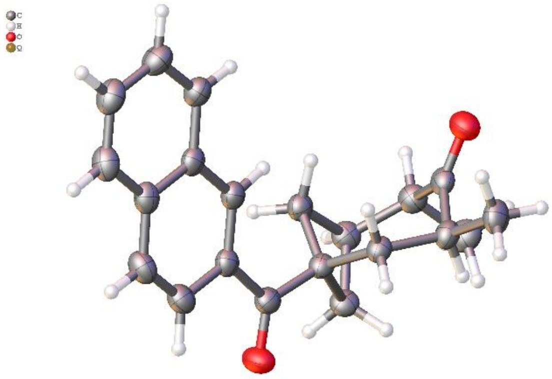

Crystals of compound **4ad** were collected upon spontaneous crystallization out of deuterated chloroform at room temperature.

Experimental. Single clear pale colourless prism-shaped crystals of **4ad** were used as supplied. A suitable crystal with dimensions 0.21 × 0.19 × 0.13 mm^3 was selected and mounted on a XtaLAB Synergy R, DW system, HyPix-Arc 150 diffractometer. The crystal was kept at a steady T = 140.00(10) K during data collection. The structure was solved with the ShelXT (Sheldrick, 2015) solution program using dual methods and by using Olex2 1.5 (Dolomanov et al., 2009) as the graphical interface. The model was refined with ShelXL 2019/3 (Sheldrick, 2015) using full matrix least squares minimisation on $\boldsymbol{F^2}$.

Crystal Data. $C_{21}H_{22}O_2$, M_r = 306.38, triclinic, P-1 (No. 2), a = 8.11476(18) Å, b = 10.4720(2) Å, c = 10.7595(4) Å, α = 67.728(3)°, β = 74.021(2)°, γ = 82.9324(19)°, V = 813.23(4) Å^3, T = 140.00(10) K, Z = 2, Z' = 1, μ(Cu K_α) = 0.617, 17602 reflections measured, 3274 unique (R_{int} = 0.0196) which were used in all calculations. The final wR_2 was 0.1140 (all data) and R_1 was 0.0409 (I≥2 σ(I)).

Compound 4ad

Formula	$C_{21}H_{22}O_2$
$D_{calc.}$/ g cm^{-3}	1.251
μ/mm^{-1}	0.617
Formula Weight	306.38
Colour	clear pale colourless
Shape	prism
Size/mm^3	0.21×0.19×0.13
T/K	140.00(10)
Crystal System	triclinic
Space Group	*P*-1
a/Å	8.11476(18)
b/Å	10.4720(2)
c/Å	10.7595(4)
α/°	67.728(3)
β/°	74.021(2)
γ/°	82.9324(19)
V/Å^3	813.23(4)
Z	2
Z'	1
Wavelength/Å	1.54184
Radiation type	Cu K□
Θ_{min}/°	4.564
Θ_{max}/°	75.563
Measured Refl's.	17602
Indep't Refl's	3274
Refl's I≥2 □(I)	2901
R_{int}	0.0196
Parameters	297
Restraints	0
Largest Peak	0.288
Deepest Hole	-0.199
GooF	1.070
wR_2 (all data)	0.1140
wR_2	0.1107
R_1 (all data)	0.0455
R_1	0.0409

Structure Quality Indicators

**Reflections:**	d min (CuKα) 2Θ=151.1° 0.80	I/σ(I) 72.0	Rint m=5.38 1.96%	Full 135.4° 97% to 151.1° 99.5
**Refinement:**	Shift 0.000	Max Peak 0.3	Min Peak -0.2	GooF 1.070

A clear pale colourless prism-shaped crystal with dimensions 0.21 × 0.19 × 0.13 mm^3 was mounted. Data were collected using a XtaLAB Synergy R, DW system, HyPix-Arc 150 diffractometer operating at *T* = 140.00(10) K.

Data were measured using ω scans with Cu K_α radiation. The diffraction pattern was indexed and the total number of runs and images was based on the strategy calculation from the program CrysAlisPro system (CCD 43.105a 64-bit (release 11-01-2024)). The maximum resolution that was achieved was Θ = 75.563° (0.80 Å).

The unit cell was refined using CrysAlisPro 1.171.43.105a (Rigaku OD, 2024) on 11017 reflections, 63% of the observed reflections.

Data reduction, scaling and absorption corrections were performed using CrysAlisPro 1.171.43.105a (Rigaku OD, 2024). The final completeness is 99.50 % out to 75.563° in □. A gaussian absorption correction was performed using CrysAlisPro 1.171.43.105a (Rigaku Oxford Diffraction, 2024). The numerical absorption correction was based on gaussian integration over a multifaceted crystal model. The empirical absorption correction was done using spherical harmonics, implemented in SCALE3 ABSPACK scaling algorithm. The absorption coefficient μ of this crystal is 0.617 mm^{-1} at this wavelength (λ = 1.54184Å) and the minimum and maximum transmissions are 0.637 and 1.000.

The structure was solved and the space group *P*-1 (# 2) determined by the ShelXT (Sheldrick, 2015) structure solution program using dual methods and refined by full matrix least squares minimisation on $\boldsymbol{F^2}$ using version 2019/3 of ShelXL 2019/3 (Sheldrick, 2015). All non-hydrogen atoms were refined anisotropically. Hydrogen atom positions were calculated geometrically and refined freely.

There is a single formula unit in the asymmetric unit, which is represented by the reported sum formula. In other words: Z is 2 and Z' is 1. The moiety formula is C21 H22 O2.

Data Plots: Diffraction Data

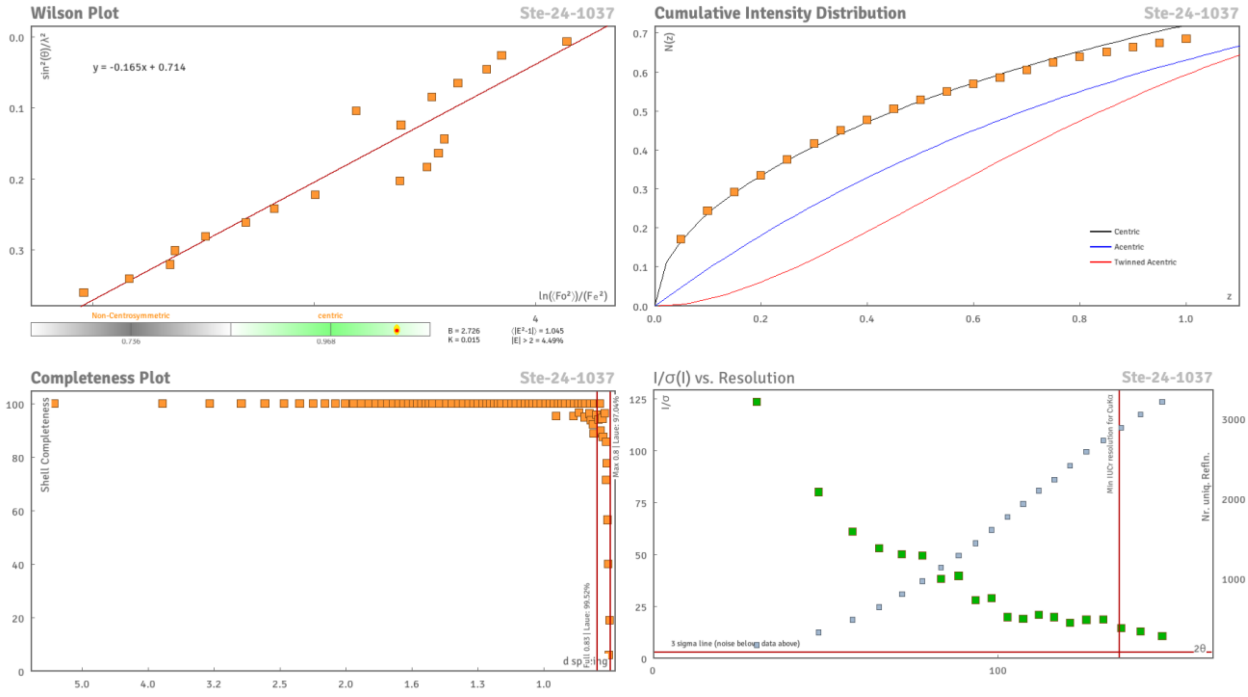

Data Plots: Refinement and Data

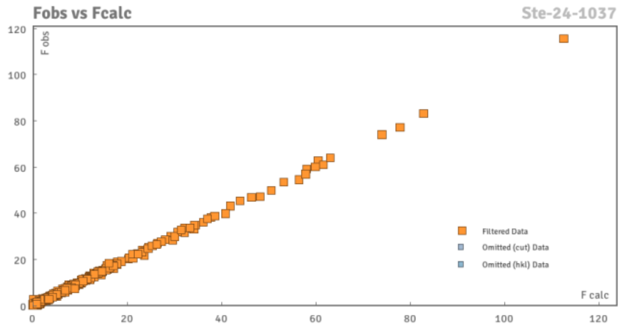

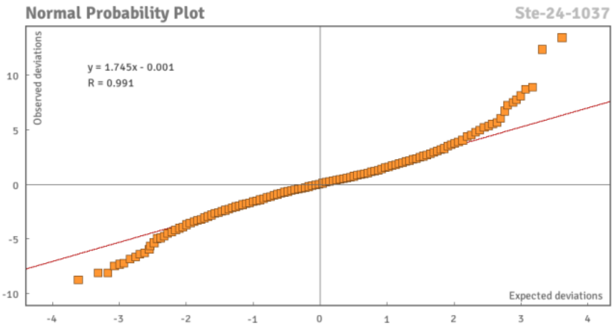

Reflection Statistics

Total reflections (after filtering)	17602	Unique reflections	3274
Completeness	0.97	Mean I/σ	36.86
hkl_{max} collected	(9, 13, 13)	hkl_{min} collected	(-10, -12, -13)
hkl_{max} used	(10, 13, 13)	hkl_{min} used	(-9, -11, 0)
Lim d_{max} collected	100.0	Lim d_{min} collected	0.77
d_{max} used	9.69	d_{min} used	0.8
Friedel pairs	2131	Friedel pairs merged	1
Inconsistent equivalents	1	R_{int}	0.0196
R_{sigma}	0.0139	Intensity transformed	0
Omitted reflections	0	Omitted by user (OMIT hkl)	0
Multiplicity	(1397, 1495, 1019, 541, 299, 164, 98, 76, 67, 38, 35, 25, 26, 21, 9, 20, 15, 11, 16, 8, 7, 12, 6)	Maximum multiplicity	23
Removed systematic absences	0	Filtered off (Shel/OMIT)	0

Table 9: Fractional Atomic Coordinates (×10^4) and Equivalent Isotropic Displacement Parameters (Å^2×10^3) for **4ad**. U_{eq} is defined as 1/3 of the trace of the orthogonalised U_{ij}.

Atom	x	y	z	U_{eq}
O1	3750.8(14)	300.2(10)	3570.8(11)	46.1(3)
O2	-1378.3(13)	3996.1(10)	6597.8(11)	44.4(3)
C1	3586.1(17)	1426.4(13)	3691.8(14)	32.7(3)
C2	2196.3(16)	1659.4(12)	4863.6(13)	29.2(3)
C3	1501.4(19)	331.4(13)	6103.0(14)	34.2(3)
C4	1487.1(18)	1079.6(13)	7101.4(14)	34.7(3)
C5	-190.8(18)	1782.0(14)	7621.2(14)	36.5(3)
C6	-877.1(16)	2896.5(13)	6476.2(14)	32.7(3)
C7	-998.9(16)	2578.3(13)	5243.7(14)	32.9(3)
C8	771.2(16)	2578.8(12)	4241.9(13)	29.6(3)
C9	2760.8(17)	2117.9(14)	5906.6(14)	32.8(3)
C10	-1606(2)	752.3(17)	8550.9(16)	45.6(4)
C11	-2186.2(19)	3586.1(17)	4434.8(17)	42.4(3)
C12	4720.3(15)	2581.8(13)	2656.1(13)	30.6(3)
C13	4648.6(16)	3859.3(13)	2751.1(13)	30.6(3)
C14	5763.9(16)	4923.9(13)	1750.3(13)	30.6(3)
C15	5730.8(18)	6231.6(14)	1853.4(14)	35.8(3)

Atom	x	y	z	U_{eq}
C16	6840.6(19)	7233.0(15)	886.1(15)	40.9(3)
C17	8032.6(19)	6958.1(16)	-219.2(15)	43.2(4)
C18	8087.3(19)	5712.0(16)	-351.9(15)	41.0(3)
C19	6953.9(17)	4651.6(14)	633.1(13)	34.2(3)
C20	6977.4(19)	3336.1(15)	540.2(15)	39.9(3)
C21	5902.2(18)	2331.8(14)	1516.4(14)	37.0(3)

Table 10: Anisotropic Displacement Parameters (×10[4]) for **4ad**. The anisotropic displacement factor exponent takes the form: $-2\pi^2[h^2a^{*2} \times U_{11} + ... +2hka^* \times b^* \times U_{12}]$

Atom	U_{11}	U_{22}	U_{33}	U_{23}	U_{13}	U_{12}
O1	48.1(6)	33.0(5)	54.5(6)	-20.2(5)	-1.4(5)	-2.9(4)
O2	42.1(6)	35.9(5)	52.3(6)	-17.5(5)	-5.8(5)	1.3(4)
C1	31.5(7)	29.1(6)	37.4(7)	-10.4(5)	-11.5(5)	1.6(5)
C2	29.7(6)	23.9(6)	31.2(6)	-5.8(5)	-8.8(5)	-1.3(5)
C3	40.0(7)	24.3(6)	33.7(7)	-3.9(5)	-11.9(6)	0.4(5)
C4	39.1(7)	31.3(6)	30.8(6)	-5.2(5)	-13.6(6)	1.8(5)
C5	38.8(7)	35.5(7)	32.1(7)	-8.9(5)	-7.6(6)	-3.5(6)
C6	25.7(6)	29.5(6)	36.9(7)	-7.7(5)	-2.7(5)	-4.4(5)
C7	28.0(6)	30.7(6)	35.3(7)	-5.4(5)	-9.1(5)	-2.8(5)
C8	29.1(6)	25.5(6)	30.5(6)	-4.1(5)	-9.4(5)	-2.4(5)
C9	29.9(7)	32.9(7)	36.2(7)	-11.4(5)	-11.8(5)	1.6(5)
C10	50.9(9)	42.1(8)	35.4(8)	-5.7(6)	-5.2(7)	-11.4(7)
C11	31.6(8)	45.2(8)	45.0(8)	-8.2(7)	-14.4(6)	3.1(6)
C12	25.8(6)	32.2(6)	32.3(6)	-8.6(5)	-10.5(5)	2.5(5)
C13	26.4(6)	34.0(6)	30.4(6)	-9.3(5)	-8.8(5)	-0.3(5)
C14	25.9(6)	33.6(6)	30.9(6)	-6.7(5)	-12.1(5)	0.1(5)
C15	34.0(7)	37.9(7)	34.0(7)	-10.0(6)	-8.8(6)	-4.8(5)
C16	41.9(8)	37.6(7)	40.1(7)	-6.9(6)	-12.0(6)	-9.3(6)
C17	38.1(8)	44.3(8)	36.3(7)	-0.9(6)	-8.5(6)	-8.5(6)
C18	34.7(7)	44.7(8)	31.6(7)	-3.8(6)	-4.4(6)	0.2(6)
C19	29.3(6)	38.8(7)	29.6(6)	-5.7(5)	-10.4(5)	2.4(5)
C20	37.7(7)	41.8(7)	33.1(7)	-11.1(6)	-4.7(6)	6.6(6)
C21	36.5(7)	35.3(7)	36.9(7)	-11.9(6)	-10.2(6)	6.0(5)

Table 11: Bond Lengths in Å for **4ad**.

Atom	Atom	Length/Å
O1	C1	1.2223(16)
O2	C6	1.2158(16)
C1	C2	1.5177(18)
C1	C12	1.4967(18)
C2	C3	1.5504(17)
C2	C8	1.5412(17)

Atom	Atom	Length/Å
C2	C9	1.5638(18)
C3	C4	1.5482(19)
C4	C5	1.5342(19)
C4	C9	1.5456(18)
C5	C6	1.5224(18)
C5	C10	1.529(2)
C6	C7	1.5148(19)
C7	C8	1.5402(18)
C7	C11	1.5215(18)
C12	C13	1.3728(18)
C12	C21	1.4234(19)
C13	C14	1.4224(18)
C14	C15	1.4116(19)
C14	C19	1.4183(19)
C15	C16	1.3722(19)
C16	C17	1.411(2)
C17	C18	1.360(2)
C18	C19	1.4230(19)
C19	C20	1.416(2)
C20	C21	1.362(2)

Table 12: Bond Angles in ° for **4ad**.

Atom	Atom	Atom	Angle/°
O1	C1	C2	119.87(12)
O1	C1	C12	119.76(12)
C12	C1	C2	120.34(11)
C1	C2	C3	115.23(10)
C1	C2	C8	108.24(10)
C1	C2	C9	117.78(10)
C3	C2	C9	87.54(9)
C8	C2	C3	112.89(10)
C8	C2	C9	114.15(10)
C4	C3	C2	88.95(9)
C5	C4	C3	118.18(12)
C5	C4	C9	112.36(10)
C9	C4	C3	88.27(10)
C6	C5	C4	114.32(11)
C6	C5	C10	109.06(12)
C10	C5	C4	112.71(12)
O2	C6	C5	119.45(12)
O2	C6	C7	121.59(12)
C7	C6	C5	118.89(11)

Atom	Atom	Atom	Angle/°
C6	C7	C8	111.87(10)
C6	C7	C11	112.43(12)
C11	C7	C8	108.62(11)
C7	C8	C2	117.18(10)
C4	C9	C2	88.55(9)
C13	C12	C1	123.02(12)
C13	C12	C21	119.32(12)
C21	C12	C1	117.66(12)
C12	C13	C14	121.14(12)
C15	C14	C13	121.71(12)
C15	C14	C19	119.43(12)
C19	C14	C13	118.86(12)
C16	C15	C14	120.52(13)
C15	C16	C17	120.03(14)
C18	C17	C16	120.82(13)
C17	C18	C19	120.56(14)
C14	C19	C18	118.64(13)
C20	C19	C14	118.97(12)
C20	C19	C18	122.39(13)
C21	C20	C19	121.01(13)
C20	C21	C12	120.68(13)

Table 14: Torsion Angles in ° for **4ad**.

Atom	Atom	Atom	Atom	Angle/°
O1	C1	C2	C3	20.29(18)
O1	C1	C2	C8	-107.15(13)
O1	C1	C2	C9	121.52(14)
O1	C1	C12	C13	-177.75(12)
O1	C1	C12	C21	3.19(19)
O2	C6	C7	C8	107.85(14)
O2	C6	C7	C11	-14.70(17)
C1	C2	C3	C4	139.01(11)
C1	C2	C8	C7	161.02(11)
C1	C2	C9	C4	-136.71(11)
C1	C12	C13	C14	179.35(11)
C1	C12	C21	C20	-179.68(12)
C2	C1	C12	C13	4.22(19)
C2	C1	C12	C21	-174.84(11)
C2	C3	C4	C5	95.02(12)
C2	C3	C4	C9	-19.53(10)
C3	C2	C8	C7	32.25(15)
C3	C2	C9	C4	-19.34(9)

Atom	Atom	Atom	Atom	Angle/°
C3	C4	C5	C6	-59.27(16)
C3	C4	C5	C10	66.01(15)
C3	C4	C9	C2	19.36(9)
C4	C5	C6	O2	-134.40(13)
C4	C5	C6	C7	48.60(16)
C5	C4	C9	C2	-100.54(12)
C5	C6	C7	C8	-75.22(14)
C5	C6	C7	C11	162.24(12)
C6	C7	C8	C2	53.21(15)
C8	C2	C3	C4	-95.93(12)
C8	C2	C9	C4	94.70(11)
C9	C2	C3	C4	19.31(10)
C9	C2	C8	C7	-65.70(14)
C9	C4	C5	C6	41.30(16)
C9	C4	C5	C10	166.57(12)
C10	C5	C6	O2	98.43(15)
C10	C5	C6	C7	-78.58(15)
C11	C7	C8	C2	177.90(11)
C12	C1	C2	C3	-161.68(11)
C12	C1	C2	C8	70.88(14)
C12	C1	C2	C9	-60.45(15)
C12	C13	C14	C15	-178.50(12)
C12	C13	C14	C19	0.58(18)
C13	C12	C21	C20	1.2(2)
C13	C14	C15	C16	178.91(12)
C13	C14	C19	C18	-178.91(12)
C13	C14	C19	C20	0.83(18)
C14	C15	C16	C17	-0.3(2)
C14	C19	C20	C21	-1.2(2)
C15	C14	C19	C18	0.20(18)
C15	C14	C19	C20	179.94(12)
C15	C16	C17	C18	0.8(2)
C16	C17	C18	C19	-0.7(2)
C17	C18	C19	C14	0.3(2)
C17	C18	C19	C20	-179.47(13)
C18	C19	C20	C21	178.51(13)
C19	C14	C15	C16	-0.2(2)
C19	C20	C21	C12	0.2(2)
C21	C12	C13	C14	-1.61(19)

Table 15: Hydrogen Fractional Atomic Coordinates (×10^4) and Equivalent Isotropic Displacement Parameters (Å^2×10^3) for **4ad**. U_{eq} is defined as 1/3 of the trace of the orthogonalised U_{ij}.

Atom	x	y	z	U_{eq}
H3A	2370(20)	-409(16)	6175(16)	37(4)
H3B	400(20)	-24(16)	6119(16)	39(4)
H4	1922(19)	511(16)	7910(16)	35(4)
H5	20(20)	2287(16)	8236(16)	38(4)
H7	-1450(20)	1630(17)	5611(16)	39(4)
H8A	1130(20)	3554(16)	3777(16)	38(4)
H8B	580(20)	2284(16)	3526(17)	37(4)
H9A	2550(20)	3116(17)	5817(16)	38(4)
H9B	3940(20)	1819(15)	5939(16)	37(4)
H10A	-1970(20)	249(18)	7985(19)	54(5)
H10B	-2690(20)	1272(19)	8911(19)	57(5)
H10C	-1150(20)	70(20)	9350(20)	62(5)
H11A	-1750(20)	4520(20)	4049(19)	54(5)
H11B	-3350(30)	3627(19)	5000(20)	58(5)
H11C	-2290(20)	3309(19)	3680(20)	55(5)
H13	3879(18)	4093(14)	3493(15)	29(3)
H15	4890(20)	6433(15)	2594(17)	37(4)
H16	6780(20)	8160(18)	959(17)	47(4)
H17	8850(20)	7696(19)	-930(20)	57(5)
H18	8910(20)	5482(17)	-1086(18)	45(4)
H20	7790(20)	3181(18)	-248(19)	51(5)
H21	5910(20)	1405(19)	1424(19)	55(5)

6-(2-Naphthoyl)-4,5-cis-dimethylbicyclo[4.1.1]octan-3-one (4ae)
CCDC deposition Number 2356953

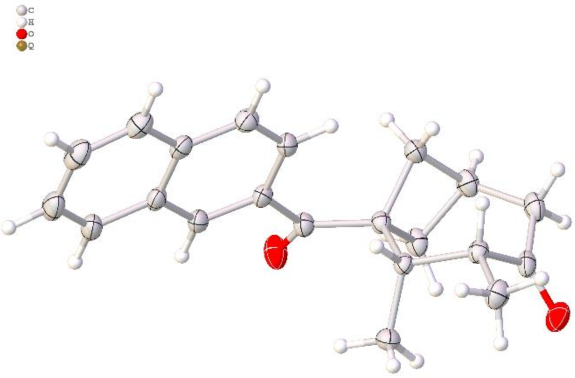

Crystals of compound **4ae** were collected upon spontaneous crystallization out of deuterated chloroform at room temperature.

Experimental. Single clear pale colourless prism-shaped crystals of **4ae** were used as supplied. A suitable crystal with dimensions 0.67 × 0.23 × 0.10 mm^3 was selected and mounted on a SuperNova, Dual, Cu at home/near, AtlasS2 diffractometer. The crystal was kept at a steady T = 140.00(10) K during data collection. The structure was solved with the ShelXT (Sheldrick, 2015) solution program using dual methods and by using Olex2 1.5 (Dolomanov et al., 2009) as the graphical interface. The model was refined with ShelXL 2019/3 (Sheldrick, 2015) using full matrix least squares minimisation on $\boldsymbol{F}^2$.

Crystal Data. $C_{21}H_{22}O_2$, M_r = 306.38, monoclinic, $P2_1/c$ (No. 14), a = 17.7615(2) Å, b = 6.40467(8) Å, c = 13.86477(15) Å, β = 95.9318(11)°, $\alpha = \gamma$ = 90°, V = 1568.76(3) Å^3, T = 140.00(10) K, Z = 4, Z' = 1, μ(Cu K_α) = 0.640, 7738 reflections measured, 3208 unique (R_{int} = 0.0228) which were used in all calculations. The final wR_2 was 0.1104 (all data) and R_1 was 0.0405 (I≥2 σ(I)).

**Compound**	**4ae**
Formula	$C_{21}H_{22}O_2$
$D_{calc.}$/ g cm^{-3}	1.297
μ/mm^{-1}	0.640
Formula Weight	306.38
Colour	clear pale colourless
Shape	prism
Size/mm^3	0.67×0.23×0.10
T/K	140.00(10)
Crystal System	monoclinic
Space Group	$P2_1/c$
a/Å	17.7615(2)
b/Å	6.40467(8)
c/Å	13.86477(15)
α/°	90
β/°	95.9318(11)
γ/°	90
V/Å^3	1568.76(3)
Z	4
Z'	1
Wavelength/Å	1.54184
Radiation type	Cu K□
Θ_{min}/°	5.007
Θ_{max}/°	76.026
Measured Refl's.	7738
Indep't Refl's	3208
Refl's I≥2 □(I)	2925
R_{int}	0.0228
Parameters	297
Restraints	0
Largest Peak	0.271
Deepest Hole	-0.203
GooF	1.042
wR_2 (all data)	0.1104
wR_2	0.1060
R_1 (all data)	0.0442
R_1	0.0405

Structure Quality Indicators

**Reflections:**	d min (CuKα) 2Θ=152.1° 0.79	I/σ(I) 36.3	Rint m=2.55 2.28%	Full 135.4° 98% to 152.1° 99.9
**Refinement:**	Shift 0.001	Max Peak 0.3	Min Peak -0.2	GooF 1.042

A clear pale colourless prism-shaped crystal with dimensions 0.67 × 0.23 × 0.10 mm^3 was mounted. Data were collected using a SuperNova, Dual, Cu at home/near, AtlasS2 diffractometer operating at *T* = 140.00(10) K.

Data were measured using ω scans with Cu K_α radiation. The diffraction pattern was indexed and the total number of runs and images was based on the strategy calculation from the program CrysAlisPro system (CCD 43.123a 64-bit (release 20-05-2024)). The maximum resolution that was achieved was Θ = 76.026° (0.79 Å).

The unit cell was refined using CrysAlisPro 1.171.43.123a (Rigaku OD, 2024) on 4923 reflections, 64% of the observed reflections.

Data reduction, scaling and absorption corrections were performed using CrysAlisPro 1.171.43.123a (Rigaku OD, 2024). The final completeness is 99.90 % out to 76.026° in Θ. A

gaussian absorption correction was performed using CrysAlisPro 1.171.43.123a (Rigaku Oxford Diffraction, 2024). The numerical absorption correction was based on gaussian integration over a multifaceted crystal model. The empirical absorption correction was done using spherical harmonics, implemented in SCALE3 ABSPACK scaling algorithm. The absorption coefficient μ of this crystal is 0.640 mm^{-1} at this wavelength (λ = 1.54184Å) and the minimum and maximum transmissions are 0.643 and 1.000.

The structure was solved and the space group $P2_1/c$ (# 14) determined by the ShelXT (Sheldrick, 2015) structure solution program using dual methods and refined by full matrix least squares minimisation on $\boldsymbol{F}^2$ using version 2019/3 of ShelXL (Sheldrick, 2015). All non-hydrogen atoms were refined anisotropically. Hydrogen atom positions were calculated geometrically and refined using the riding model.

There is a single formula unit in the asymmetric unit, which is represented by the reported sum formula. In other words: Z is 4 and Z' is 1. The moiety formula is C21 H22 O2.

Data Plots: Diffraction Data

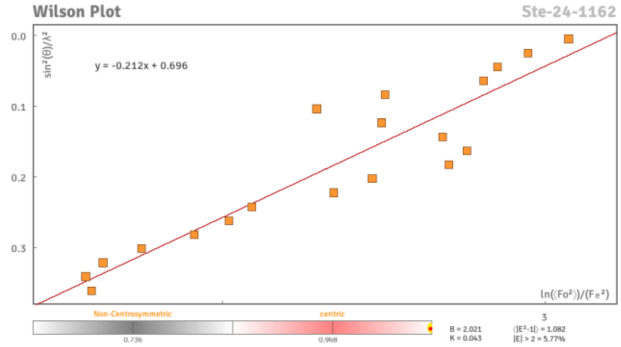

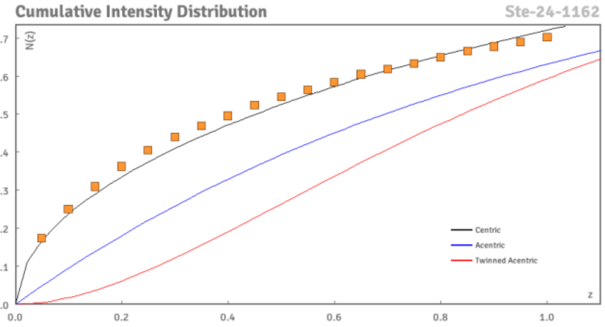

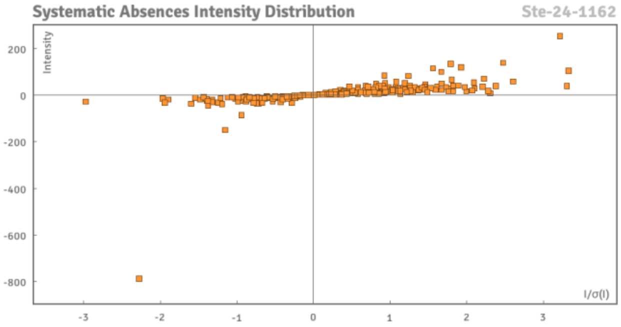

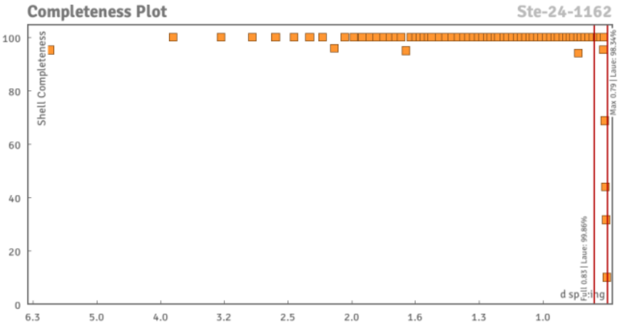

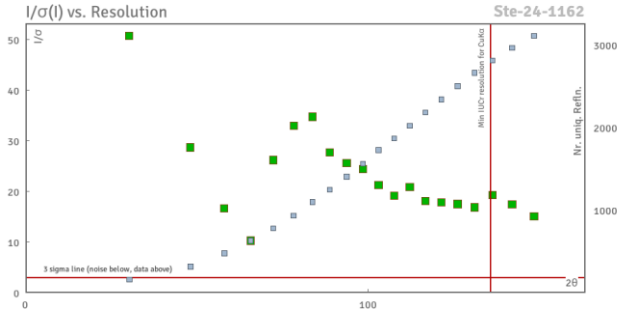

Data Plots: Refinement and Data

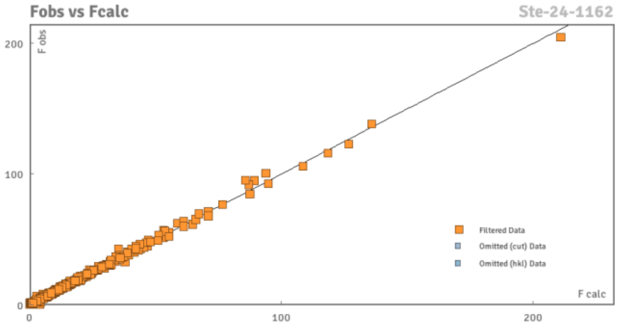

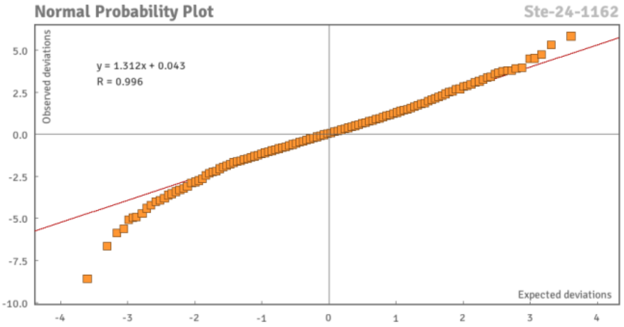

Reflection Statistics

Total reflections (after filtering)	8182
Completeness	0.983
hkl_{max} collected	(22, 4, 15)
hkl_{max} used	(22, 7, 17)
Lim d_{max} collected	100.0
d_{max} used	13.79
Friedel pairs	482
Inconsistent equivalents	5
R_{sigma}	0.0275
Omitted reflections	0
Multiplicity	(3486, 1526, 423, 80, 11)
Removed systematic absences	444
Unique reflections	3208
Mean I/σ	23.2
hklmin collected	(-21, -7, -17)
hklmin used	(-22, 0, 0)
Lim dmin collected	0.77
dmin used	0.79
Friedel pairs merged	1
Rint	0.0228
Intensity transformed	0
Omitted by user (OMIT hkl)	0
Maximum multiplicity	8
Filtered off (Shel/OMIT)	0

Table 16: Fractional Atomic Coordinates ($\times 10^4$) and Equivalent Isotropic Displacement Parameters (Å$^2\times 10^3$) for **4ae**. U_{eq} is defined as 1/3 of the trace of the orthogonalised U_{ij}.

Atom	x	y	z	U_{eq}
O1	9417.9(5)	4200.3(15)	8141.9(6)	34.2(2)
O2	7533.1(5)	-349.5(14)	5097.3(7)	34.0(2)
C1	9235.7(6)	4673.2(17)	7301.1(8)	24.6(2)
C2	9766.0(6)	4360.9(19)	6527.6(9)	28.7(3)
C3	9433.2(6)	3048.7(19)	5662.8(9)	27.3(3)
C4	8911.6(6)	1274.6(18)	5959.1(9)	25.9(2)
C5	8212.5(6)	2694.3(16)	5665.2(7)	20.3(2)
C6	7910.5(5)	3916.1(16)	6513.0(7)	19.9(2)

Atom	x	y	z	U_{eq}
C7	8462.1(6)	5609.9(17)	6980.0(7)	22.1(2)
C8	8736.9(6)	3996.4(18)	5046.1(8)	25.0(2)
C9	7656.3(6)	2394.5(19)	7267.7(8)	26.6(2)
C10	8131.3(7)	6732(2)	7812.0(9)	30.4(3)
C11	7548.5(6)	1547.1(17)	5117.5(7)	22.4(2)
C12	6888.0(6)	2771.8(17)	4642.6(7)	21.1(2)
C13	6945.8(6)	4882.0(17)	4346.4(7)	23.0(2)
C14	6324.2(6)	5910.2(18)	3915.5(8)	25.1(2)
C15	5603.8(6)	4931.6(19)	3790.6(7)	24.1(2)
C16	4942.6(7)	5991(2)	3384.5(8)	30.3(3)
C17	4252.9(7)	5015(2)	3315.2(9)	34.9(3)
C18	4186.3(7)	2939(2)	3625.8(9)	34.7(3)
C19	4816.6(6)	1856(2)	3995.2(8)	29.0(3)
C20	5539.5(6)	2830.9(18)	4095.2(7)	23.4(2)
C21	6197.2(6)	1774.4(17)	4502.8(7)	22.5(2)

Table 17: Anisotropic Displacement Parameters (×10^4) for **4ae**. The anisotropic displacement factor exponent takes the form: $-2\pi^2[h^2a^{*2} \times U_{11}+ ... +2hka^* \times b^* \times U_{12}]$

Atom	U_{11}	U_{22}	U_{33}	U_{23}	U_{13}	U_{12}
O1	33.3(4)	33.8(5)	32.3(4)	4.0(3)	-11.9(3)	0.1(4)
O2	30.6(4)	20.1(4)	48.9(5)	-3.3(3)	-7.4(4)	-1.2(3)
C1	23.2(5)	18.2(5)	30.2(5)	-0.1(4)	-7.2(4)	-3.5(4)
C2	18.3(5)	27.4(6)	39.1(6)	1.1(5)	-3.6(4)	-1.6(4)
C3	19.0(5)	29.2(6)	33.8(6)	-1.2(4)	3.0(4)	-0.2(4)
C4	20.5(5)	21.4(5)	34.7(6)	-1.2(4)	-1.7(4)	2.0(4)
C5	18.5(5)	18.6(5)	23.1(5)	1.0(4)	-1.4(4)	-0.4(4)
C6	18.9(4)	19.1(5)	20.9(5)	1.8(4)	-1.9(4)	-0.1(4)
C7	22.9(5)	19.3(5)	22.9(5)	0.8(4)	-3.1(4)	0.3(4)
C8	22.9(5)	27.6(6)	24.3(5)	0.5(4)	2.3(4)	-3.0(4)
C9	27.3(5)	26.7(6)	25.3(5)	5.0(4)	0.9(4)	-3.3(4)
C10	31.7(6)	28.8(6)	29.8(6)	-6.0(5)	-1.4(4)	1.8(5)
C11	21.8(5)	20.9(5)	24.0(5)	-1.6(4)	0.1(4)	-1.7(4)
C12	22.0(5)	22.4(5)	18.3(4)	-2.8(4)	-1.2(4)	-0.8(4)
C13	23.0(5)	22.9(5)	22.4(5)	-1.7(4)	-0.3(4)	-3.6(4)
C14	29.4(5)	21.9(5)	23.4(5)	0.2(4)	0.0(4)	-0.1(4)
C15	24.6(5)	29.4(6)	17.7(4)	-2.8(4)	-0.3(4)	2.1(4)
C16	30.0(6)	35.0(7)	24.9(5)	-0.7(4)	-1.0(4)	6.7(5)
C17	24.7(5)	50.1(8)	28.8(6)	-1.4(5)	-3.0(4)	8.5(5)
C18	22.1(5)	52.3(8)	28.7(6)	-3.1(5)	-1.7(4)	-3.7(5)
C19	23.9(5)	37.7(7)	24.6(5)	-1.8(4)	-1.2(4)	-5.5(5)
C20	21.9(5)	29.9(6)	17.8(4)	-3.6(4)	-0.9(4)	-2.2(4)
C21	24.1(5)	22.4(5)	20.4(5)	-1.8(4)	-0.4(4)	-3.1(4)

Table 18: Bond Lengths in Å for **4ae**.

Atom	Atom	Length/Å
O1	C1	1.2155(15)
O2	C11	1.2153(15)
C1	C2	1.5119(16)
C1	C7	1.5231(14)
C2	C3	1.5319(16)
C3	C4	1.5480(16)

Atom	Atom	Length/Å
C3	C8	1.5525(15)
C4	C5	1.5589(14)
C5	C6	1.5531(14)
C5	C8	1.5701(14)
C5	C11	1.5239(14)
C6	C7	1.5571(14)
C6	C9	1.5318(14)
C7	C10	1.5275(15)
C11	C12	1.5051(14)
C12	C13	1.4194(16)
C12	C21	1.3790(15)
C13	C14	1.3687(16)
C14	C15	1.4191(16)
C15	C16	1.4212(16)
C15	C20	1.4183(17)
C16	C17	1.3699(19)
C17	C18	1.406(2)
C18	C19	1.3705(18)
C19	C20	1.4216(15)
C20	C21	1.4162(15)

Table 19: Bond Angles in ° for **4ae**.

Atom	Atom	Atom	Angle/°
O1	C1	C2	121.58(10)
O1	C1	C7	121.41(10)
C2	C1	C7	117.00(9)
C1	C2	C3	114.17(9)
C2	C3	C4	112.84(10)
C2	C3	C8	115.94(9)
C4	C3	C8	88.20(8)
C3	C4	C5	89.13(8)
C4	C5	C8	87.20(8)
C6	C5	C4	115.09(8)
C6	C5	C8	114.64(8)
C11	C5	C4	113.80(9)

Atom	Atom	Atom	Angle/°
C11	C5	C6	108.06(8)
C11	C5	C8	117.13(9)
C5	C6	C7	114.46(8)
C9	C6	C5	110.23(9)
C9	C6	C7	112.11(8)
C1	C7	C6	110.91(8)
C1	C7	C10	111.74(9)
C10	C7	C6	111.56(9)
C3	C8	C5	88.57(8)
O2	C11	C5	120.51(10)
O2	C11	C12	119.75(10)
C12	C11	C5	119.66(9)
C13	C12	C11	123.11(9)
C21	C12	C11	117.51(10)
C21	C12	C13	119.37(10)
C14	C13	C12	120.34(10)
C13	C14	C15	121.15(10)
C14	C15	C16	122.35(11)
C20	C15	C14	118.82(10)
C20	C15	C16	118.82(10)
C17	C16	C15	120.42(12)
C16	C17	C18	120.86(11)
C19	C18	C17	120.10(11)
C18	C19	C20	120.58(12)
C15	C20	C19	119.18(10)
C21	C20	C15	118.91(10)
C21	C20	C19	121.90(11)
C12	C21	C20	121.33(10)

Table 20: Torsion Angles in ° for **4ae**.

Atom	Atom	Atom	Atom	Angle/°
O1	C1	C2	C3	124.70(12)
O1	C1	C7	C6	-99.59(12)
O1	C1	C7	C10	25.57(14)
O2	C11	C12	C13	-158.41(11)

Atom	Atom	Atom	Atom	Angle/°
O2	C11	C12	C21	22.18(15)
C1	C2	C3	C4	-35.59(13)
C1	C2	C3	C8	64.02(13)
C2	C1	C7	C6	80.46(11)
C2	C1	C7	C10	-154.38(10)
C2	C3	C4	C5	97.67(10)
C2	C3	C8	C5	-94.93(10)
C3	C4	C5	C6	-96.36(10)
C3	C4	C5	C8	19.61(8)
C3	C4	C5	C11	138.13(9)
C4	C3	C8	C5	19.69(8)
C4	C5	C6	C7	66.56(11)
C4	C5	C6	C9	-60.91(11)
C4	C5	C8	C3	-19.56(8)
C4	C5	C11	O2	13.48(14)
C4	C5	C11	C12	-169.76(9)
C5	C6	C7	C1	-54.25(11)
C5	C6	C7	C10	-179.52(9)
C5	C11	C12	C13	24.80(14)
C5	C11	C12	C21	-154.60(10)
C6	C5	C8	C3	96.84(9)
C6	C5	C11	O2	-115.68(11)
C6	C5	C11	C12	61.09(11)
C7	C1	C2	C3	-55.35(13)
C8	C3	C4	C5	-19.83(8)
C8	C5	C6	C7	-32.38(12)
C8	C5	C6	C9	-159.85(9)
C8	C5	C11	O2	113.03(12)
C8	C5	C11	C12	-70.20(12)
C9	C6	C7	C1	72.25(11)
C9	C6	C7	C10	-53.02(12)
C11	C5	C6	C7	-165.01(8)
C11	C5	C6	C9	67.51(10)
C11	C5	C8	C3	-134.96(9)
C11	C12	C13	C14	179.82(9)

Atom	Atom	Atom	Atom	Angle/°
C11	C12	C21	C20	177.59(9)
C12	C13	C14	C15	2.63(16)
C13	C12	C21	C20	-1.84(15)
C13	C14	C15	C16	177.19(10)
C13	C14	C15	C20	-1.85(16)
C14	C15	C16	C17	-177.19(11)
C14	C15	C20	C19	178.42(10)
C14	C15	C20	C21	-0.73(15)
C15	C16	C17	C18	-1.24(18)
C15	C20	C21	C12	2.57(15)
C16	C15	C20	C19	-0.65(15)
C16	C15	C20	C21	-179.80(9)
C16	C17	C18	C19	-0.63(19)
C17	C18	C19	C20	1.83(18)
C18	C19	C20	C15	-1.18(16)
C18	C19	C20	C21	177.95(10)
C19	C20	C21	C12	-176.55(10)
C20	C15	C16	C17	1.85(16)
C21	C12	C13	C14	-0.78(15)

Table 21: Hydrogen Fractional Atomic Coordinates (×10^4) and Equivalent Isotropic Displacement Parameters (Å^2×10^3) for **Ste-24-1162**. U_{eq} is defined as 1/3 of the trace of the orthogonalised U_{ij}.

Atom	x	y	z	U_{eq}
H2A	9919(9)	5740(30)	6319(12)	37(4)
H2B	10222(10)	3680(30)	6814(12)	38(4)
H3	9825(9)	2600(20)	5288(11)	28(4)
H4A	8988(9)	780(30)	6645(12)	32(4)
H4B	8915(9)	60(30)	5526(12)	33(4)
H6	7469(8)	4730(20)	6222(10)	21(3)
H7	8528(8)	6660(20)	6472(11)	26(3)
H8A	8680(8)	5500(30)	5022(11)	26(3)
H8B	8706(9)	3500(30)	4393(11)	30(4)
H9A	7376(10)	3070(30)	7743(13)	40(4)
H9B	7316(10)	1320(30)	6958(12)	40(4)
H9C	8075(9)	1640(30)	7635(11)	30(4)

Atom	x	y	z	U_{eq}
H10A	8111(10)	5840(30)	8390(14)	44(4)
H10B	8438(10)	8010(30)	8003(12)	40(4)
H10C	7600(10)	7180(30)	7611(13)	45(5)
H13	7414(9)	5600(30)	4426(11)	30(4)
H14	6365(9)	7300(30)	3708(11)	31(4)
H16	4994(9)	7380(30)	3157(11)	32(4)
H17	3818(11)	5790(30)	3056(13)	46(5)
H18	3684(11)	2250(30)	3583(13)	46(5)
H19	4783(9)	410(30)	4195(11)	33(4)
H21	6157(8)	370(20)	4707(10)	24(3)

7. NMR Spectra of new compounds

Bicyclo[1.1.0]butan-1-yl(3-methoxyphenyl)methanone (1d)

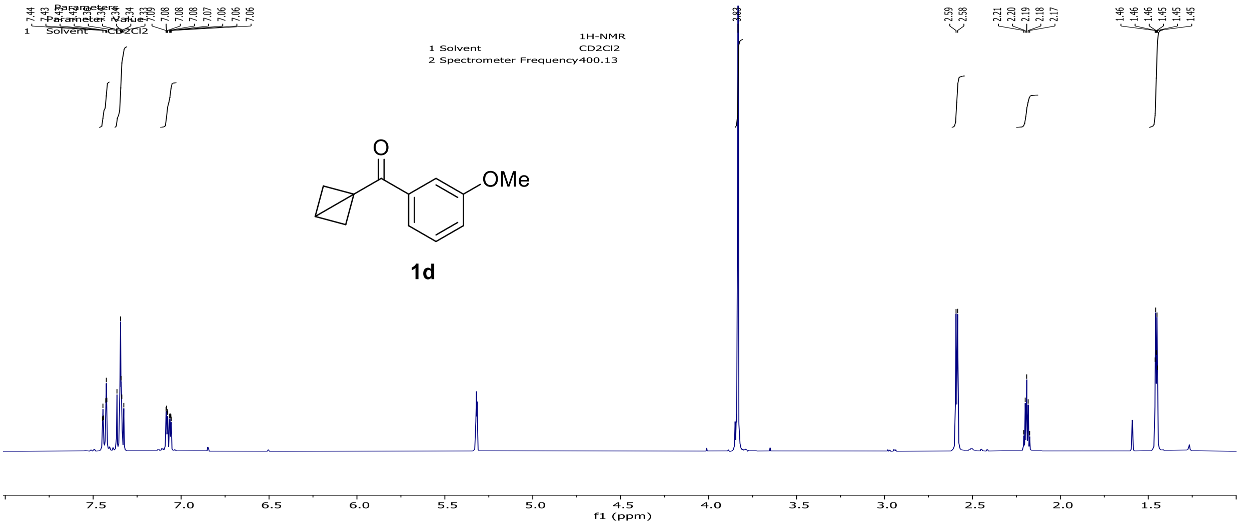

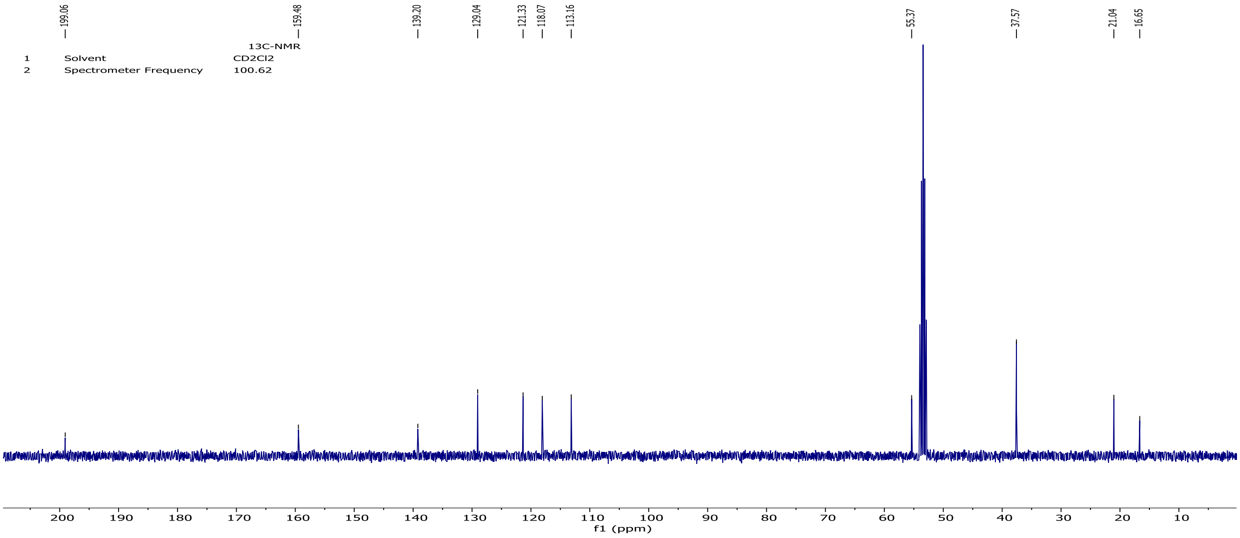

Bicyclo[1.1.0]butan-1-yl(2-methoxyphenyl)methanone (1e)

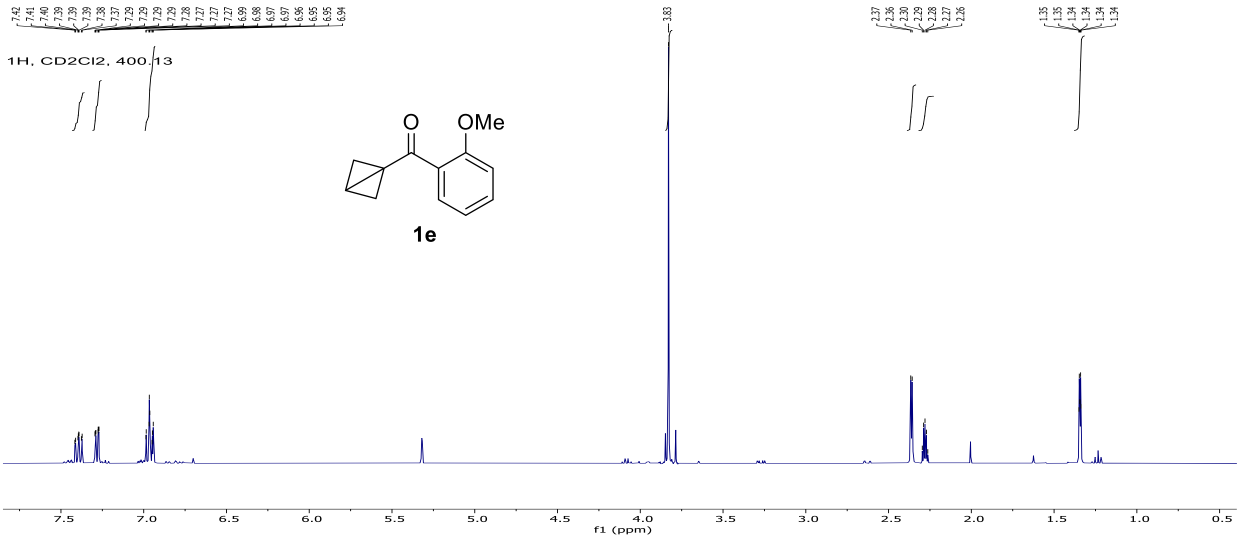

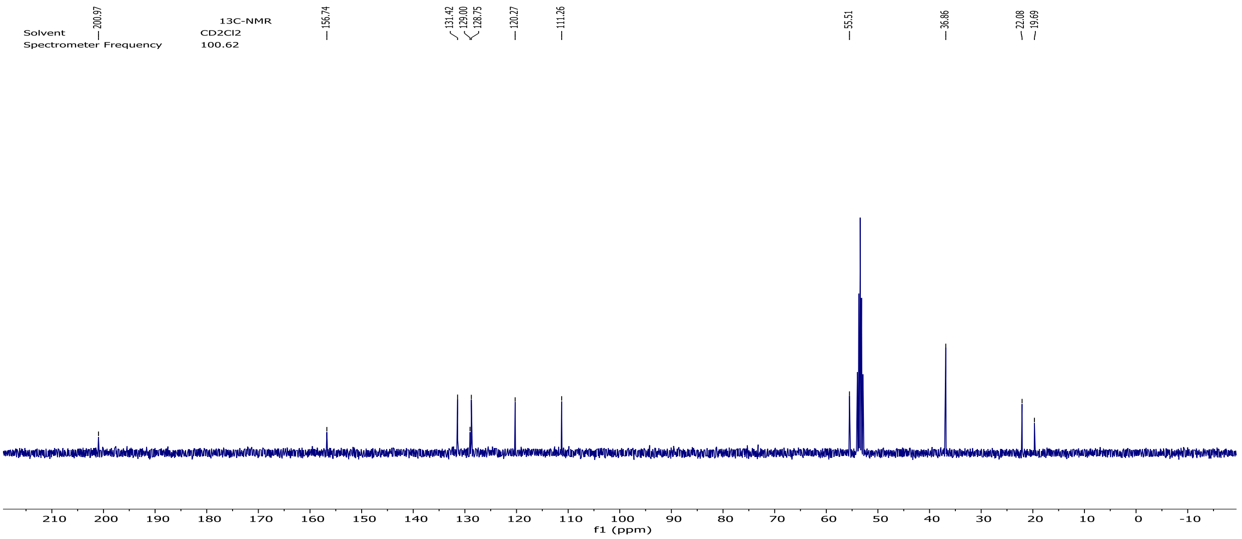

4-(Bicyclo[1.1.0]butane-1-carbonyl)benzonitrile (1h)

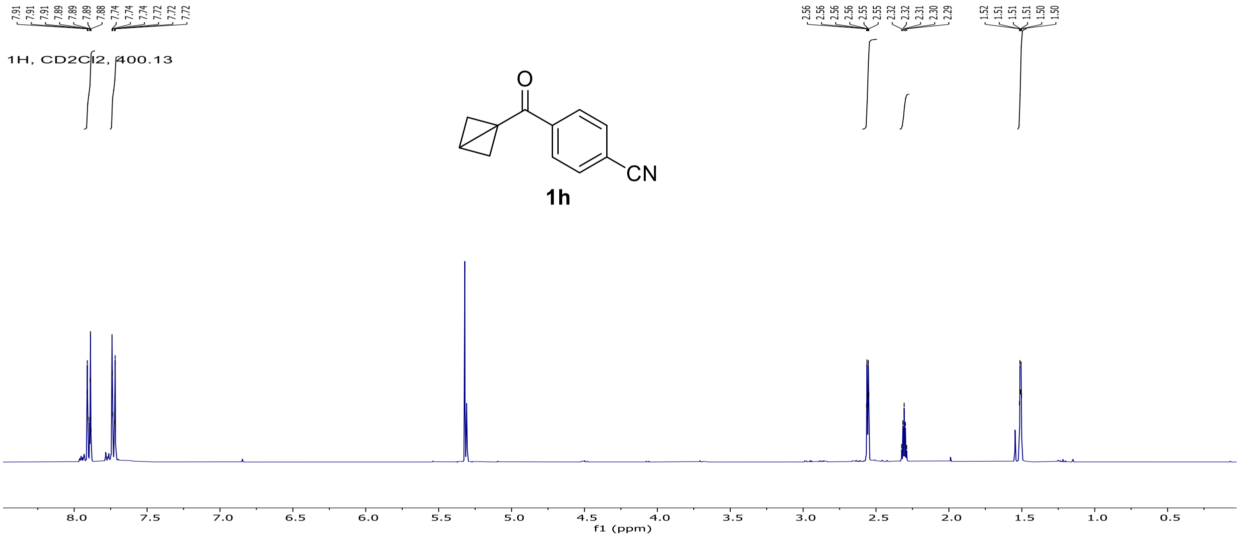

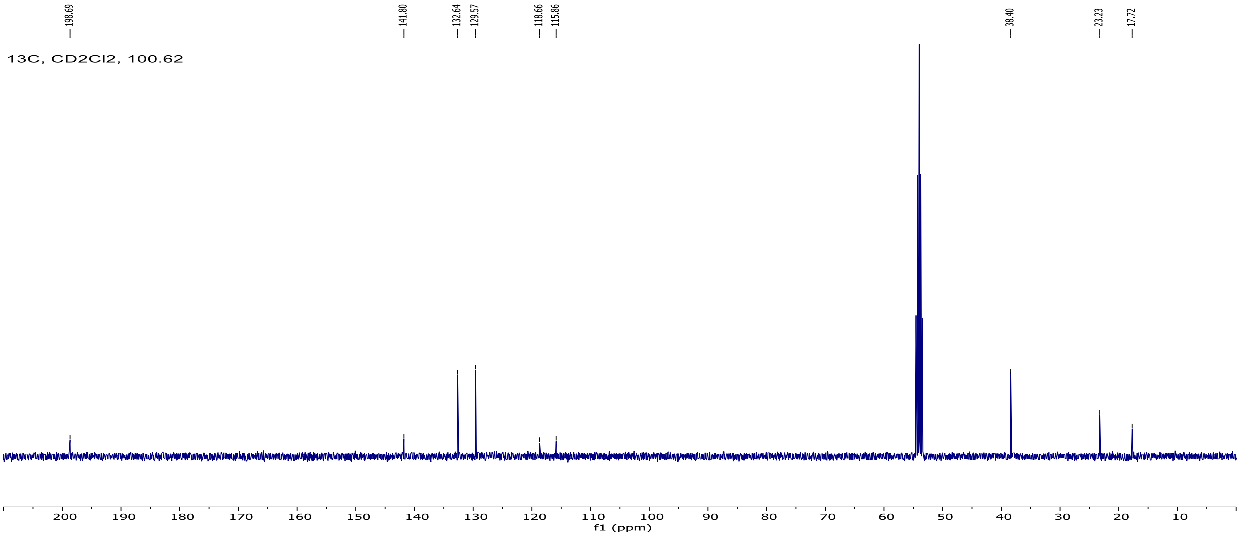

Bicyclo[1.1.0]butan-1-yl(5-bromothiophen-2-yl)methanone (1j)

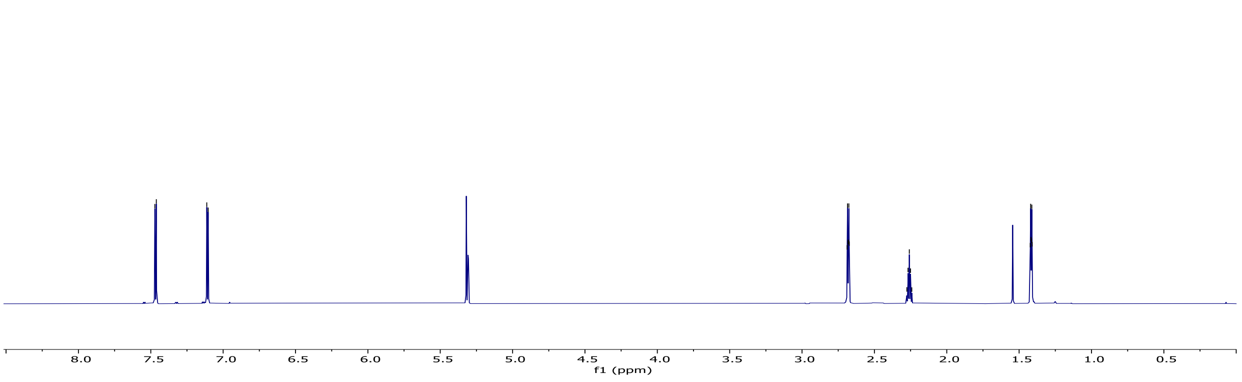

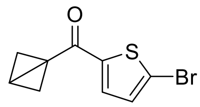

1j

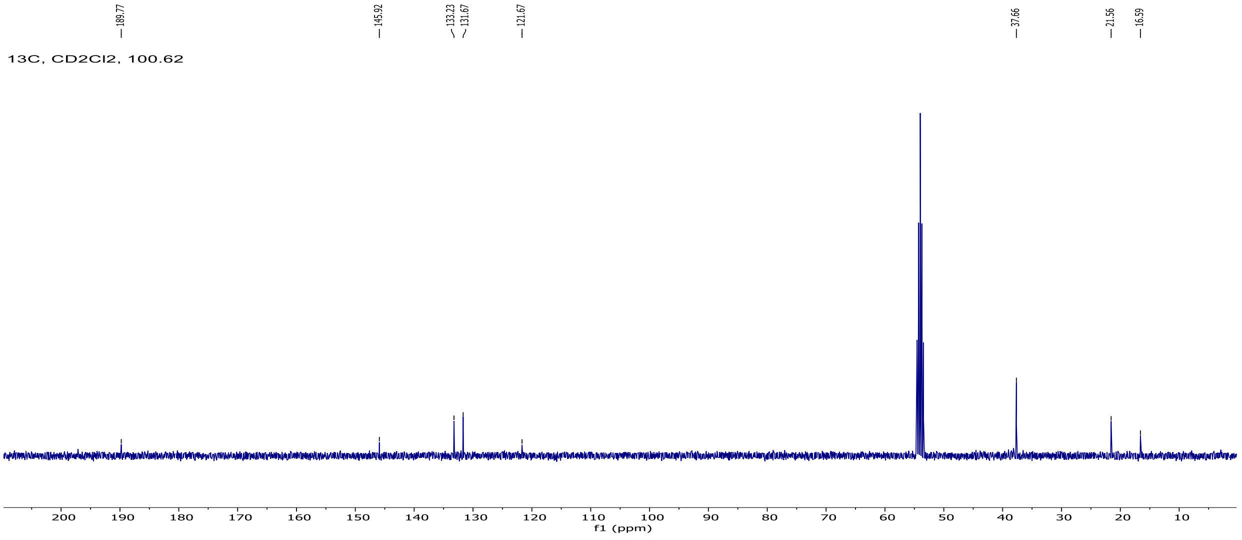

(3-(3,4-Difluorophenyl)bicyclo[1.1.0]butan-1-yl)(naphthalen-2-yl)methanone (1l)

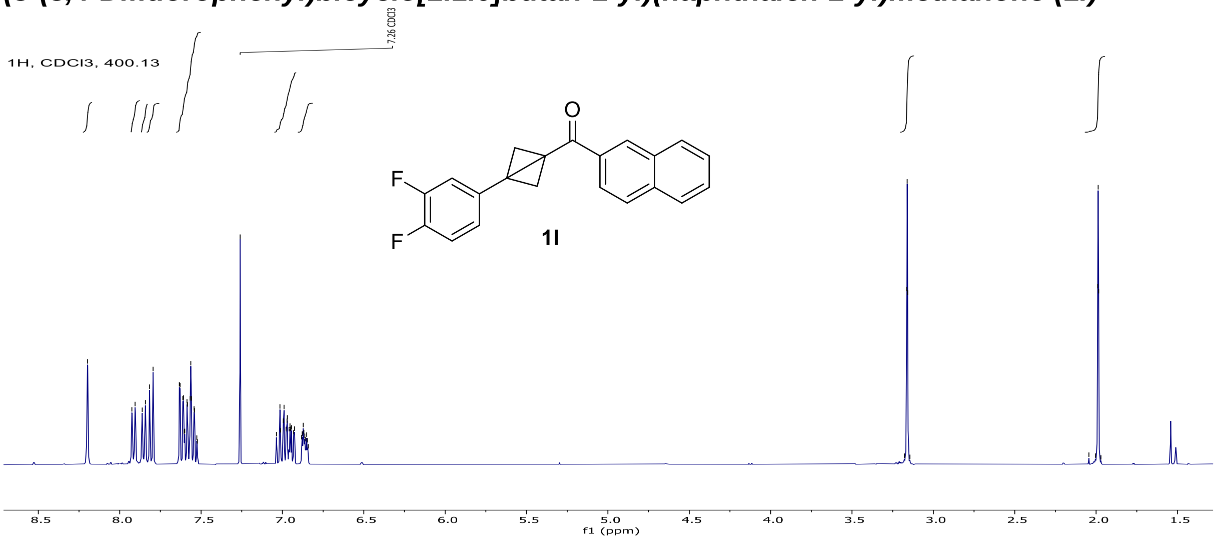

1l

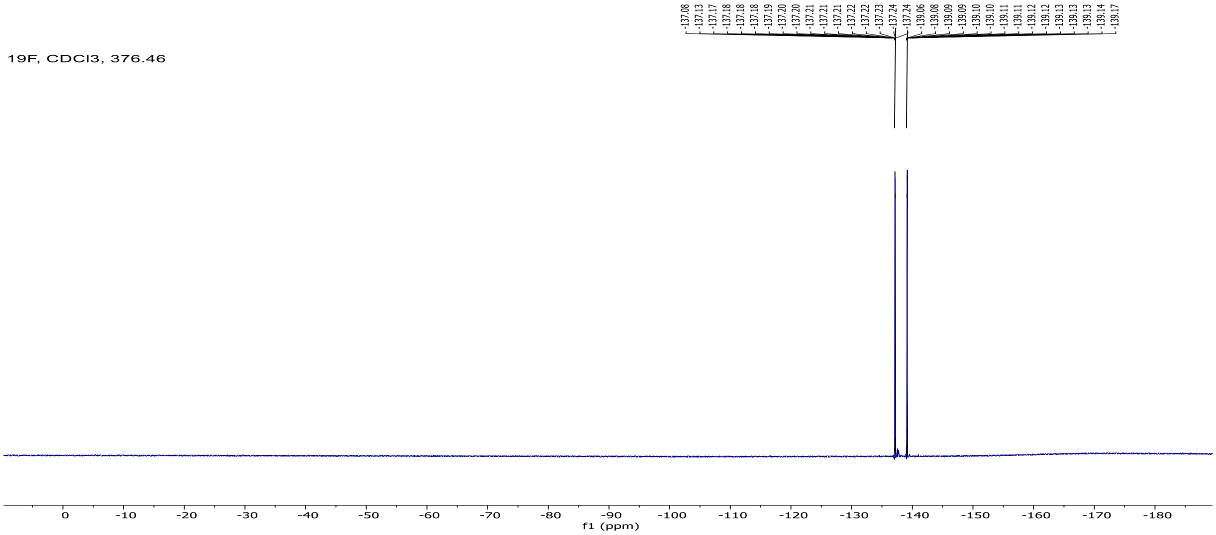

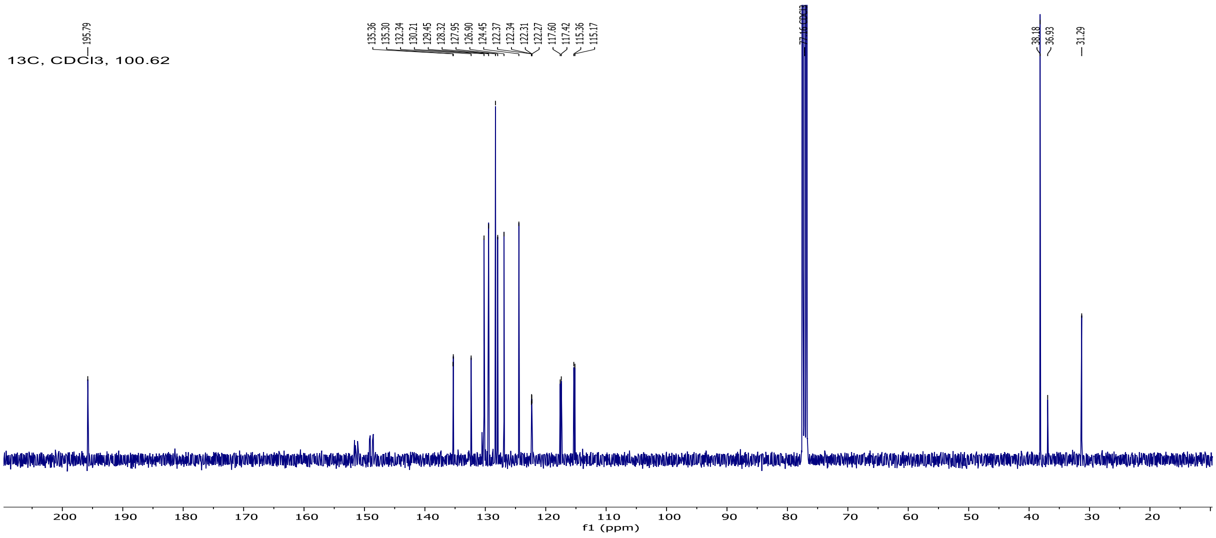

Naphthalen-2-yl(3-(4-(trifluoromethyl)phenyl)bicyclo[1.1.0]butan-1-yl)methanone (1m)

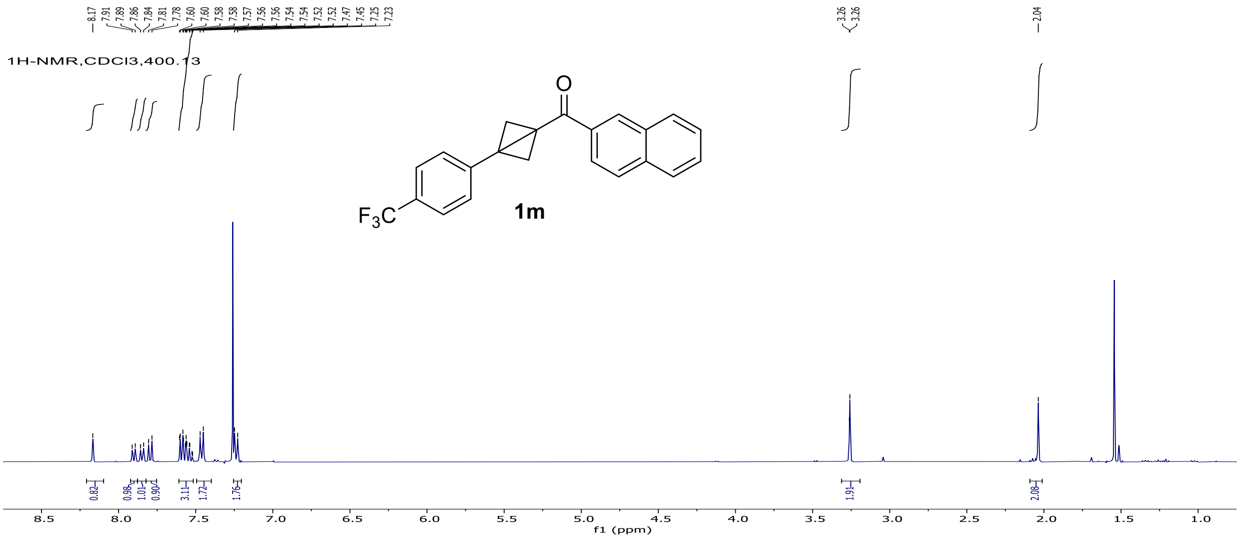

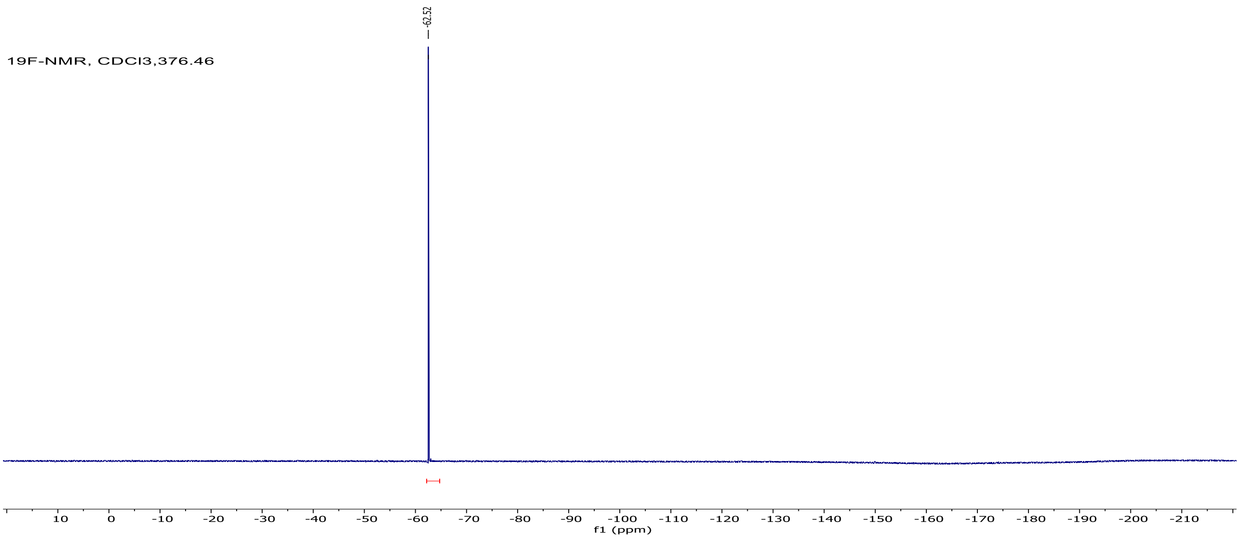

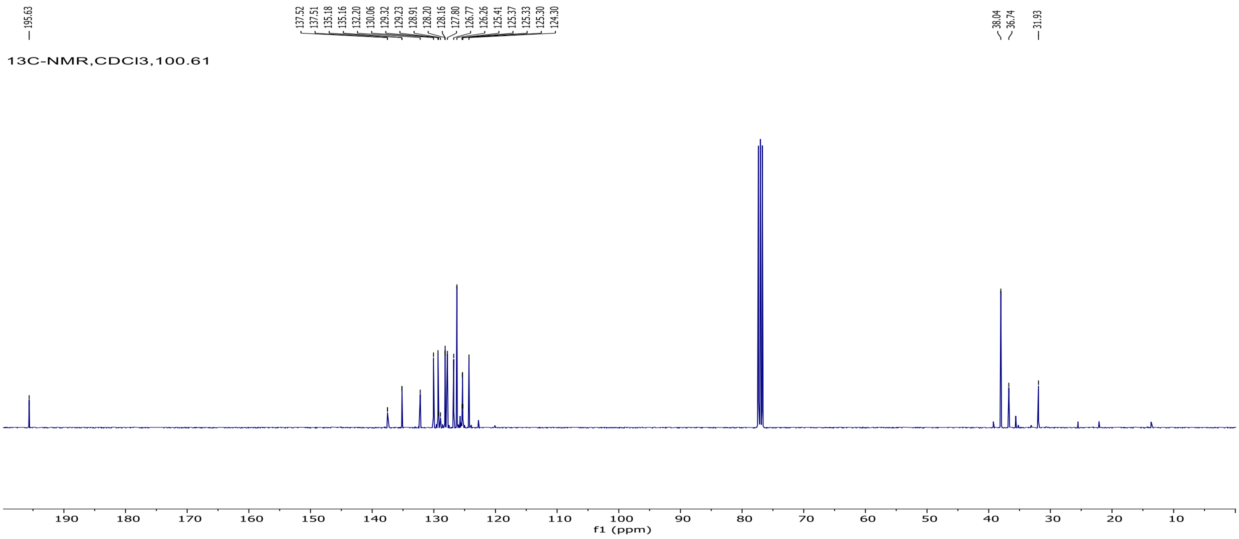

(Z)-tert-Butyl(penta-1,3-dien-3-yloxy)diphenylsilane (2c)

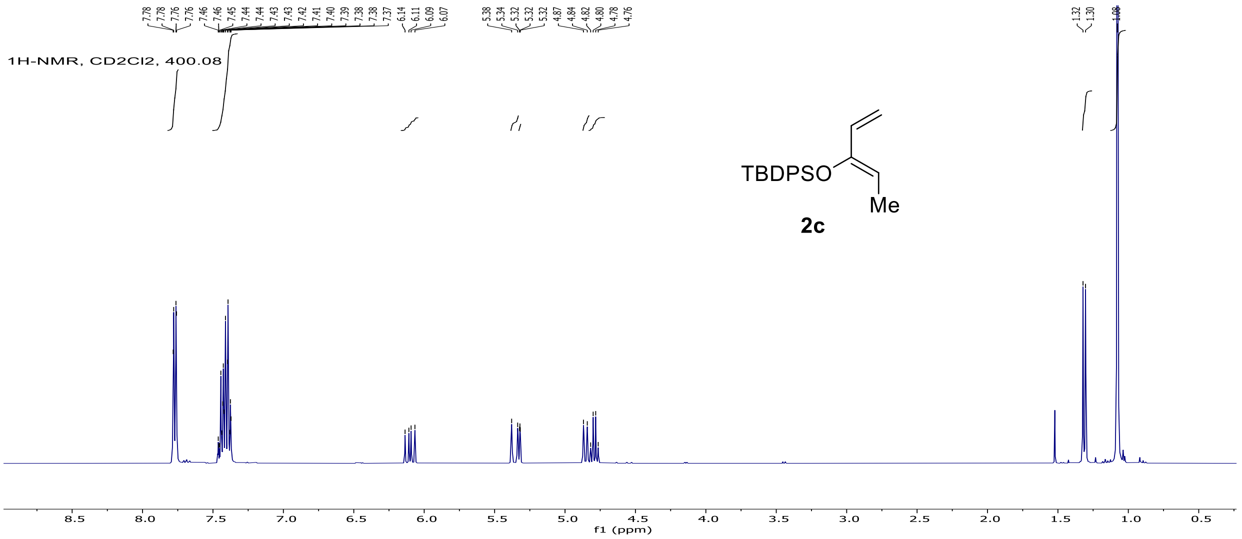

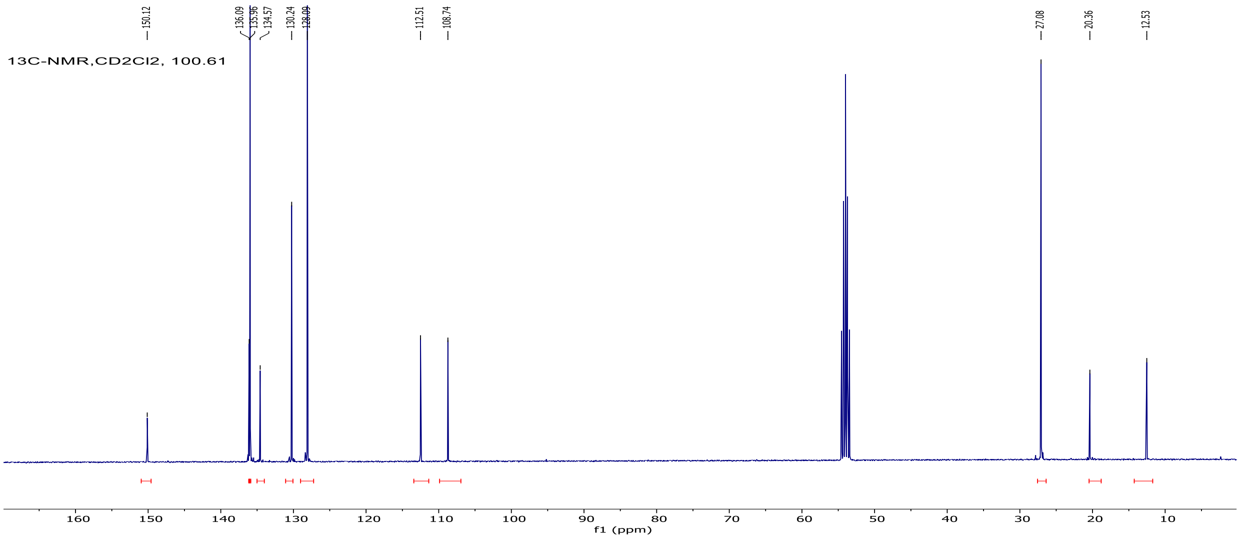

(Z)-tert-Butyl((2-methylpenta-1,3-dien-3-yl)oxy)diphenylsilane (2d)

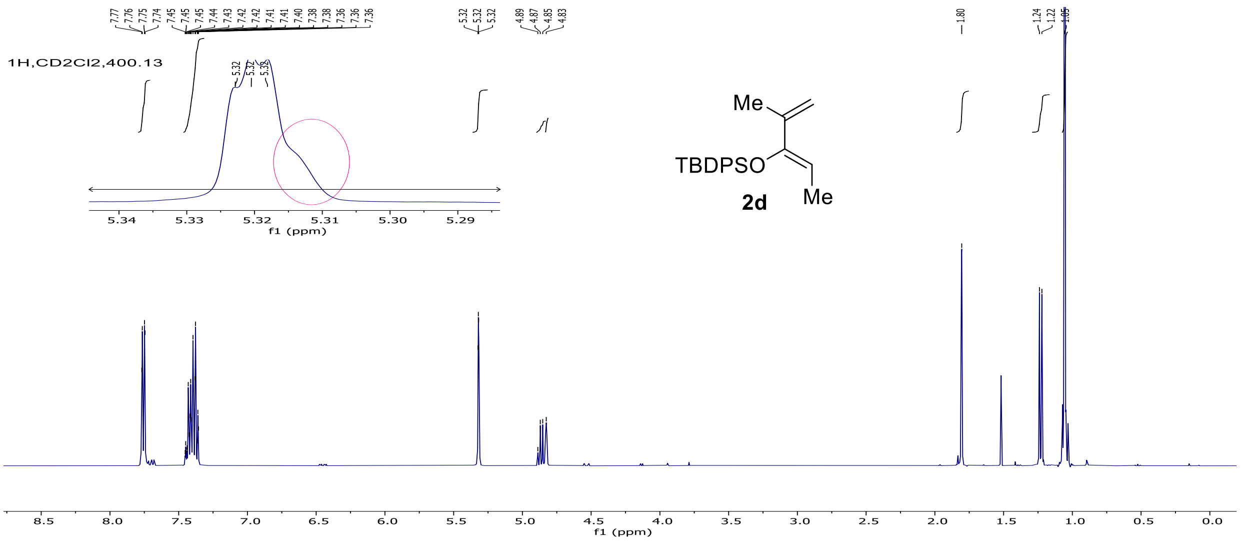

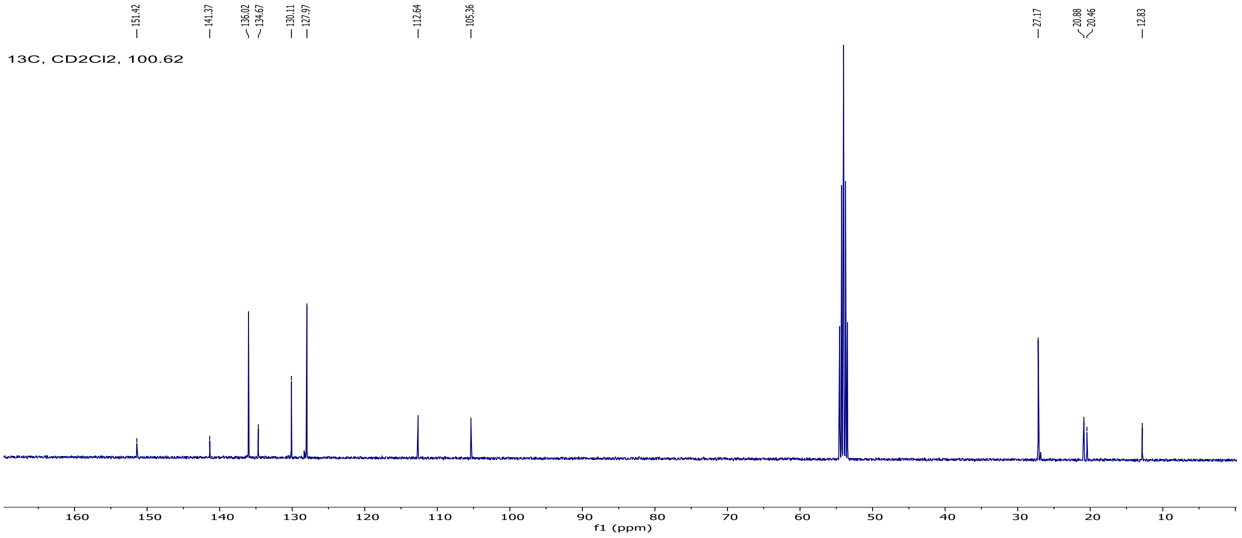

NOESY experiment for the attribution of the C=C geometry:

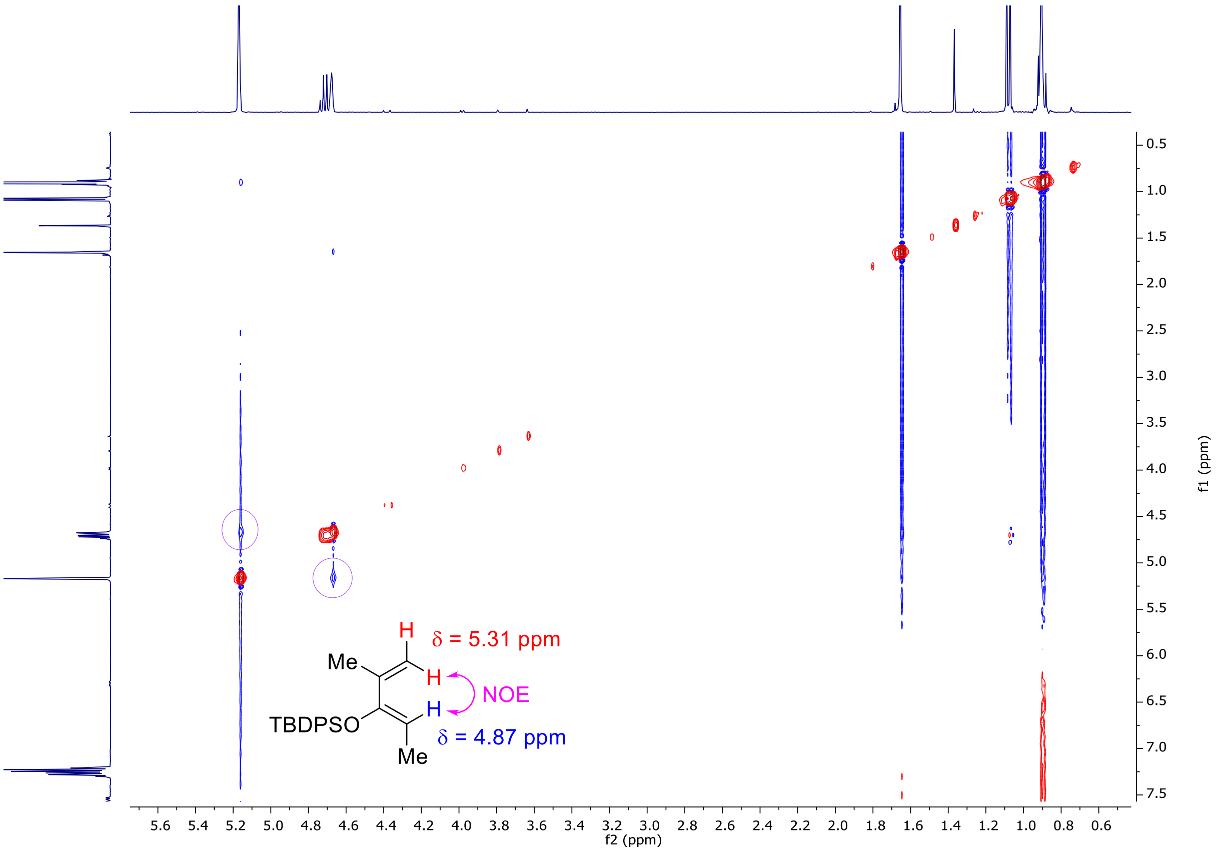

(E)-tert-Butyl((3-methylpenta-1,3-dien-2-yl)oxy)diphenylsilane (2e)

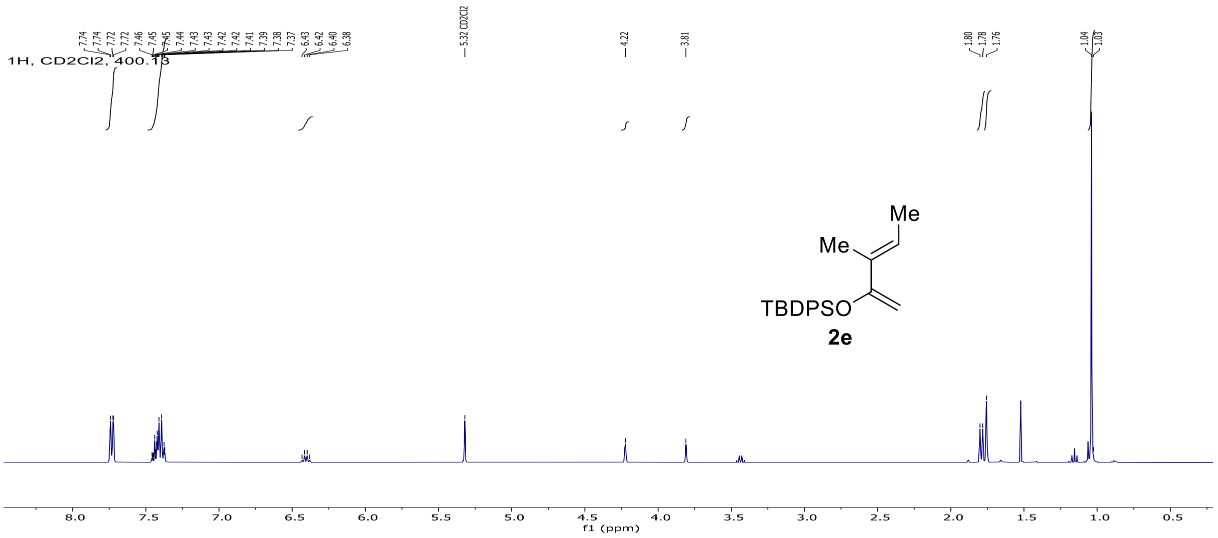

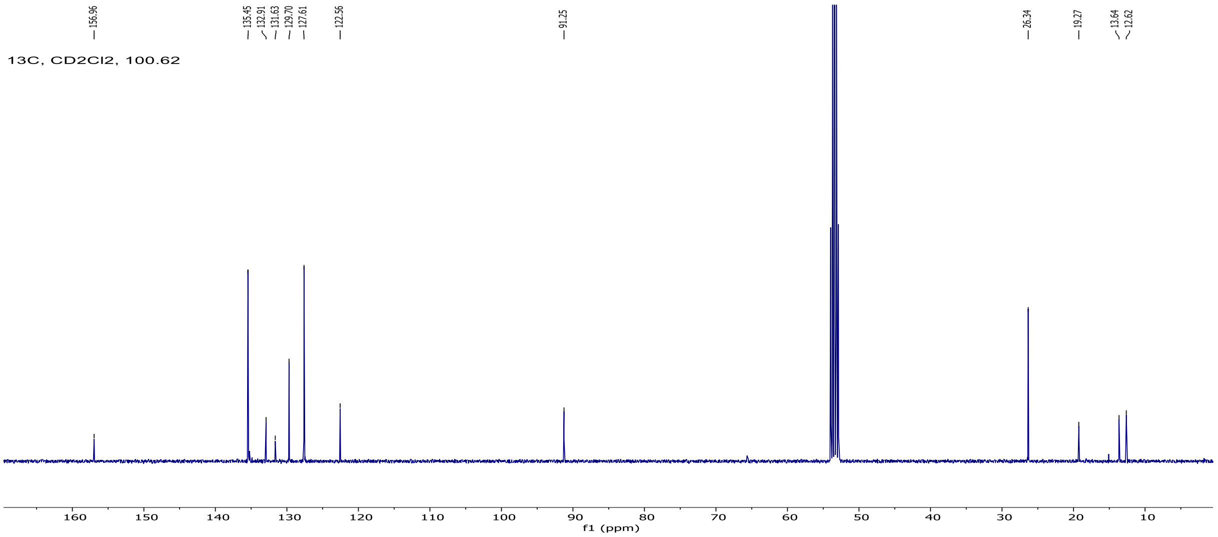

(E)-tert-Butyl(penta-1,3-dien-2-yloxy)diphenylsilane (2e')

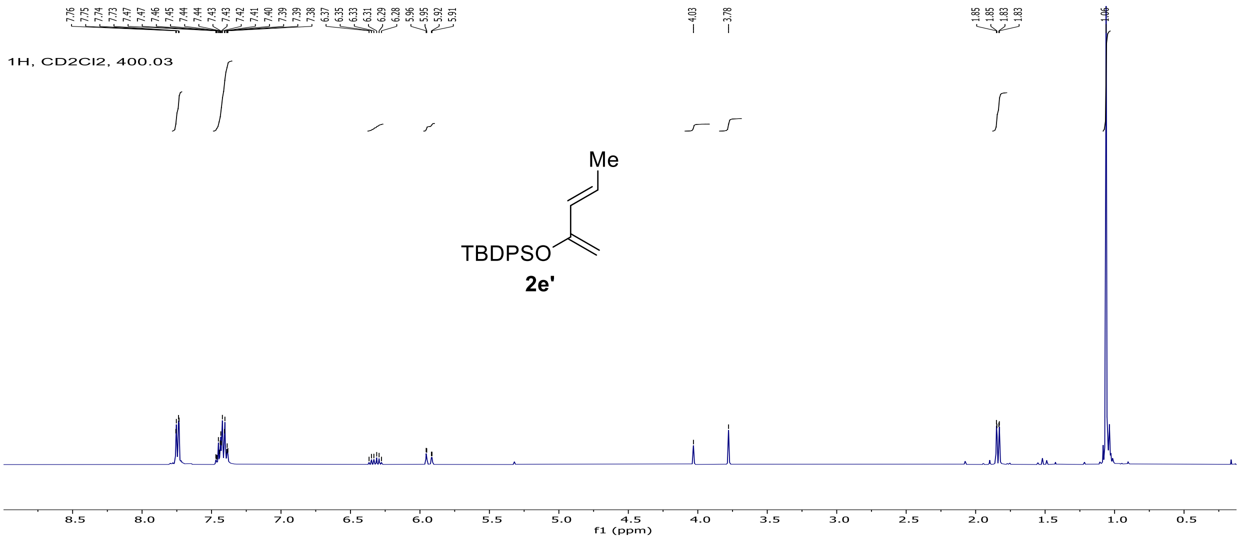

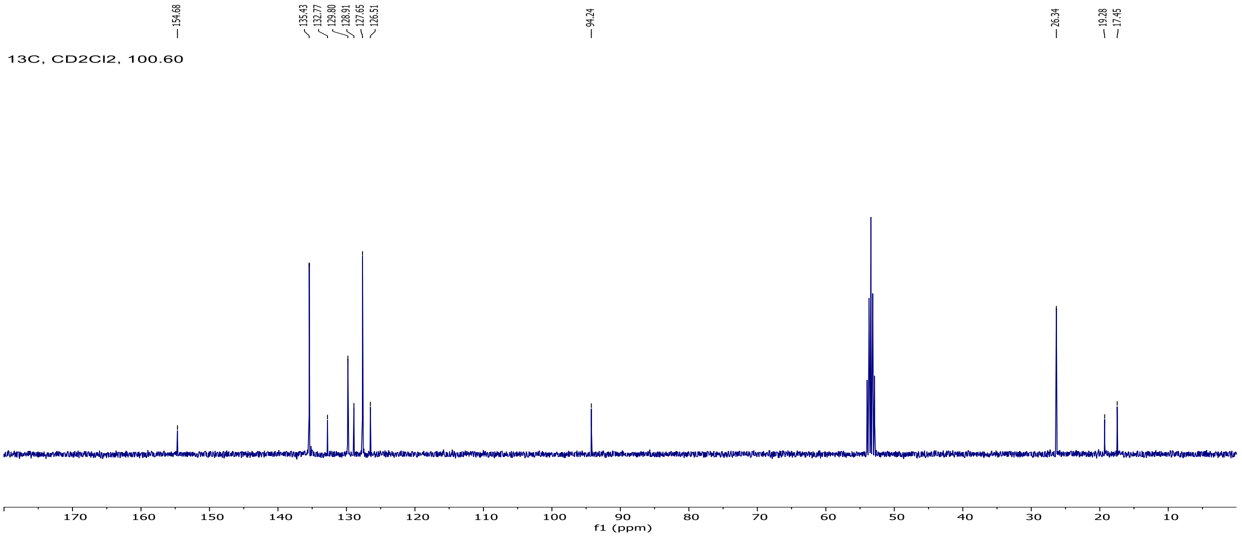

tert-Butyl((3-methylenehept-1-en-2-yl)oxy)diphenylsilane (2g)

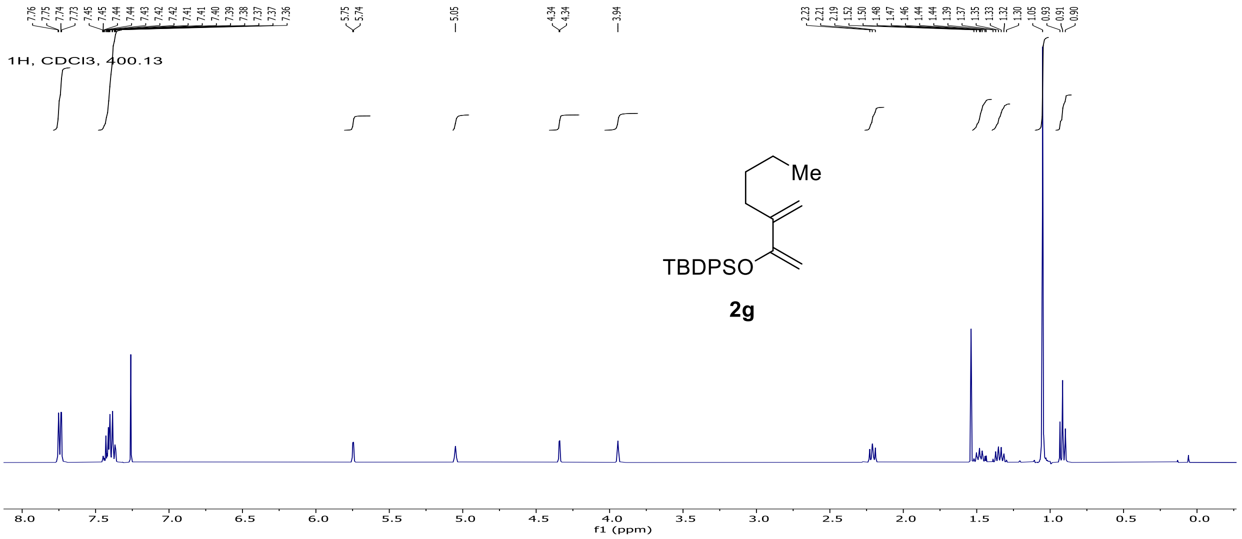

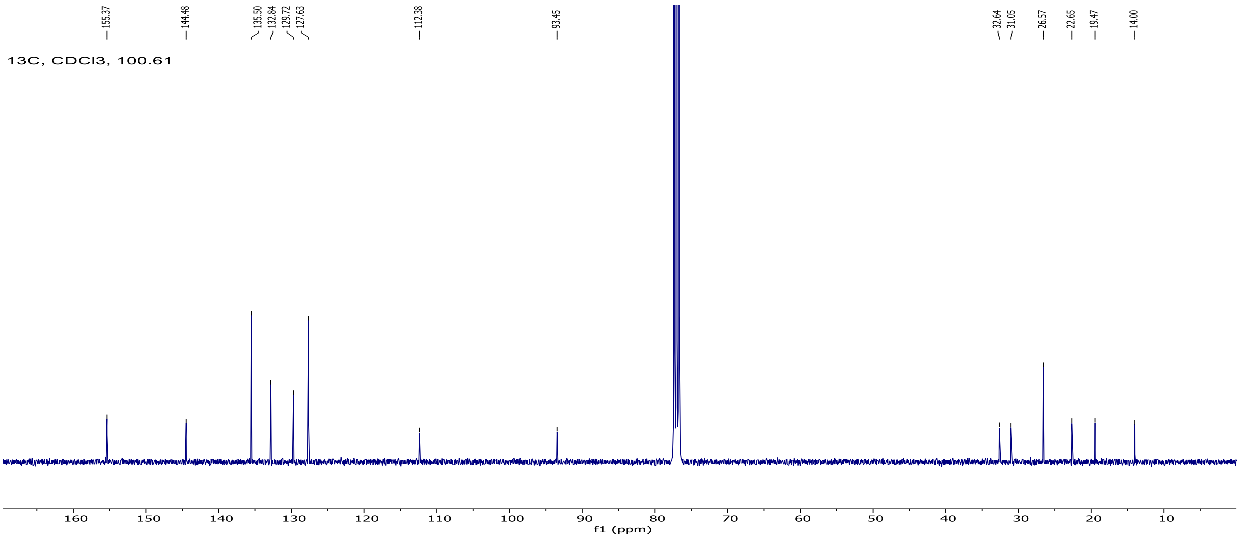

tert-Butyl((3-cyclohexylbuta-1,3-dien-2-yl)oxy)diphenylsilane (2h)

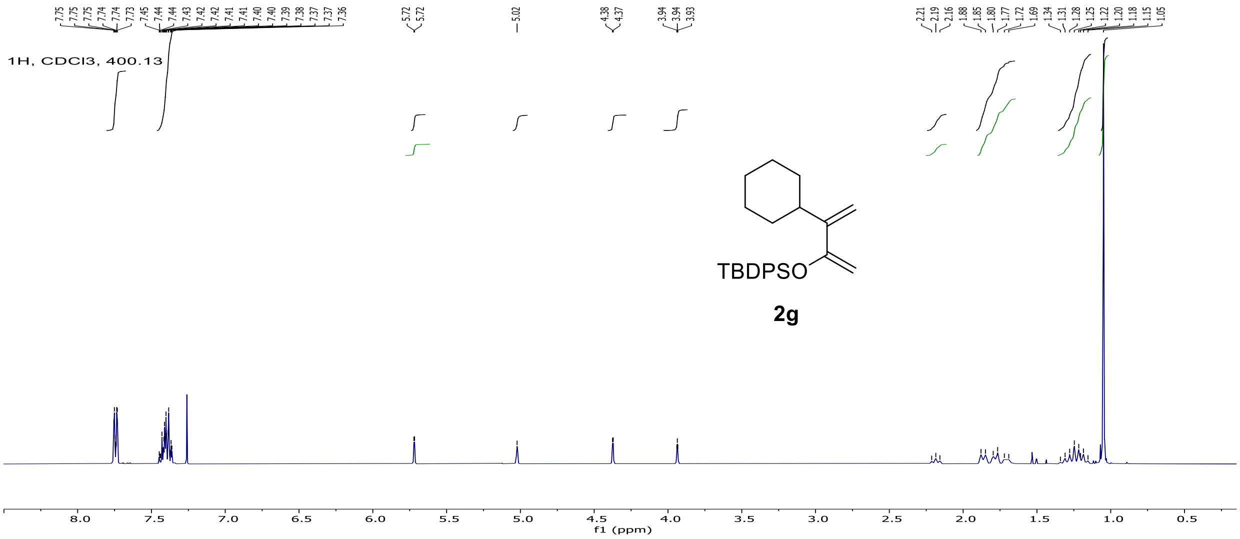

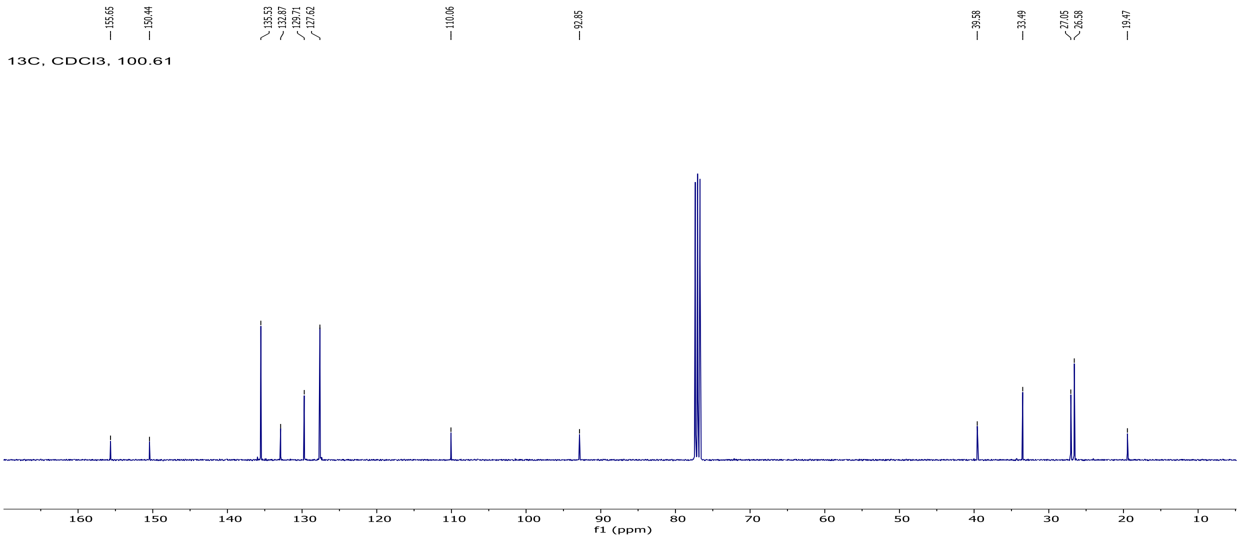

((3-(Benzofuran-5-yl)buta-1,3-dien-2-yl)oxy)(tert-butyl)diphenylsilane (2j)

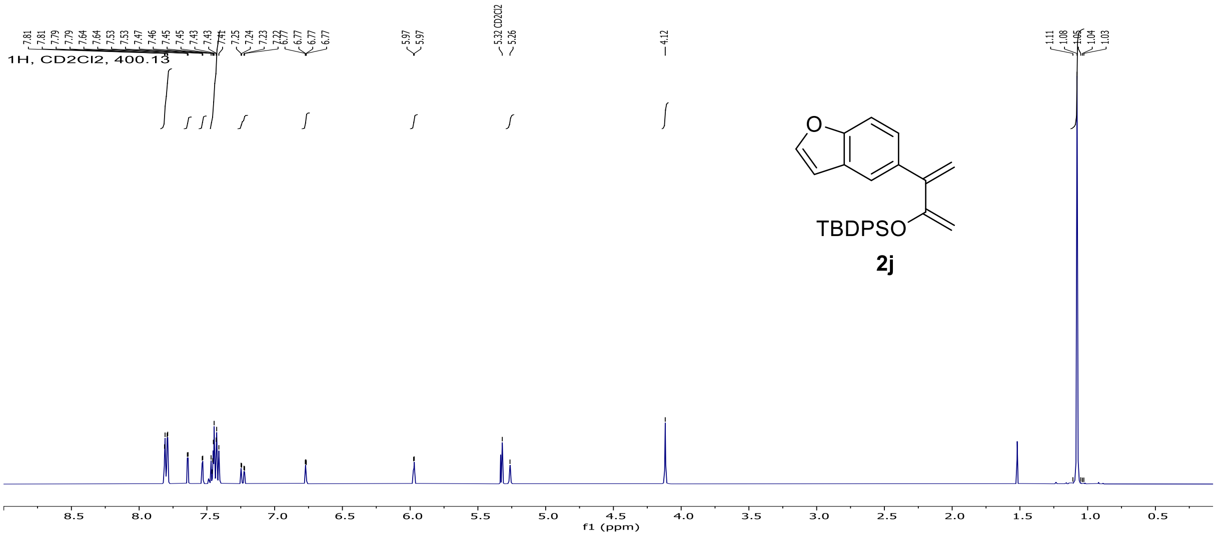

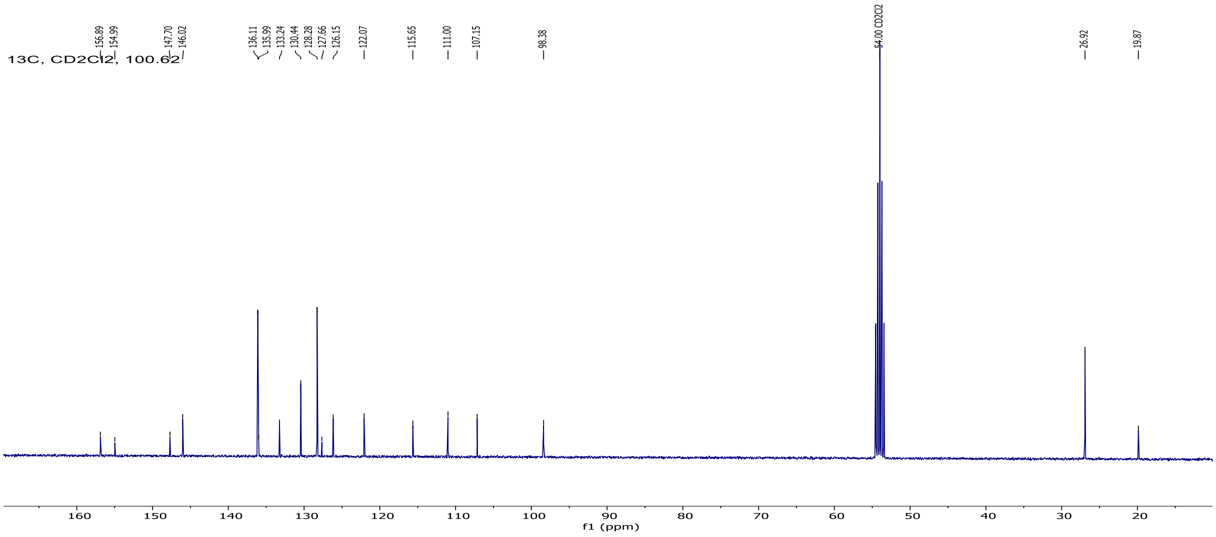

tert-Butyl((3-(4-chlorophenyl)buta-1,3-dien-2-yl)oxy)diphenylsilane (2k)

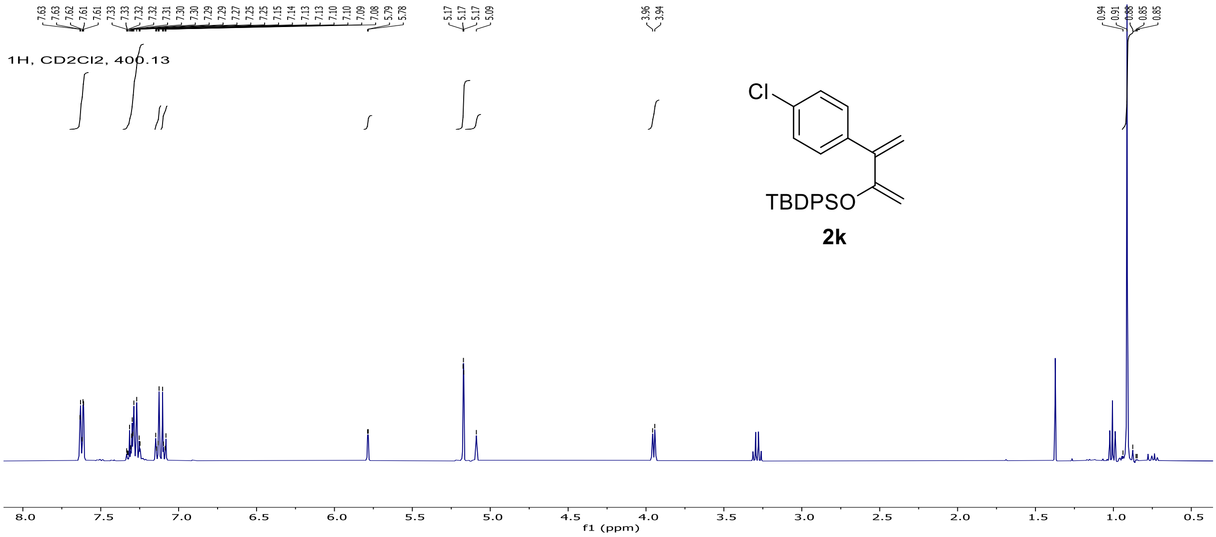

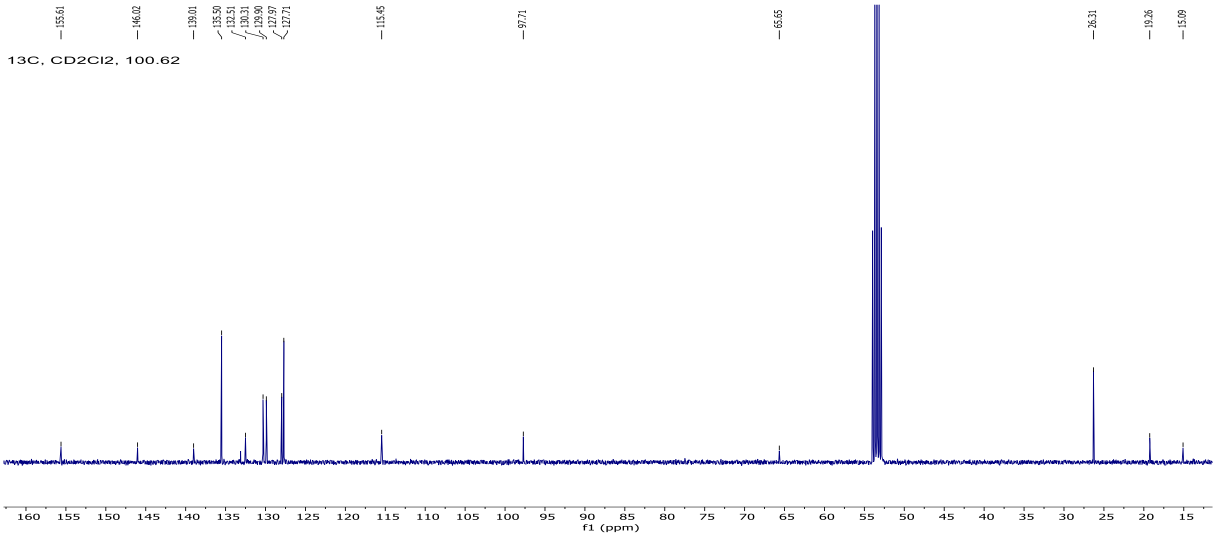

tert-Butyl((3-(naphthalen-1-yl)buta-1,3-dien-2-yl)oxy)diphenylsilane (2l)

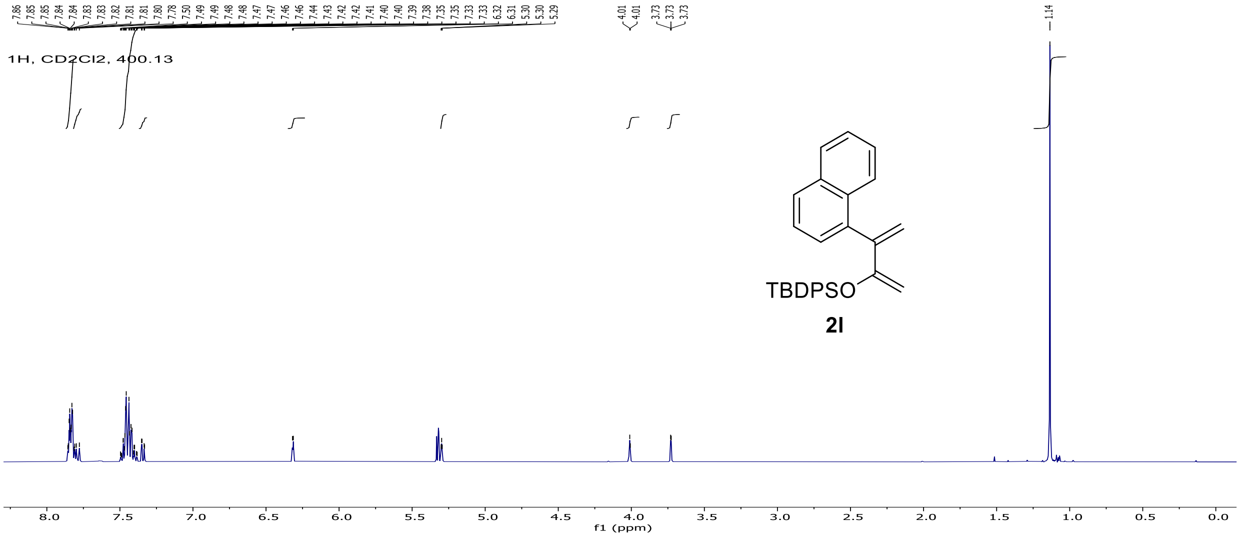

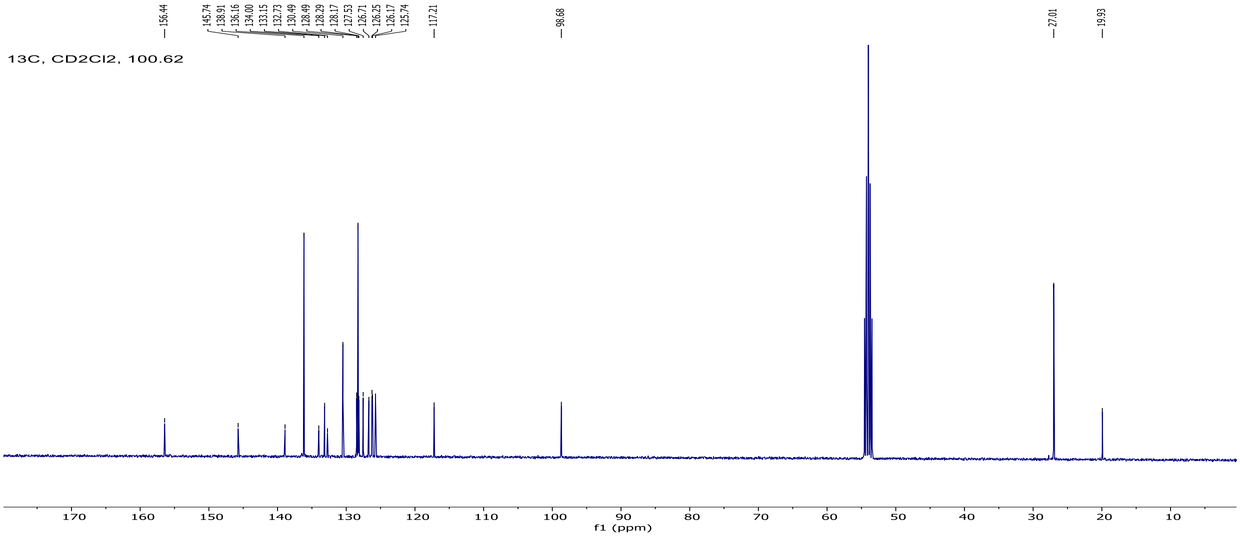

Characterization of 6-(2-naphthoyl)-4-methylbicyclo[4.1.1]octan-3-one (4aa)

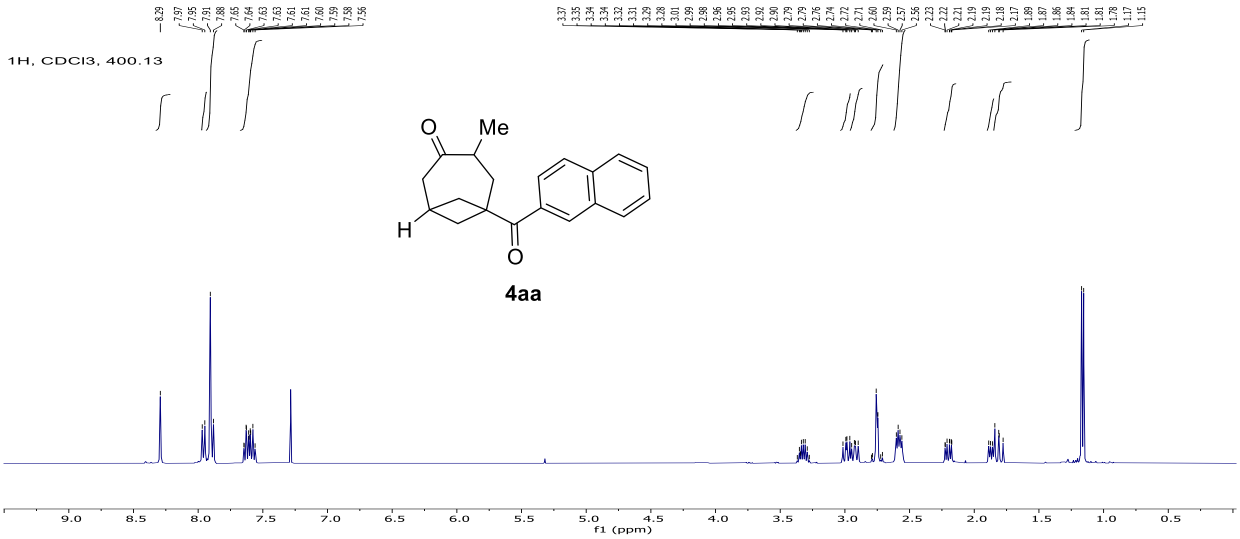

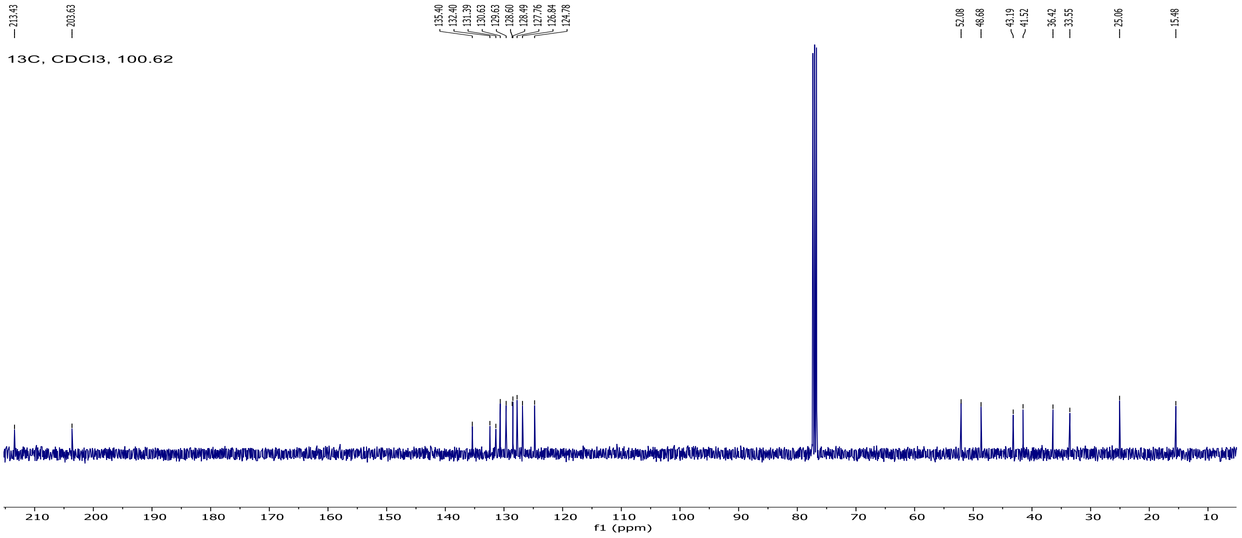
213.43
203.63
135.40
132.40
131.39
130.63
129.63
128.60
128.49
127.76
126.84
124.78
52.08
48.68
43.19
41.52
36.42
33.55
25.06
15.48
13C, CDCl3, 100.62
f1 (ppm)

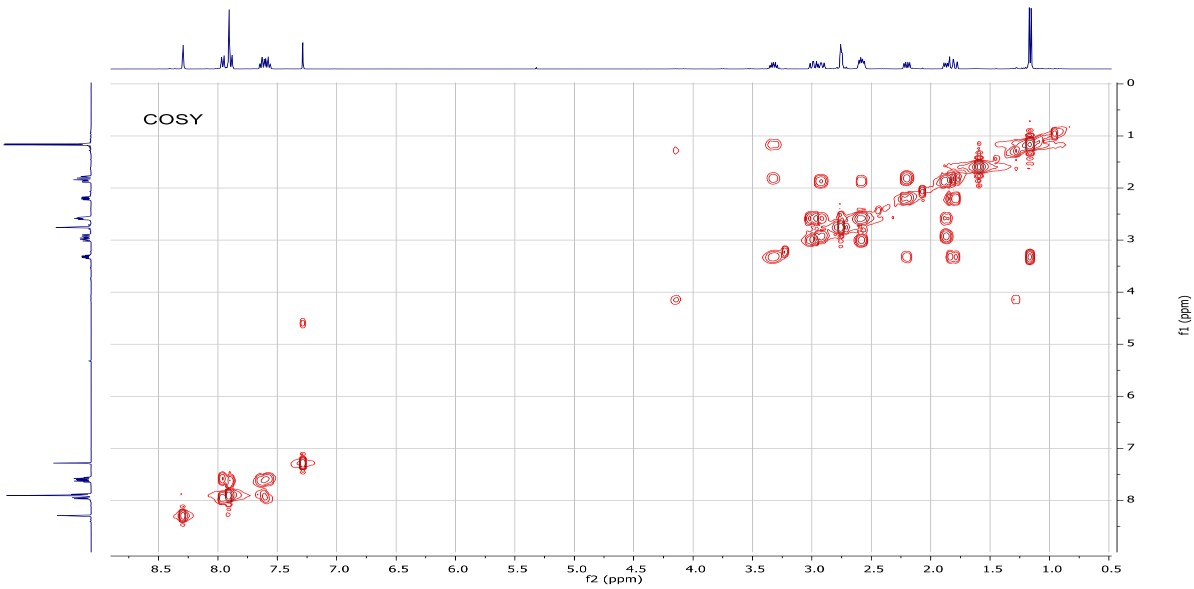
COSY
f2 (ppm)
f1 (ppm)

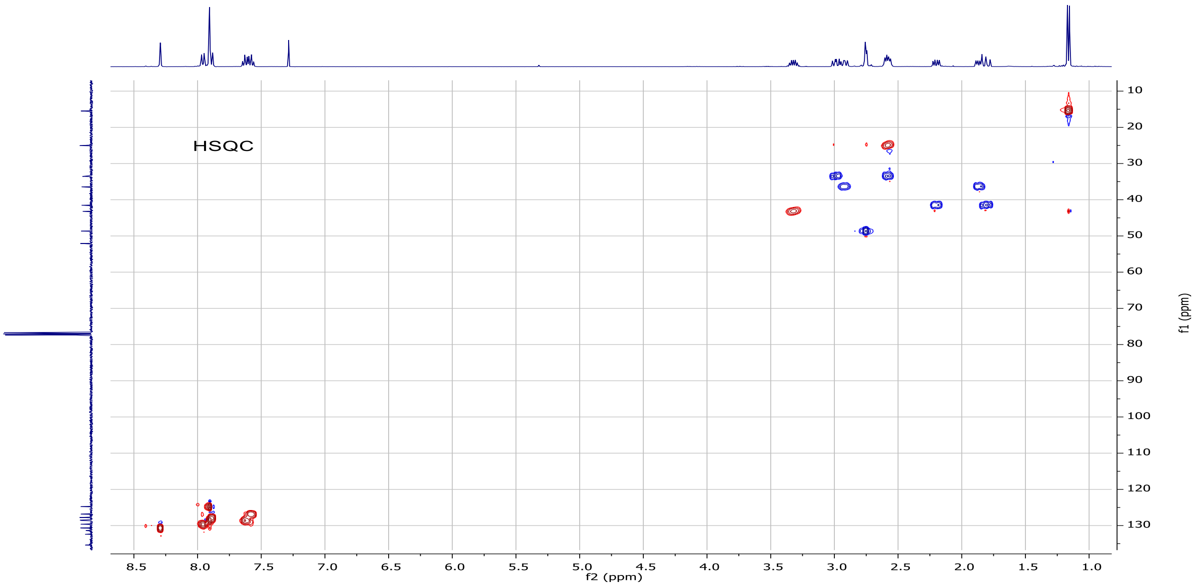
HSQC
f2 (ppm)
f1 (ppm)

(4-((tert-butyldiphenylsilyl)oxy)-3-methylbicyclo[4.1.1]oct-3-en-1-yl)(naphthalen-2-yl)methanoneenol 3 (ca. 90% pure; some impurities are indicated by **)

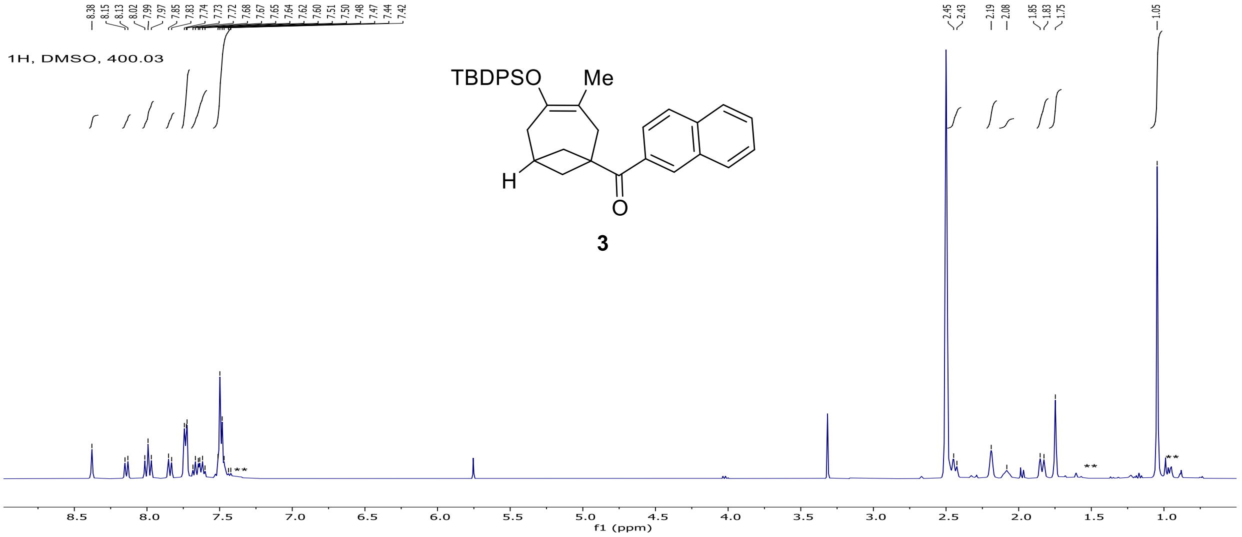

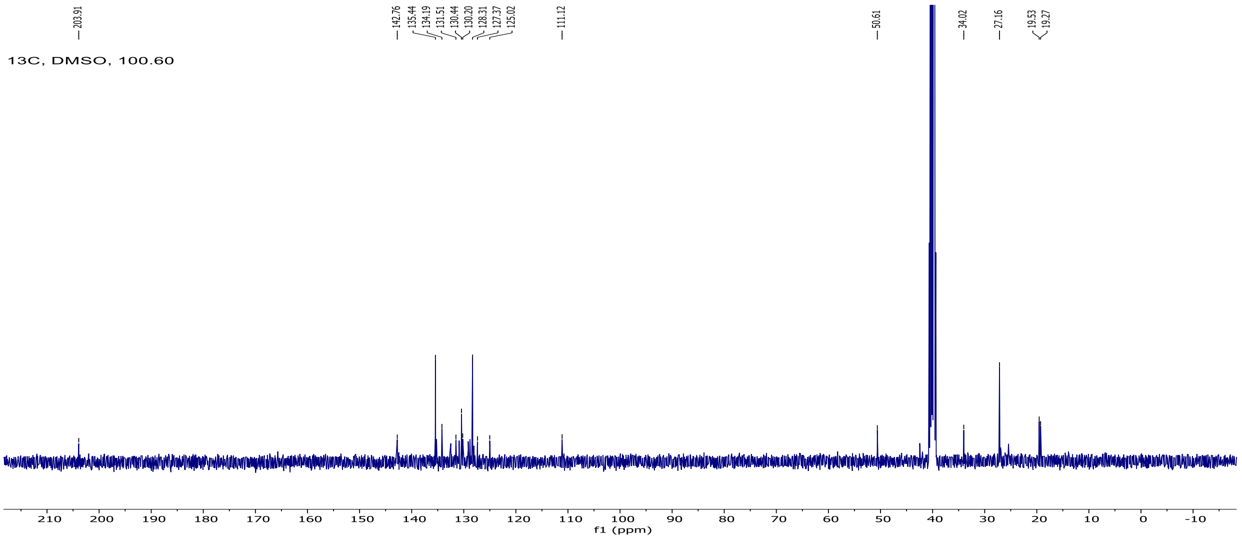

Bicyclo[1.1.0]butan-1-yl(phenyl)methanone (4ba)

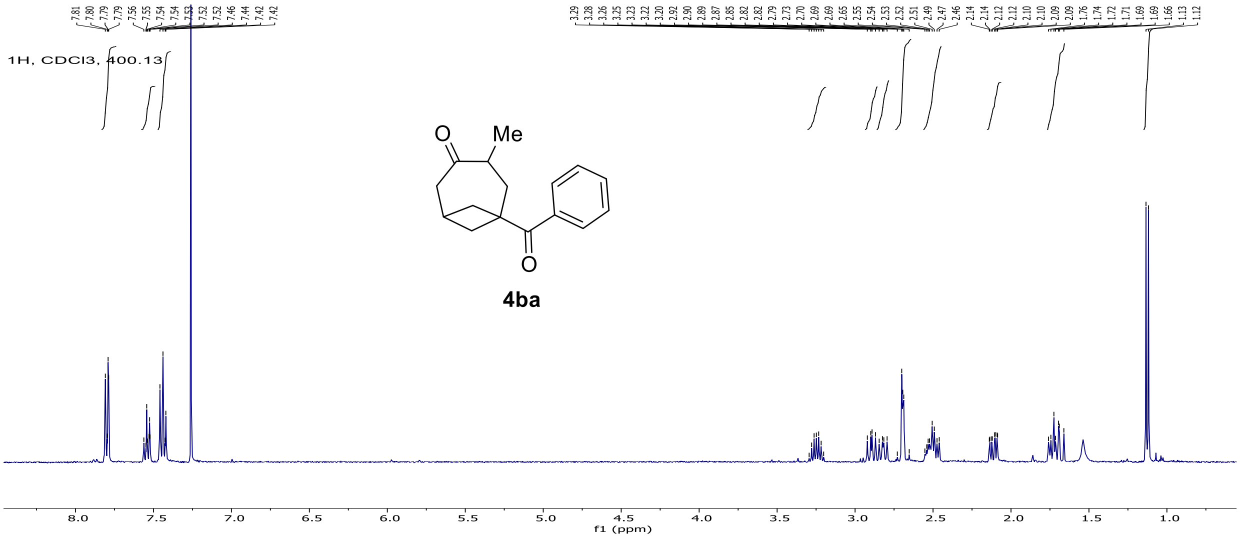

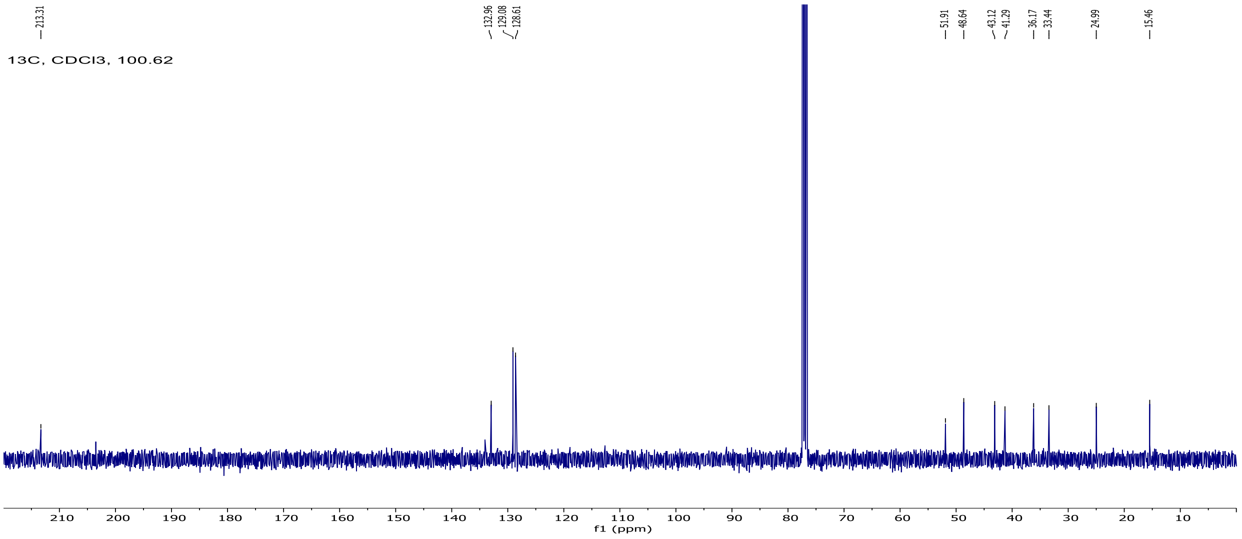
213.31
132.96
129.08
128.61
51.91
48.64
43.12
41.29
36.17
33.44
24.99
15.46
13C, CDCl3, 100.62
f1 (ppm)

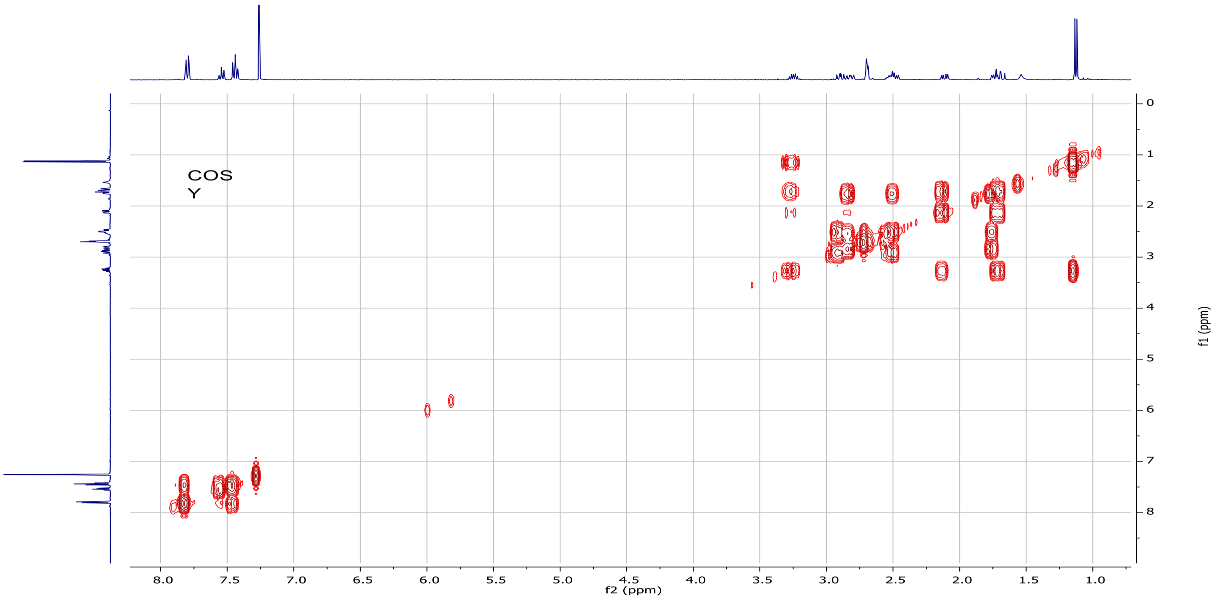
COS
Y
f2 (ppm)
f1 (ppm)

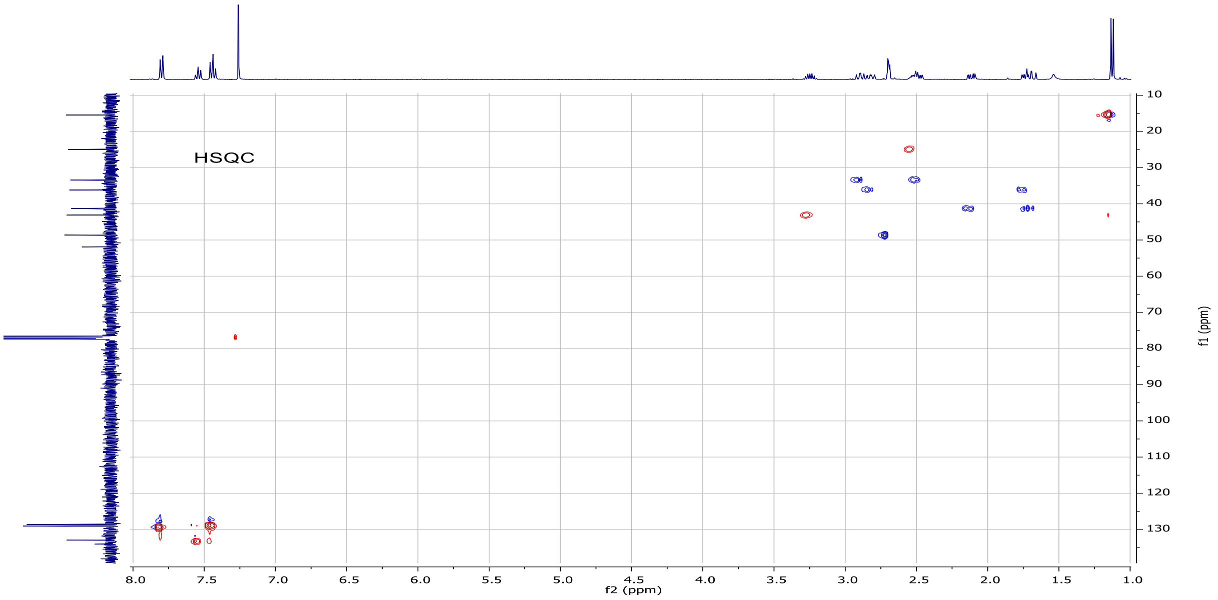
HSQC
f2 (ppm)
f1 (ppm)

6-(4-methoxybenzoyl)-4-methylbicyclo[4.1.1]octan-3-one (4ca)

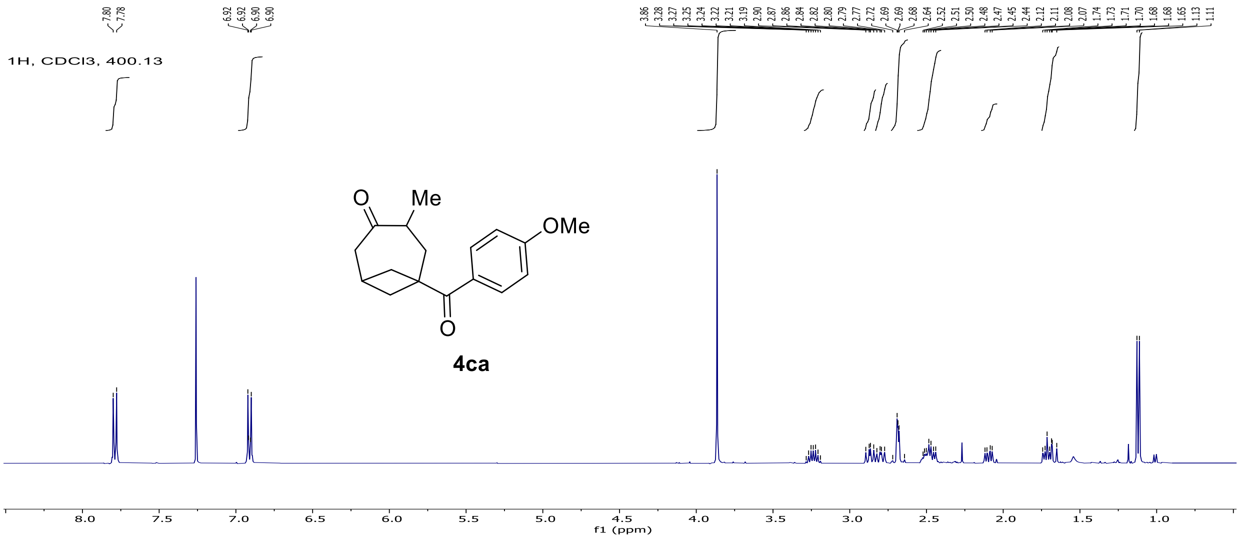

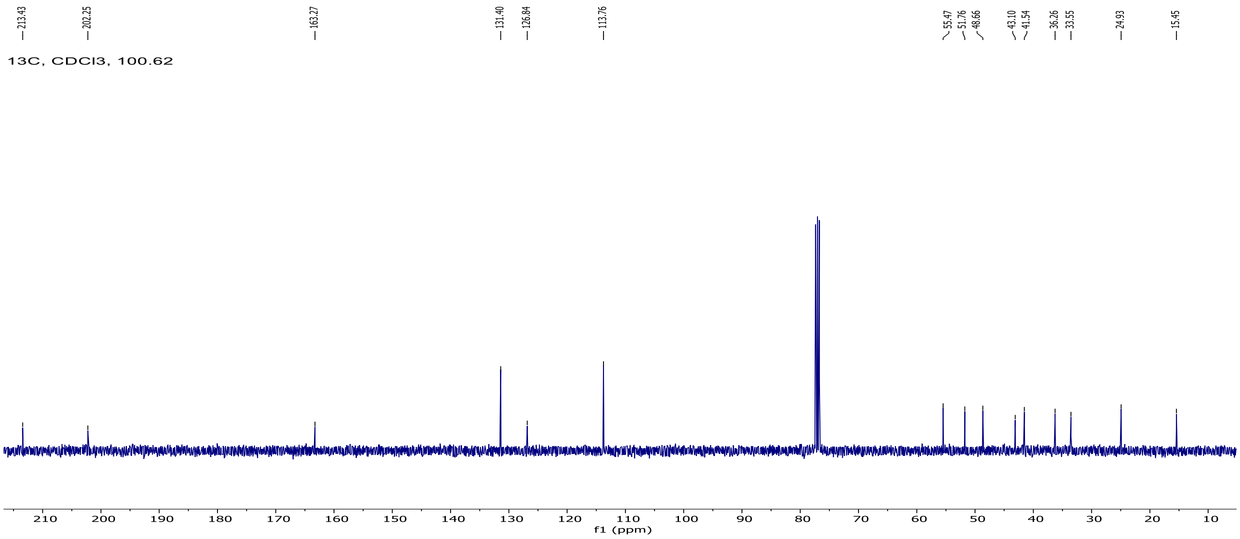

6-(3-Methoxybenzoyl)-4-methylbicyclo[4.1.1]octan-3-one (4da)

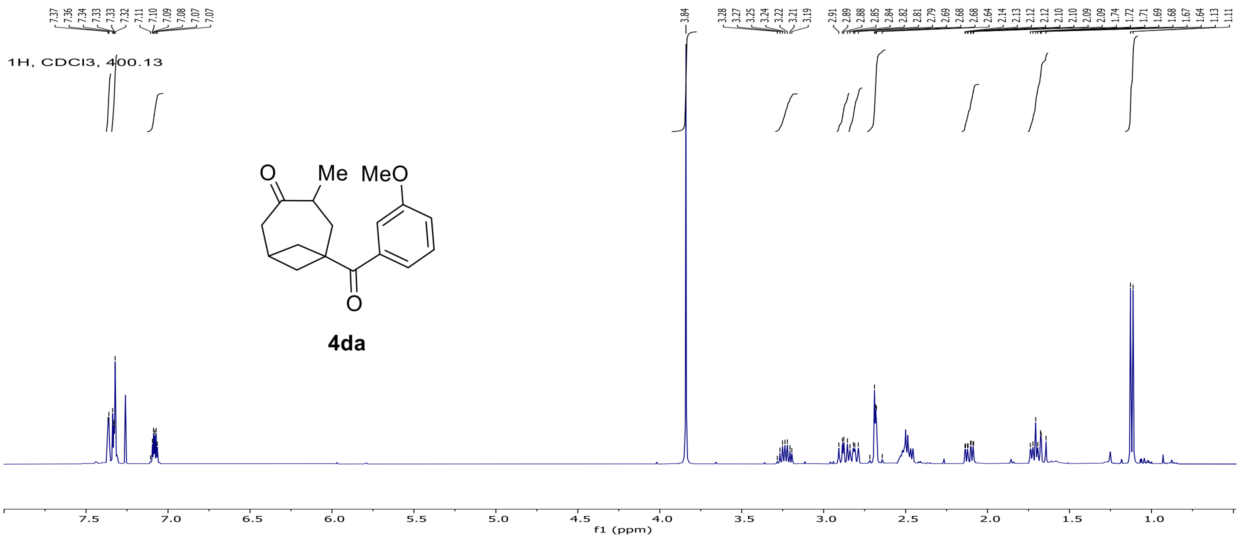

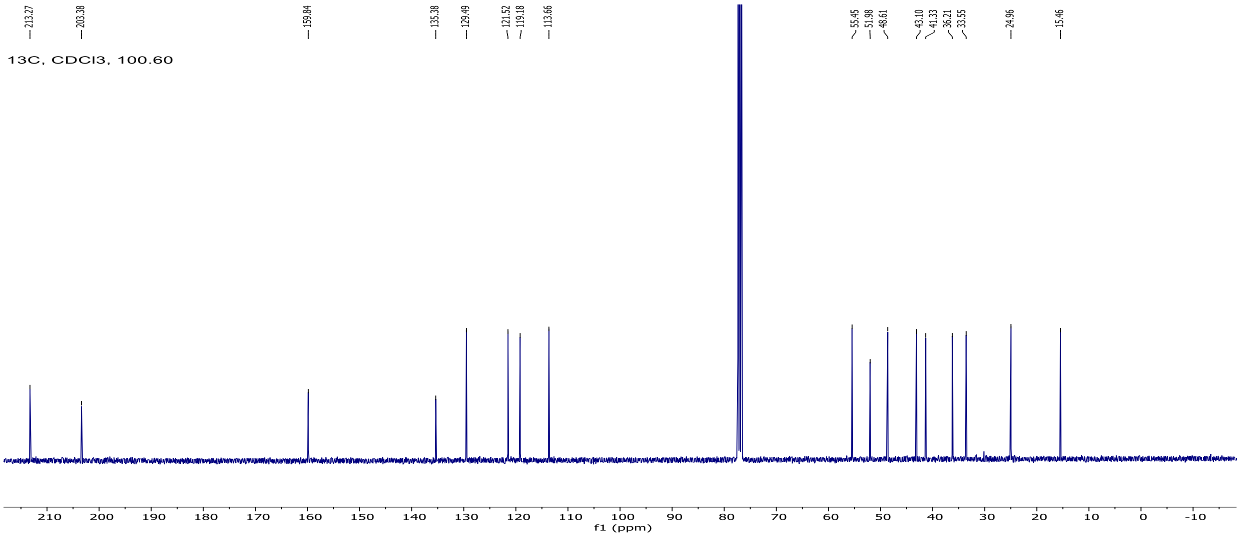

6-(2-Methoxybenzoyl)-4-methylbicyclo[4.1.1]octan-3-one (4ea)

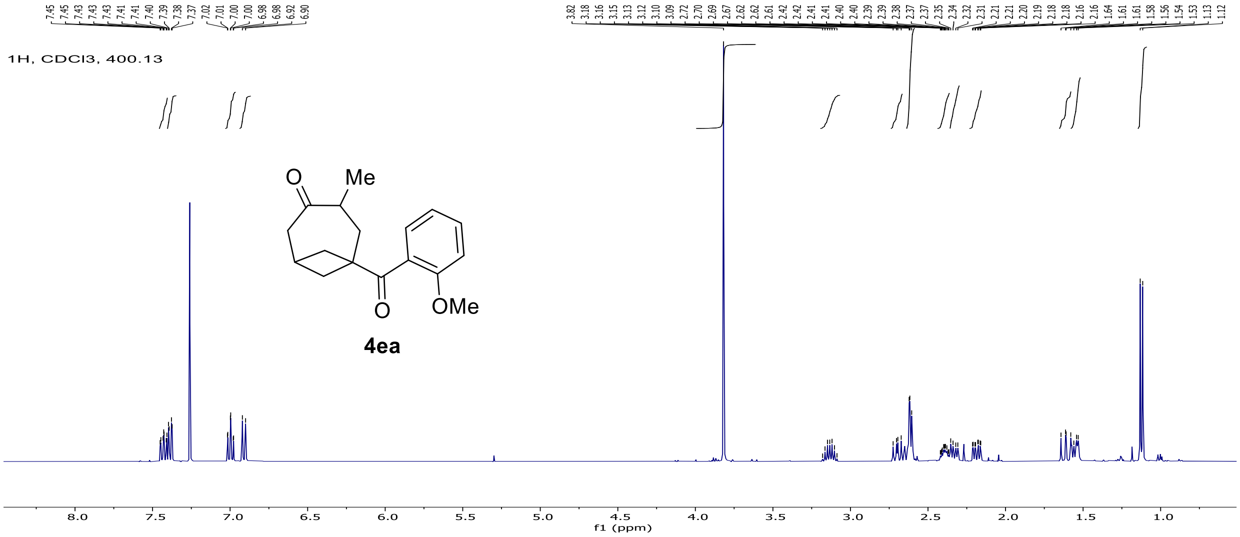

4ea

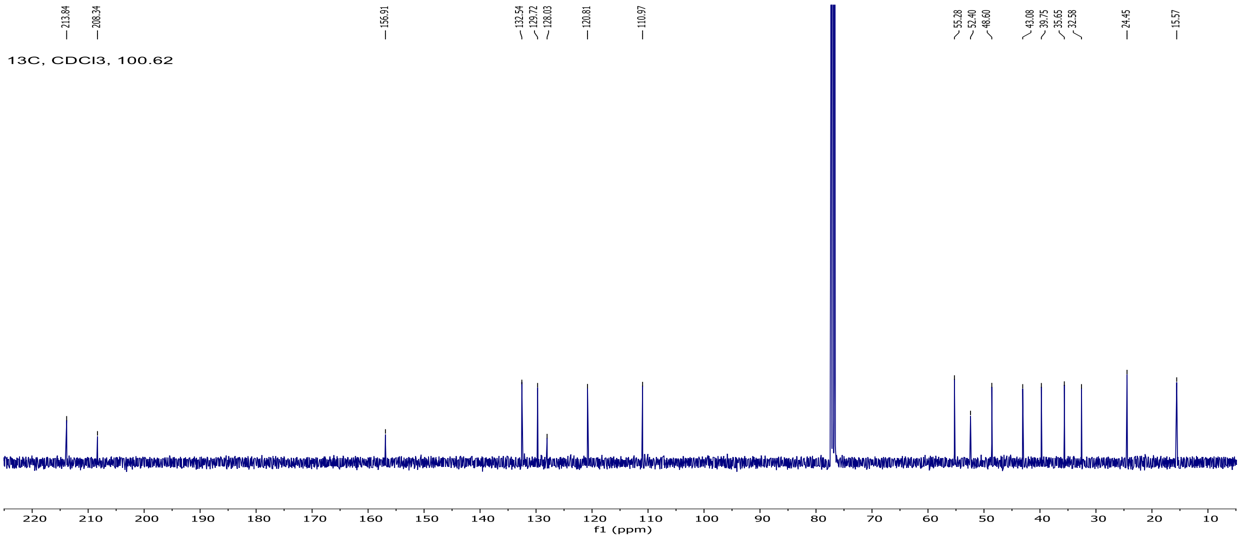

6-(4-bromobenzoyl)-4-methylbicyclo[4.1.1]octan-3-one (4fa)

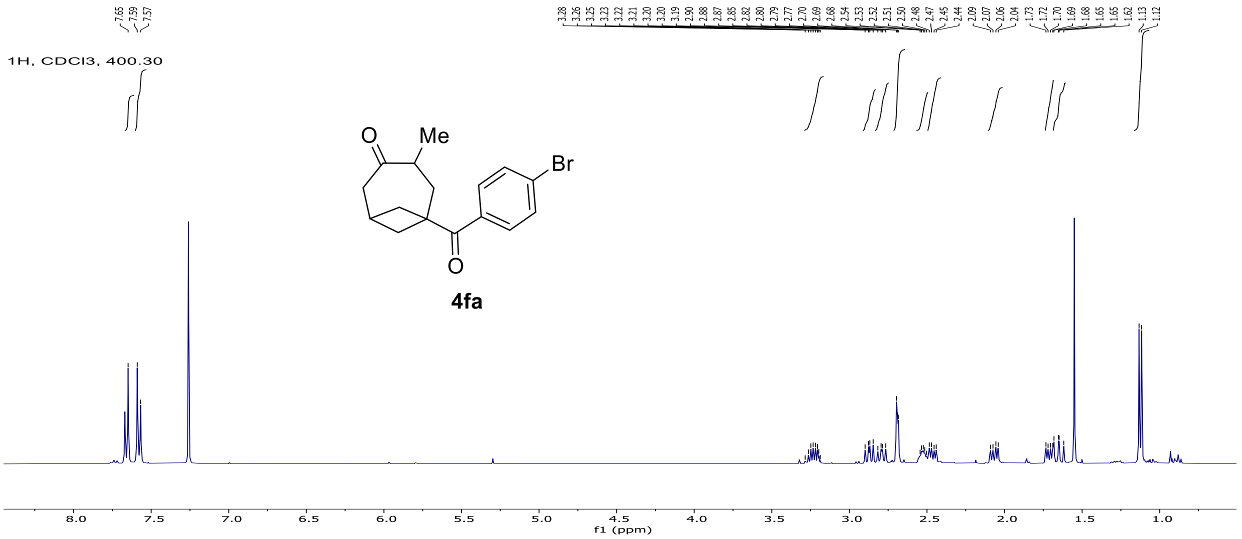

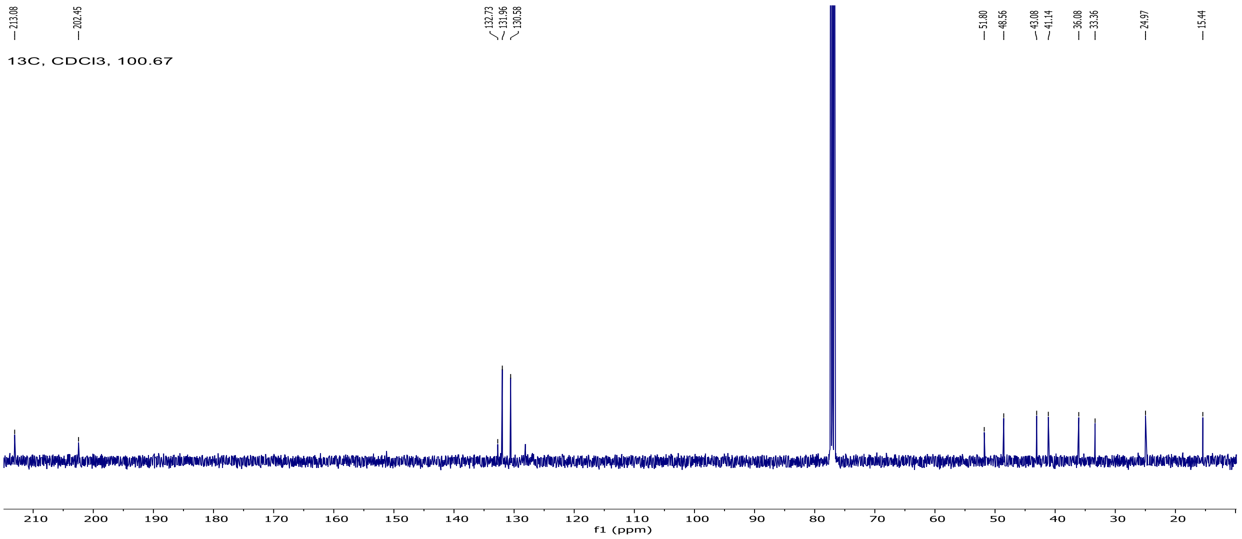

4-Methyl-6-(4-(trifluoromethyl)benzoyl)bicyclo[4.1.1]octan-3-one (4ga)

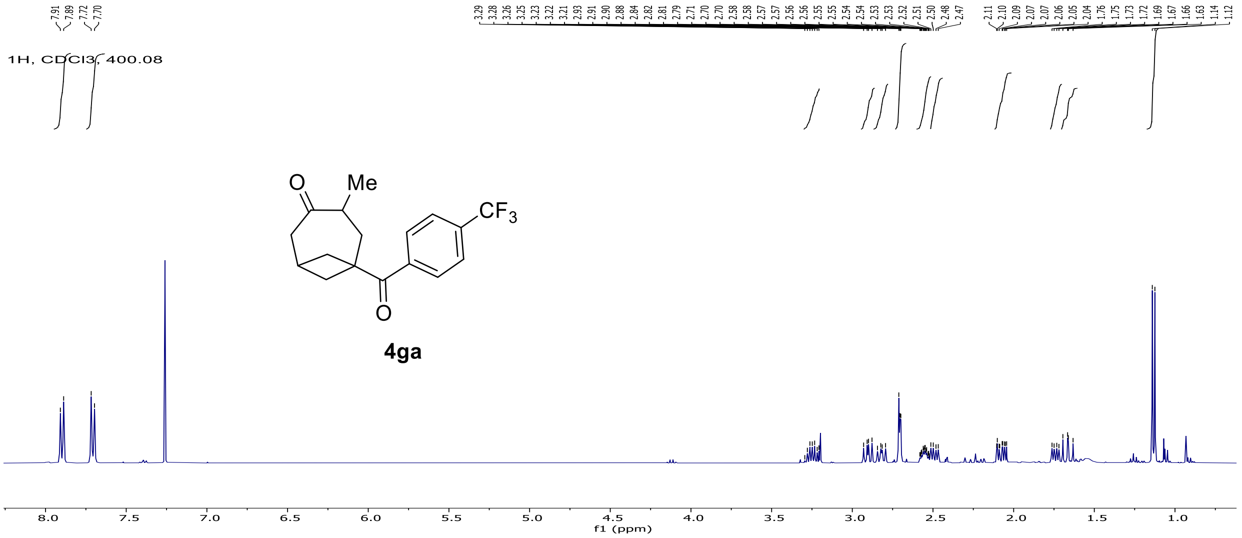

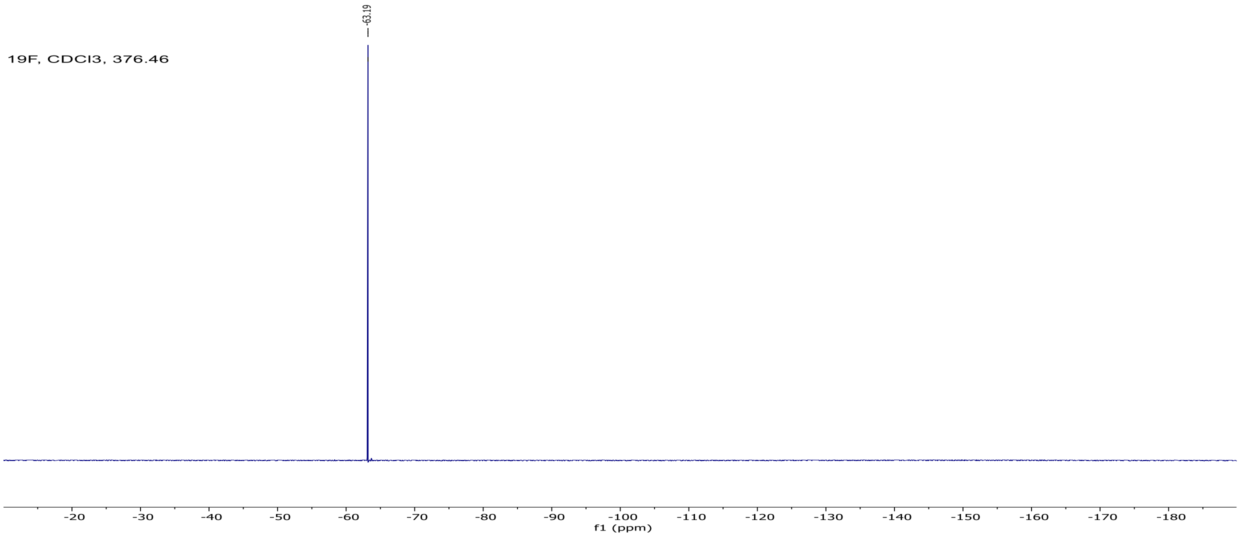

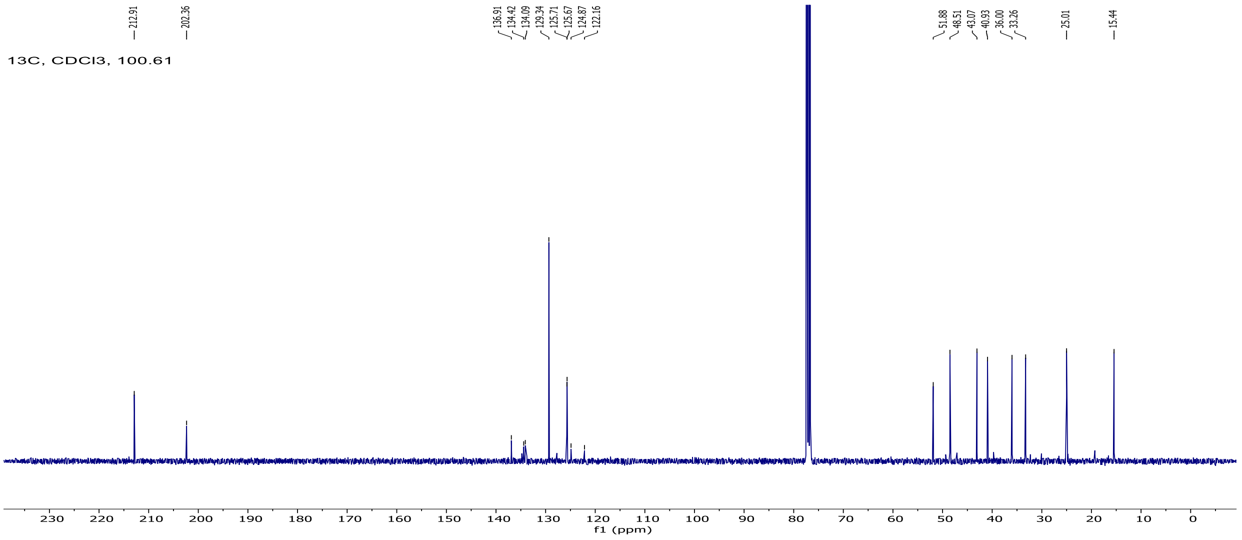

4-(3-Methyl-4-oxobicyclo[4.1.1]octane-1-carbonyl)benzonitrile (4ha)

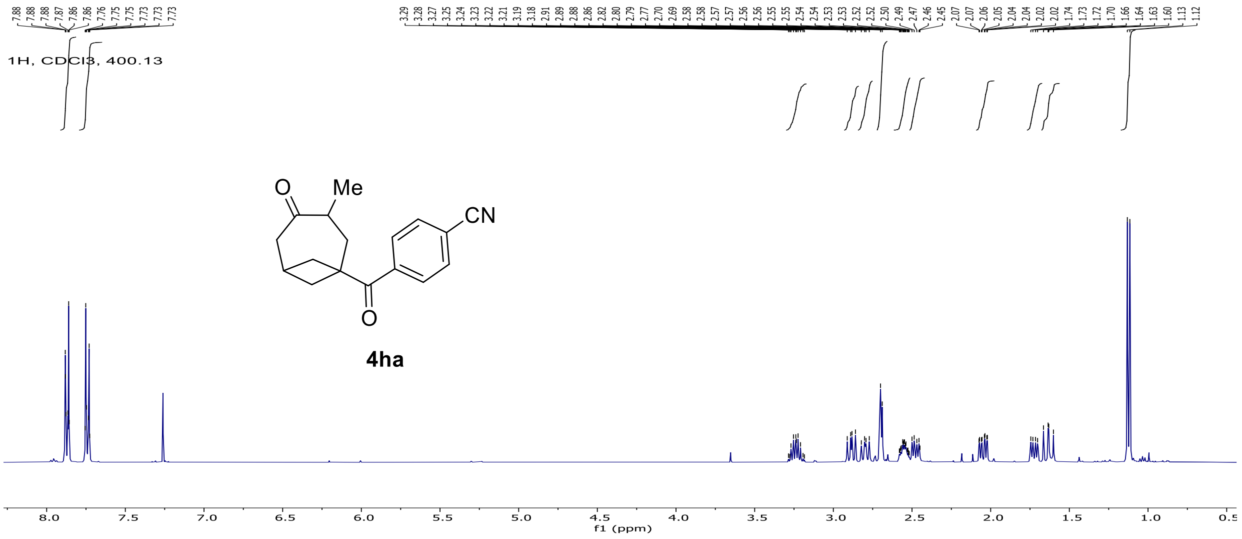

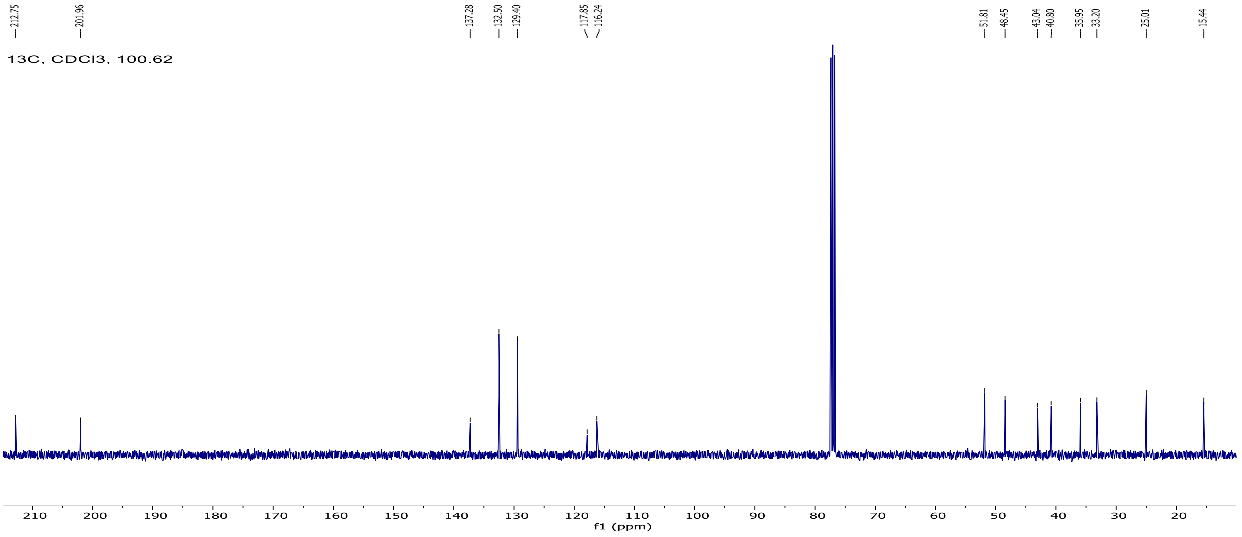

6-(5-Bromofuran-2-carbonyl)-4-methylbicyclo[4.1.1]octan-3-one (4ia)

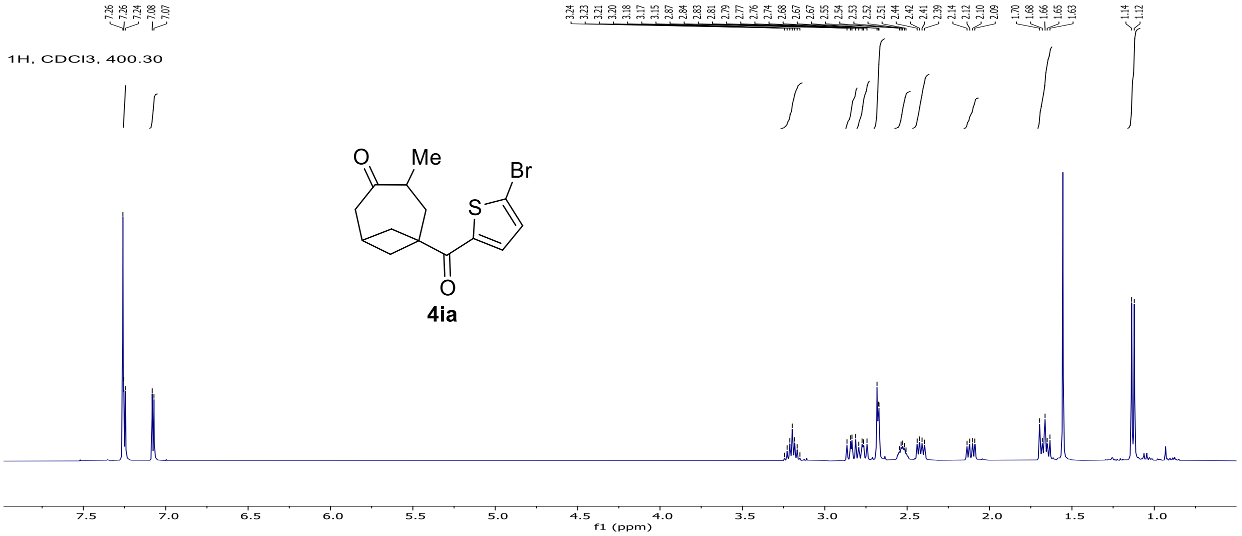

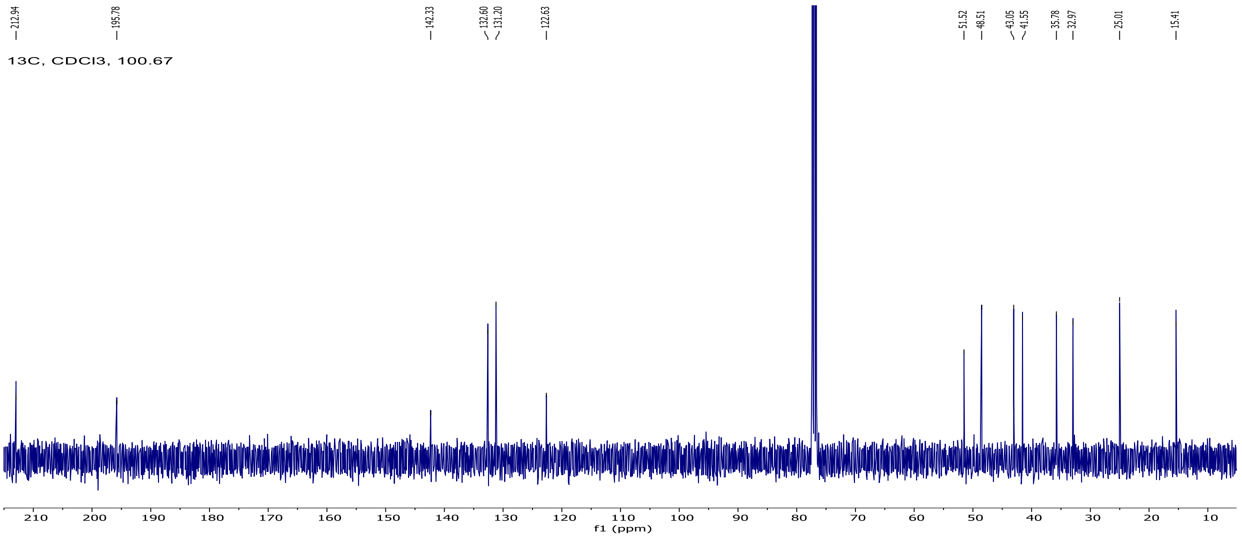

6-(2-Naphthoyl)-1,4-dimethylbicyclo[4.1.1]octan-3-one (4ja)

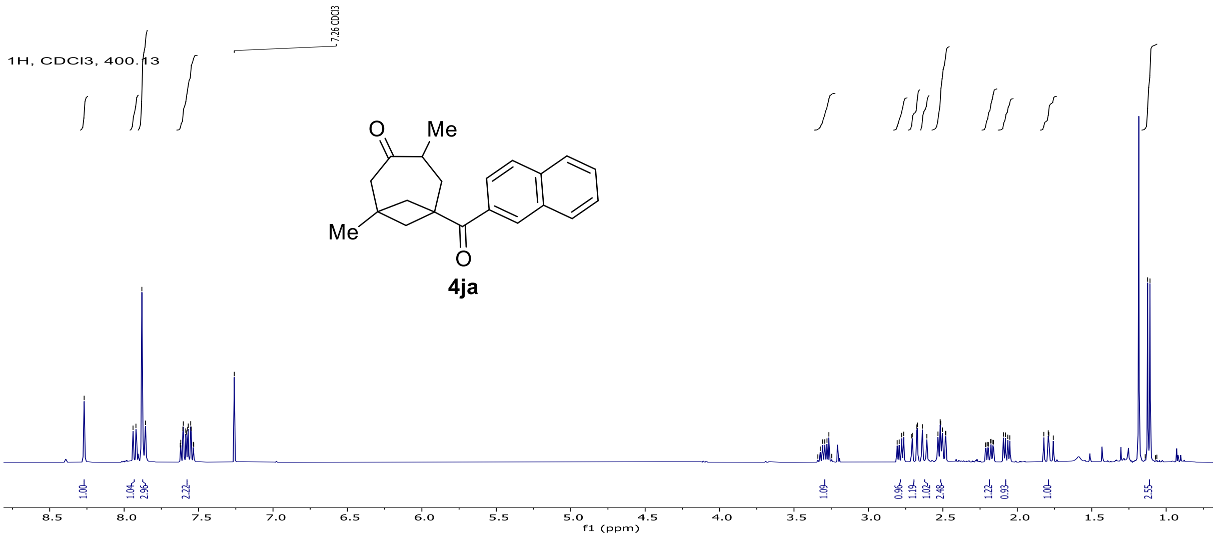

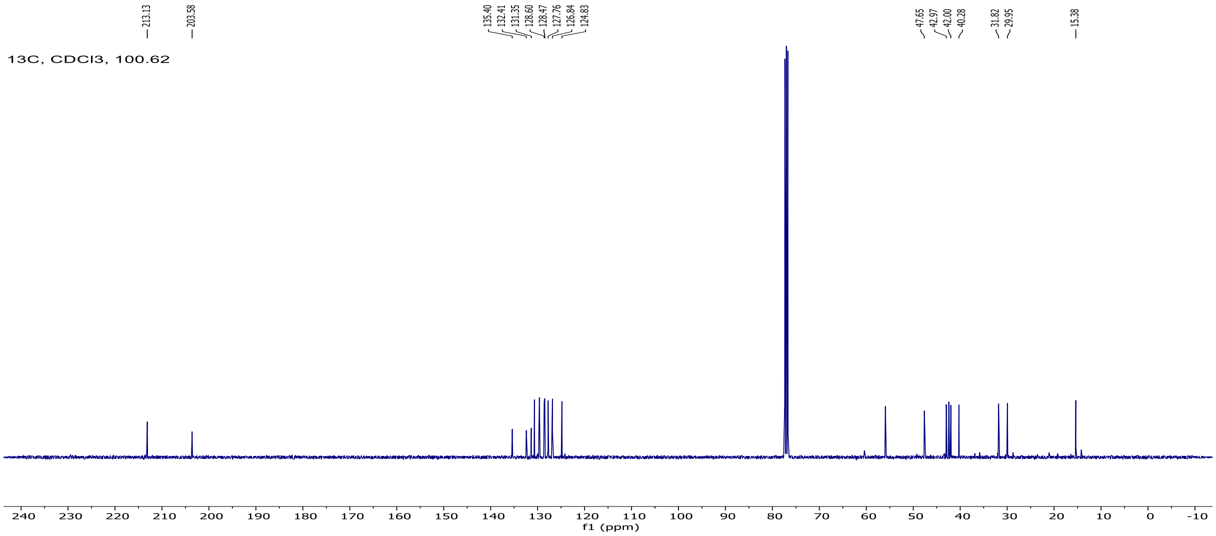

Naphthalen-2-yl(3-phenylbicyclo[1.1.0]butan-1-yl)methanone (4ka)

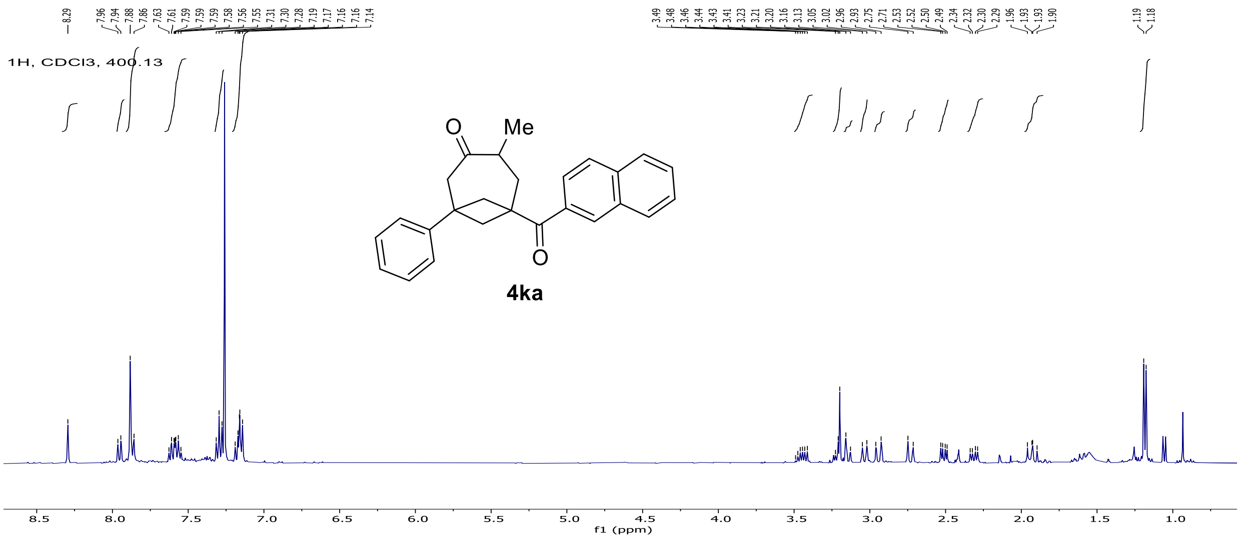

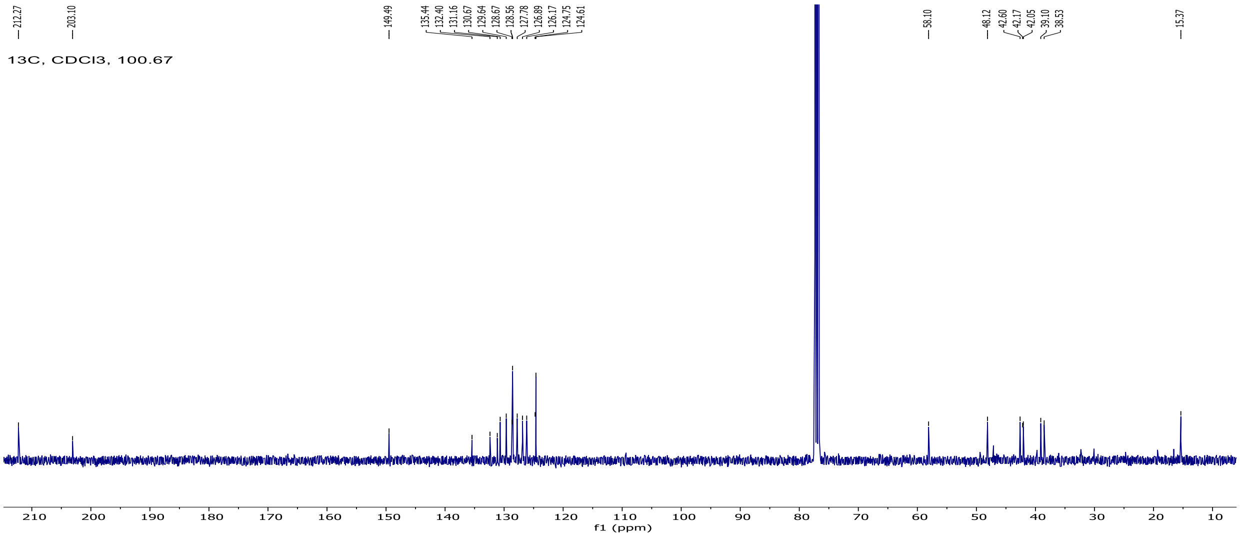

6-(2-Naphthoyl)-1-(3,4-difluorophenyl)-4-methylbicyclo[4.1.1]octan-3-one (4la)

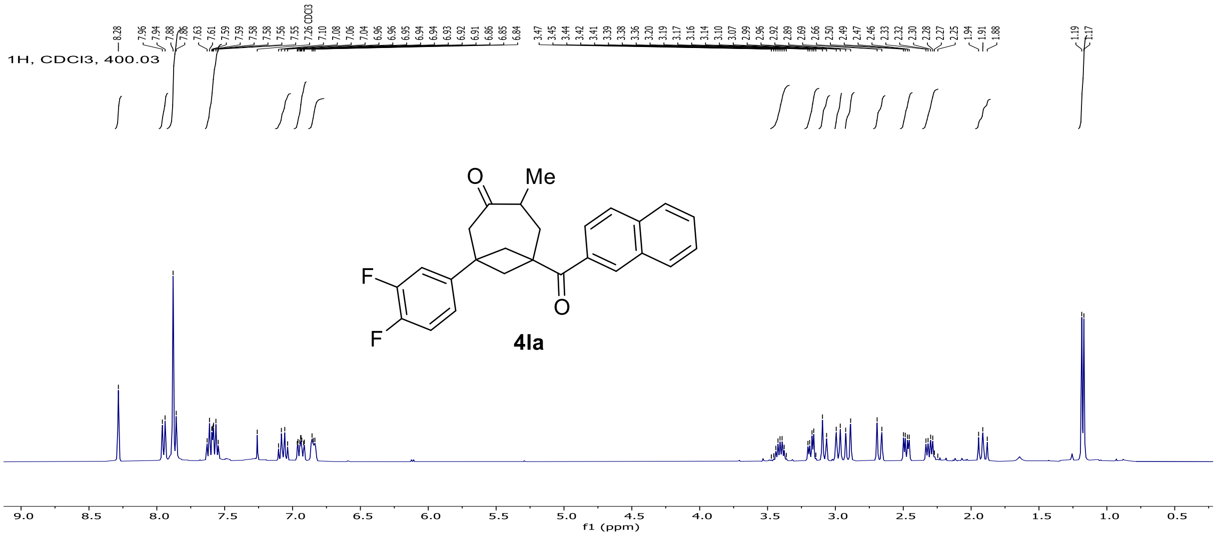

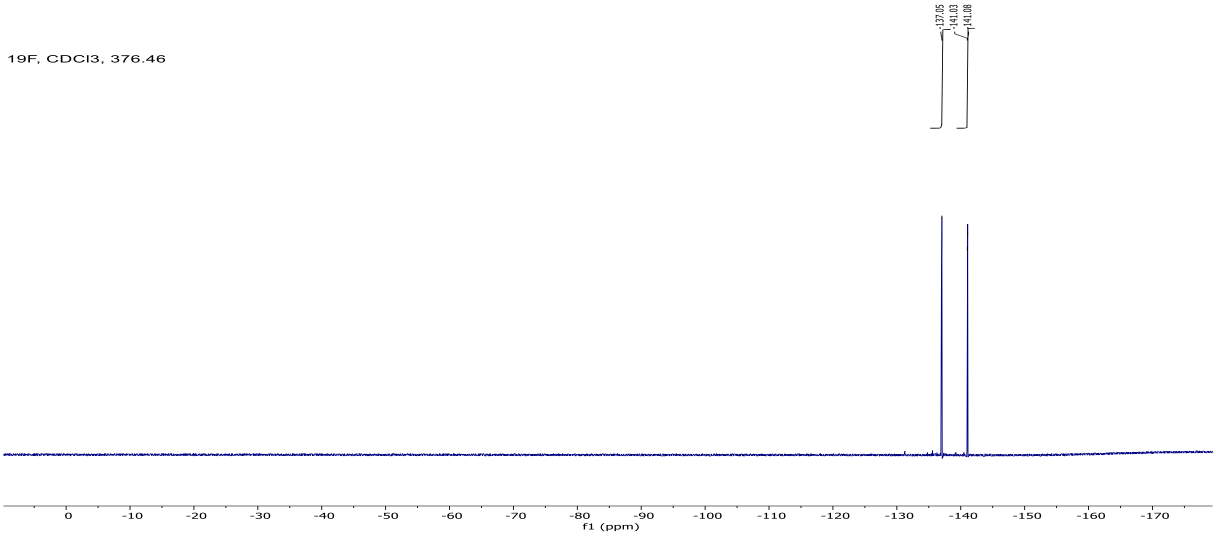

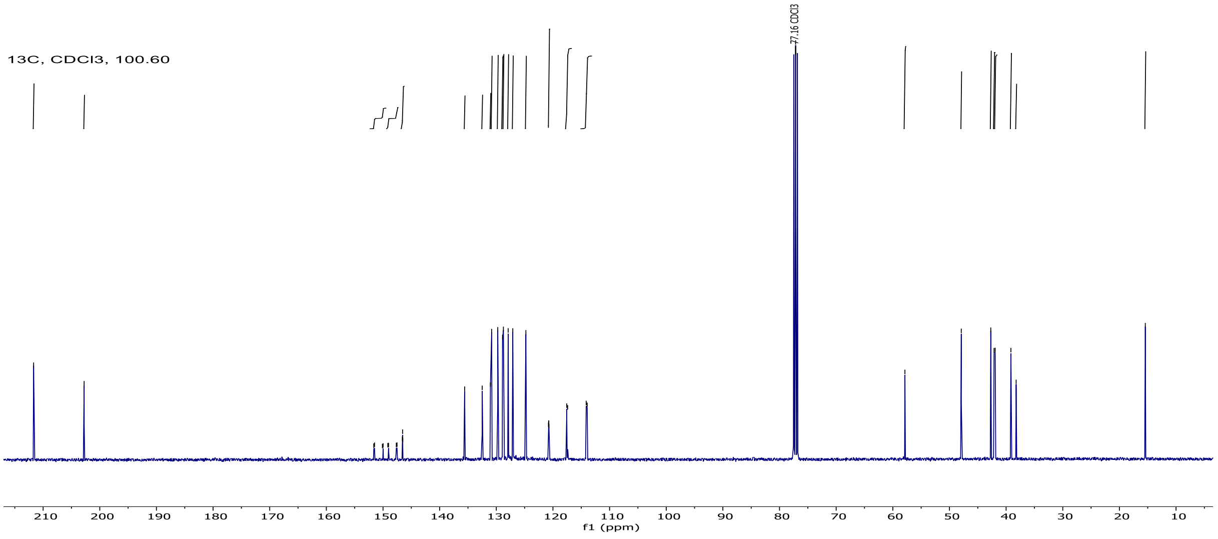

6-(2-Naphthoyl)-4-methyl-1-(4-(trifluoromethyl)phenyl)bicyclo[4.1.1]octan-3-one (4ma)

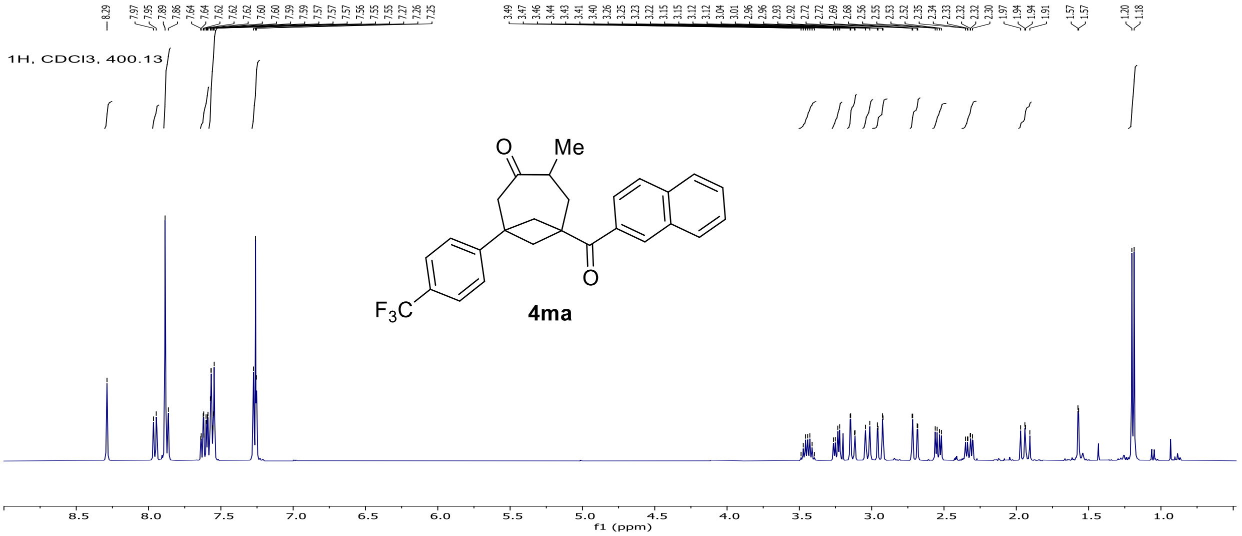

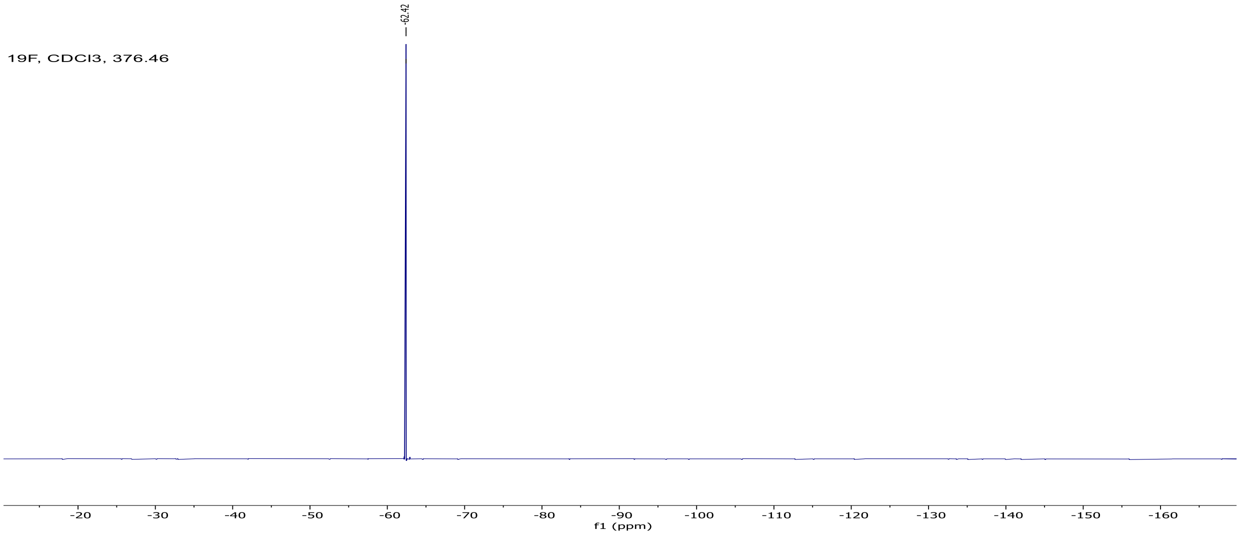

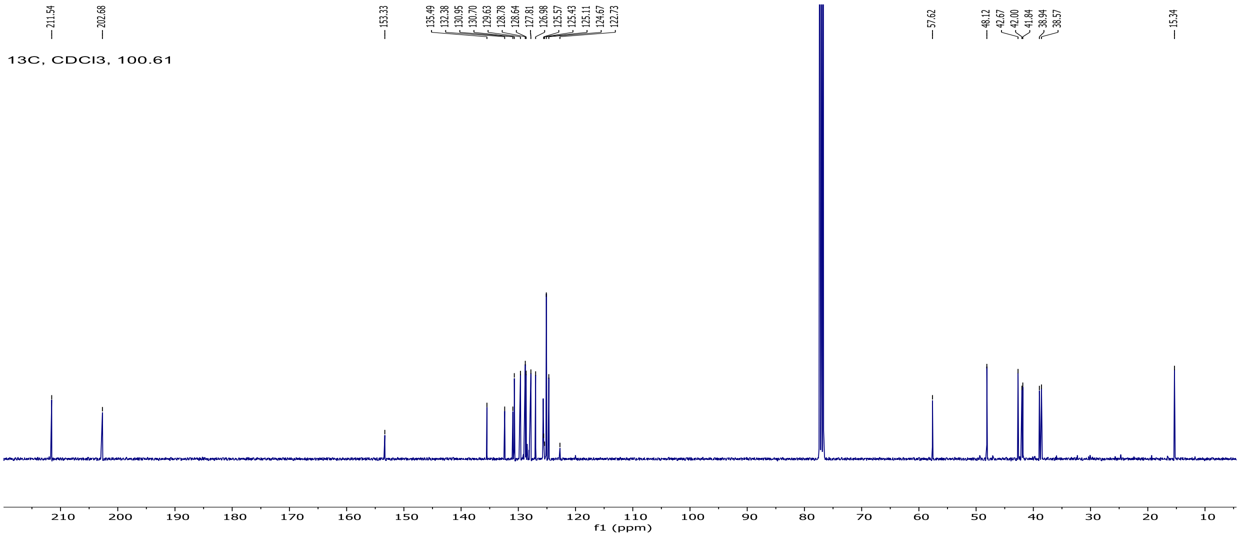

4-Methyl-6-pentanoylbicyclo[4.1.1]octan-3-one (4ma)

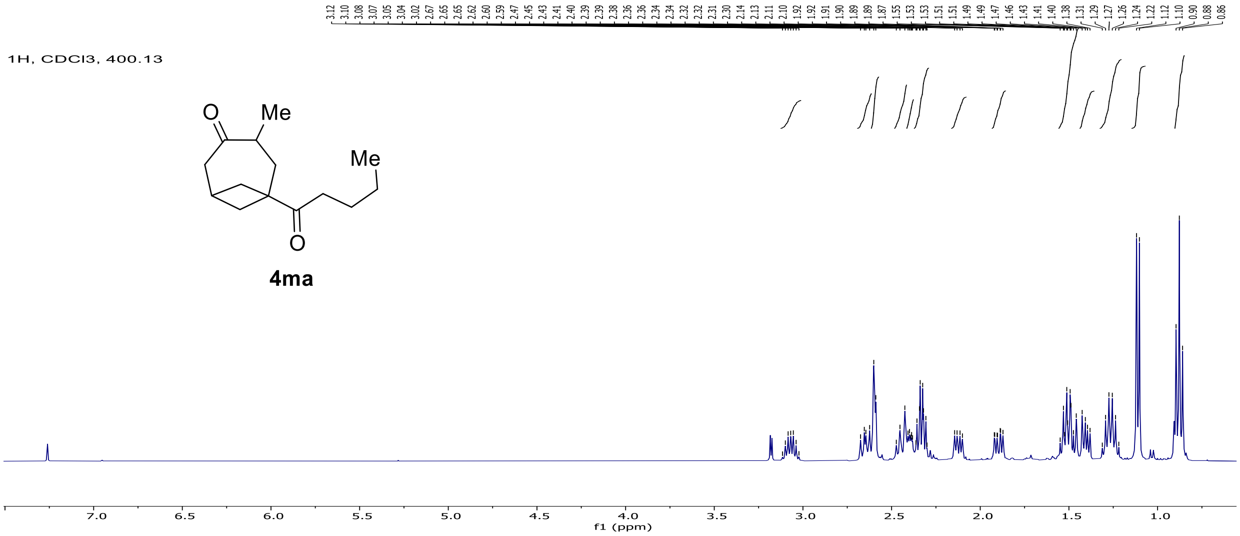

4ma

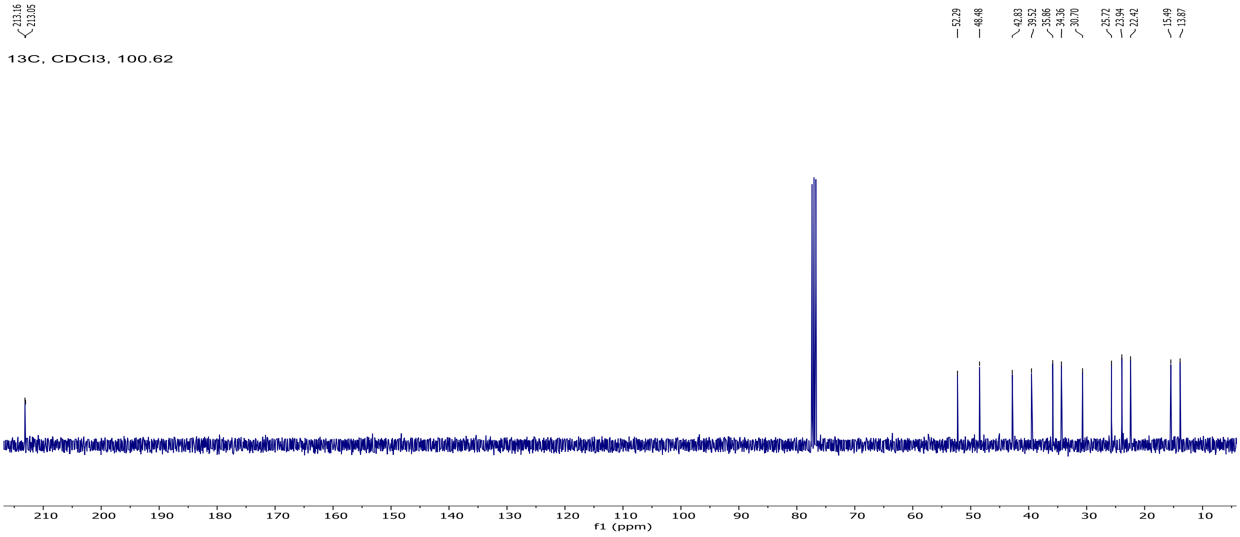

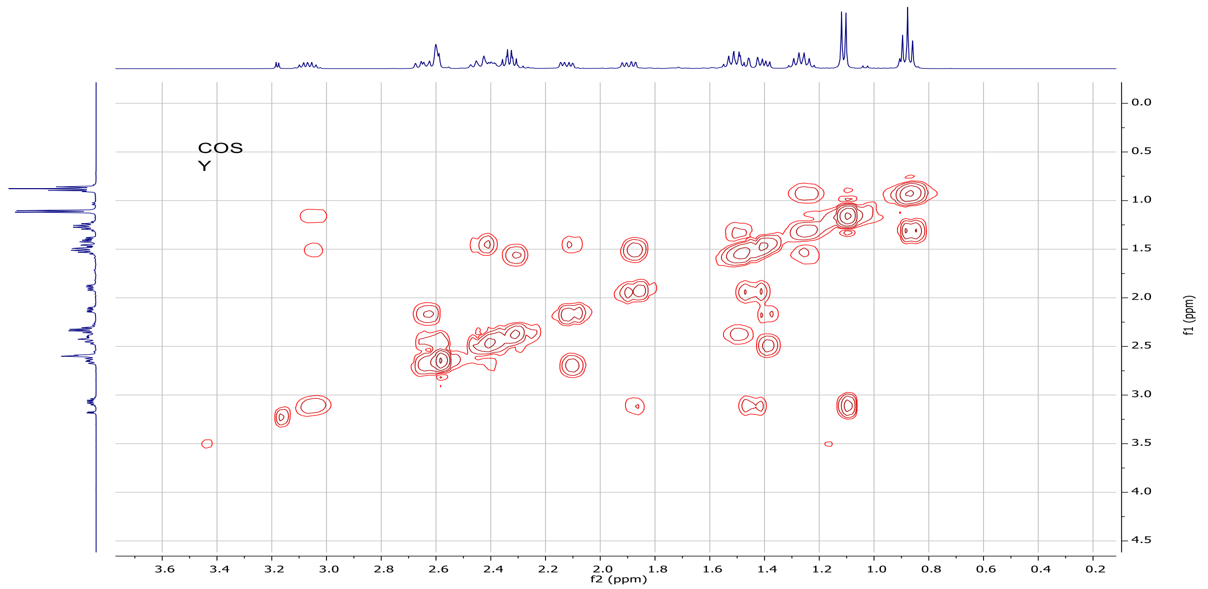

6-(Cyclohexanecarbonyl)-4-methylbicyclo[4.1.1]octan-3-one (4na)

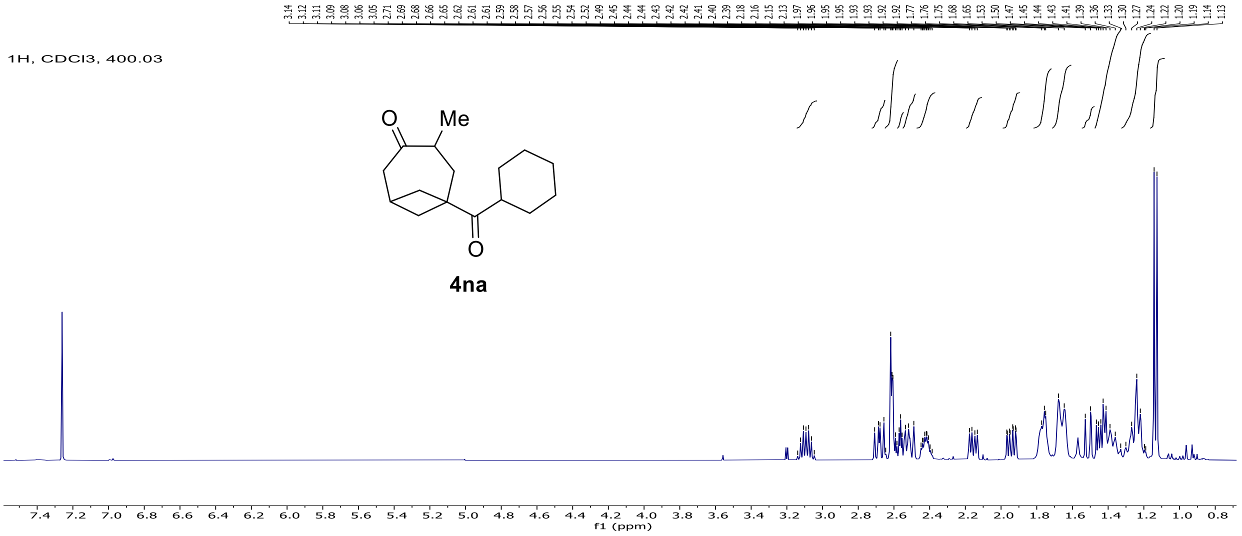

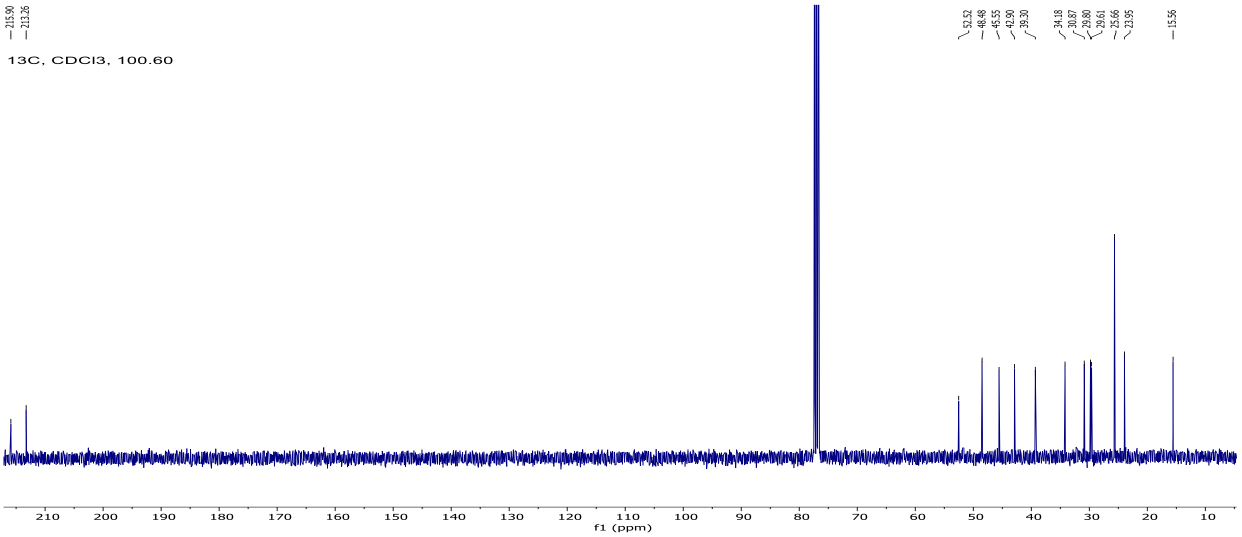

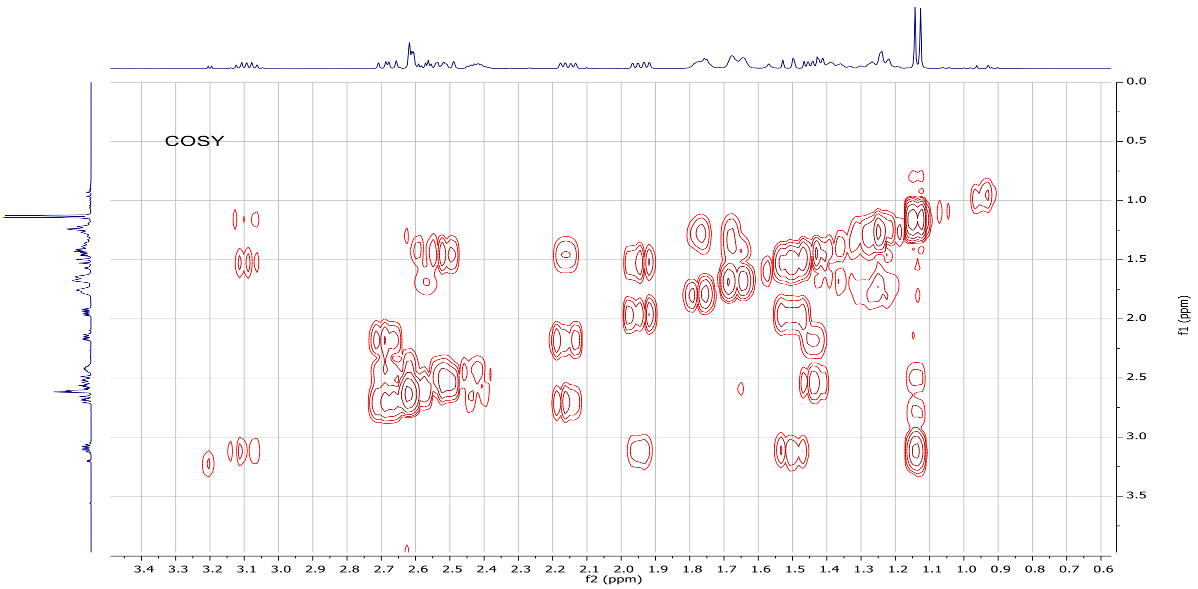

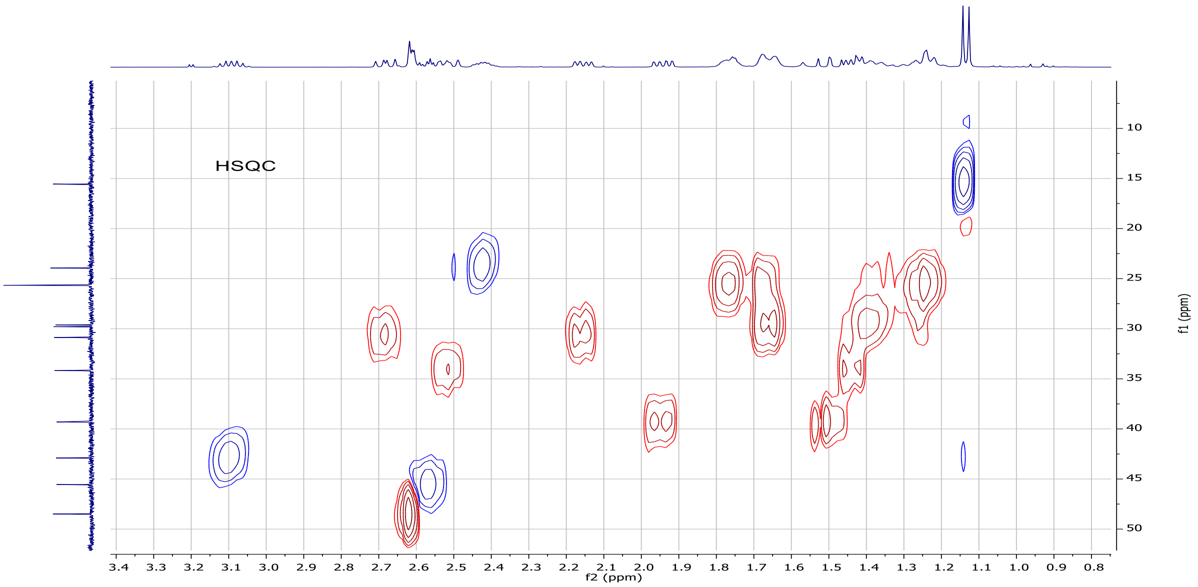

4-Methyl-6-pivaloylbicyclo[4.1.1]octan-3-one (4oa)

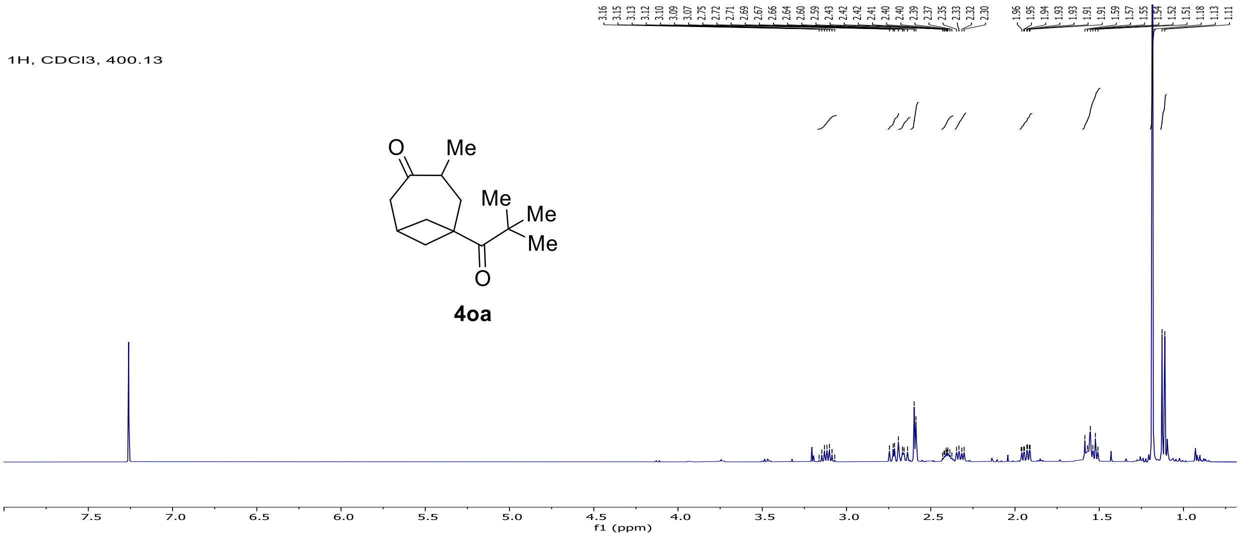

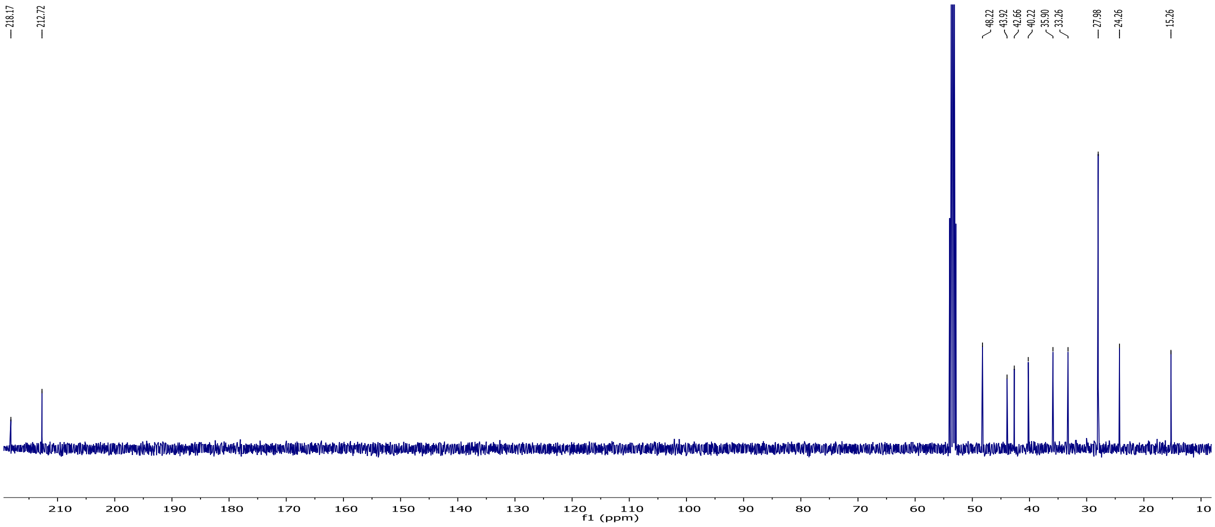

6-(2-naphthoyl)bicyclo[4.1.1]octan-3-one (4ab)

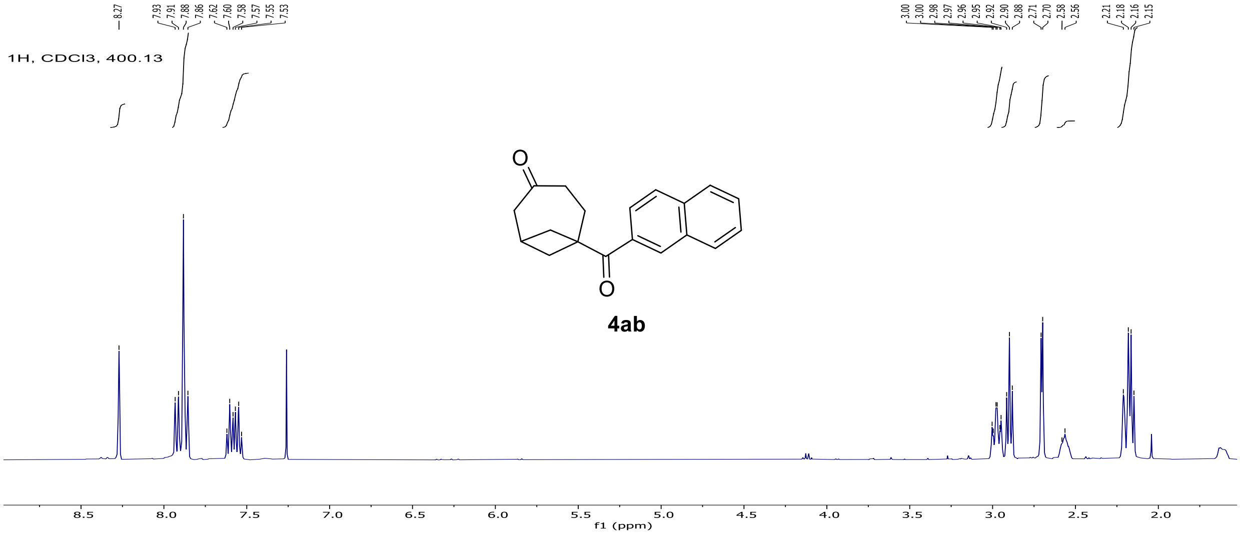

4ab

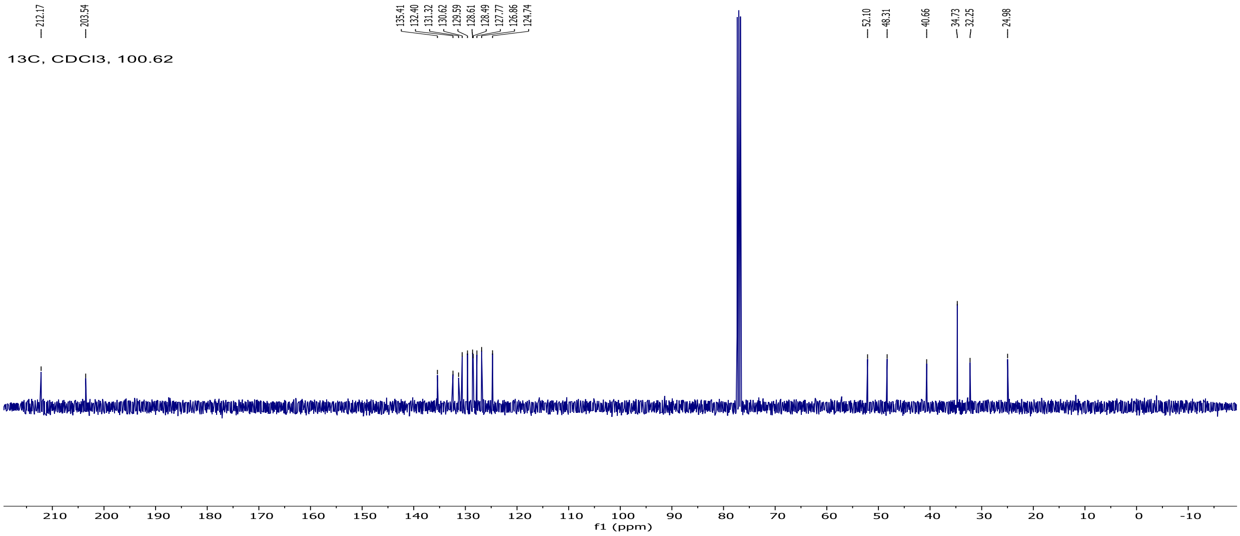

6-(2-Naphthoyl)-2-methylbicyclo[4.1.1]octan-3-one (4ac)

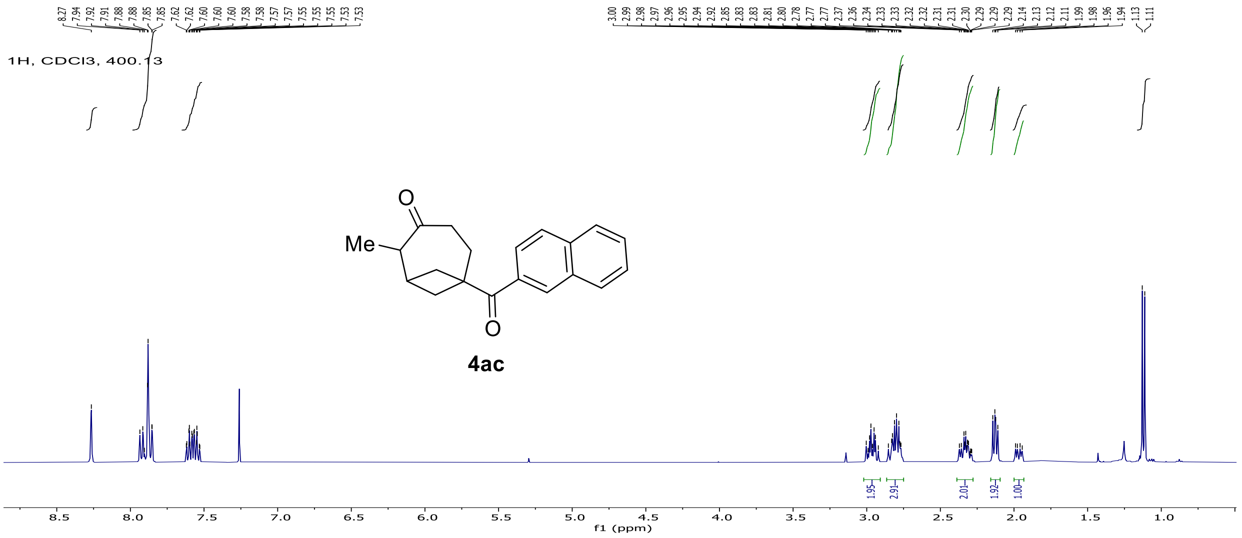

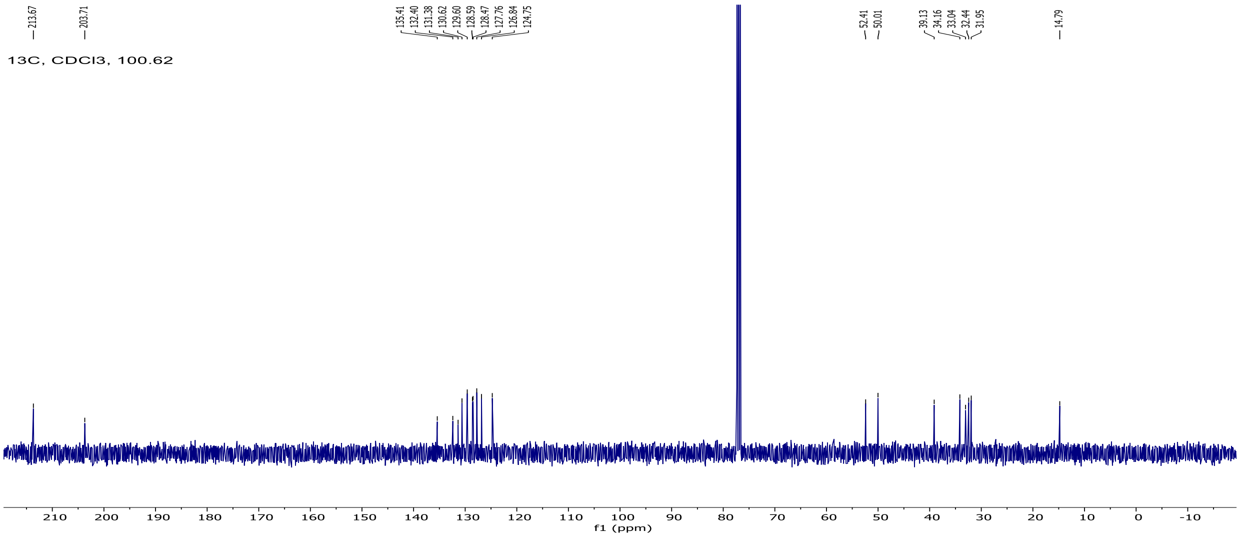

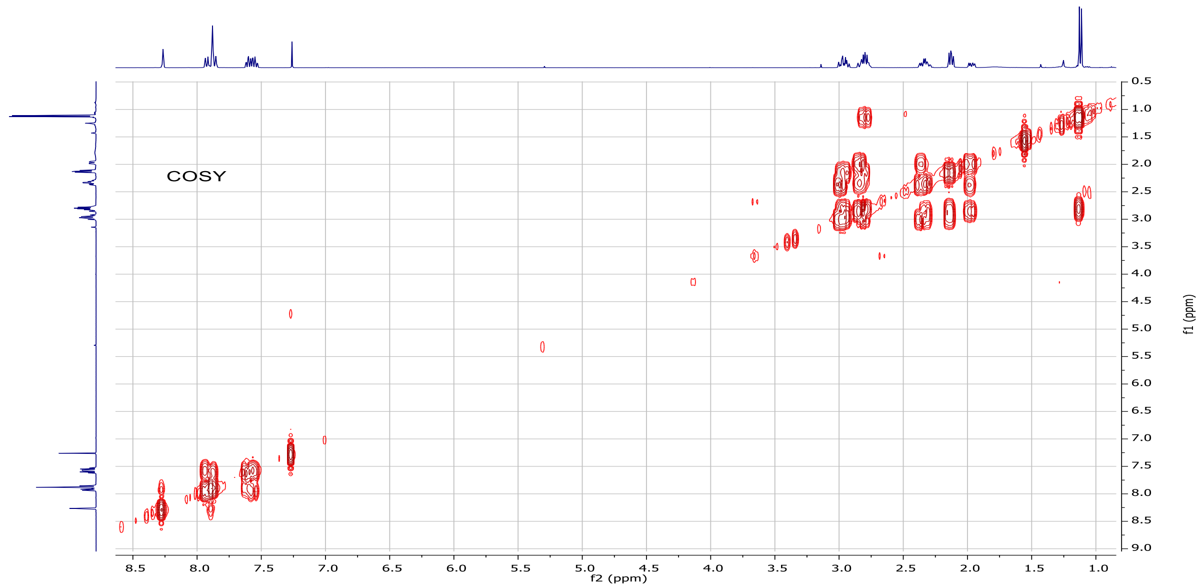

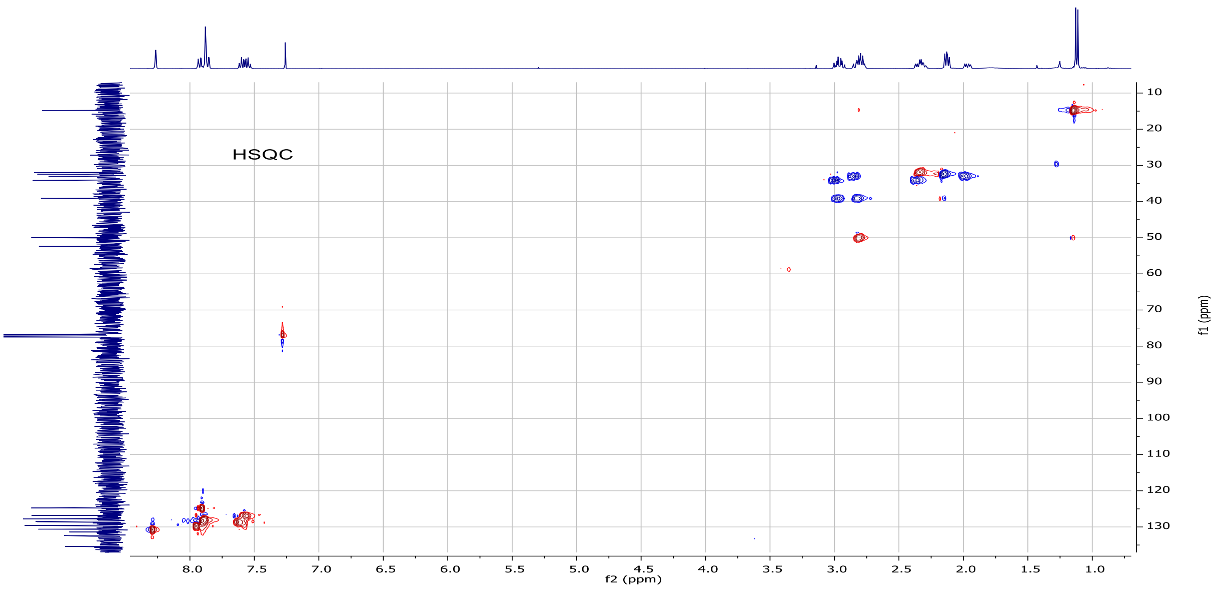

6-(2-Naphthoyl)-2,4-trans-dimethylbicyclo[4.1.1]octan-3-one (4ad)

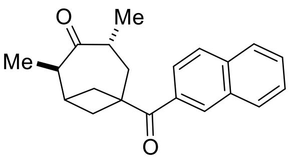

4ad

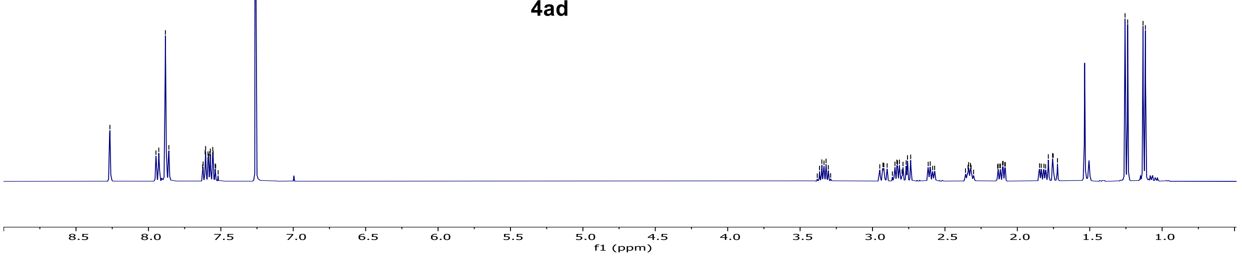

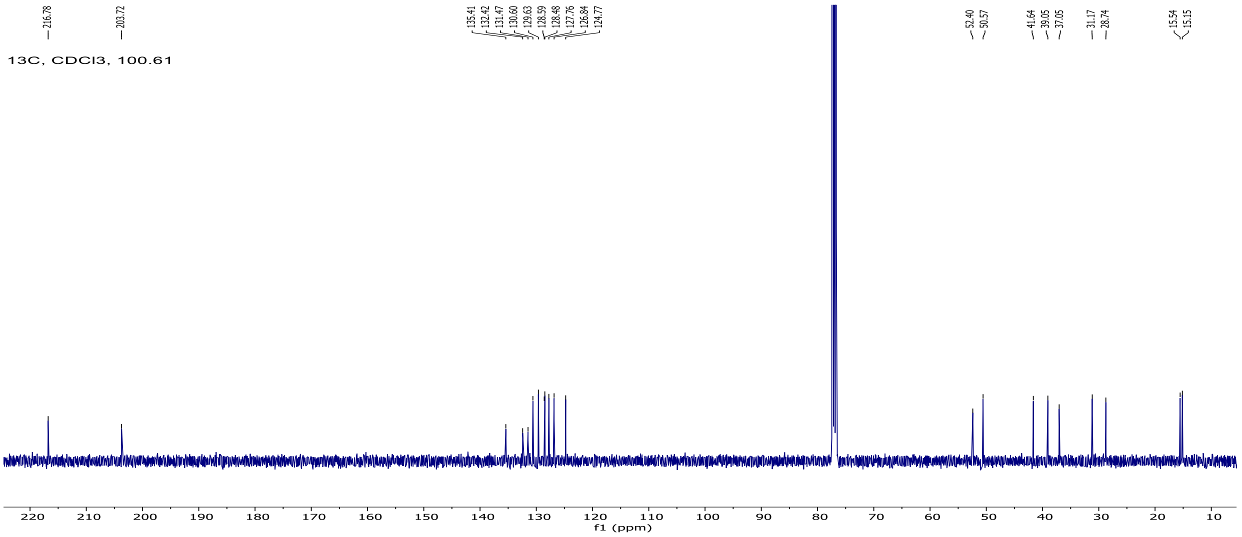

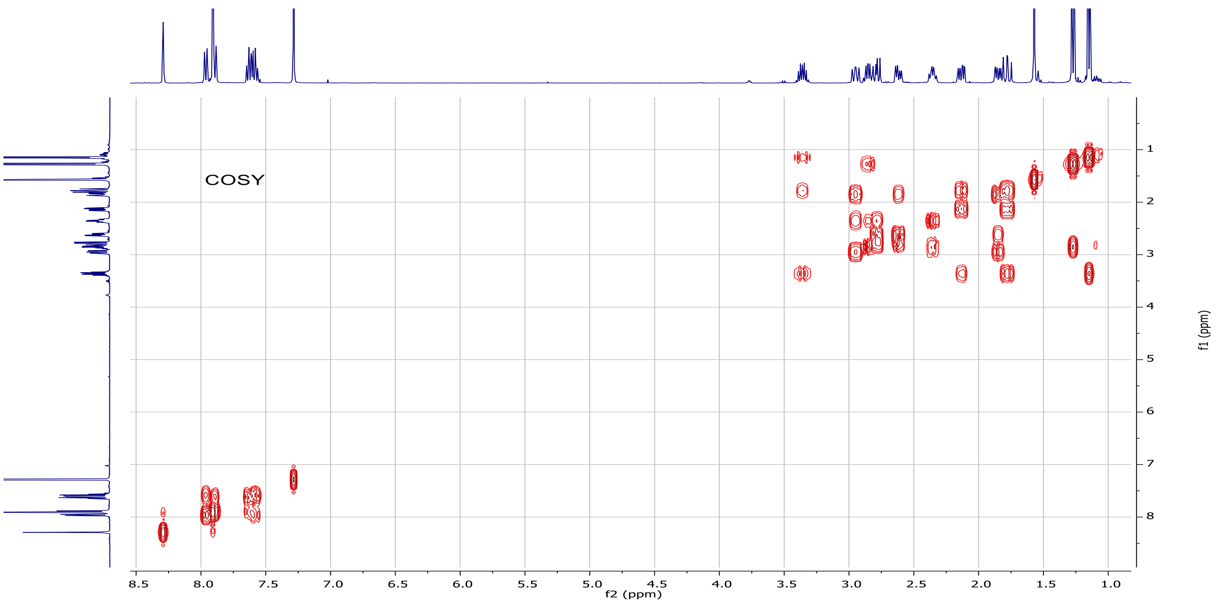
COSY
f2 (ppm)
f1 (ppm)

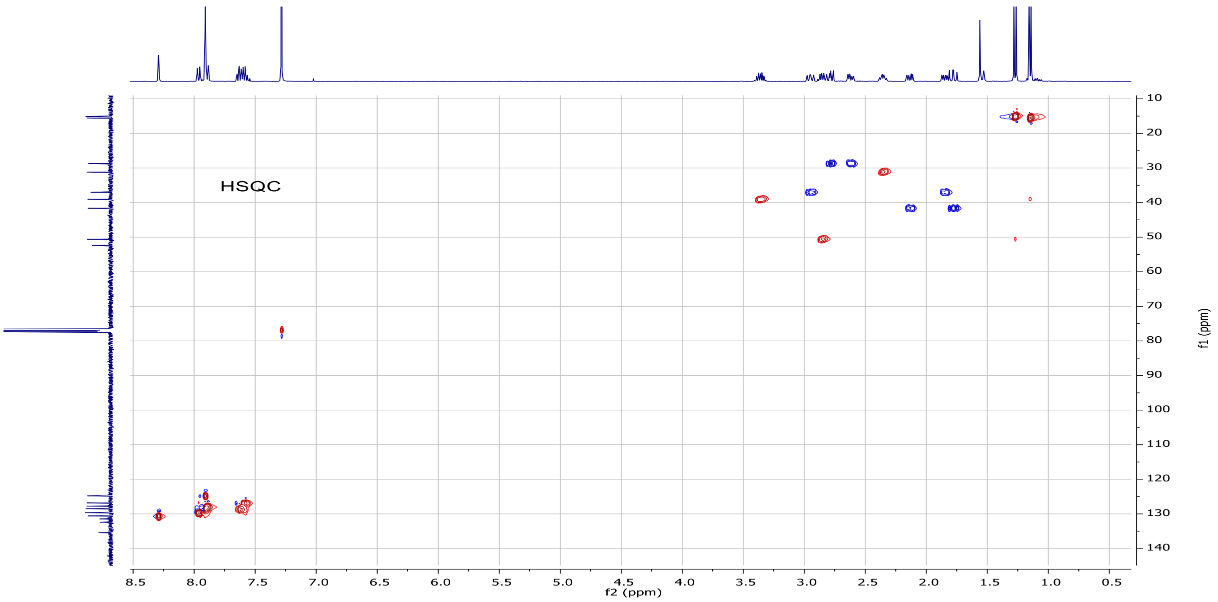
HSQC
f2 (ppm)
f1 (ppm)

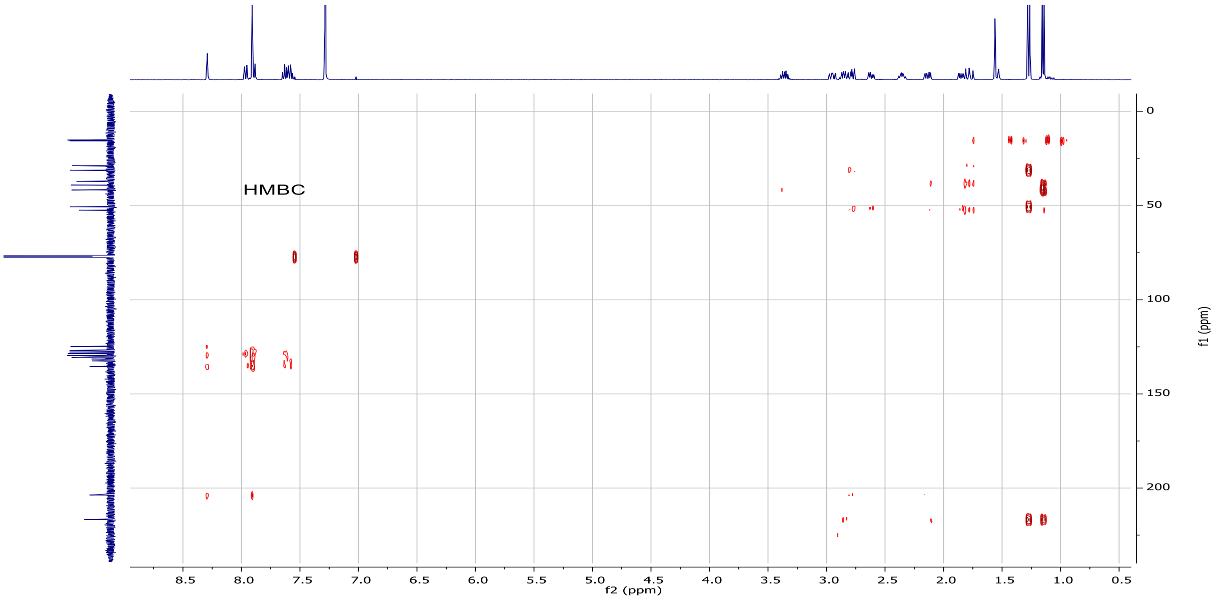
HMBC
f2 (ppm)
f1 (ppm)

6-(2-Naphthoyl)-4,5-trans-dimethylbicyclo[4.1.1]octan-3-one (4ae)

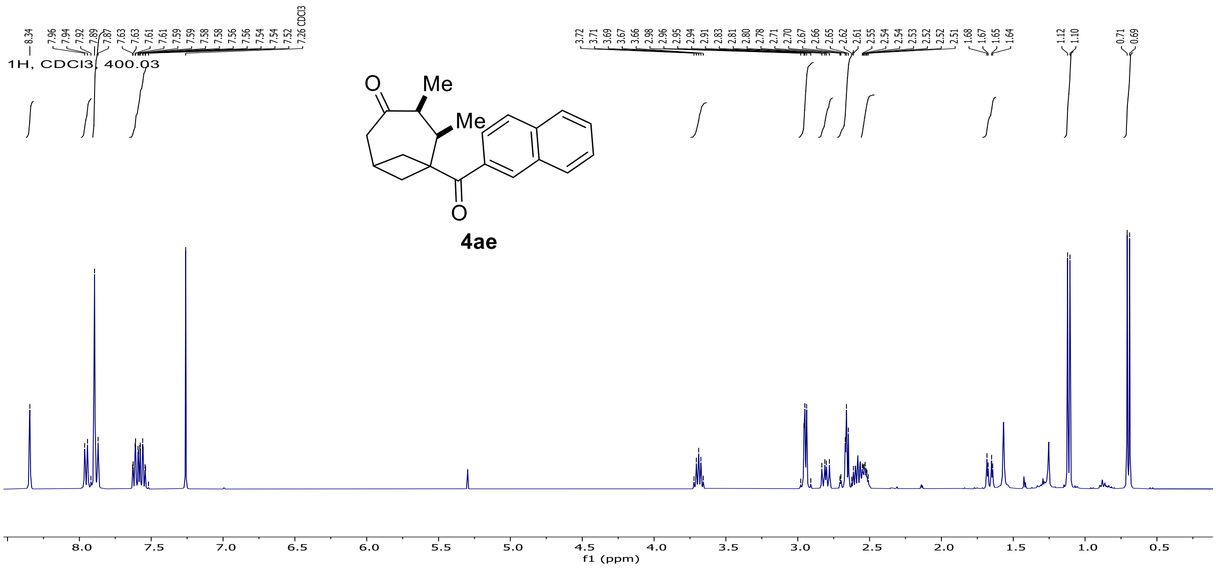

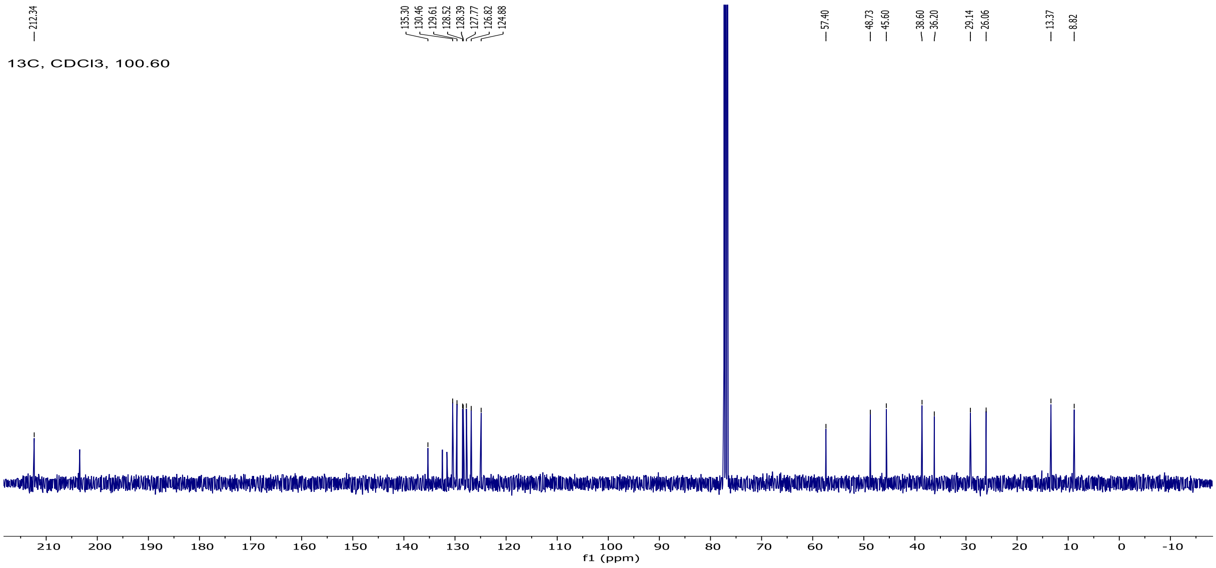

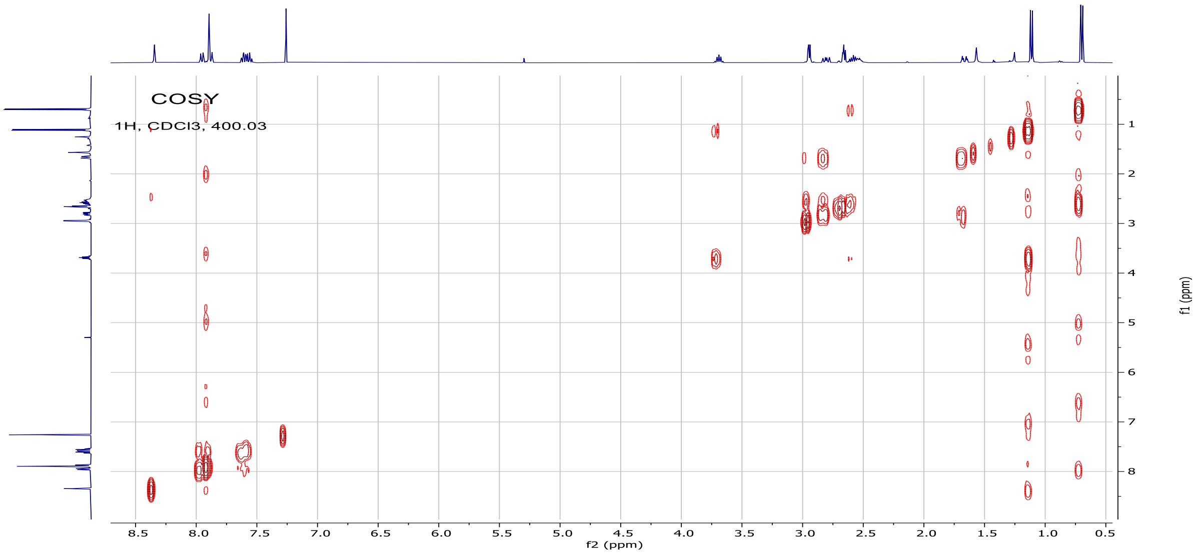

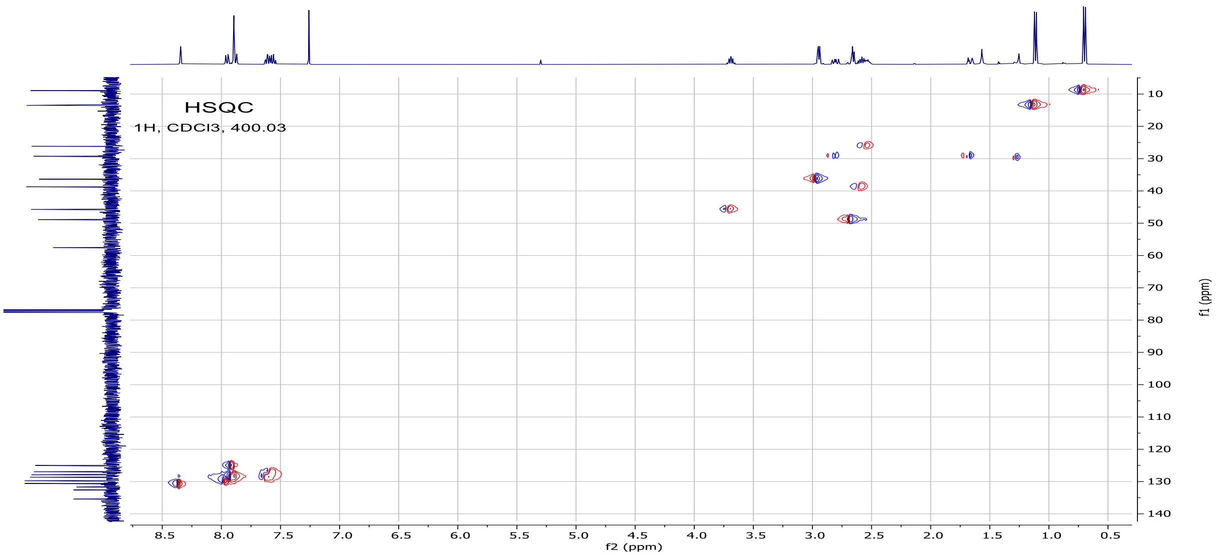

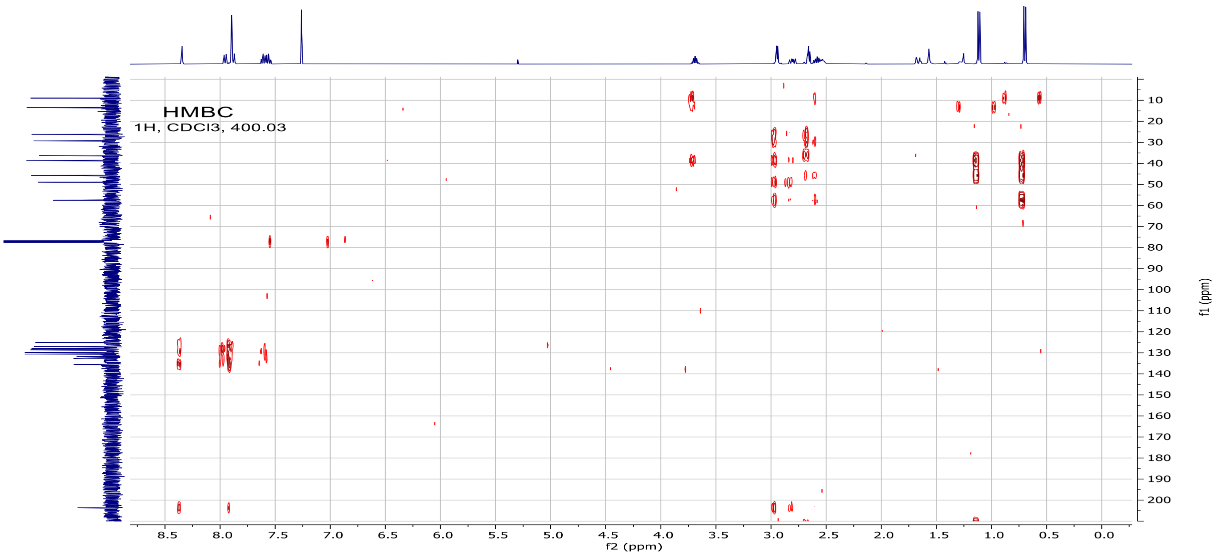

1-(3-(2-Naphthoyl)cyclobutyl)-3-methylpent-3-en-2-one (4ae') *(Impurities are highlighted in red boxes)*

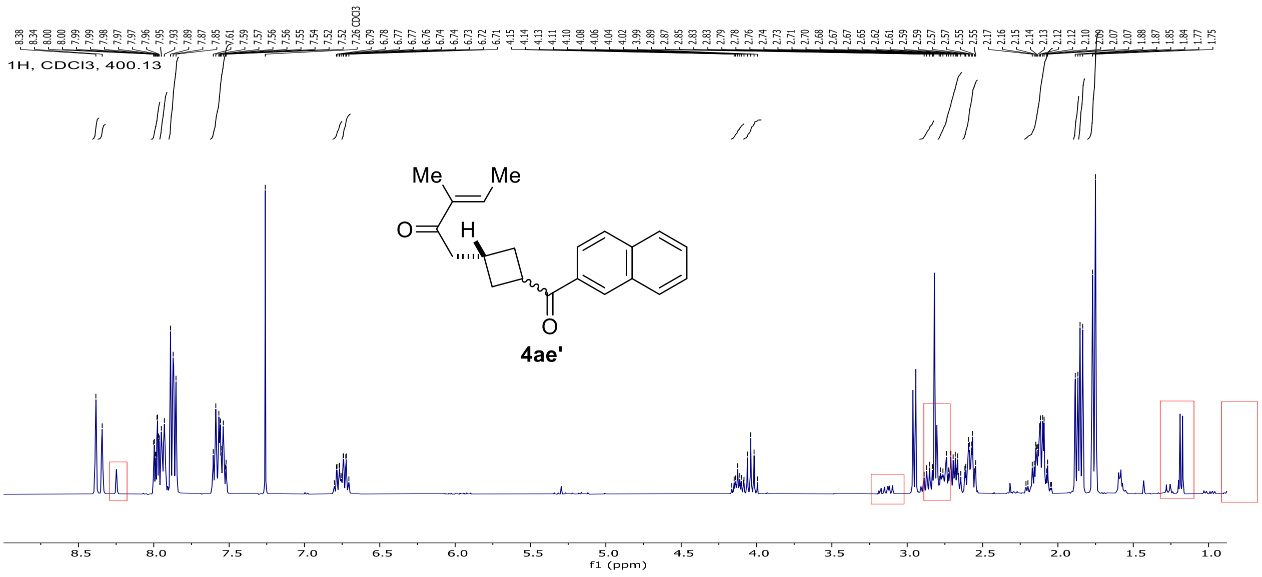

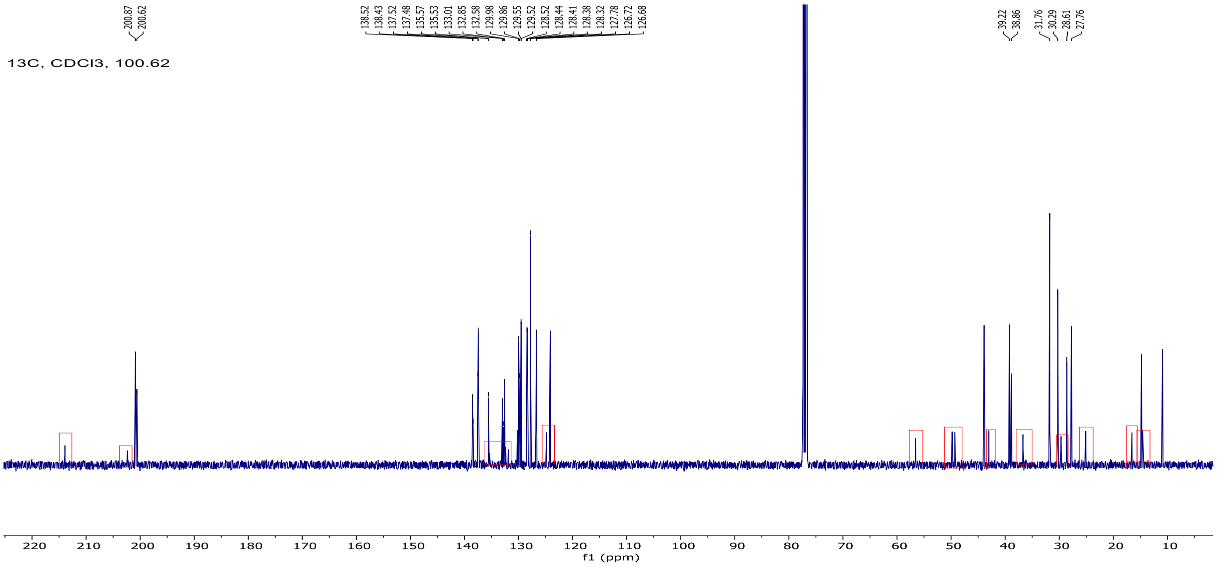

6-(2-Naphthoyl)-4-benzylbicyclo[4.1.1]octan-3-one (4ag)

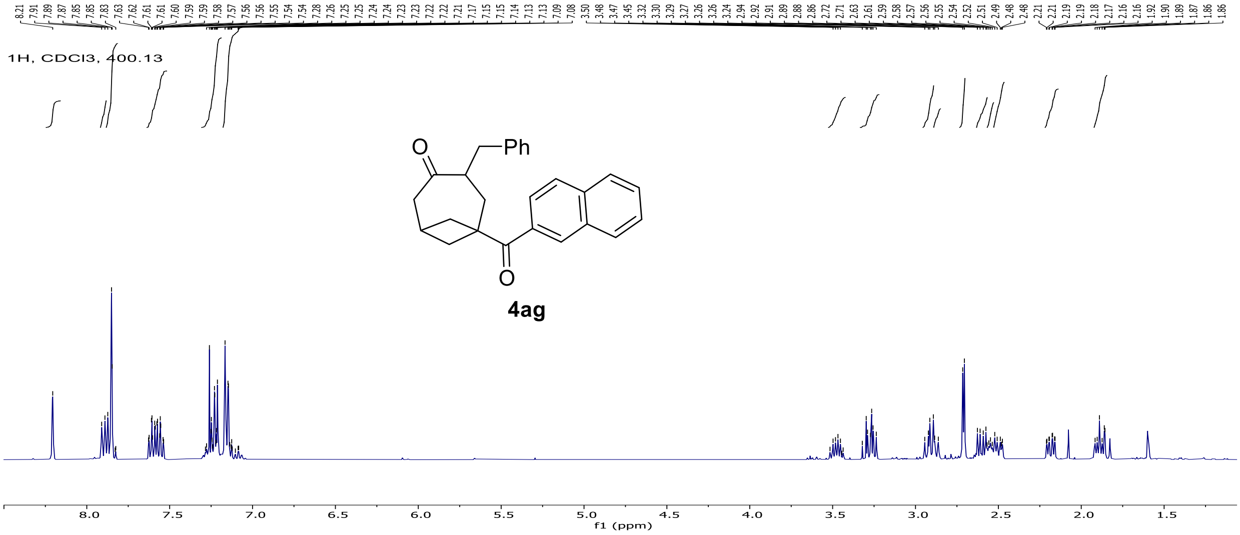

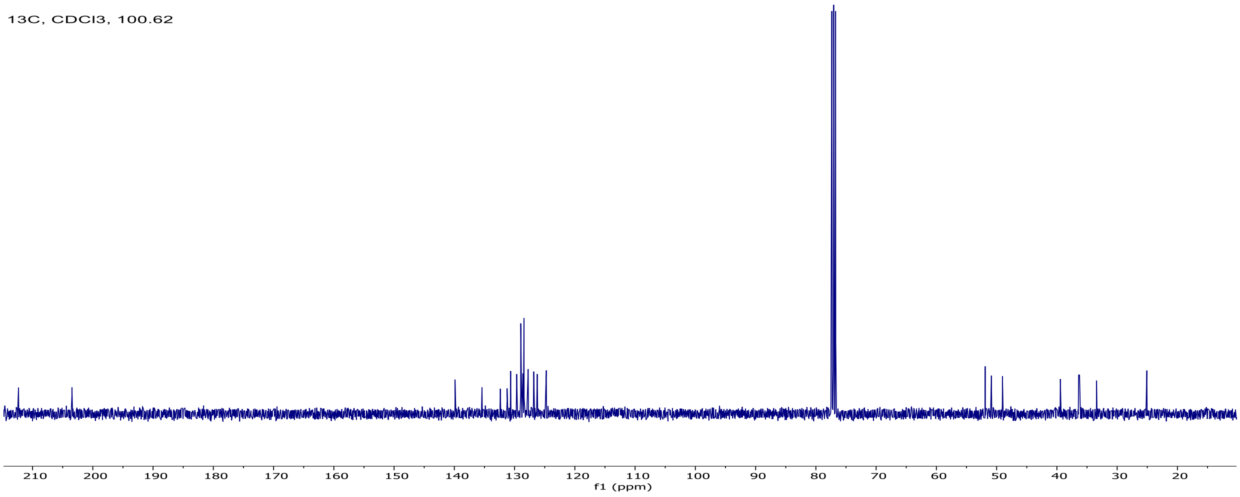

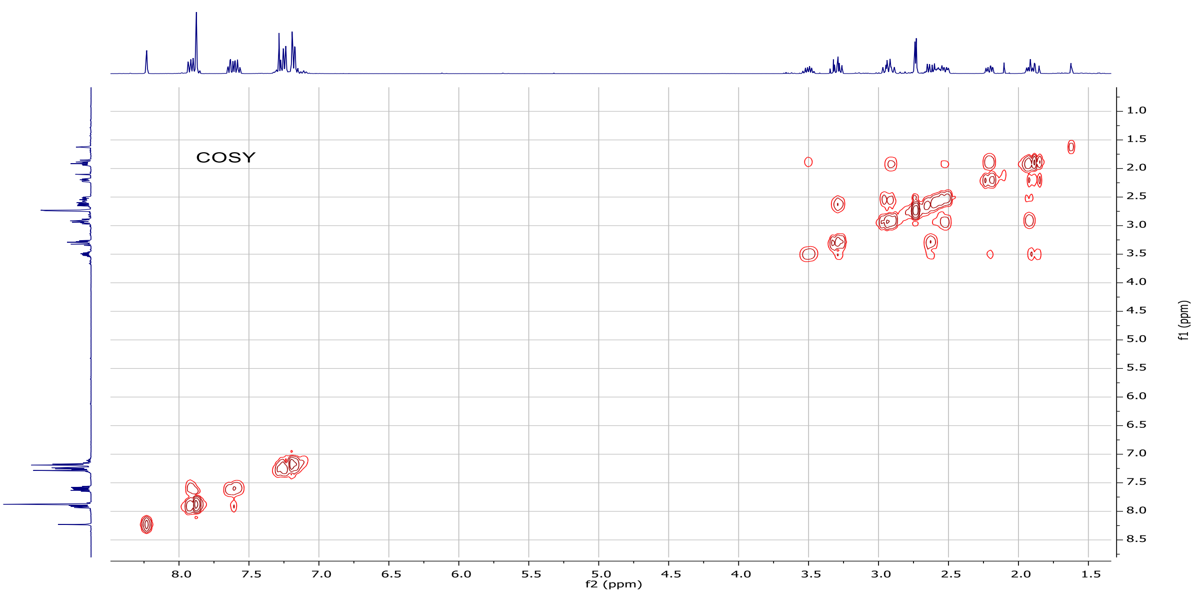

6-(2-Naphthoyl)-4-butylbicyclo[4.1.1]octan-3-one (4ah)

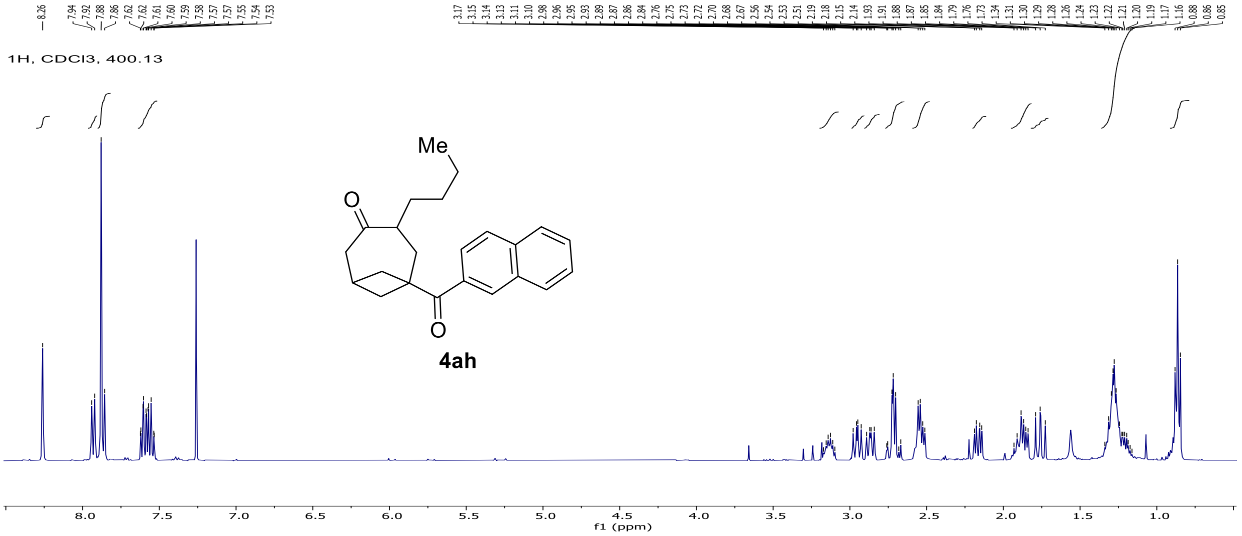

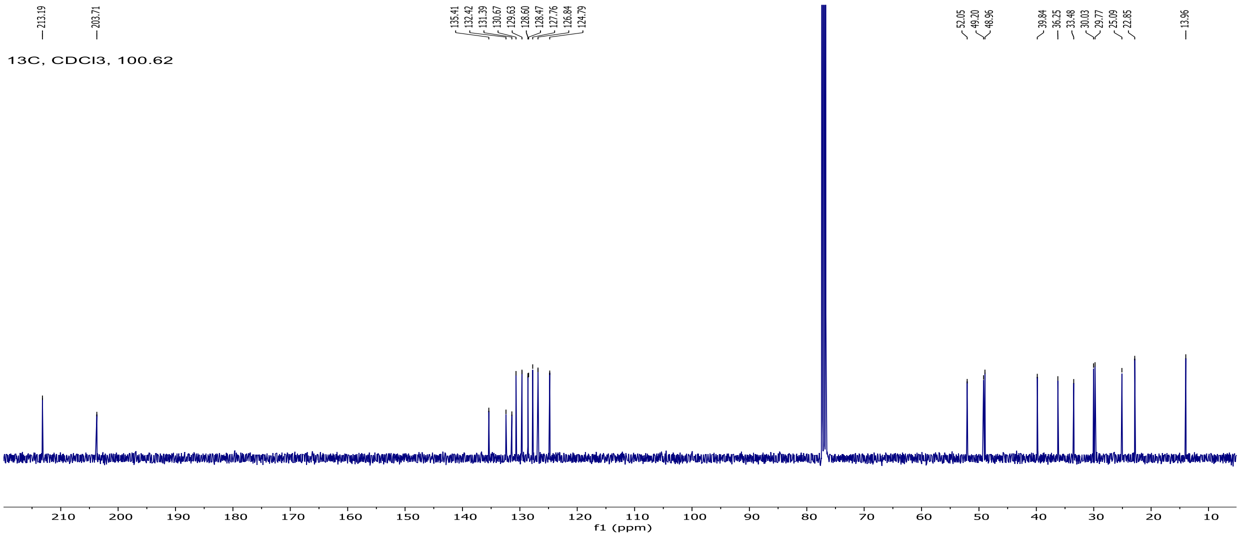

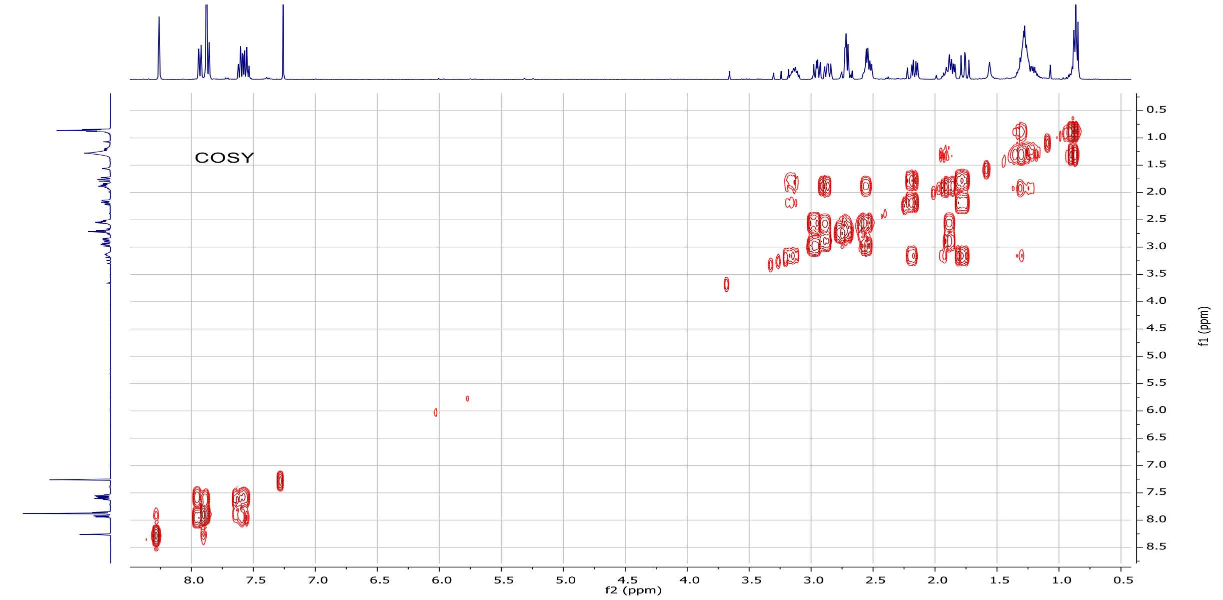

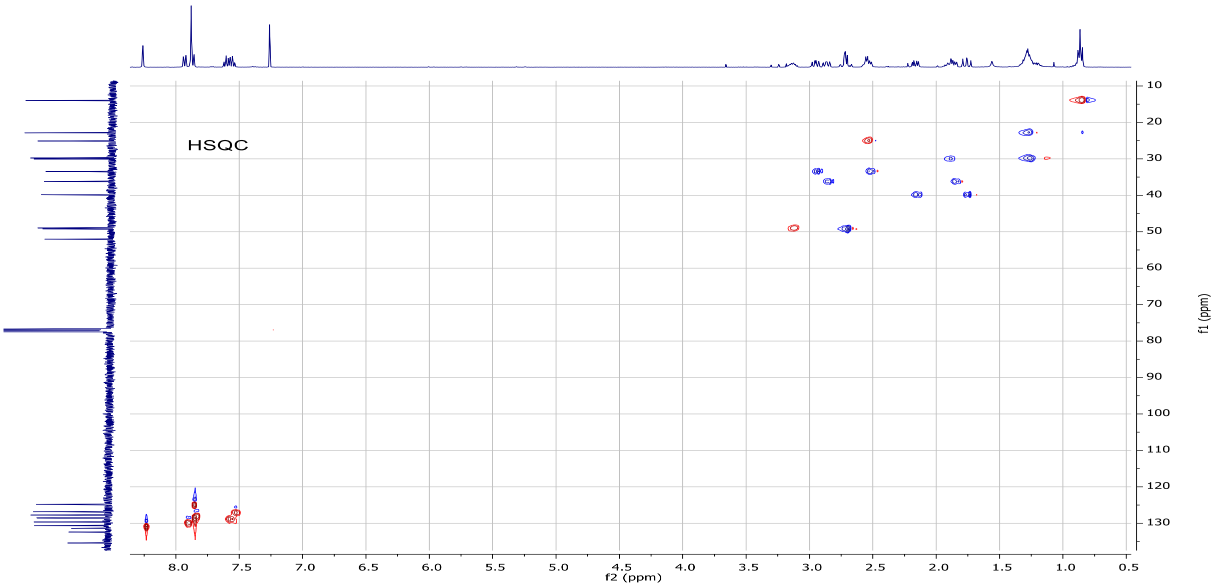

6-(2-Naphthoyl)-4-cyclohexylbicyclo[4.1.1]octan-3-one (4ai)

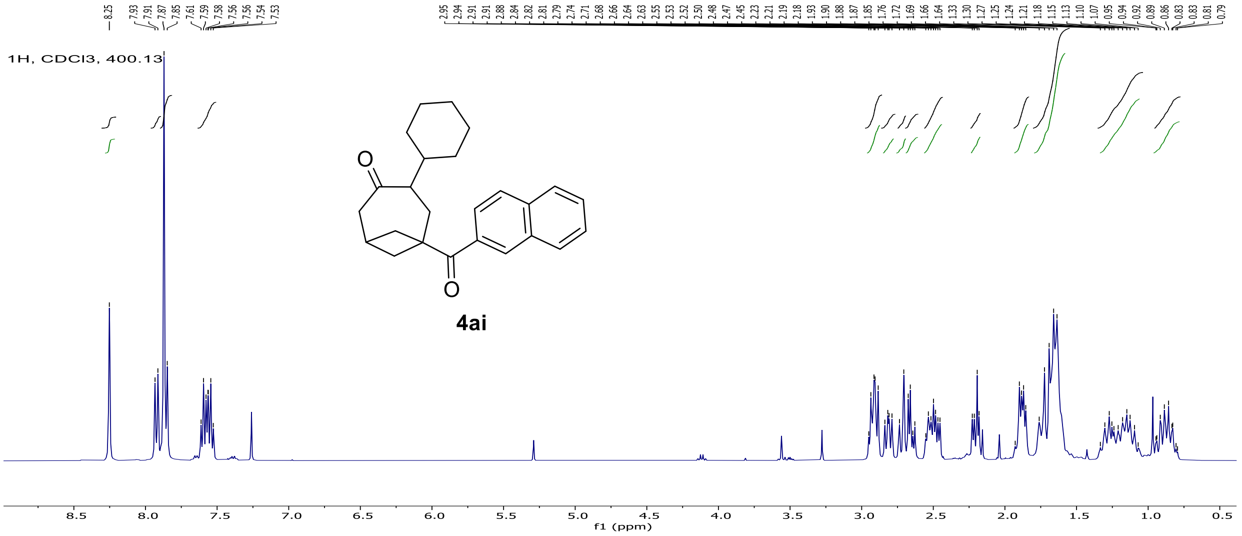

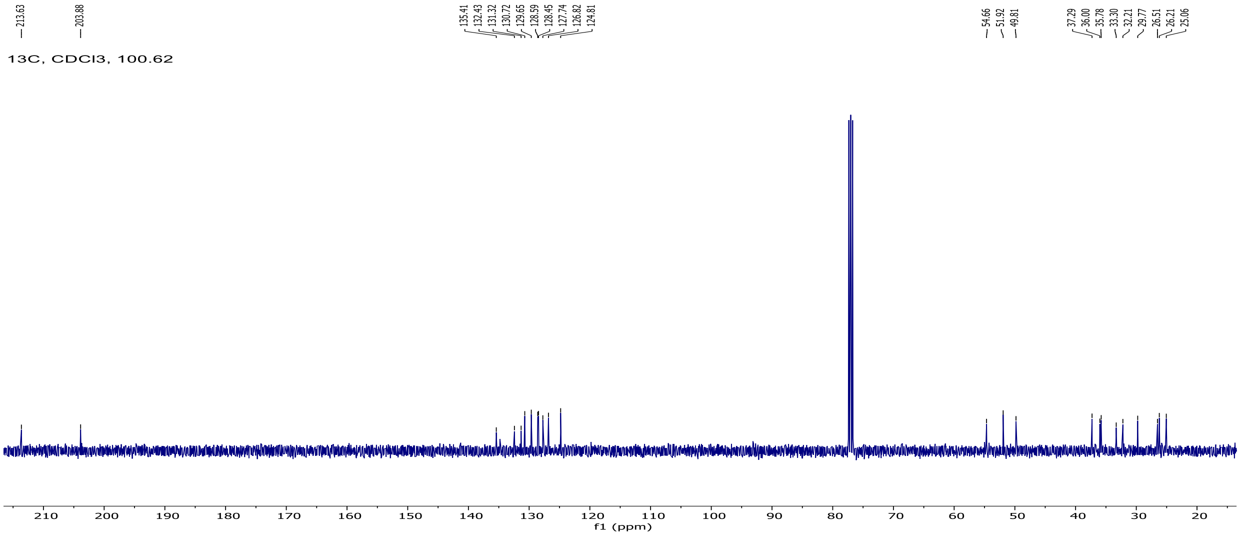

6-(2-Naphthoyl)-4-(4-methoxyphenyl)bicyclo[4.1.1]octan-3-one (4aj)

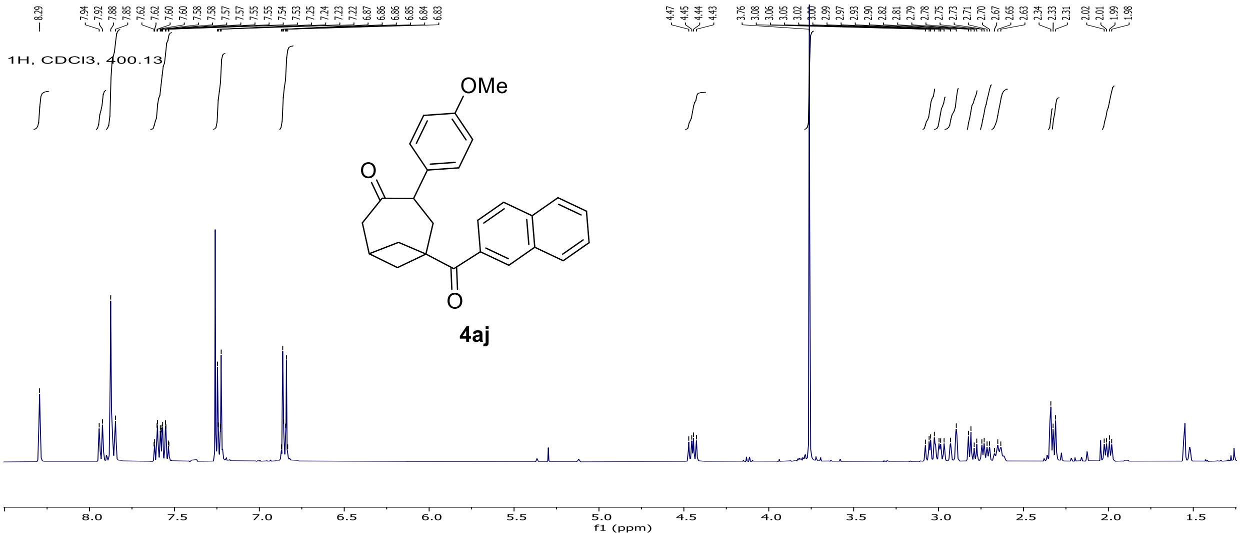

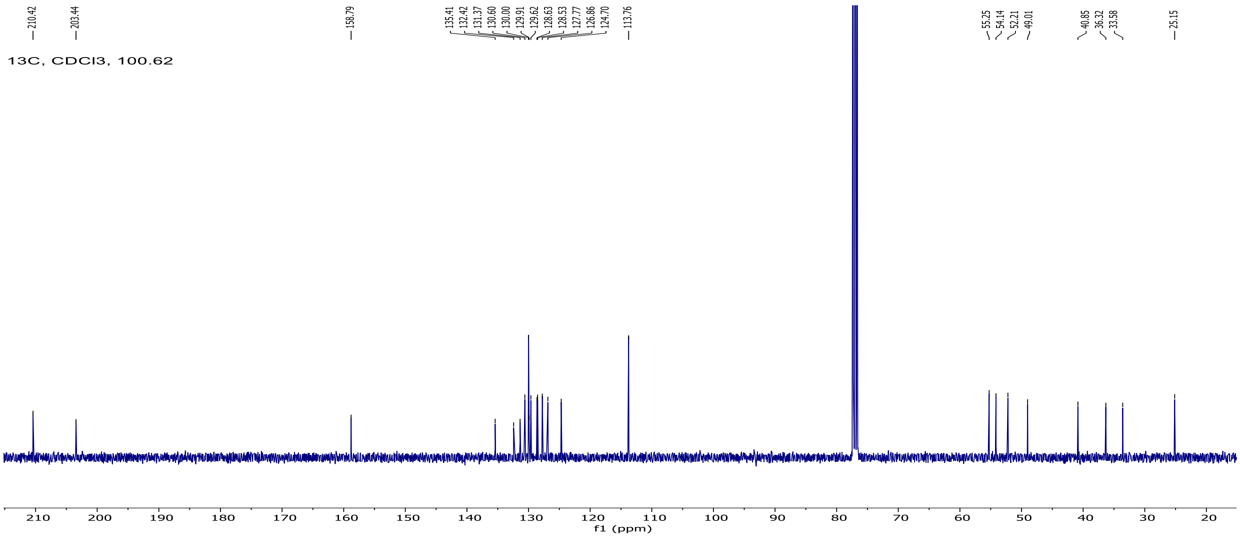

6-(2-Naphthoyl)-4-(benzofuran-5-yl)bicyclo[4.1.1]octan-3-one (4ak)

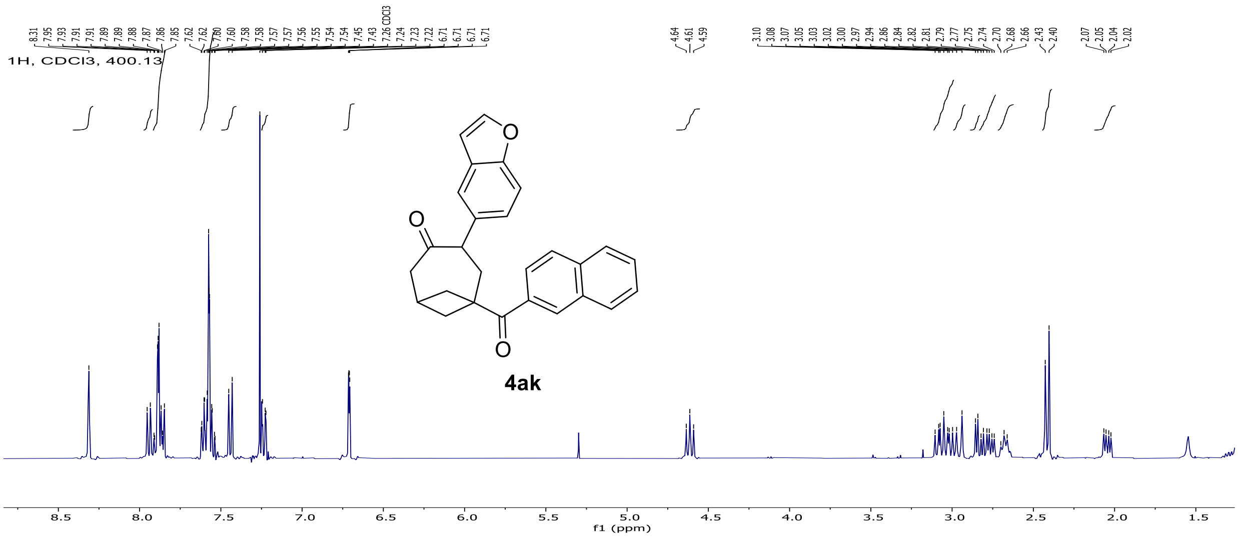

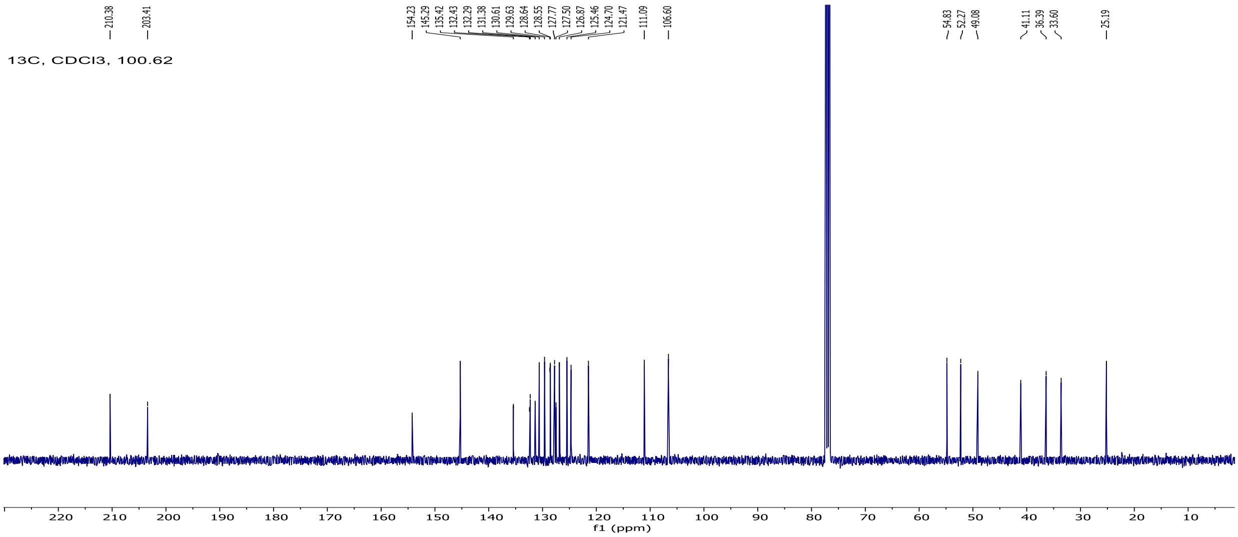

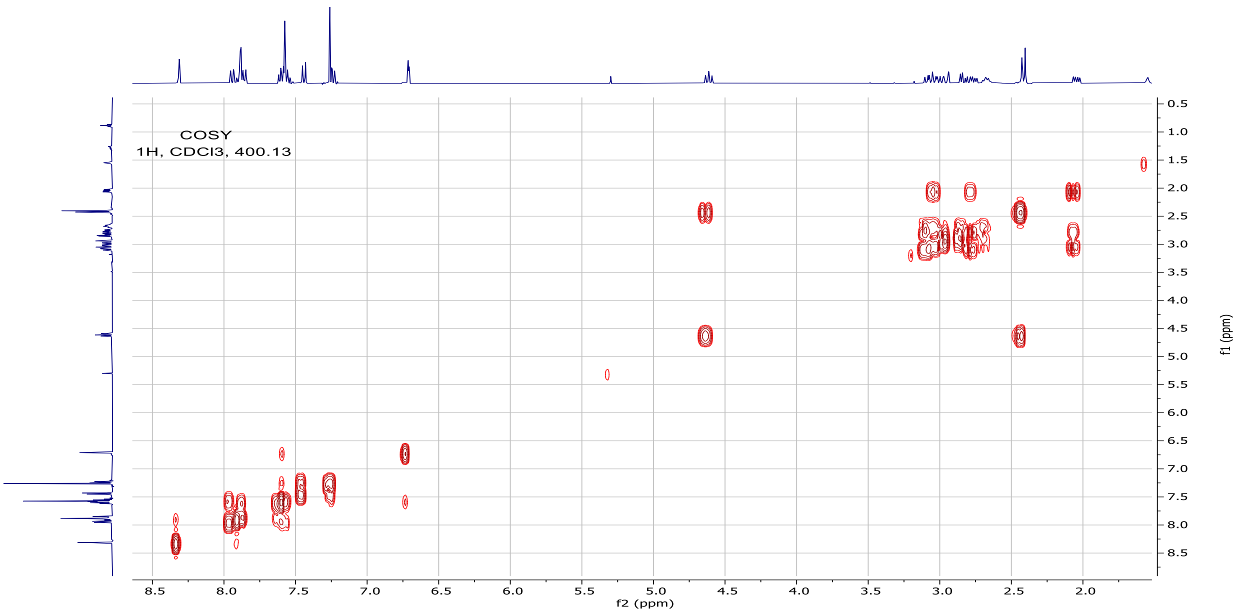

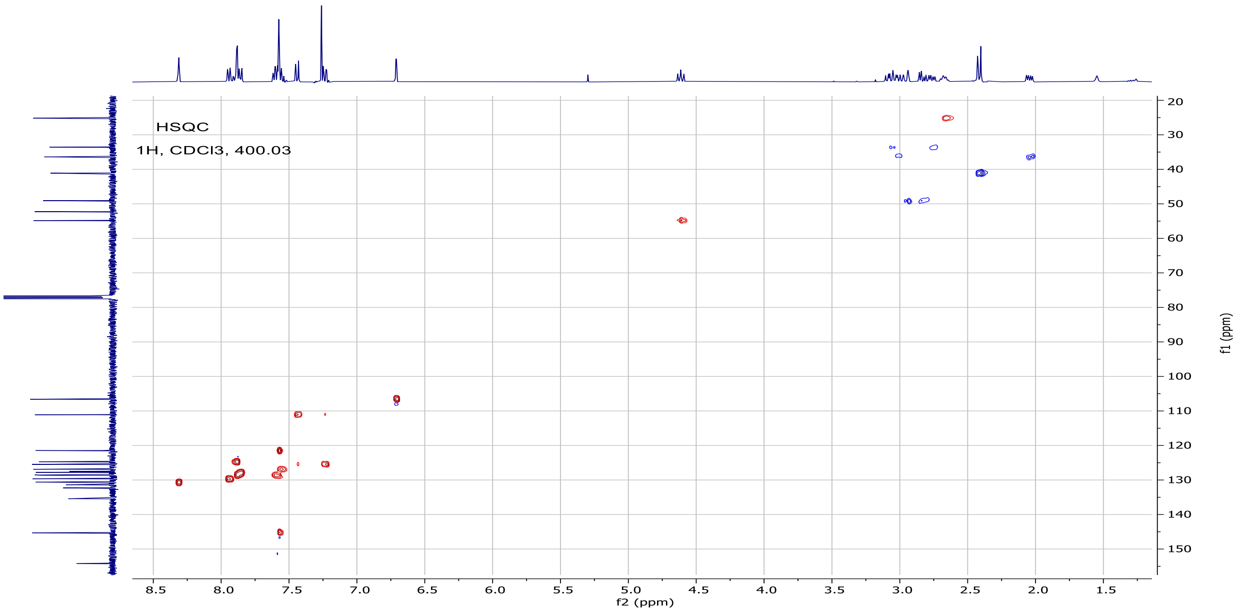

6-(2-Naphthoyl)-4-(4-chlorophenyl)bicyclo[4.1.1]octan-3-one (4al)

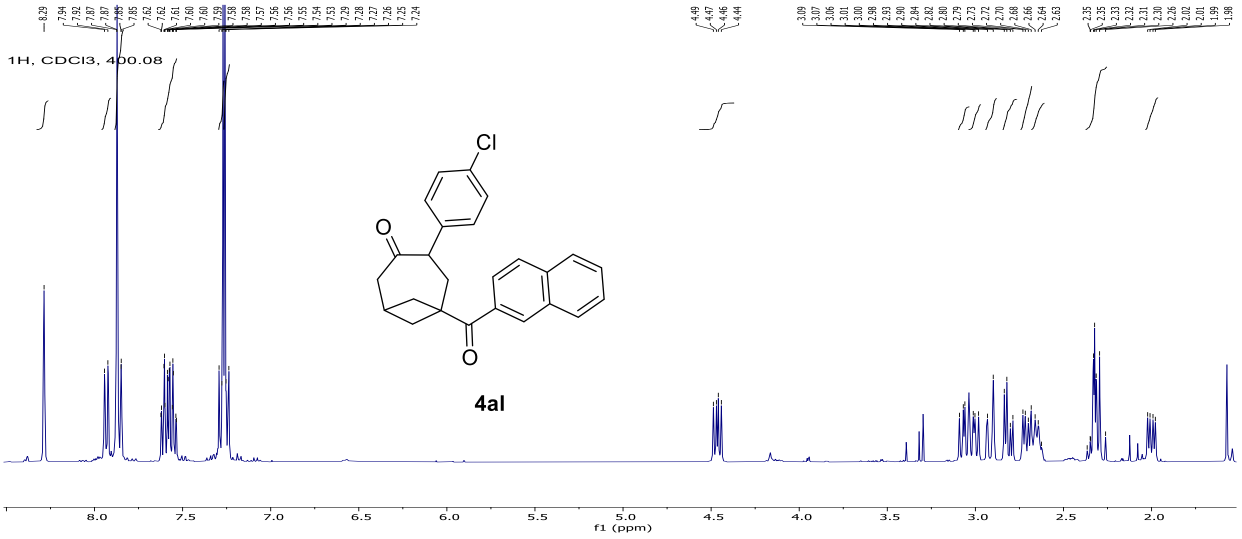

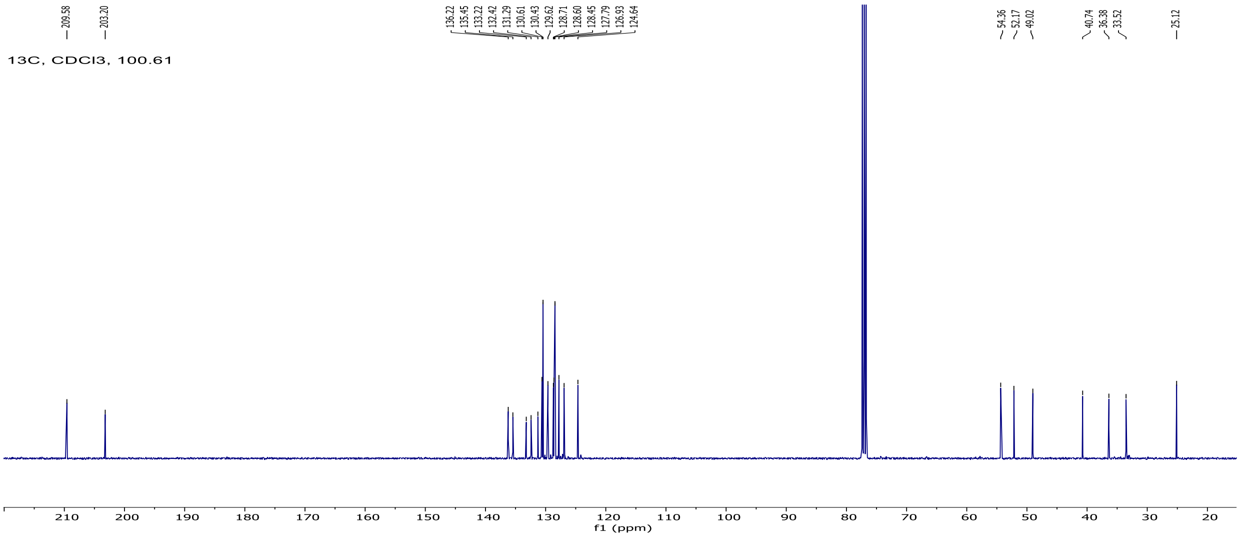

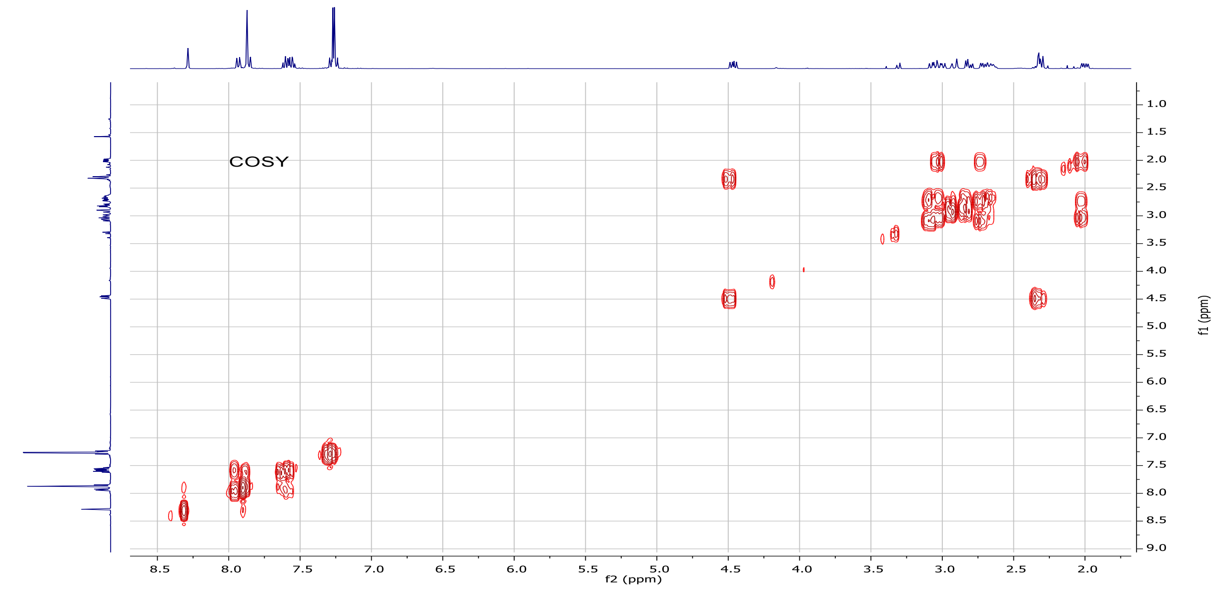

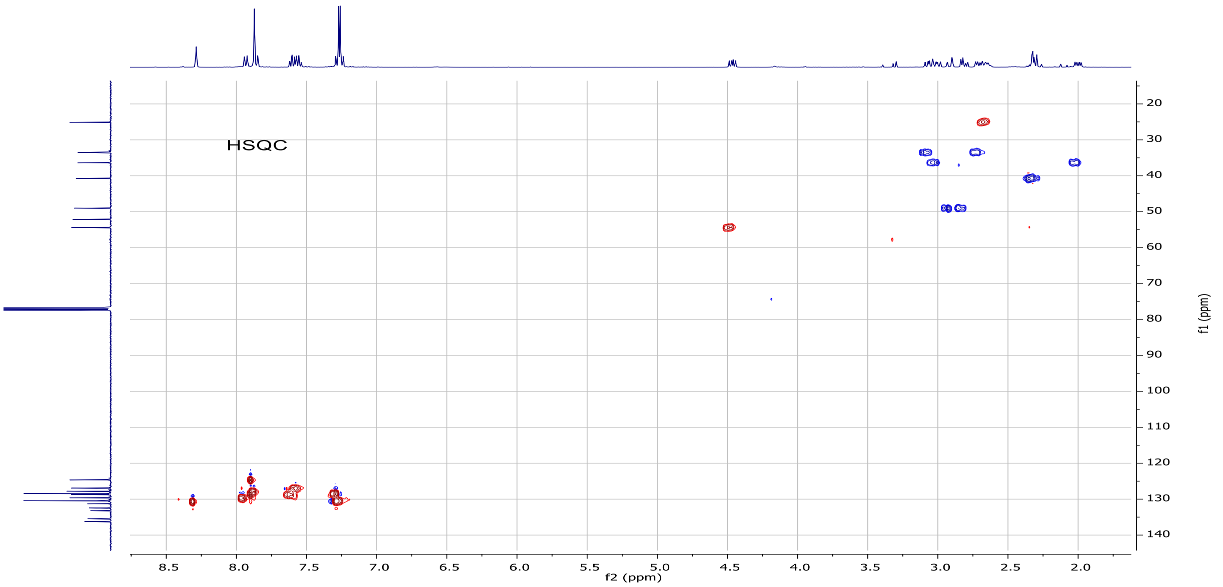

6-(2-Naphthoyl)-4-(naphthalen-1-yl)bicyclo[4.1.1]octan-3-one (4am)

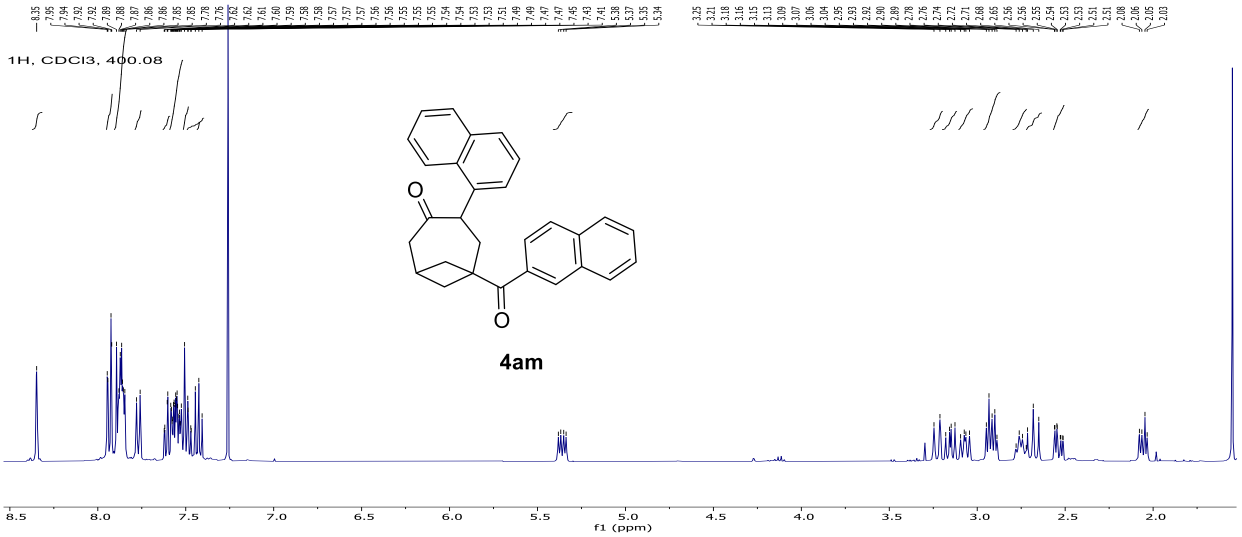

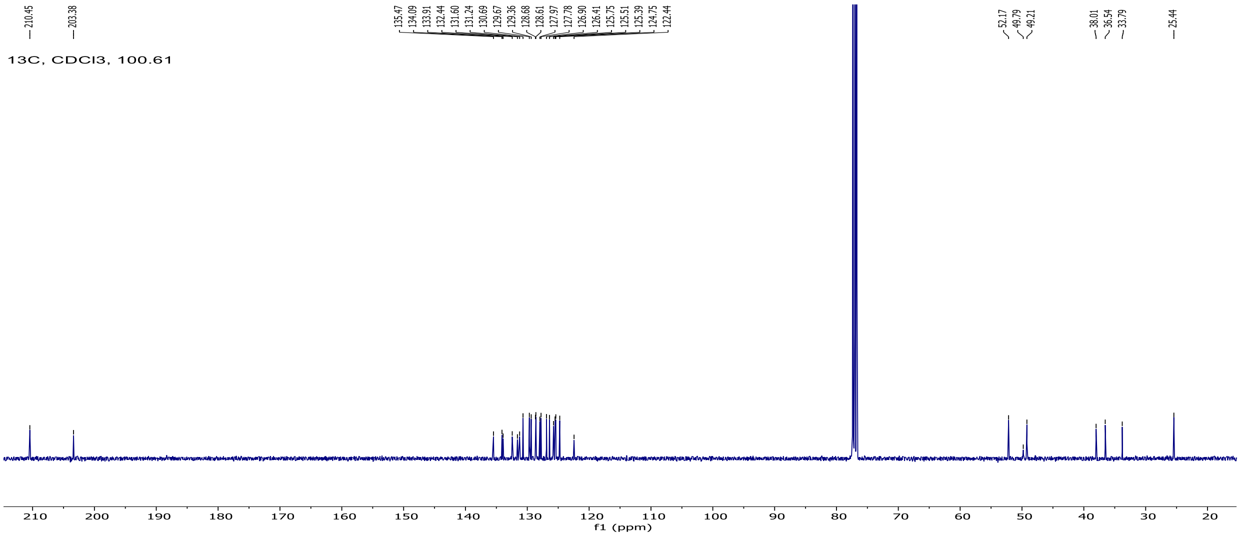
13C, CDCl3, 100.61
210.45
203.38
135.47
134.09
133.91
132.44
131.60
131.24
130.69
129.67
129.36
128.68
128.61
127.97
127.78
126.90
126.41
125.75
125.51
125.39
124.75
122.44
52.17
49.79
49.21
38.01
36.54
33.79
25.44
f1 (ppm)

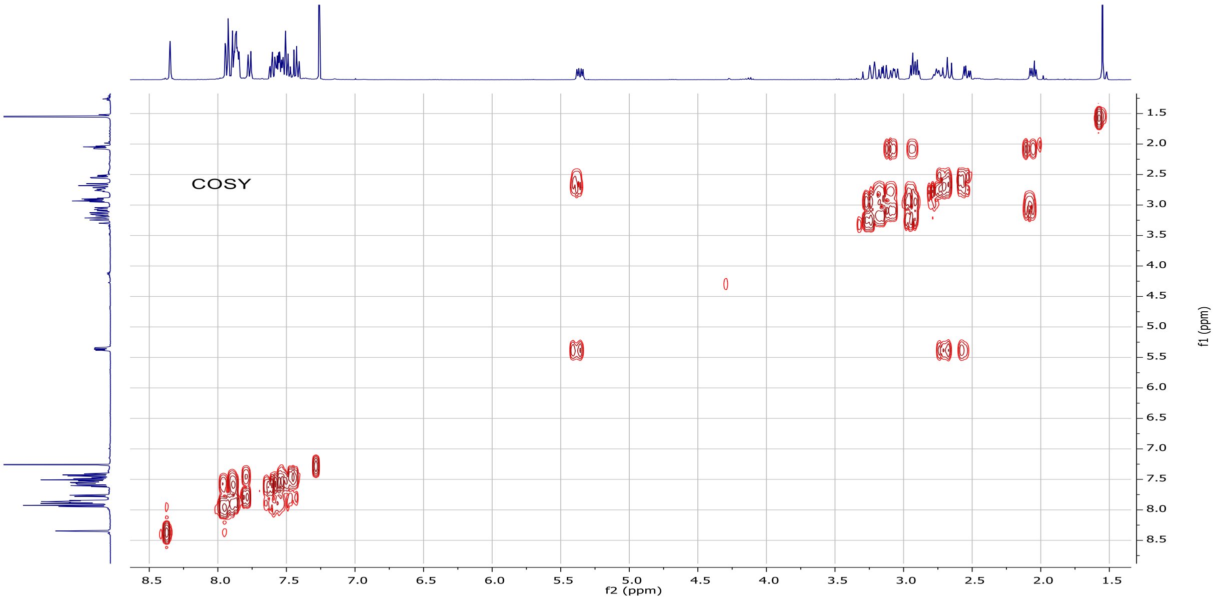
COSY
f2 (ppm)
f1 (ppm)

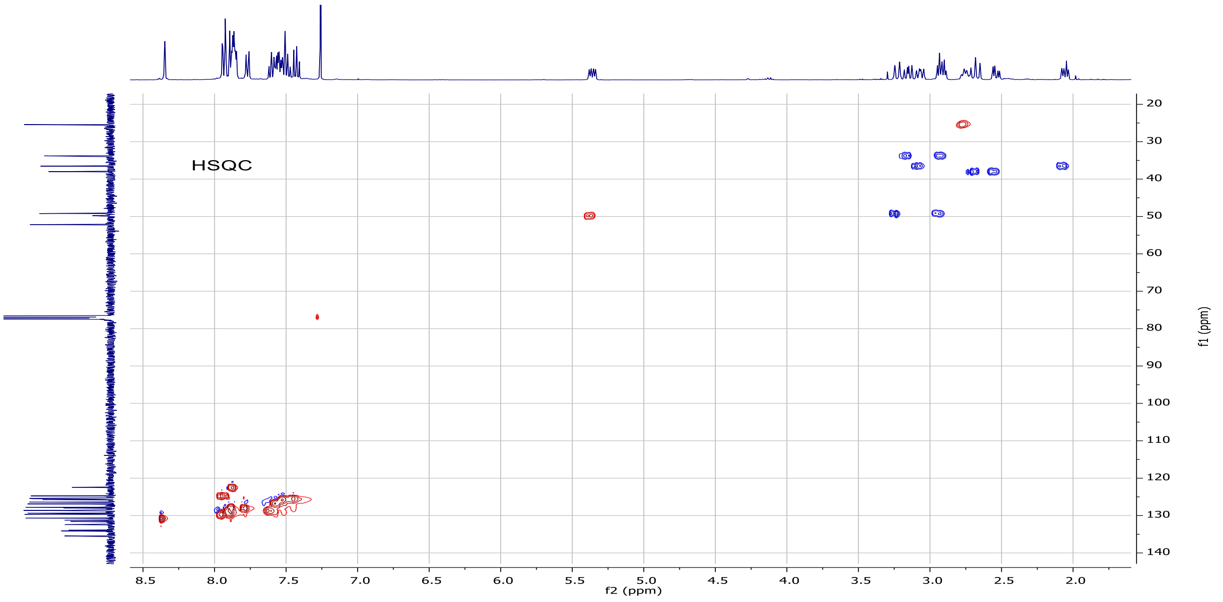
HSQC
f2 (ppm)
f1 (ppm)

4-((tert-Butyldiphenylsilyl)oxy)-N-methoxy-N,3-dimethylbicyclo[4.1.1]oct-3-ene-1-carboxamide (4qa)

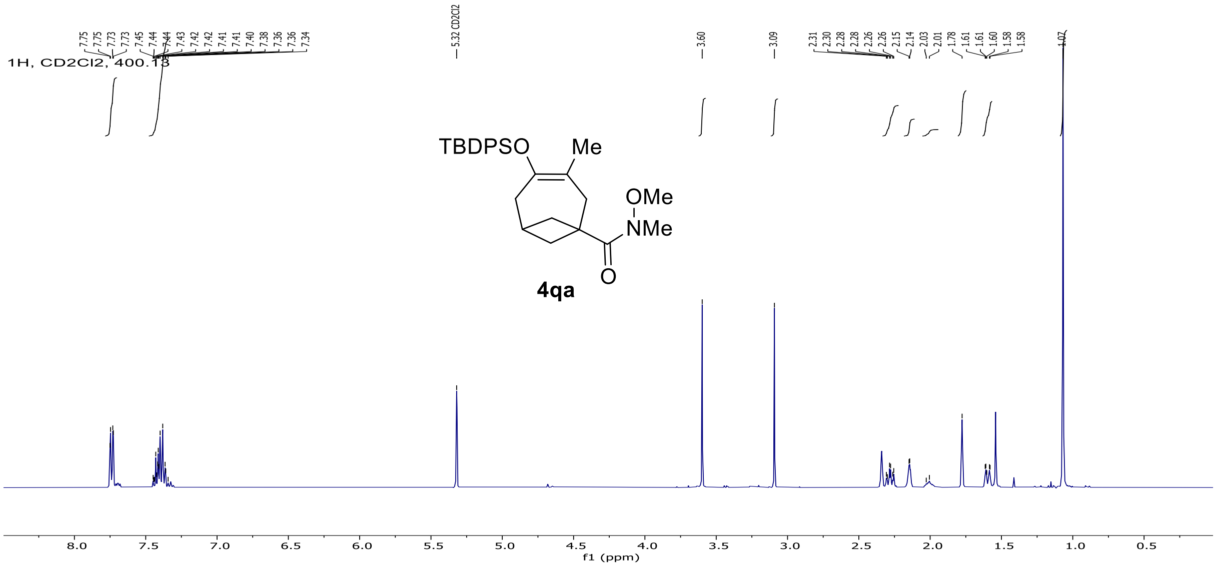

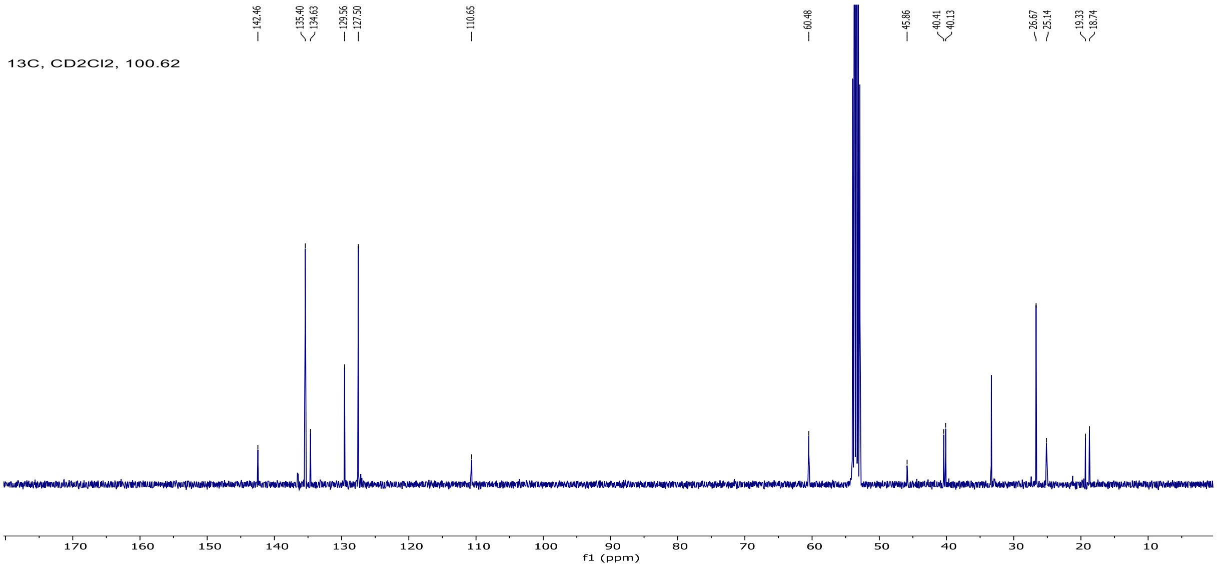

6-(2-Naphthoyl)-4-methylbicyclo[4.1.1]oct-2-en-3-yl trifluoromethanesulfonate (5)

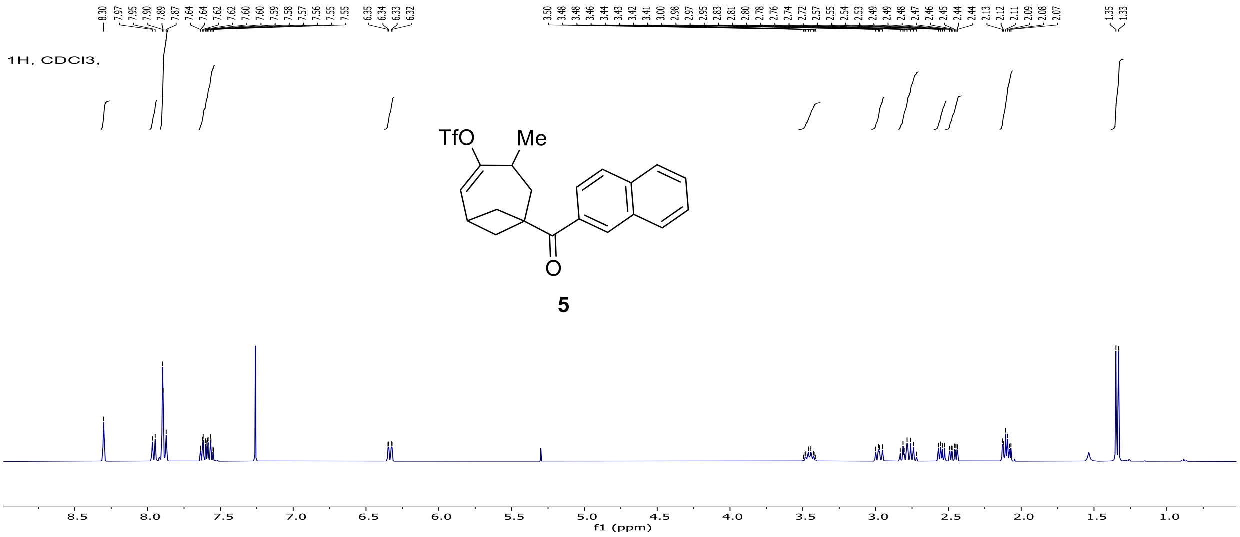

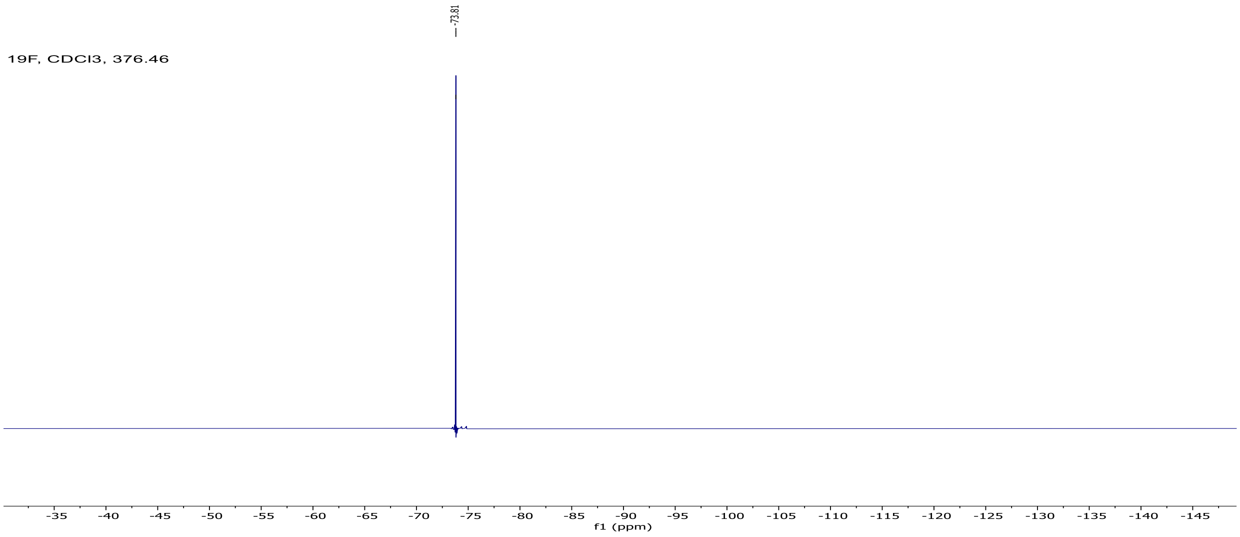
19F, CDCl3, 376.46
-73.81
f1 (ppm)

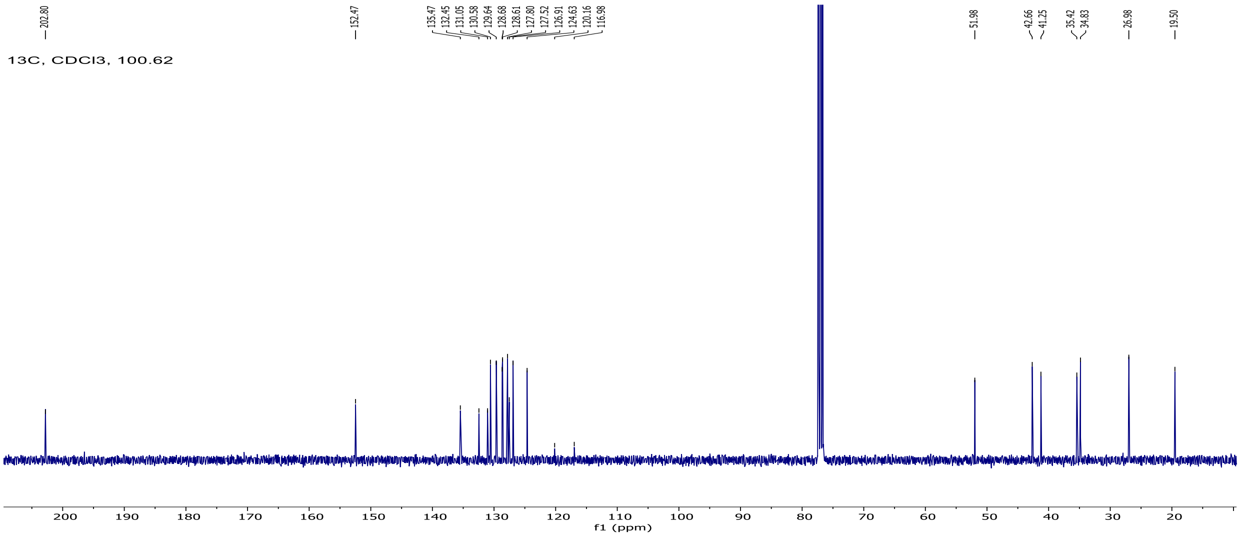
13C, CDCl3, 100.62
202.80
152.47
135.47
132.45
131.05
130.58
129.64
128.68
128.61
127.80
127.52
126.91
124.63
120.16
116.98
51.98
42.66
41.25
35.42
34.83
26.98
19.50
f1 (ppm)

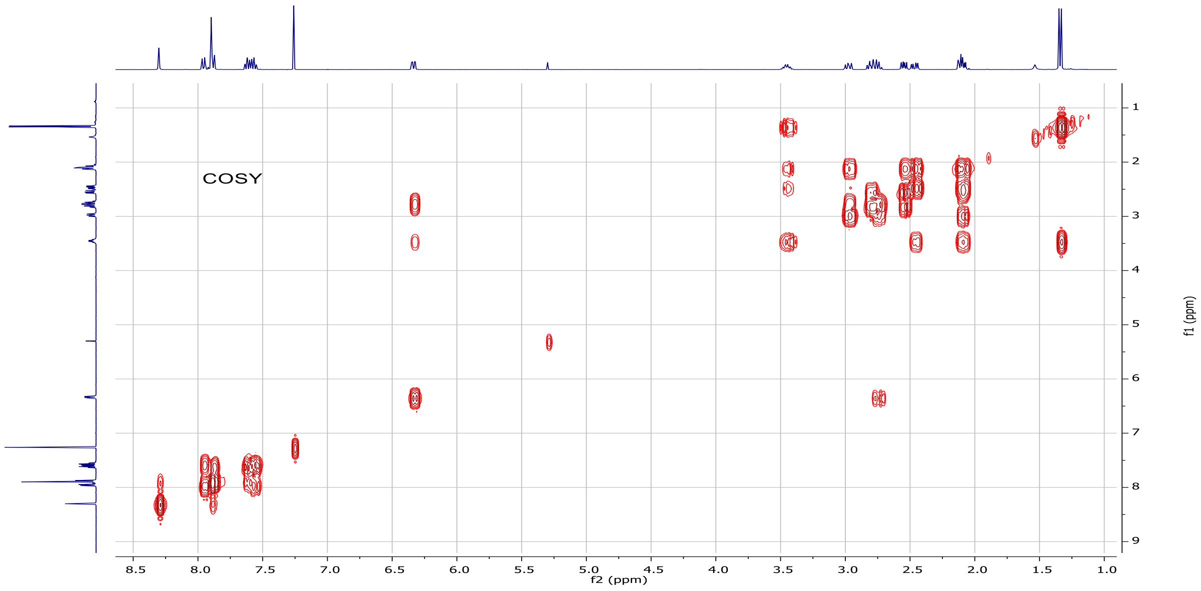
COSY
f2 (ppm)
f1 (ppm)

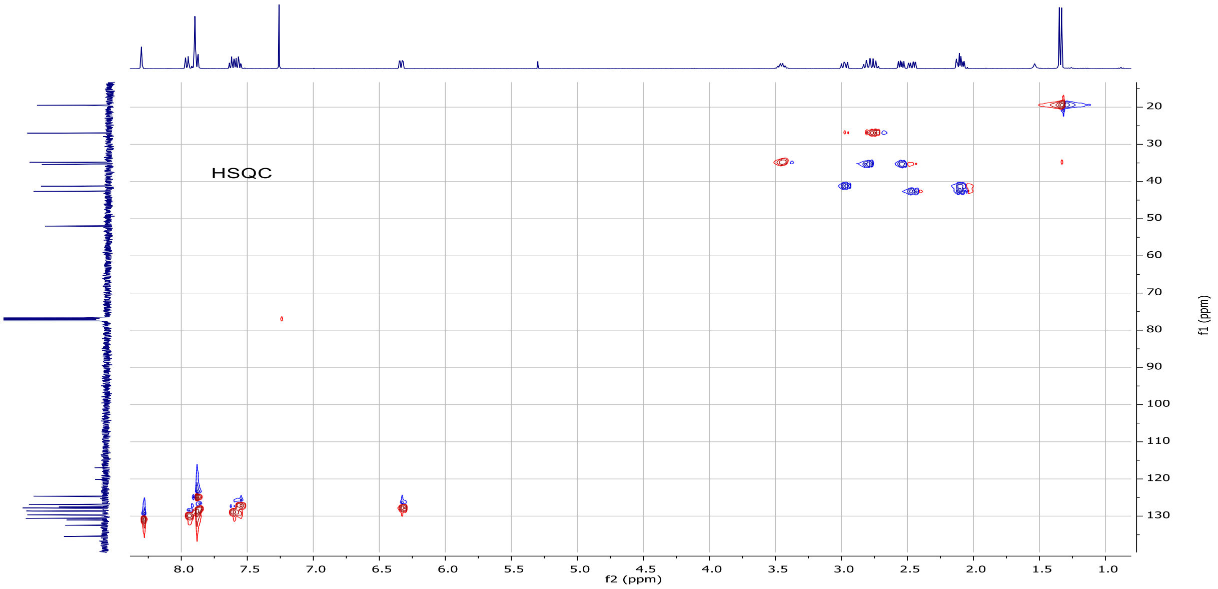

(3-Methyl-4-(p-tolyl)bicyclo[4.1.1]oct-4-en-1-yl)(naphthalen-2-yl)methanone (6)

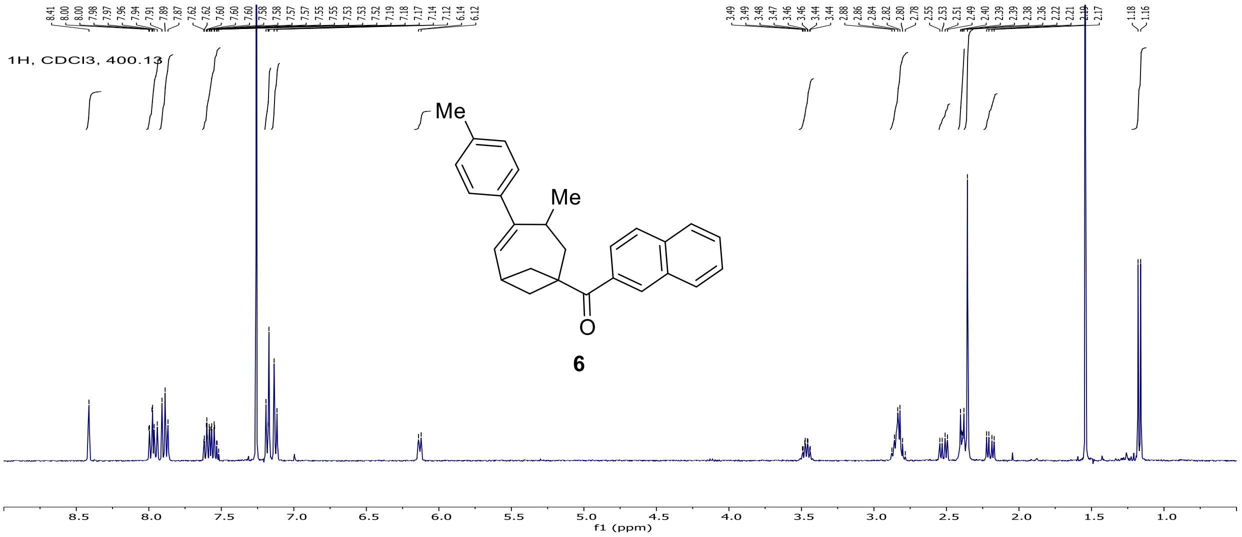

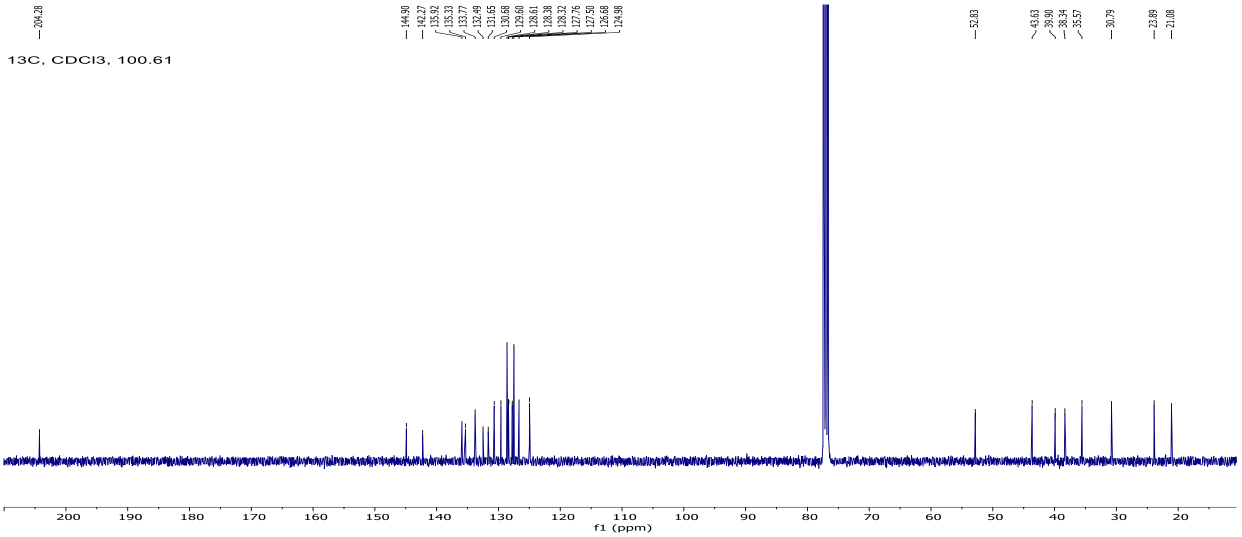

(3-Methylbicyclo[4.1.1]oct-4-en-1-yl)(naphthalen-2-yl)methanone (7)

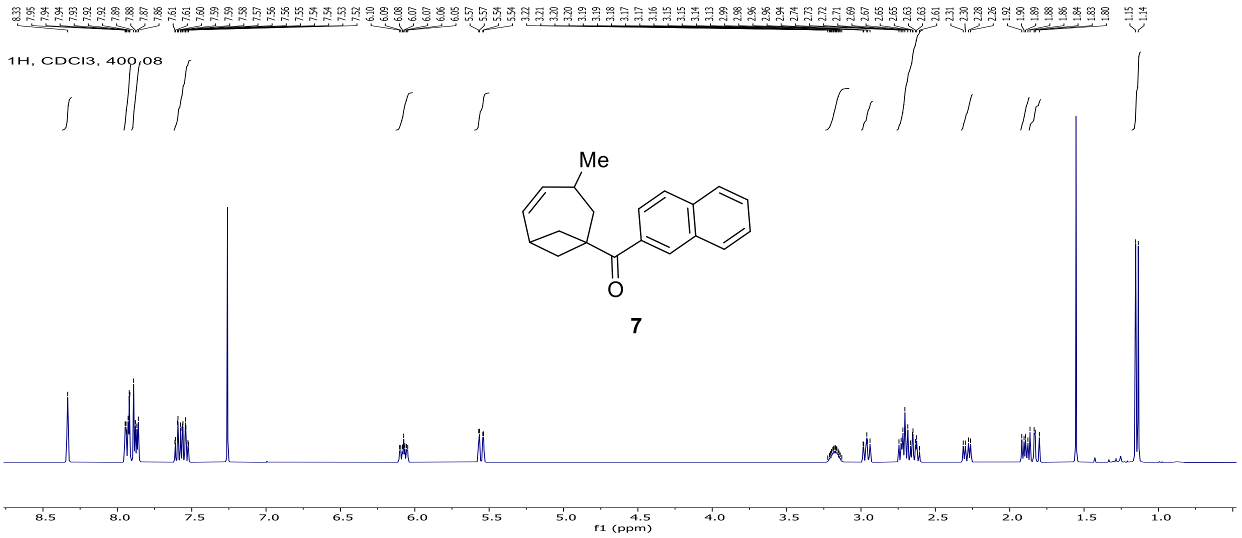

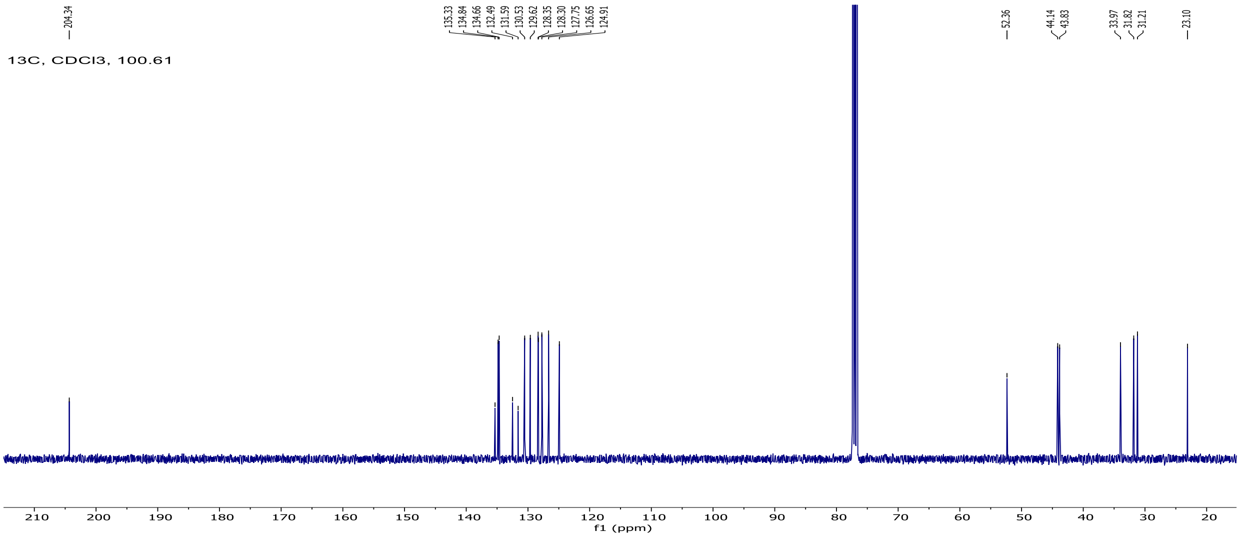

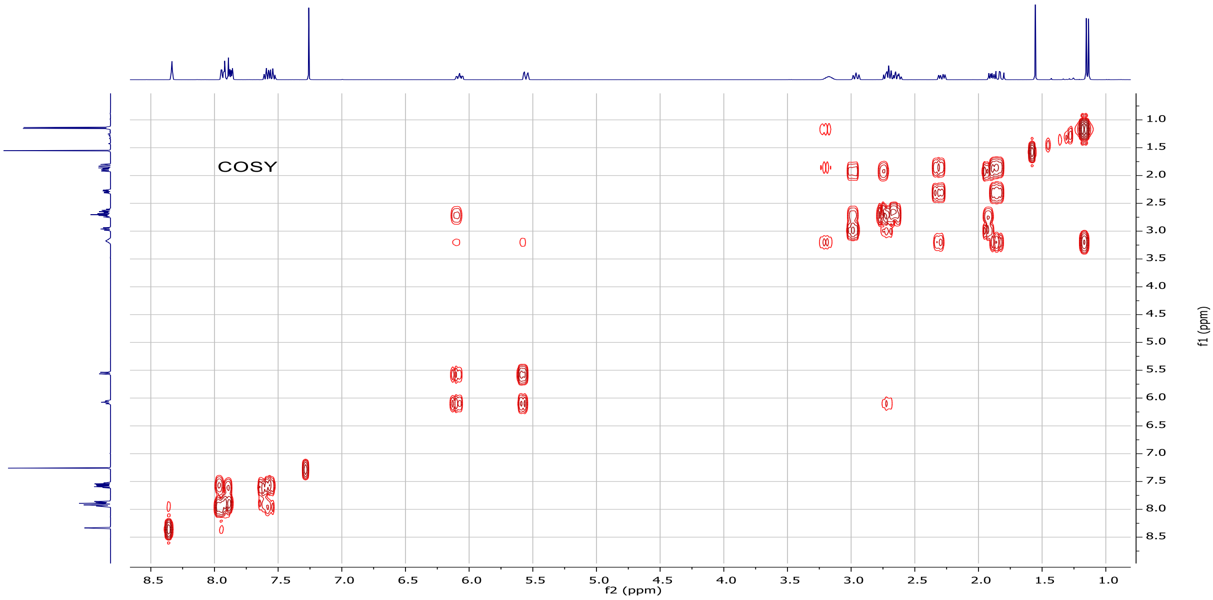

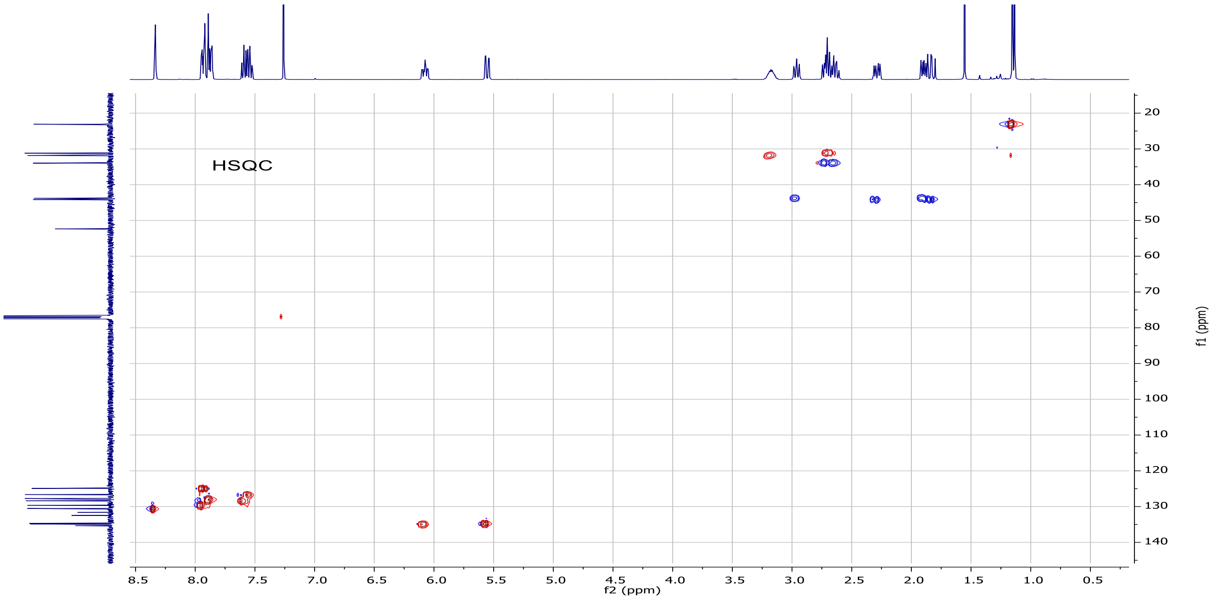

(3-Methyl-4-vinylbicyclo[4.1.1]oct-4-en-1-yl)(naphthalen-2-yl)methanone (8)

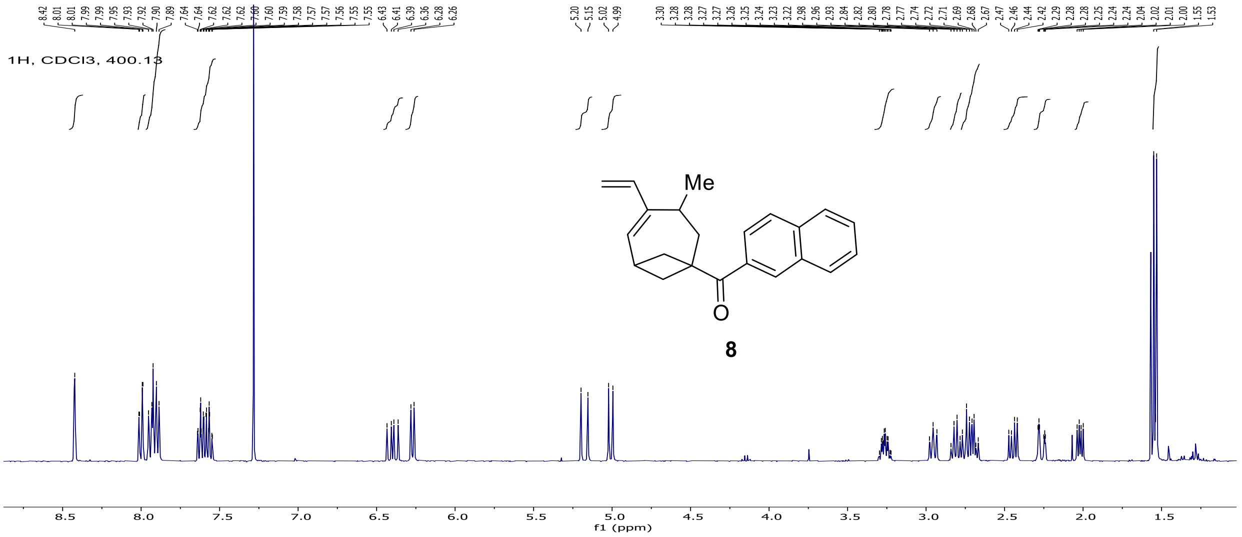

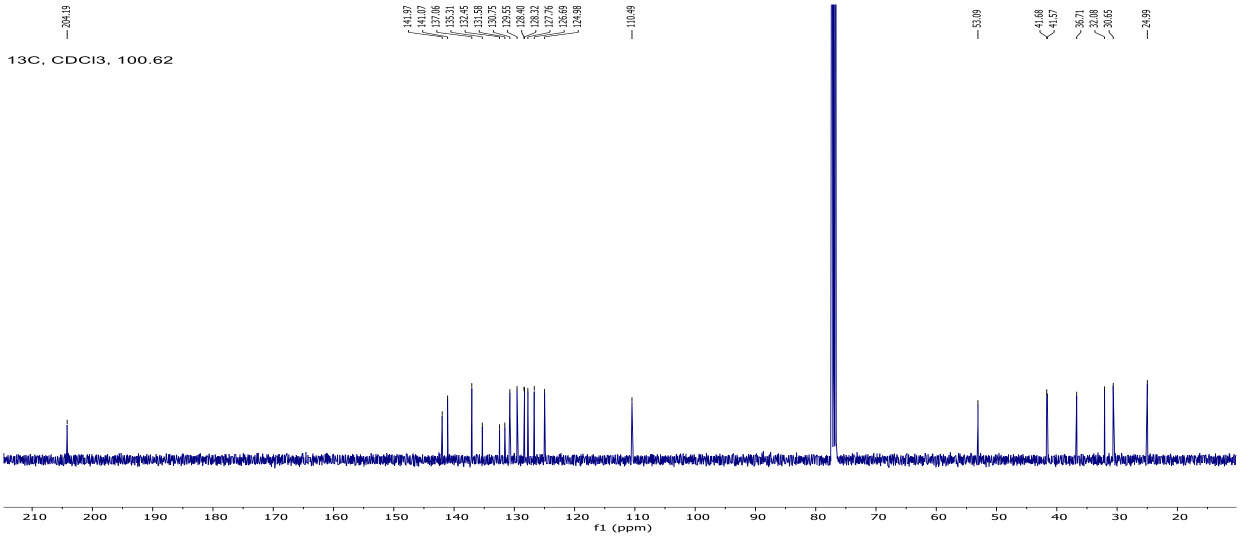

Dimethyl 7-(2-naphthoyl)-9-methyl-6,7,8,9-tetrahydro-5H-5,7-methanobenzo[7]annulene-2,3-dicarboxylate (9)

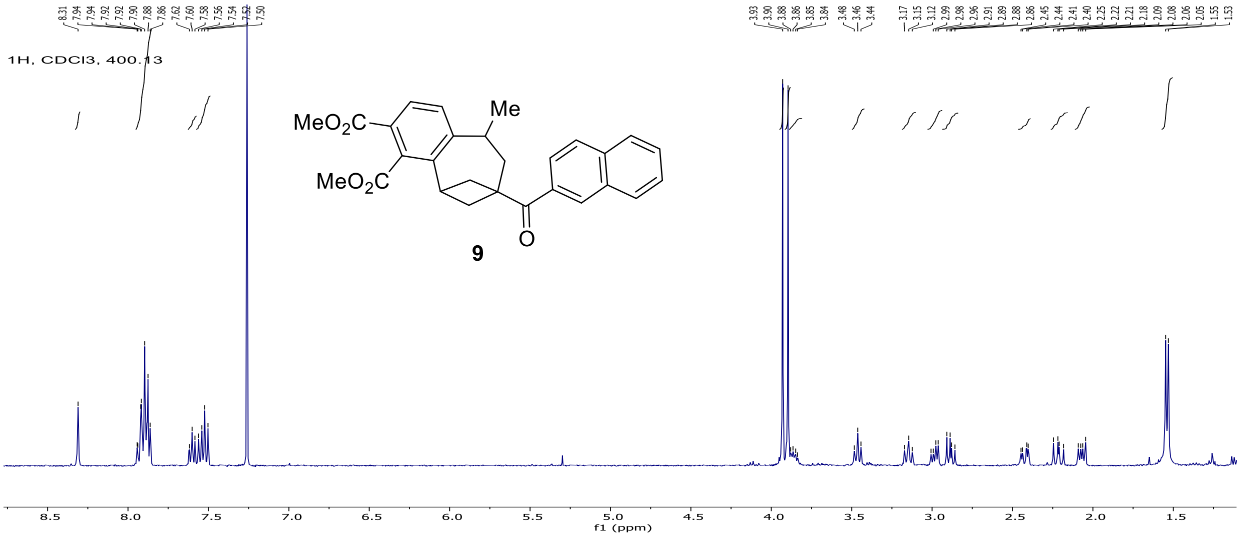

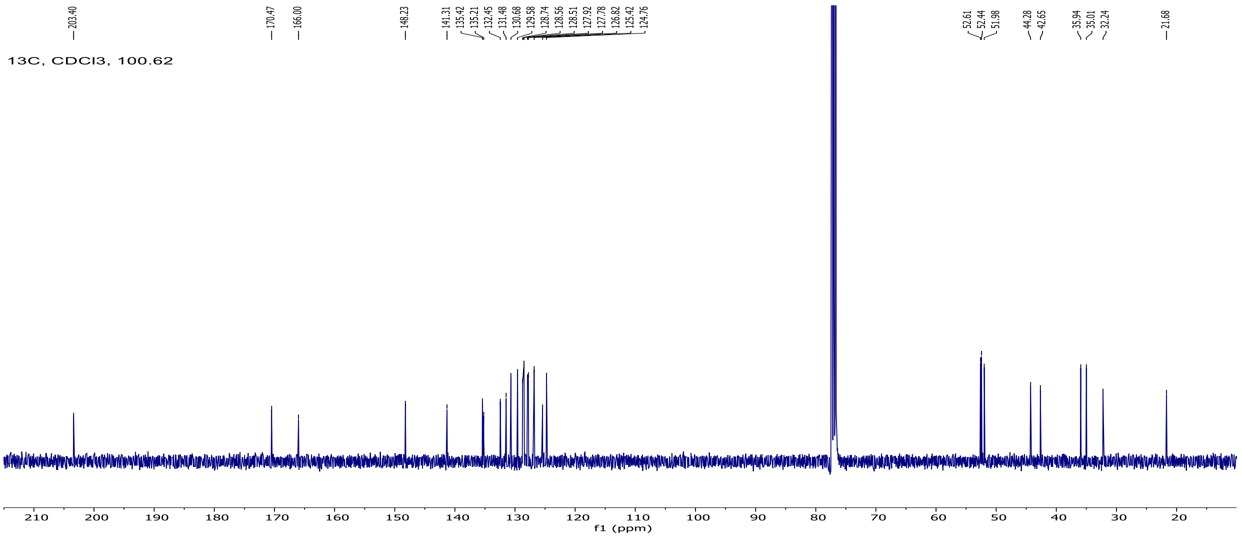

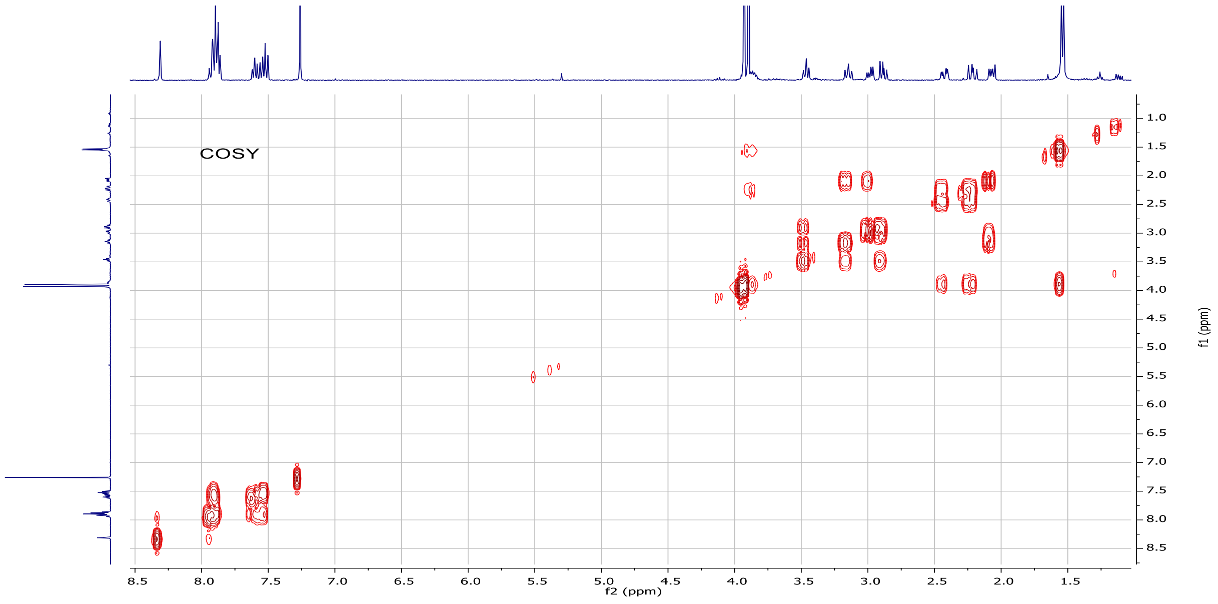

(3-Methylbicyclo[4.1.1]octan-1-yl)(naphthalen-2-yl)methanone (10)

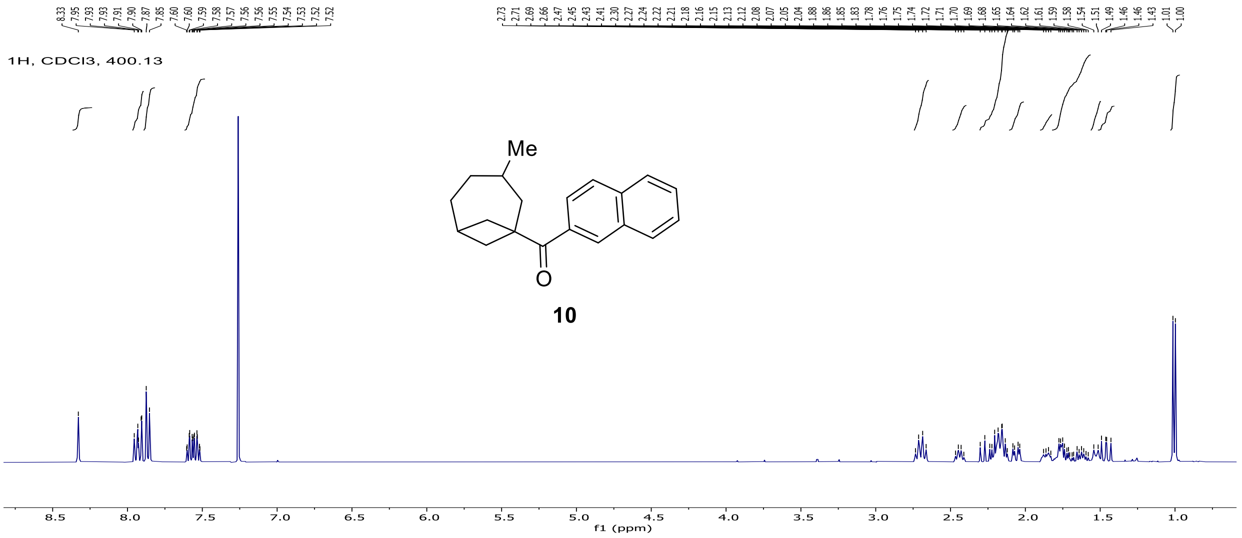

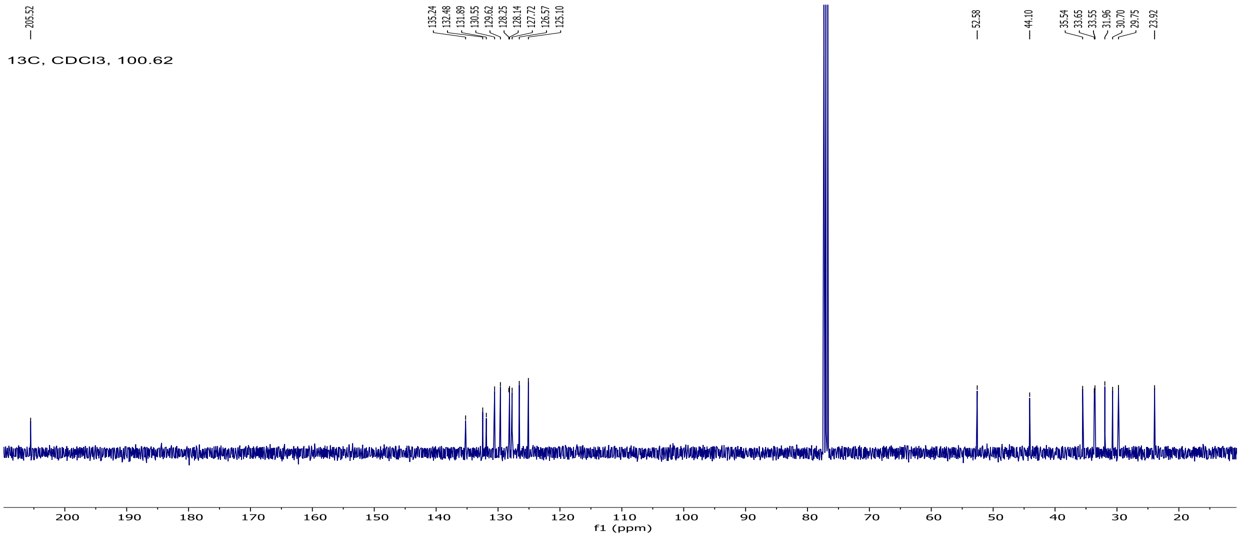

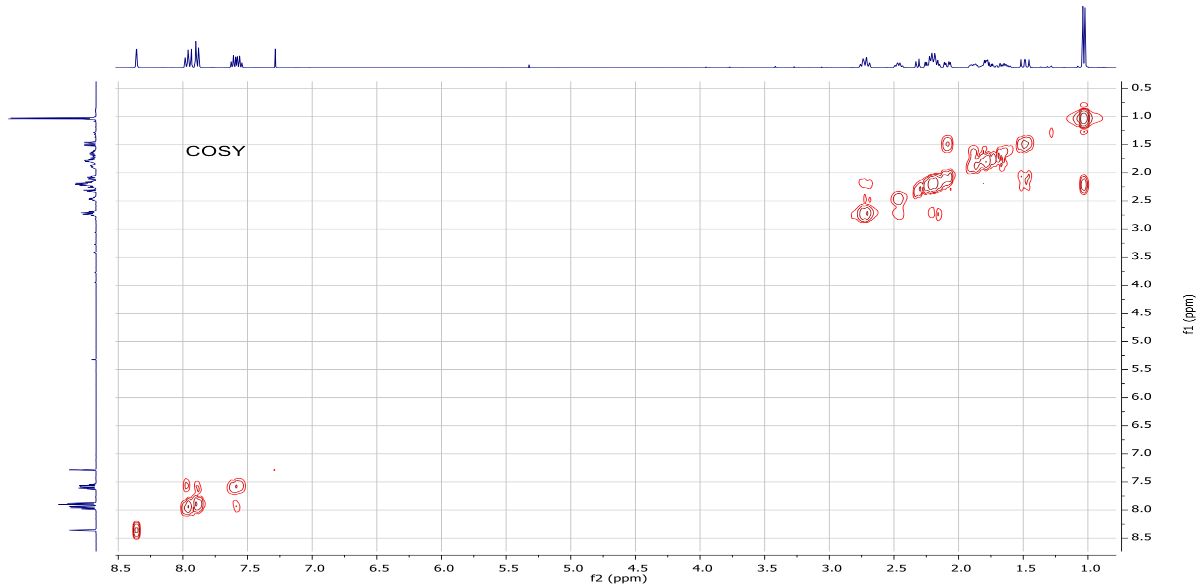

(10a-Methyl-7,8,10,10a-tetrahydro-7,9-methanocyclohepta[b]indol-9(6H)-yl)(naphthalen-2-yl)methanone (11)

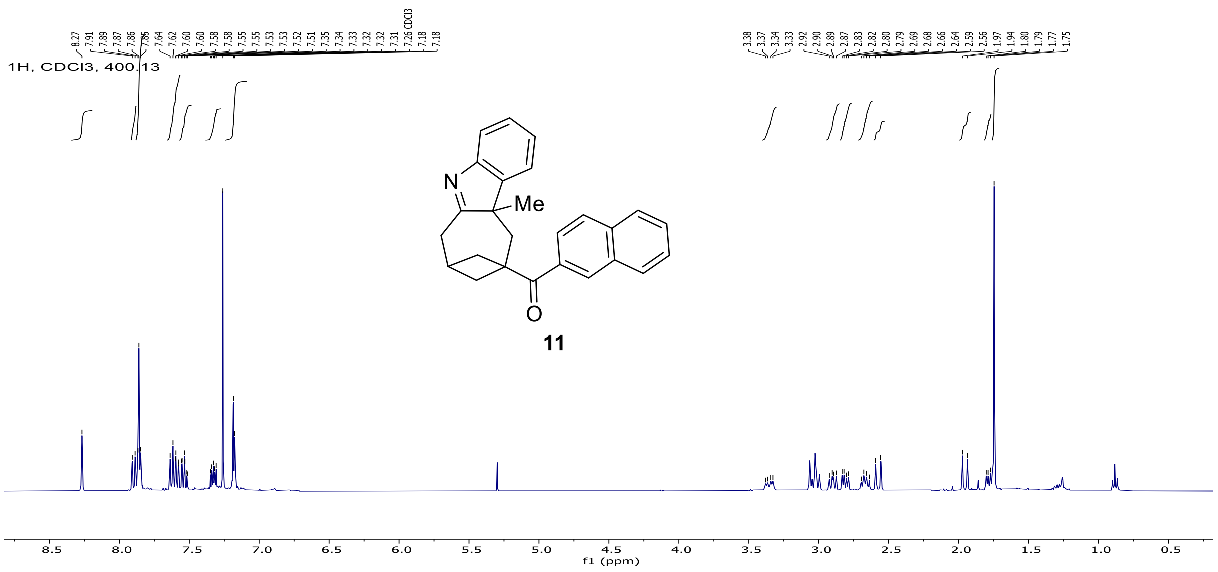

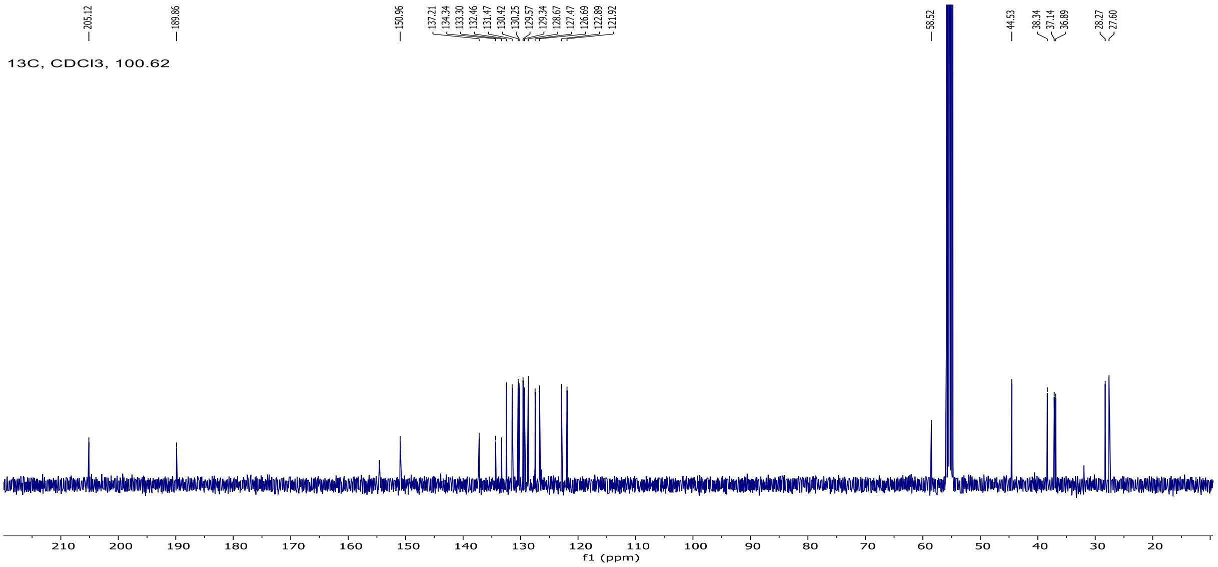

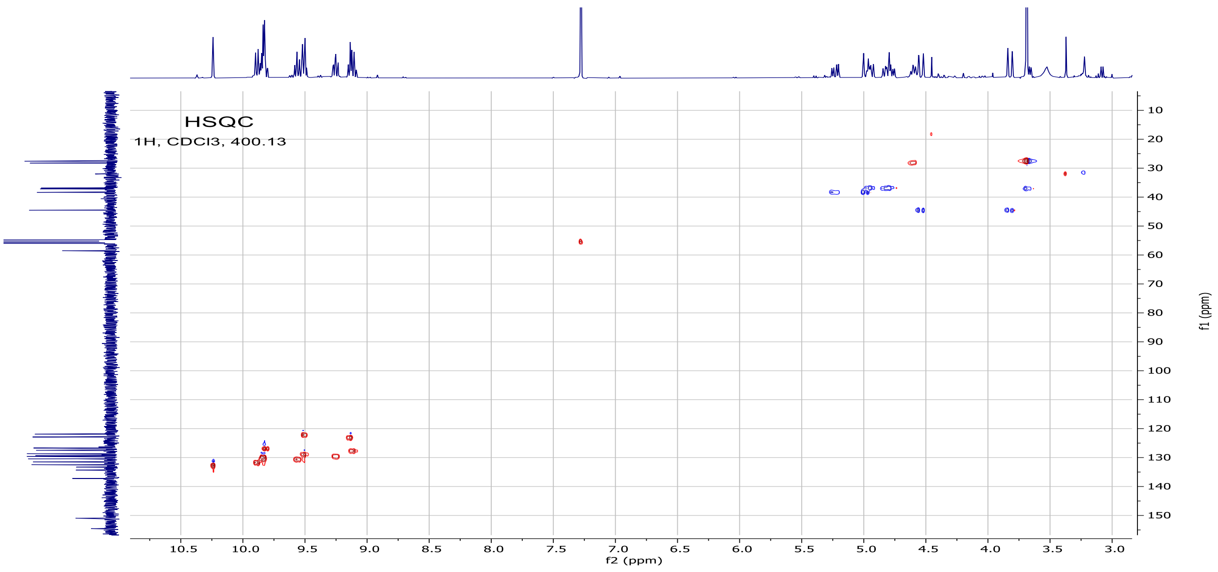

Supplement: SC-015-D4SC02767A-s001 [file SC-015-D4SC02767A-s001.pdf]
